# miRNA Digger: a comprehensive pipeline for genome-wide novel miRNA mining

**DOI:** 10.1038/srep18901

**Published:** 2016-01-06

**Authors:** Lan Yu, Chaogang Shao, Xinghuo Ye, Yijun Meng, Yincong Zhou, Ming Chen

**Affiliations:** 1 College of Life Sciences, Huzhou University, Huzhou 313000, P.R. China; 2 College of Life and Environmental Sciences, Hangzhou Normal University, Hangzhou 310036, P.R. China; 3 Department of Bioinformatics, College of Life Sciences, Zhejiang University, Hangzhou 310058, P. R. China

## Abstract

MicroRNAs (miRNAs) are important regulators of gene expression. The recent advances in high-throughput sequencing (HTS) technique have greatly facilitated large-scale detection of the miRNAs. However, thoroughly discovery of novel miRNAs from the available HTS data sets remains a major challenge. In this study, we observed that Dicer-mediated cleavage sites for the processing of the miRNA precursors could be mapped by using degradome sequencing data in both animals and plants. In this regard, a novel tool, miRNA Digger, was developed for systematical discovery of miRNA candidates through genome-wide screening of cleavage signals based on degradome sequencing data. To test its sensitivity and reliability, miRNA Digger was applied to discover miRNAs from four organs of *Arabidopsis*. The results revealed that a majority of already known mature miRNAs along with their miRNA*s expressed in these four organs were successfully recovered. Notably, a total of 30 novel miRNA-miRNA* pairs that have not been registered in miRBase were discovered by miRNA Digger. After target prediction and degradome sequencing data-based validation, eleven miRNA–target interactions involving six of the novel miRNAs were identified. Taken together, miRNA Digger could be applied for sensitive detection of novel miRNAs and it could be freely downloaded from http://www.bioinfolab.cn/miRNA_Digger/index.html.

MicroRNAs (miRNAs) are a class of small (18–25 nt in length) non-coding RNAs (ncRNAs) acting as negative regulators in gene expression. Previous studies showed that the regulatory activities of miRNAs could be involved in many biological processes, such as organ development^1^,^2^, metabolism^3^, immune response^4^ and tumorigenesis^5^,^6^. Certain miRNAs also showed great potential of being biomarkers for specific diseases^7^,^8^. In addition to mechanistic studies on already known miRNAs, discovery of novel miRNAs is becoming a major task and a hot research topic for thoroughly understanding of the miRNA world.

With the advances of high-throughput sequencing (HTS) techniques, a number of bioinformatics tools have been developed for systematical discovery of novel miRNAs based on different principles^9^, including the miRNA biogenesis mechanism^10^,^11^,^12^, the homology from known miRNAs^13^,^14^,^15^, the energy distribution pattern and Random Forest algorithm^16^. Although each of these approaches has its own merit and serviceability, several tools were included for performance comparison, in order to provide valuable information for selection of the ideal tool to meet different research needs^9^. However, accurate detection and quantification of these available tools are challenged by complex properties of the miRNAs and their precursors^17^, including the existence of miRNA variants (called isomRs), the secondary structures of the miRNA precursors^9^, and the Dicer-mediated processing of the miRNA precursors^18^,^19^. Therefore, thoroughly prediction and identification of the miRNAs, especially for the novel miRNAs, from the huge pool of HTS data remains a challenge for the following functional studies on miRNAs.

Degradome sequencing is a widely used approach for direct and global identification of miRNA-mediated target cleavages^20^,^21^,^22^. The miRNA-mediated cleavage sites could be mapped by using degradome signatures^20^, and a reversed framework for novel plant miRNA detection has been developed in our previous work, based on the degradome-supported miRNA cleavage loci and the highly complementarity of miRNA and the targets in plants^22^. Interestingly, several recent reports have showed that a portion of the degradome signatures could be mapped to the ends of the miRNA- and/or the miRNA*-coding loci on the miRNA precursors. Thus, it was speculated that these degradome signatures might be evidences for Dicer-mediated processing of the miRNA precursors^20^,^23^. However, no further investigation was conducted. In our previous work, mapping of degradome signatures onto the plant genomes has uncovered massive cleavage signals^22^. Intriguingly, a portion of the degradome signatures were found to locate on the known miRNA precursors. Although several miRNAs were indicated to regulate their own precursors by cleavages^24^, it could not explain the existence of the degradome signatures mapped to the ends of the miRNA- and/or the miRNA*-coding loci on the precursors. Further investigations revealed that a great number of degradome signatures on the newly identified miRNA precursors in *Arabidopsis* were resulted from DCL1 (Dicer-like 1)-mediated cleavages, suggesting that the processing of miRNA precursors could be supported by degradome signatures^25^. In this regard, degradome sequencing data could be utilized for the systematical identification of DCL1-dependent miRNA and miRNA* loci on their precursors in plants. Notably, as the miRNA-miRNA* duplexes are released by Dicer-mediated processing of miRNA precursors in animals^26^, the miRNA and miRNA* loci could also be supported by degradome signatures theoretically. In this scenario, the degradome-based evidences of Dicer-mediated cleavages could increase the reliability of systematical detection of novel miRNA loci in both animals and plants.

In this study, the miRNA precursors of *Mus musculus* and *Arabidopsis thaliana* registered in miRBase (release 21) were included for degradome-based scanning of Dicer-mediated cleavage signals. We demonstrated that Dicer-mediated processing of miRNA-miRNA* duplexes could be well supported by degradome signatures. Based on the results, a publicly available software, miRNA Digger, was developed for genomic-wide discovery of novel miRNA/miRNA* loci on specific precursors in plants and animals. To demonstrate its sensitivity and reliability, miRNA Digger was applied for the identification of already known and novel miRNAs from different tissues of *Arabidopsis*.

## Results

### Known miRNA/miRNA* loci were well supported by degradome signatures produced during Dicer-mediated processing of miRNA precursors

MiRNA biogenesis begins with the transcription of a primary miRNA (pri-miRNA). In animals, the pri-miRNA is recognized and cropped by RNase III enzyme Drosha to produce the pre-miRNA (precursor miRNA), and further processed by another RNase III enzyme, Dicer, to generate a miRNA-miRNA* duplex with 2-nt 3′ overhangs. In plants, this process is carried out by a single RNase III enzyme, Dicer-like 1 (DCL1)^26^. After processing, one strand (miRNA*) of the short duplex is typically degraded whereas the mature miRNA strand is incorporated into the RISC (RNA-induced silencing complex) to exert regulatory functions^27^.

According to the previous work, thousands of DCL1-dependent small RNA (sRNA) loci on their precursors were supported by degradome signatures in *Arabidopsis*^25^. We hypothesized that during the processing of the miRNA precursors, Dicer cut pri-miRNA at the 5′ and 3′ ends of miRNA and miRNA*, and release the processing intermediates which could be detected by degradome sequencing. In another word, the miRNA-miRNA* duplex loci within the corresponding precursors could be supported by degradome signatures.

To test this hypothesis, the known mature miRNAs and their corresponding pre-miRNAs of *Mus musculus* and *Arabidopsis thaliana* (miRBase release 21) were recruited. We took advantage of the degradome sequencing data sets from previous studies, including GSM561025, GSM561026, GSM561027, GSM561028, GSM561029 and GSM561030 of *Mus musculus* [retrieved from GEO (Gene Expression Omnibus); http://www.ncbi.nlm.nih.gov/geo/], and AxIDT_raw, AxIRP_raw, AxSRP_raw, Col_raw, ein5l_raw, TWF_summary and Tx4F_summary of *Arabidopsis* (retrieved from PARE (*Arabidopsis* Parallel Analysis of RNA Ends); http://mpss.udel.edu/at_pare/)^28^. The degradome data sets were pre-treated as described in “Materials and Methods”, and the degradome tags were mapped onto the pre-miRNAs of *Mus musculus* and *Arabidopsis thaliana* respectively for extraction of the degradome-based cleavage evidences (see details in “Materials and Methods”). As the Dicer-mediated cleavages locate at 5′ or 3′ ends of miRNAs and miRNA*s on the corresponding precursors, the degradome-supported Dicer cleavage loci could be detected by mapping the degradome tags to pre-miRNAs. Therefore, we searched for the miRNA/miRNA* loci with degradome signatures mapped at either 5′ or 3′ ends which were considered as the Dicer-mediated cropping sites.

**Figure 1:**
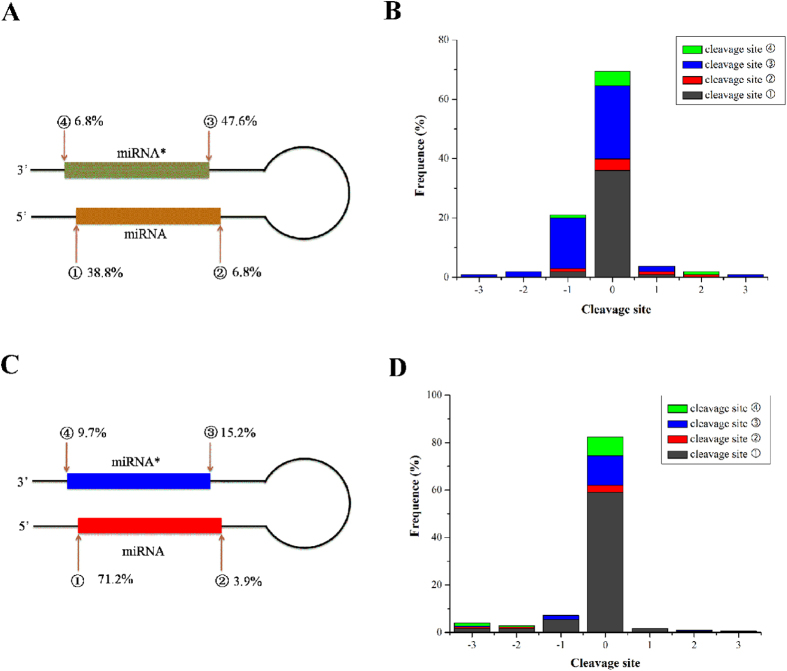
Statistic analysis of the miRNA-miRNA* duplex loci supported by degradome signatures. The frequency of degradome signatures occurred at different ends ① (5′ end of miRNA), ② (3′ end of miRNA), ③ (5′ end of miRNA*) and ④ (3′ end of miRNA*)(marked by red arrows) of recovered known miRNAs within the corresponding precursors in *Mus musculus* (**A**) and *Arabidopsis* (**C**) were estimated and present in the figures. If two or more degradome signatures were detected locating at 5′/3′ ends of a known miRNA-miRNA* duplex, then these cleavage sites were considered have equal distribution on the discovery of this miRNA-miRNA* duplex. The frequency of degradome signatures occurred at the migration sites of 5′/3′ ends of known miRNA-miRNA* duplexes on their pre-miRNAs in *Mus musculus* (**B**) and *Arabidopsis* (**D**) were evaluated and profiled. Each ends of duplexes were set as cleavage site “0”, and the migration sites located at its upstream are set as −1, −2 and −3, and downstream are set as +1,+2 and +3.

The results revealed that, 66 miRNA-miRNA* duplex loci including 123 known mature miRNAs in *Mus musculus*, corresponding to 96.1% of known miRNAs from the 69 corresponding precursors which contain detectable degradome signatures, were well supported by degradome signatures mostly located at the 5′ end of the miRNAs/miRNA*s (Fig. 1A, Supplementary Table S1). As shown in Fig. 1C and Supplementary Table S1, 140 miRNA-miRNA* duplex loci including 150 known mature miRNAs, corresponding to 74.3% of known miRNAs from 182 miRNA precursors which contain detectable degradome signatures, were found to be supported by the degradome signatures mostly located at the 5′ end of the miRNAs in *Arabidopsis* as well. However, a fraction of degradome signatures were found to offset from the 5′ or 3′ ends of miRNA-miRNA* duplexes by 1 to 3 nt (Fig. 1B,D). This phenomenon might be caused by Dicer-mediated alternative cropping of pre-miRNAs^17^. Taken together, the above results demonstrated that a great portion of the miRNA/miRNA* loci could be well supported by the degradome signatures.

### Overview of miRNA Digger pipeline

**Figure 2:**
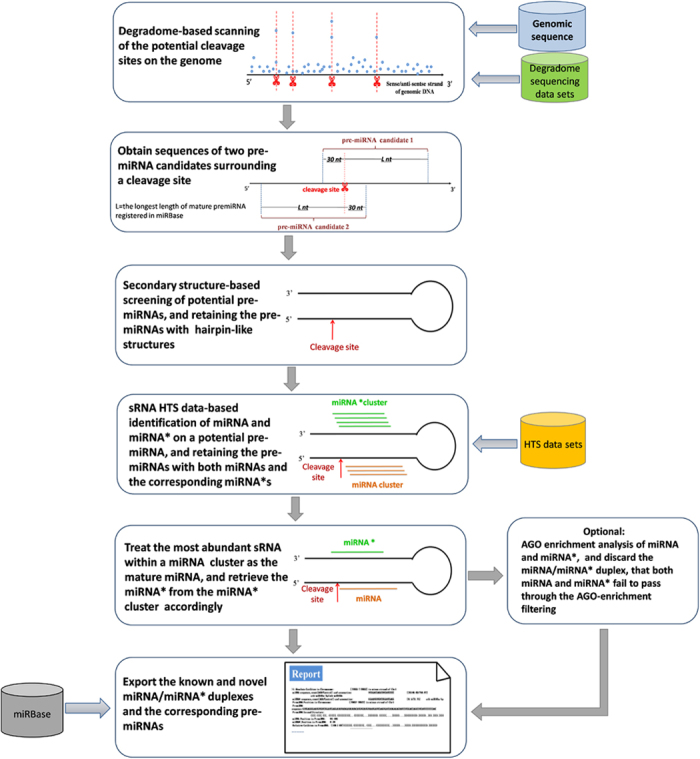
Workflow of miRNA Digger.

Based on the above observation, we developed miRNA Digger, a software for genome-wide mining of novel miRNA-miRNA* loci in animals and plants, by using the degradome-supported Dicer cropping sites as the “guidepost”. An overview of the miRNA Digger algorithm is shown in Fig. 2. Briefly, the miRNA Digger starts from the scanning of the degradome signatures on both sense and antisense strands of the genomic sequences. Then, it extracts potential miRNA precursors based on the degradome signatures followed by secondary structure prediction. Only the miRNA precursor candidates with hairpin-like structures are retained. Then, the sRNA short reads from HTS data sets are mapped onto these candidates to identify the miRNA-miRNA* duplexes. AGO enrichment analysis, an optional filtering step, is provided to extract AGO-associated miRNAs. Finally, miRNA Digger reports the known and novel miRNA-miRNA* duplexes and the corresponding pre-miRNAs.

In more details, miRDigger initially subdivides the genomic sequences into 10,000-nt fragments with 500-nt overlaps. The degradome tags are mapped onto the 10,000-nt fragments to obtain the degradome signatures(see “Extraction of the degradome signatures” in “Materials and Methods”)and their positions on the genome. Two pre-miRNA candidates of (30+L) nt in length will be obtained based on a single potential cleavage locus (Fig. 2). The secondary structures of each pre-miRNA candidates were predicted by using RNAfold. Based on the predictions, miRNA Digger retains the pre-miRNA candidates containing classic stem-loop structures (see “RNAfold-based secondary structure identification” in “Materials and Methods”).

Based on the biogenesis model of miRNAs, cropping of a precursor by Dicer could result in the production of a miRNA and a miRNA*^26^. And, slight variations at the Dicer recognition sites on miRNA precursors could result in the generation of miRNA-centered isomiR clusters along with miRNA*-centered isomiR* clusters^22^. In this regard, miRNA Digger retains potential pre-miRNAs containing detectable miRNA-miRNA* duplex (see “Screen of potential pre-miRNAs” in “Materials and Methods”).

It is well known that, miRNAs exert their regulatory function by incorporating into AGO-associated RISCs. However, several recent reports have revealed that a considerable number of AGO-associated miRNA*s have biological regulatory roles^20^,^22^,^29^. Therefore, if the AGO-associated sRNA HTS data sets are available, the optional step “AGO-enrichment analysis” is strongly recommended. Thus, miRNA Digger retains miRNA-miRNA* duplexes containing AGO-enriched miRNA(*)s, which can provide researchers with valuable hints for further investigations.

Finally, miRNA Digger outputs a report containing information about the pre-miRNA-coding loci on the genome (which can be used as their original identity), the degradome signatures loci on pre-miRNAs, the sequences and read counts (in RPM) of miRNAs and miRNA*s, the miRNA-miRNA* duplexes loci within the corresponding pre-miRNAs, and the sequences and the secondary structures of the pre-miRNAs. If a miRNA(*) sequence has been registered in miRBase, its name will be output simultaneously.

The miRNA Digger software package is available at http://www.bioinfolab.cn/miRNA_Digger/index.html. Additionally, it carries several auxiliary programs for data pre-processing. The sRNA HTS data sets and degradome sequencing data sets should be pre-treated by using the “HTS Data Process” option (see “pre-treatment of high-throughput sequencing data” in “Materials and Methods”) before they could be used for novel miRNA-miRNA* duplex digging. The downloaded chromosome sequences file should be separated into several files by using the “Chromosome Process” option (each file contains one chromosome sequence in fasta format). The “miRBase Update” option links to the miRBase Database, which can be used for downloading the latest version of registered mature miRNAs and pre-miRNAs of different species. MiRNA Digger is designed for Windows System (Vista, Win7 or advanced versions). As the degradome-based scanning of cleavage signals is a time-consuming task, several weeks might be required for scanning a single chromosome. Thus, in order to reduce the total running time, miRNA Digger is designed to run several algorithms in parallel. Additionally, if the running tasks are unfortunately break off under particular situation, miRNA Digger could continue the unfinished tasks when it is restarted.

### Case study: discovery of novel miRNAs from different tissues of *Arabidopsis*

To estimate the sensitivity of miRNA Digger, it was further employed for miRNA-miRNA* duplexes discovery from eight previous reported sRNA HTS data sets of *Arabidopsis*. Then all of the predicted novel miRNAs and miRNA*s were further recruited for the identification of miRNA–target interactions.

**Figure 3:**
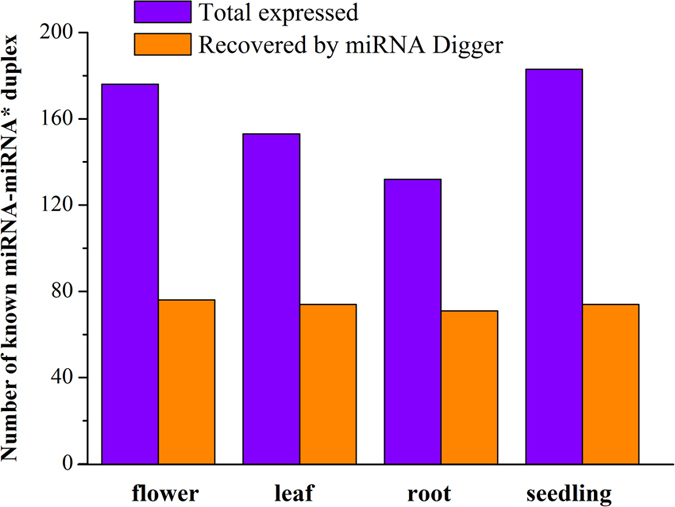
Recovery of expressed known miRNAs from different tissues of *Arabidopsis* by miRNA Digger. The total numbers of expressed known miRNA-miRNA* duplexes in different tissues of *Arabidopsis* were shown in blue columns and the numbers of recovered miRNA-miRNA* duplexes by miRNA Digger were shown in orange yellow columns.

**Figure 4:**
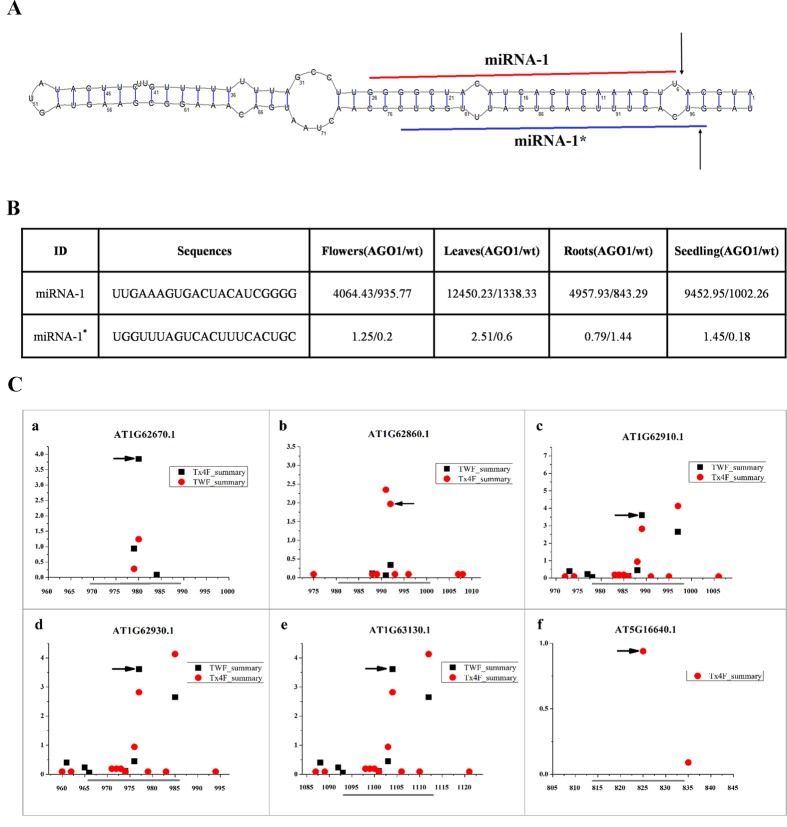
Discovery of novel miRNA from different tissues of *Arabidopsis*. The degradome-supported novel miRNA-1/miRNA-1* loci within the corresponding pre-miRNAs were showed in panel **A**. The miRNA-1 sequences was marked by red line the miRNA-1* was marked by blue line, and the degradome signature loci were marked by arrows. The expression level of miRNA-1/miRNA-1* in different tissues were listed in panel **B**. The degradome sequencing data-based validation of the miRNA-target interactions were showed in panel **C**, in which two libraries of degradome sequencing data libraries (TWF_summary and Tx4F_summary) were recruited for T-plot profiling (a to f). The IDs of the target transcripts were listed on the top. The *y* axes measured the normalized reads (in RMP, reads per million) of the degradome signals, and the *x* axes represented the position of the cleavage signals on the target transcripts. The binding sites of the miRNA-1 on its target transcripts were denoted by grey horizontal lines, and the dominant cleavage signals were marked by black arrows.

The sRNA HTS data sets from the flowers (six-week-old wild type plants, GSM707678; AGO1-associated sRNA, GSM707682), the leaves (four-week-old wild type plants, GSM707679; AGO1-associated sRNA, GSM707683), the roots (four-week-old wild type plants, GSM707680; AGO1-associated sRNA, GSM707684) and the seedlings (wild type seedlings, GSM707681; AGO1-associated sRNA, GSM707685) of *Arabidopsis* were contributed by Wang *et al.*^30^. The genomic DNA sequences of *Arabidopsis* were retrieved from the *Arabidopsis* Information Resource (TAIR, release 10). Eight degradome data sets (AxIDT_raw, AxIRP_raw, AxSRP_raw, Col_raw, ein5l_raw, TWF_summary and Tx4F_summary) were retrieved from PARE (http://mpss.udel.edu/at_pare/)^28^. The sRNA HTS and degradome sequencing data sets were pre-treated by using the “HTS Data Process” option, and the genomic DNA sequences (chromosome 1 to chromosome 5) were pre-processed by using the “Chromosome Process” option. Then, all of the pre-treated data sets were imported into miRNA Digger. As a result, 76 out of 176, 74 out of 153, 71 out of 132, and 74 out of 183 expressed known miRNA-miRNA* duplexes were identified from the flowers, leaves, roots and seedlings, respectively (Fig. 3 and supplementary table S2). It indicates that a large portion of the expressed miRNA-miRNA* duplexes could be uncovered by miRNA Digger. Notably, although these sRNA HTS data sets have been intensively mined for miRNAs^30^,^31^,^32^ miRNA Digger still discovered 24, 25, 21 and 22 novel miRNA-miRNA* duplexes in flowers, leaves, roots and seedlings, respectively (Fig. 4, supplementary table S3 and supplementary figure S1).

All of the miRNAs and miRNA*s from these newly discovered miRNA-miRNA* duplexes were recruited for target prediction by using miRU algorithm^33^, and then followed by the degradome-based identification of miRNA–target interactions^20^,^22^. As a result, 11 novel miRNA–target interactions involving six novel miRNAs were validated (Table 1 and Supplementary Figure S2).

Among them, miRNA-1 was found to target an RNA processing factor 2 (RPF2) gene (AT1G62670.1), three *PPR* genes (AT1G62860.1, AT1G62910.1 and AT5G16640.1) and two *TPR* genes(AT1G62930.1 and AT1G63130.1). The RPF2 protein has been revealed involving in the generation of a distinct 5′ terminus of transcripts of subunit 9 of the NADH DEHYDROGENASE complex (nad9), and RPF2 also determines the efficiency of 5′ end formation of the mRNAs for subunit 3 of the CYTOCHROME C OXIDASE (cox3), in *Arabidopsis*^34^. The TRP and PPR proteins are wide-spread on the *Arabidopsis* and regulate RNA maturation, additionally, the TPR proteins are also considered to function in protein—protein interaction^35^. miRNA-2 was detected targeting an Argonaute 2 (AGO2) encoding gene, AT1G31280.1, which has been revealed play important role in antibacterial immune responses in *Arabidopsis* by Jin and his teammates^36^,^37^.

miRNA-3 was detected to target an mRNA capping enzyme family protein encoding gene, AT3G09100.1. Eukaryotic mRNA capping enzymes are bifunctional, carrying both RNA triphosphatase (RTPase) and guanylyltransferase (GTase) activities, which is essential for gene expression^38^,^39^.

The transposable element gene AT5G33223.1, which contributes to plant gene and genome evolution^40^, was found to be regulated by miRNA-4. miRNA-5 regulates a MKK9 gene which has been revealed act as a negative regulator of the abiotic stress response in *Arabidopsis*. miRNA-6 was found to target genes (AT3G54990.1, AT4G36920.1, AT5G04275.1, AT5G60120.1 and AT5G60120.2) involving in plant development, AT3G54990.1 and AT4G36920.1 encoding integrase-type DNA-binding superfamily protein, which has been revealed may regulating the progression of plant developmental cycle^41^. miR172b (AT5G04275.1) controls the Transition to Autotrophic Development of *Arabidopsis*^42^. The targets of early activation tagged (EAT)2, AT5G60120.1 and AT5G60120.2 have been revealed involving in flowering and change of phenotype of *Arabidopsis*^43^.

The above results indicated that miRNA Digger could successfully recovered the known miRNA-miRNA* duplexes and the corresponding pre-miRNAs with reasonable sensitivity (52–61%). Secondly, by recruiting HTS data sets, miRNA Digger could perform genomic-wide scan to search for the novel miRNA-miRNA* duplexes. Finally, although the data sets used have already been specifically mined for miRNA^22^,^30^, miRNA Digger still predicts 30 novel miRNA-miRNA* duplexes, and six of them were identified to play important regulatory functions in *Arabidopsis*. Therefore, miRNA Digger is a sensitive and reliable tool for genome-wide prediction of novel miRNA-miRNA* duplexes.

**Figure 5:**
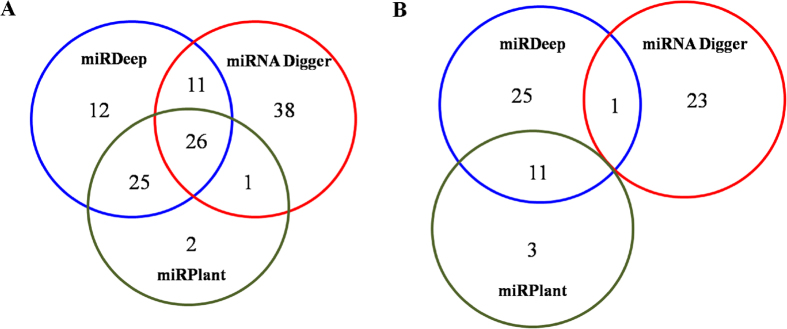
Venn diagram showing the comparison of results produced by miRNA Digger, miRDeep and miRPlant. The number of uncovered known (**A**) and novel (**B**) miRNA-miRNA* pairs by miRNA Digger, miRDeep and miRPlant were profiled by venn diagram.

miRDeep is a popular software for miRNA detection based on the biological model of Dicer-mediated processing of the miRNA precursors^44^. miRPlant is a plant-specific tool for miRNA discovery based on the similar algorithm^45^. In order to test the functionality of miRNA Digger, we compared all of the miRNAs discovered by miRNA Digger from the flowers of *Arabidopsis* (Supplementary table S2a and S3a) with those reported by miRDeep and miRPlant with an additional AGO1-enrichment filter step (Supplementary table S4 and S5). As a results, 76, 74 and 54 known miRNA-miRNA* pairs, were recovered by miRNA Digger, miRDeep and miRPlant, respectively. However, only 26 known pairs were recovered by all three tools, and 38 known pairs were specifically recovered by miRNA Digger (Fig. 5A). The results indicated that, miRNA Digger presented a different sensitivity and specificity for miRNA detection by relying on the algorithm of finding degradome-supported Dicer cleavage sites. Additionally, 24, 38 and 14 novel miRNA-miRNA* pairs were also discovered by miRNA Digger, miRDeep and miRPlant, respectively. However, no miRNA candidates were discovered by all these three tools (Fig. 5B).

## Discussion

Various bioinformatics tools have been developed for systematical detection of novel miRNAs, however, these available tools appeared diverse sensitivity and specificity in miRNA detection among different species^9^,^44^. In this study, we proposed a novel algorithm for detection of miRNA-miRNA* duplexes from sRNA HTS data based on the genomic-wide scanning of degradome-supported Dicer/DCL1 cropping sites on genome of animals/plants. Although similar with miRNA Digger that, miRDeep and miRPlant discovers miRNAs based on the processing mechanism of the miRNA precursors by Dicer, they are utterly dependent on the sRNA HTS data sets. However, our approach has the virtue of finding evidence of Dicer-mediated cleavage on miRNA precursors, which reduces the interference of other non-Dicer processed sRNAs and provides a different sensitivity and specificity for miRNA discovery. Therefore, the algorithm of miRNA Digger is a good complement of those previous algorithms with respect to digging miRNA genes from the available sRNA HTS data. Additionally, as it is the first time that the degradome-supported Dicer cleavage sites scanning was reported integrating into the core heart of an algorithm for miRNA detection, we believe that, compared with the previous reported algorithms, miRNA Digger can discover the miRNA-miRNA* duplexes more confidently with this additional criteria.

The sensitivity of miRNA Digger is also determined by the availability and depth of degradome sequencing data and sRNA HTS data. Therefore, miRNA Digger is database-supported, and the advances in the field of next-generation sequencing will greatly contribute to the efficiency of miRNA Digger. Additionally, as the application of degradome sequencing technology focused on the validation of miRNA-target interaction previously^20^,^21^,^22^,^23^,^24^, the construction and application of miRNA Digger for miRNA mining significantly expanded its application range.

Further improvements of miRNA Digger will be performed in our future work including the enhancement of the rate of degradome-based cleavage signals scanning by integrating with other algorithms to reduce the whole processing time for miRNA discovery. Besides, miRNA Digger is designed only for genome scanning in this study, further improvement will allow the input of expressed sequence tag (EST) for analysis, when no genome sequencing is available.

## Conclusion

We developed a novel tool, miRNA Digger, for miRNA detection from different samples of animals and plants, based on the evidence that, the Dicer/DCL1 cropping sites on pre-miRNAs can be well supported by the degradome signatures. miRNA Digger was tested and proved to capable of both recovering the majority of known miRNA-miRNA* duplexes present in heterogeneous samples, and reporting novel miRNAs with high confidence, indicating that, miRNA Digger can be widely utilized for novel miRNA discovery and investigation of gene regulation mechanism in both animals and plants.

## Materials and Methods

### Pre-treatment of high-throughput sequencing data sets

The short sequences containing ‘N’s or with ‘0’ read counts were discarded, and then the read count of each short sequence was normalized and measured with reads per million (PRM) as described previously^21^, to enable the cross-comparison between different libraries.

### Extraction of the degradome signatures

The following criteria are applied to extract the degradome-based cleavage signals on genome: 1) for the degradome tags from one library, the averaged read count (in RPM) of all the degradome tags with their 5′-ends mapped to a potential cleavage site should be ‘five times or more’^22^,^46^ than that mapped to the regions surrounding this cleavage site; 2) for the tags from the same degradome library, among the degradome tags mapped to a potential cleavage site, the most abundant tag should be among the top 12 most abundant degradome tags that perfectly matched to the corresponding 10000-nt fragment^19^.

### Extraction of pre-miRNA candidates

All of the pre-miRNAs registered in miRBase have been deposited into miRNA Digger, which can be periodically updated. Then, the length of the longest pre-miRNA (the parameter “L”) was calculated for each plant or animal species. When using miRNA Digger, the selection of a species can automatically result in a search for pre-miRNAs by using the default length parameter “L”. Dicer-mediated cleavages probably locate at either end of the miRNA(*), and the length of the mature miRNAs of animals and plants are less than 30 nt^25^. In this consideration, for the 3′ armed miRNA(*), if a potential cleavage site (degradome-based cleavage signal) locates at the 5′or 3′ end of the miRNA(*), the pre-miRNA candidate will be included within the downstream “L”-nt region plus the 30-nt upstream region. And, for the 5′ armed miRNA(*), if the potential cleavage site locates at the 5′or 3′ end of the miRNA(*), the pre-miRNA candidate will be included within the upstream “L”-nt region plus the 30-nt downstream region. Thus, based on a single potential cleavage locus, two pre-miRNA candidates of (30+L) nt in length will be obtained for further screening.

### RNAfold-based secondary structure identification

**Figure 6:**
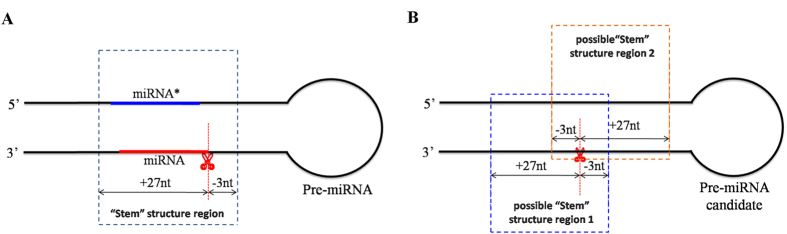
The “Stem” structure region of pre-miRNA. The 30 nt “Stem” structure regions of the known pre-miRNAs (**A**) and two possible “Stem” structure regions (30 nt in length) of the premiRNA candidate (**B**) were determined by using the degradome-supported cleavage site (marked by scissor) as the guidepost.

RNAfold is recruited to predict the thermodynamically optimal secondary structure (minimum free energy secondary structure) of all pre-miRNA candidates^47^. As we do not know the true 5′ and 3′ ends of pre-miRNA candidates, the unrelated termini of the respective sequences which do not belong to the true pre-miRNA, which might cause the pre-miRNA structure to be thermodynamically suboptimal. Therefore, the pre-miRNA candidates were further employed for base complementarity analysis. As we do not know the precisely position of miRNA/miRNA* within a pre-miRNA candidates, two possible “Stem” structure regions (30nt in length) of a single miRNA precursor candidates around the cleavage locus were analyzed (Fig. 6), if one of them possesses complementarity higher than a setting value (adjustable parameter: the default value is set as 70%), the miRNA precursor candidate was then retained.

The default value of complementarily filtering was set based on the analysis of all “Stem” structure regions (30 nt in length including miRNA-miRNA*) of the known pre-miRNAs of *Arabidopsis* (miRBase release 21), and we found that, about 95% (404 out of 427) of them possess complementarity higher than 70%. This value can be optimized and adjusted to cart the need of researchers with their interest of miRNAs in different species.

### Screen of potential pre-miRNAs

miRNA Digger retains the potential pre-miRNA with detectable miRNA cluster and the corresponding miRNA* cluster. The most abundant miRNA within a miRNA cluster is treated as mature miRNA, and the position of its corresponding miRNA* is then determined based on the structurally featured 2-nt 3′ overhangs of the miRNA-miRNA* duplex^25^. If no such canonical miRNA* is detected within the miRNA* cluster, which might result from fast degradation of miRNA* after Dicer-mediated processing, then the most abundant isomiR* sequence within the miRNA* cluster is treated as the counterpart of mature miRNA. Thus, the filtered pre-miRNA sequences encoding the miRNA and miRNA* sequences, along with 5-nt additional sequence at both 5′ and 3′ ends is obtained.

### AGO-enrichment filter

For optional AGO-enrichment filter, the read count of each AGO-associated miRNA should above a setting value (adjustable parameter: the default value is set as 1RPM) and the AGO-enriched miRNA should be several times or more (adjustable parameter: the default parameter is set as 3) abundant than that of the control^21^.

### Compare with miRDeep and miRPlant

The sRNA HTS data sets from the flowers (GSM707678, GSM707682)^30^ and degradome data sets (AxIDT_raw, AxIRP_raw, AxSRP_raw, Col_raw, ein5l_raw, TWF_summary and Tx4F_summary) (http://mpss.udel.edu/at_pare/)^28^ were pre-treated by using the “HTS Data Process” and the genomic DNA sequences of *Arabidopsis* (TAIR, release 10) were pre-treated by using the “Chromosome Process” option. Then, all of them were imported into miRNA Digger for analysis.

The pre-treated sRNA HTS data sets of AGO1-associated sRNA in flowers (GSM707678) and the genomic DNA sequences of *Arabidopsis* (TAIR, release 10) were imported into miRDeep and miRPlant for analysis. The reported miRNA candidates were further employed for an additional AGO-enrichment filter (select the same enrichment filter parameters as those of miRNA Digger) by using the pre-treated sRNA HTS data set GSM707682 (flower tissues in Col-0) as control.

## Additional Information

**How to cite this article**: Yu, L. *et al.* miRNA Digger: a comprehensive pipeline for genome-wide novel miRNA mining. *Sci. Rep.* **6**, 18901; doi: 10.1038/srep18901 (2016).

## Supplementary Material

Supplementary Information

## Figures and Tables

**Table 1:** The targets of novel miRNAs in *Arabidopsis*.

Novel miRNA ID	Novel miRNA sequence	Target ID	Predicted binding site(s) on the target transcript	Cleavage signals detected by degradome sequencing data	targets annotation
miRNA-1	UUGAAAGUGACUACAUCGGGG	AT1G62670.1	969–989	980	RNA processing factor 2 (RPF2)
		AT1G62860.1	981–1001	992	Pseudogene of pentatricopeptide (PPR) repeat-containing protein
		AT1G62910.1	978–998	989	Pentatricopeptide repeat (PPR) superfamily protein
		AT1G62930.1	966–986	977	Tetratricopeptide repeat (TPR)-like superfamily protein
		AT1G63130.1	1093–1113	1104	Tetratricopeptide repeat (TPR)-like superfamily protein
		AT5G16640.1	814–834	825	Pentatricopeptide repeat (PPR) superfamily protein
miRNA-2	UUAGAUUCACGCACAAACUCGU	AT1G31280.1	3221–3242	3233	Argonaute family protein(AGO2)
miRNA-3	AAAUGAGUUGAUGGGUCAAAUGAG	AT3G09100.1	1095–1117	1108	mRNA capping enzyme family protein
miRNA-4	UCUUUUCUUCCUUGUGCUCGAG	AT5G33223.1	568–589	580	transposable element gene
miRNA-5	CGGCGAUGAUGAUGAAACAAGAU	AT1G73500.1	127–149	140	ATMKK9
		AT3G54990.1	965–985	976	Integrase-type DNA-binding superfamily protein(SMZ)
		AT4G36920.1	1329–1349	1340	Integrase-type DNA-binding superfamily protein (AP2)
miRNA-6	UGAAUCUUAAUGGUGCUGCAU	AT4G36920.2	1293–1313	1304	Integrase-type DNA-binding superfamily protein (AP2)
		AT5G04275.1	1–21	12	MIR172B
		AT5G60120.1	1647–1667	1658	target of early activation tagged (EAT) 2
		AT5G60120.2	1810–1830	1821	target of early activation tagged (EAT) 2
