# Supplementary material for: miRNA Digger: a comprehensive pipeline for genome-wide novel miRNA mining
Source: Sci Rep. 2016 Jan 6;6:18901. doi: 10.1038/srep18901 (PMC4702050; doi:10.1038/srep18901)
Supplement: Supplementary Information [file srep18901-s1.pdf]

miRNA Digger: a comprehensive pipeline for genome-wide novel miRNA mining

Lan Yu[1,&], Chaogang Shao[1,&], Xinghuo Ye[1], Yijun Meng[2,*], Yincong Zhou[3], Ming Chen[3,*]

1 College of Life Sciences, Huzhou University, Huzhou 313000, P.R. China

2 College of Life and Environmental Sciences, Hangzhou Normal University, Hangzhou 310036, P.R. China

3 Department of Bioinformatics, College of Life Sciences, Zhejiang University, Hangzhou 310058, P. R. China

& These authors contributed equally to this work.

***Corresponding authors:**

Yijun Meng

College of Life and Environmental Sciences, Hangzhou Normal University

Xuelin Street 16#, Xiasha, Hangzhou 310036, P. R. China

Tel: +86-571-28865198

E-mail: mengyijun@zju.edu.cn

Ming Chen

Department of Bioinformatics, College of Life Sciences, Zhejiang University, Zijingang Campus, Yu Hang Tang Road 866#, Hangzhou 310058, P. R. China

Tel: +86-571-88206612

Fax: +86-571-88206612

E-mail: mchen@zju.edu.cn

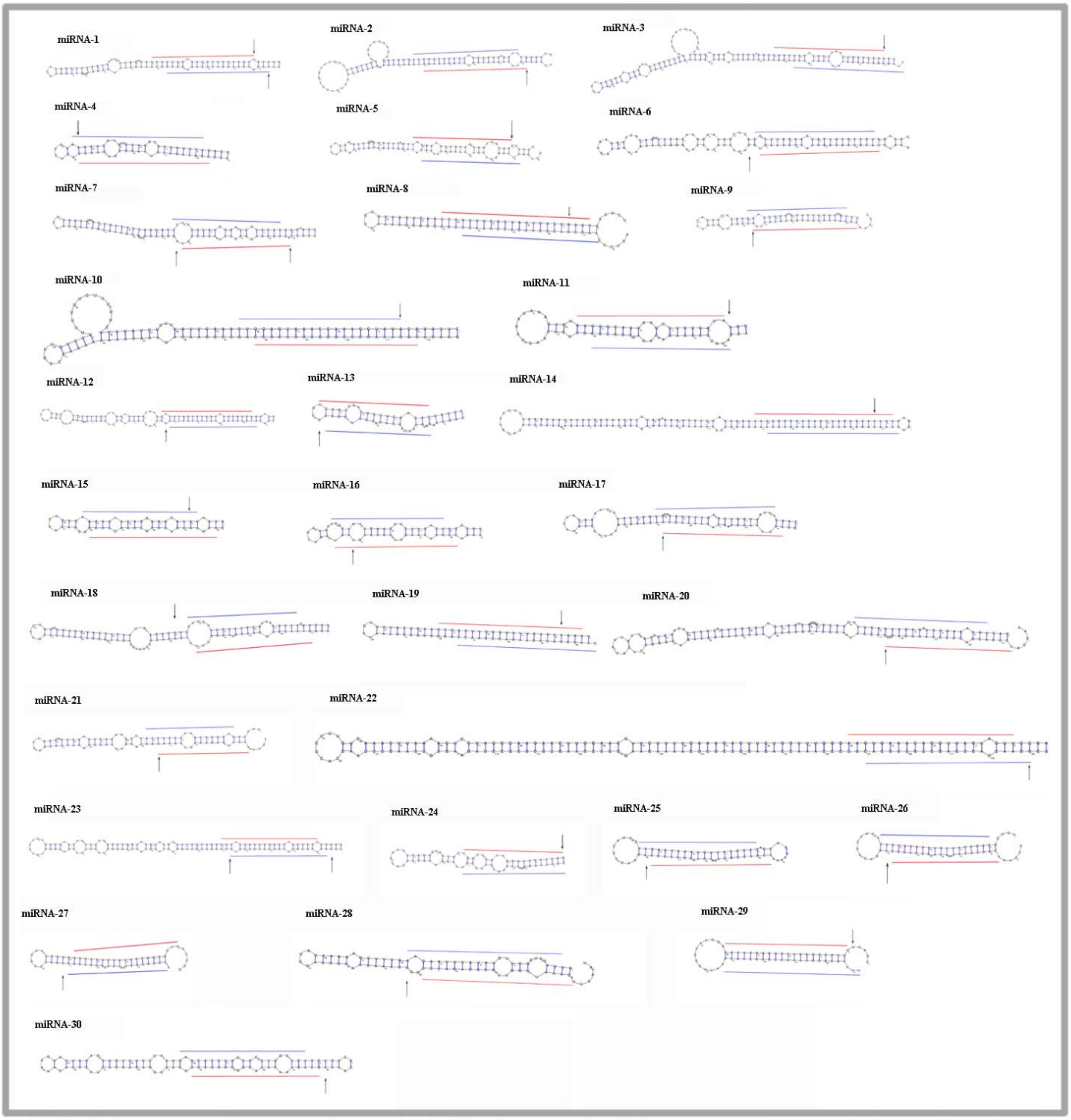

Supplementary figure S1. Degradome-supported novel miRNA-miRNA* duplexes loci within the corresponding pre-miRNAs in *Arabidopsis*.

The mature miRNAs sequences were marked by red lines and their miRNA* counterparts were marked by blue lines. The degradome signature loci were marked by arrows.

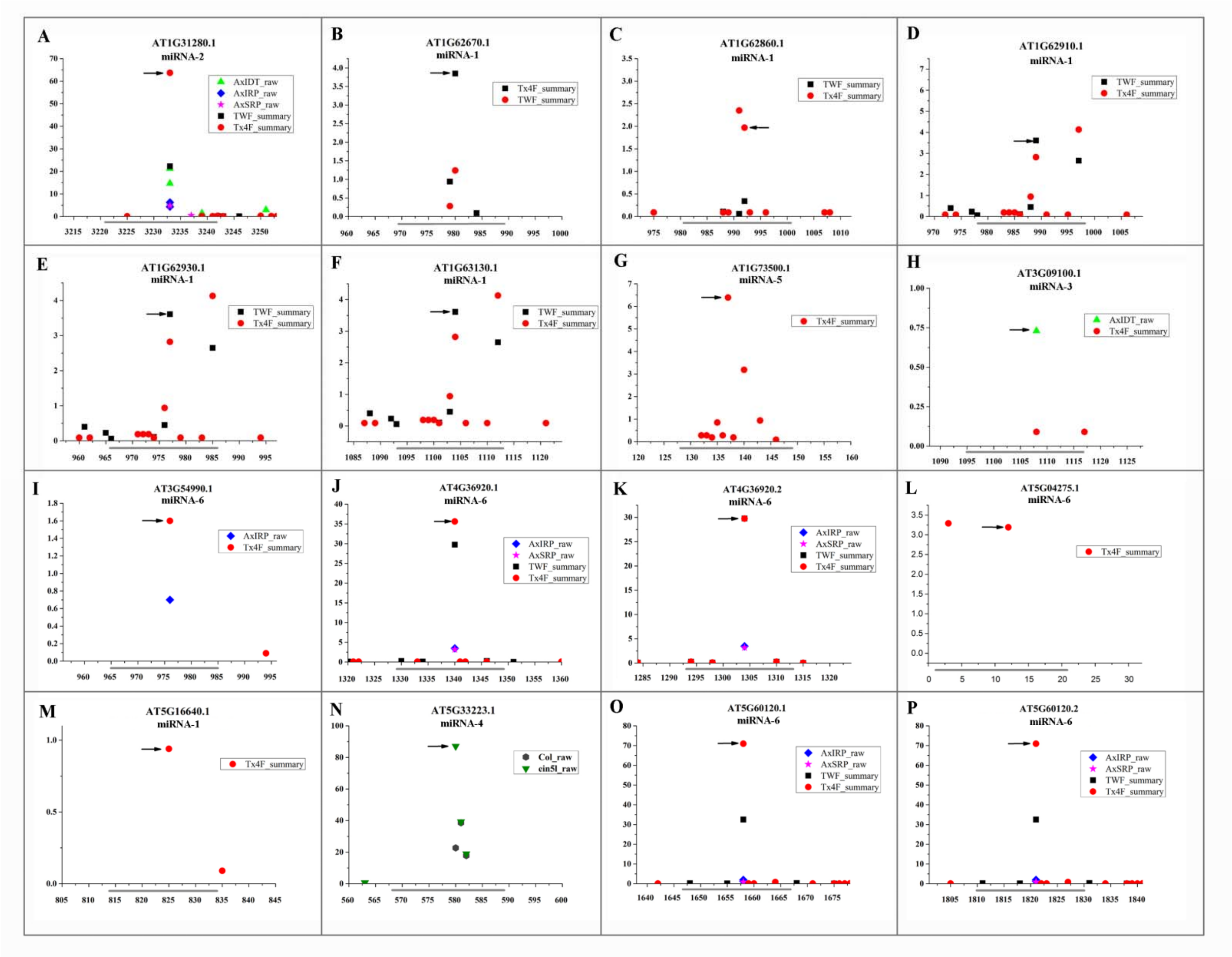

Supplementary figure S2. Degradome sequencing data-based validation of the miRNA--targets interactions in *Arabidopsis*.

Seven libraries of degradome sequencing data libraries (AxIDT_raw, AxIRP_raw, AxSRP_raw, Col_raw, ein5l_raw, TWF_summary and Tx4F_summary) were recruited for T-plot profiling (A to P)**.** The IDs of the target transcripts and the corresponding phased siRNAs are listed on the top. The *y* axes measure the normalized reads (in RMP, reads per million) of the degradome signals, and the *x* axes represent the position of the cleavage signals on the target transcripts. The binding sites of the novel miRNAs on their target transcripts were denoted by grey horizontal lines, and the dominant cleavage signals were marked by black arrows.

Supplementary table S1a. The loci of degradome signatures and known miRNAs/miRNAs* within the corresponding miRNA precursors in *Mus musculus*

PremiRNA_Name	PARE_CutSites	miRNA_Name	miRNA_Position(Start-End)	miRNA*_Position(Start-End)	PARE_CutSites vs. miRNA/miRNA*_terminal_shifting
mmu-let-7b	7	mmu-let-7b-5p	7-28	61-82	miRNA_5'_0
		mmu-let-7b-3p	61-82	7-28	miRNA*_5'_0
mmu-let-7e	15	mmu-let-7e-5p	15-36	60-81	miRNA_5'_0
		mmu-let-7e-3p	60-81	15-36	miRNA*_5'_0
mmu-let-7g	7	mmu-let-7g-5p	7-28	64-85	miRNA_5'_0
		mmu-let-7g-3p	63-84	8-29	miRNA*_5'_-1
mmu-mir-122	6	mmu-miR-122-5p	6-27	42-63	miRNA_5'_0
		mmu-miR-122-3p	41-60	9-28	miRNA*_5'_-3
mmu-mir-125a	6	mmu-miR-125a-5p	6-29	44-65	miRNA_5'_0
		mmu-miR-125a-3p	44-65	6-29	miRNA*_5'_0
mmu-mir-139	7	mmu-miR-139-5p	7-28	44-65	miRNA_5'_0
		mmu-miR-139-3p	43-64	8-29	miRNA*_5'_-1
mmu-mir-152	47	mmu-miR-152-3p	47-67	9-31	miRNA_5'_0
		mmu-miR-152-5p	8-31	47-68	miRNA*_5'_0
mmu-mir-155	4	mmu-miR-155-5p	4-26	44-64	miRNA_5'_0
		mmu-miR-155-3p	43-63	5-27	miRNA*_5'_-1
mmu-mir-17	14	mmu-miR-17-5p	14-36	58-72	miRNA_5'_0
		mmu-miR-17-3p	51-72	15-35	miRNA*_5'_-1
mmu-mir-186	7	mmu-miR-186-5p	7-28	47-67	miRNA_5'_0
		mmu-miR-186-3p	46-67	7-29	miRNA*_5'_0
mmu-mir-191	7	mmu-miR-191-5p	7-29	49-70	miRNA_5'_0
		mmu-miR-191-3p	49-70	7-29	miRNA*_5'_0
mmu-mir-195a	21	mmu-miR-195a-5p	21-41	60-81	miRNA_5'_0
		mmu-miR-195a-3p	59-80	22-42	miRNA*_5'_-1
mmu-mir-20a	27	mmu-miR-20a-5p	27-49	63-85	miRNA_5'_0
		mmu-miR-20a-3p	63-84	28-49	miRNA*_5'_-1
mmu-mir-22	57	mmu-miR-22-3p	57-78	19-40	miRNA_5'_0
		mmu-miR-22-5p	19-40	57-78	miRNA*_5'_0

mmu-mir-223	68	mmu-miR-223-3p	68-89	27-54	miRNA_5'_0
		mmu-miR-223-5p	26-48	80-90	miRNA_5'_0
mmu-mir-23a	46	mmu-miR-23a-3p	46-66	12-31	miRNA_5'_0
		mmu-miR-23a-5p	10-31	46-68	miRNA*_5'_0
mmu-mir-23b	46	mmu-miR-23b-3p	46-66	11-29	miRNA_5'_0
		mmu-miR-23b-5p	9-29	46-68	miRNA*_5'_0
mmu-mir-24-1	6	mmu-miR-24-1-5p	6-28	44-65	miRNA_5'_0
		mmu-miR-24-1-3p	44-65	6-28	miRNA_5'_0
mmu-mir-26b	15	mmu-miR-26b-5p	15-35	52-71	miRNA_5'_0
		mmu-miR-26b-3p	51-72	14-36	miRNA*_5'_+1
mmu-mir-27a	56	mmu-miR-27a-3p	56-76	14-35	miRNA_5'_0
		mmu-miR-27a-5p	14-35	56-76	miRNA*_5'_0
mmu-mir-27b	49	mmu-miR-27b-3p	49-69	7-29	miRNA_5'_0
		mmu-miR-27b-5p	7-28	50-69	miRNA*_5'_-1
mmu-mir-29c	16	mmu-miR-29c-5p	16-37	56-77	miRNA_5'_0
		mmu-miR-29c-3p	54-75	18-39	miRNA*_5'_-2
mmu-mir-30a	6/47	mmu-miR-30a-5p	6-27	49-68	miRNA_5'_0/miRNA*_5'_-2
		mmu-miR-30a-3p	47-68	6-29	miRNA_5'_0/miRNA*_5'_0
mmu-mir-320	48	mmu-miR-320-3p	48-69	16-37	miRNA_5'_0
		mmu-miR-320-5p	16-37	48-69	miRNA*_5'_0
mmu-mir-34a	20	mmu-miR-34a-5p	20-41	64-85	miRNA_5'_0
		mmu-miR-34a-3p	64-84	21-41	miRNA*_5'_-1
mmu-mir-451a	17	mmu-miR-451a	17-38	45-58	miRNA_5'_0
mmu-mir-484	5	mmu-miR-484	5-26	33-65	miRNA_5'_0
mmu-mir-652	61	mmu-miR-652-3p	61-81	21-45	miRNA_5'_0
		mmu-miR-652-5p	21-44	62-81	miRNA*_5'_-1
mmu-mir-674	24/25	mmu-miR-674-5p	25-46	60-81	miRNA_5'_0
		mmu-miR-674-3p	60-81	25-46	miRNA*_5'_0
mmu-mir-676	55	mmu-miR-676-3p	55-75	19-40	miRNA_5'_0
		mmu-miR-676-5p	18-39	56-76	miRNA*_5'_-1
mmu-mir-98	16	mmu-miR-98-5p	16-37	74-95	miRNA_5'_0
		mmu-miR-98-3p	74-95	16-37	miRNA*_5'_0
mmu-mir-99b	7	mmu-miR-99b-5p	7-28	45-66	miRNA_5'_0

		mmu-miR-99b-3p	45-66	7-28	miRNA*_5'_0
mmu-mir-1198	102	mmu-miR-1198-3p	80-101	42-63	miRNA_3'_0
		mmu-miR-1198-5p	42-63	80-101	miRNA*_3'_0
mmu-mir-1b	42	mmu-miR-1b-5p	41-61	82-103	miRNA_5'_+1
		mmu-miR-1b-3p	80-102	42-63	miRNA*_5'_0
mmu-mir-339	74	mmu-miR-339-3p	51-73	16-38	miRNA_3'_0
		mmu-miR-339-5p	16-38	51-73	miRNA*_3'_0
mmu-mir-5099	45	mmu-miR-5099	46-65	14-32	miRNA_5'_-1
mmu-mir-199b	87	mmu-miR-199b-3p	65-86	26-48	miRNA_3'_0
		mmu-miR-199b-5p	26-48	65-86	miRNA*_3'_0
mmu-mir-671	79	mmu-miR-671-3p	58-78	19-42	miRNA_3'_0
		mmu-miR-671-5p	19-41	59-78	miRNA*_3'_0
mmu-let-7a-1	13	mmu-let-7a-1-3p	64-85	12-34	miRNA*_5'_+1
		mmu-let-7a-1-5p	12-34	64-85	miRNA*_5'_+1
mmu-let-7a-2	17	mmu-let-7a-2-3p	62-82	18-38	miRNA*_5'_-1
		mmu-let-7a-2-5p	18-38	62-82	miRNA*_5'_-1
mmu-let-7f-1	8	mmu-let-7f-1-3p	64-85	8-29	miRNA*_5'_0
		mmu-let-7f-1-5p	8-29	64-85	miRNA*_5'_0
mmu-let-7f-2	8	mmu-let-7f-2-3p	58-78	9-29	miRNA*_5'_0
		mmu-let-7f-2-5p	9-29	58-78	miRNA*_5'_0
mmu-mir-16-1	16	mmu-miR-16-1-3p	58-79	17-37	miRNA*_5'_-1
		mmu-miR-16-1-5p	17-37	58-79	miRNA*_5'_-1
mmu-mir-16-2	17	mmu-miR-16-2-3p	59-80	18-39	miRNA*_5'_-1
		mmu-miR-16-2-5p	18-39	59-80	miRNA*_5'_-1
mmu-mir-1a-1	49	mmu-miR-1a-1-5p	10-32	49-70	miRNA*_5'_0
		mmu-miR-1a-1-3p	49-70	10-32	miRNA*_5'_0
mmu-mir-1a-2	47	mmu-miR-1a-2-5p	8-30	47-68	miRNA*_5'_0
		mmu-miR-1a-2-3p	47-68	8-30	miRNA*_5'_0
mmu-mir-24-2	83	mmu-miR-24-2-5p	25-46	61-82	miRNA*_3'_0
		mmu-miR-24-2-3p	61-82	25-46	miRNA*_3'_0
mmu-mir-30c-1	17	mmu-miR-30c-1-3p	56-77	17-40	miRNA*_5'_0
		mmu-miR-30c-1-5p	17-40	56-77	miRNA*_5'_0
mmu-mir-30c-2	14	mmu-miR-30c-2-3p	54-75	14-37	miRNA*_5'_0

		mmu-miR-30c-2-5p	14-37	54-75	miRNA*_5'_0
mmu-mir-7a-1	24	mmu-miR-7a-1-3p	66-87	24-47	miRNA*_5'_0
		mmu-miR-7a-1-5p	24-47	66-87	miRNA*_5'_0
mmu-mir-7a-2	19	mmu-miR-7a-2-3p	59-80	19-42	miRNA*_5'_0
		mmu-miR-7a-2-5p	19-42	59-80	miRNA*_5'_0
mmu-mir-92a-1	50	mmu-miR-92a-1-5p	11-33	50-71	miRNA*_5'_0
		mmu-miR-92a-1-3p	50-71	11-33	miRNA*_5'_0
mmu-mir-92a-2	55	mmu-miR-92a-2-5p	16-37	56-76	miRNA*_5'_-1
		mmu-miR-92a-2-3p	56-76	16-37	miRNA*_5'_-1
mmu-mir-1191a	101	mmu-miR-1191A	80-100	42-63	miRNA_3'_+1
mmu-mir-126a	46	mmu-miR-126A-5p	9-29	47-67	miRNA*_5'_-1
	46	mmu-miR-126A-3p	47-67	9_29	miRNA_5'_-1
mmu-mir-497a	14	mmu-miR-497A-5p	14-35	54-75	miRNA_5'_0
	14	mmu-miR-497A-3p	54-75	14_35	miRNA*_5'_0
mmu-mir-6516	46	mmu-miR-6516-5p	23-44	64-85	miRNA_3'_+2
	46	mmu-miR-6516-3p	64-85	23_44	miRNA*_3'_+2
mmu-mir-7079	26	mmu-miR-7079-5p	6-27	43-63	miRNA_3'_-1
	26	mmu-miR-7079-3p	43-63	6_27	miRNA*_3'_-1
mmu-mir-8094	68	mmu-miR-8094	26-46	65-85	miRNA*_5'_+3
mmu-mir-8099-1	98	mmu-miR-8099	36-56	74-94	
mmu-mir-8099-2	98	mmu-miR-8099	36-56	74-94	
mmu-mir-682	54	mmu-miR-682	74-94	11-35	

Supplementary table S1b. The loci of degradome signatures and known miRNAs/miRNAs* within the corresponding miRNA precursors in *Arabidopsis*

PremiRNA_Name	PARE_CutSites	miRNA_Name	miRNA_Position (Start-End)	miRNA*_Position(Start-End)	PARE_CutSites vs. miRNA/miRNA*_terminal_shifting
ath-MIR156a	21	ath-miR156a	21-40	83-104	miRNA_5'_0
ath-MIR156b	47/151	ath-miR156b	47-66	107-128	miRNA_5'_0
ath-MIR156c	12	ath-miR156c	12-31	75-96	miRNA_5'_0
ath-MIR156d	18/85	ath-miR156d	18-37	85-105	miRNA_5'_0/miRNA*_5'_0
ath-MIR156e	7	ath-miR156e	7-26	84-104	miRNA_5'_0
ath-MIR156f	21	ath-miR156f	21-40	92-112	miRNA_5'_0
ath-MIR156g	12	ath-miR156g	11-30	75-95	miRNA_5'_+1
ath-MIR156h	11	ath-miR156h	12-31	77-97	miRNA_5'_-1
ath-MIR156j	3	ath-miR156j	3-22	48-68	miRNA_5'_0
ath-MIR157a	4	ath-miR157a	4-24	75-96	miRNA_5'_0
ath-MIR157b	19	ath-miR157b	19-39	90-111	miRNA_5'_0
ath-MIR157c	24	ath-miR157c	24-44	170-190	miRNA_5'_0
ath-MIR157d	23	ath-miR157d	24-43	175-195	miRNA_5'_-1
ath-MIR158a	63/65	ath-miR158a	63-82	20-40	miRNA_5'_0
ath-MIR158b	55	ath-miR158b	56-75	13-34	miRNA_5'_-1
ath-MIR159a	163	ath-miR159a	163-183	4-24	miRNA_5'_0
ath-MIR159b	168	ath-miR159b	168-188	5-25	miRNA_5'_0
ath-MIR159c	195	ath-miR159c	195-215	13-32	miRNA_5'_0
ath-MIR160a	64	ath-miR160a	4-24	64-84	miRNA*_5'_0
ath-MIR160b	5/66	ath-miR160b	5-25	66-87	miRNA_5'_0/miRNA*_5'_0
ath-MIR161	46	ath-miR161.1	47-67	116-136	miRNA_5'_-1
ath-MIR162a	89	ath-miR162a	89-109	27-47	miRNA_5'_0
ath-MIR162b	79	ath-miR162b	79-99	14-34	miRNA_5'_0
ath-MIR163	294/38	ath-miR163	294-317	17-37	miRNA_5'_0/miRNA*_3'_0
ath-MIR164a	19	ath-miR164a	19-39	78-98	miRNA_5'_0
ath-MIR164b	3/114	ath-miR164b	3-23	137-153	miRNA_5'_0
ath-MIR164c	11	ath-miR164c	11-31	74-94	miRNA_5'_0
ath-MIR165a	86	ath-miR165a	86-106	8-28	miRNA_5'_0

ath-MIR165b	137/158	ath-miR165b	137-157	24-44	miRNA_5'_0/miRNA_3'_0
ath-MIR166a	134/133	ath-miR166a	134-154	21-41	miRNA_5'_0
ath-MIR166b	96	ath-miR166b	96-116	7-27	miRNA_5'_0
ath-MIR166c	103	ath-miR166c	103-123	18-38	miRNA_5'_0
ath-MIR166d	87	ath-miR166d	87-107	9-29	miRNA_5'_0
ath-MIR166e	119	ath-miR166e	119-139	7-27	miRNA_5'_0
ath-MIR166f	78	ath-miR166f	78-98	10-30	miRNA_5'_0
ath-MIR166g	85	ath-miR166g	85-105	18-38	miRNA_5'_0
ath-MIR167a	19	ath-miR167a	19-39	101-121	miRNA_5'_0
ath-MIR167b	10	ath-miR167b	10-30	80-102	miRNA_5'_0
ath-MIR167d	18	ath-miR167d	18-39	341-361	miRNA_5'_0
ath-MIR168a	18/103	ath-miR168a	18-38	103-123	miRNA_5'_0/miRNA*_5'_0
ath-MIR168b	19	ath-miR168b	19-39	89-109	miRNA_5'_0
ath-MIR169b	43	ath-miR169b	43-63	124-145	miRNA_5'_0
ath-MIR169c	113	ath-miR169c	113-133	308-328	miRNA_5'_0
ath-MIR170	18	ath-miR170	59-79	18-38	miRNA*_5'_0
ath-MIR171a	88	ath-miR171a	88-108	19-39	miRNA_5'_0
ath-MIR171b	78	ath-miR171b	78-98	15-35	miRNA_5'_0
ath-MIR171c	90	ath-miR171c	90-110	28-48	miRNA_5'_0
ath-MIR172a	78	ath-miR172a	78-98	5-25	miRNA_5'_0
ath-MIR172b	5/14	ath-miR172b-5p	5-24	72-91	miRNA_5'_0
		ath-miR172b-3p	71-91	5-25	miRNA*_5'_0
ath-MIR172c	98	ath-miR172c	98-118	18-38	miRNA_5'_0
ath-MIR172d	88	ath-miR172d	88-108	18-38	miRNA_5'_0
ath-MIR172e	13/95	ath-miR172e	95-115	13-33	miRNA_5'_0/miRNA*_5'_0
ath-MIR173	9	ath-miR173-5p	9-30	76-96	miRNA_5'_0
		ath-miR173-3p	75-95	10-31	miRNA*_5'_0
ath-MIR1886	4/13/84	ath-miR1886.1	4-24	93-113	miRNA_5'_0
		ath-miR1886.2	13-33	84-104	miRNA_5'_0
		ath-miR1886.3	84-104	13-33	miRNA_5'_0
ath-MIR1887	37/7	ath-miR1887	39-59	7-27	miRNA_5'_-2/miRNA*_5'_0
ath-MIR1888b	111	ath-miR1888b	26-46	93-113	miRNA*_3'_-3
ath-MIR2111b	45	ath-miR2111b-3	107-127	45-65	miRNA*_5'_0

		p			
		ath-miR2111b-5p	45-65	107-127	miRNA_5'_0
ath-MIR2112	123/8	ath-miR2112-3p	123-143	14-34	miRNA_5'_0
		ath-miR2112-5p	14-34	123-143	miRNA*_5'_0
ath-MIR319b	108/152	ath-miR319b	152-172	3-23	miRNA_5'_0
ath-MIR319c	169/126	ath-miR319c	170-190	12-32	miRNA_5'_-1
ath-MIR3434	273	ath-miR3434-3p	253-272	80-99	miRNA_3'_0
		ath-miR3434-5p	80-99	253-272	miRNA*_3'_0
ath-MIR3440b	86/84	ath-miR3440b-3p	84-104	22-42	miRNA_5'_0
		ath-miR3440b-5p	24-44	82-102	miRNA*_5'_0
ath-MIR390a	18/70	ath-miR390a	18-38	70-90	miRNA_5'_0/miRNA*_5'_0
ath-MIR390b	15	ath-miR390b	15-35	88-108	miRNA_5'_0
ath-MIR391	68	ath-miR391	68-88	119-139	miRNA_5'_0
ath-MIR3933	7/14	ath-miR3933	7-27	64-84	miRNA_5'_0
ath-MIR393a	105/11	ath-miR393a	11-32	105-125	miRNA_5'_0/miRNA*_5'_0
ath-MIR393b	132	ath-miR393b	11-32	132-152	miRNA*_5'_0
ath-MIR396a	11/123	ath-miR396a	11-31	123-143	miRNA_5'_0/miRNA*_5'_0
ath-MIR396b	11/107	ath-miR396b	11-31	107-127	miRNA_5'_0/miRNA*_5'_0
ath-MIR397a	87/11	ath-miR397a	11-31	79-99	miRNA_5'_0
ath-MIR398a	75/13	ath-miR398a	75-95	13-33	miRNA_5'_0/miRNA*_5'_0
ath-MIR398b	86/13	ath-miR398b	86-106	13-33	miRNA_5'_0/miRNA*_5'_0
ath-MIR398c	85/12/91/18	ath-miR398c	85-105	13-33	miRNA_5'_0/miRNA*_5'_-1
ath-MIR399a	13	ath-miR399a	93-113	13-34	miRNA*_5'_0
ath-MIR399b	105	ath-miR399b	105-125	13-33	miRNA_5'_0
ath-MIR399c	84	ath-miR399c	84-104	13-33	miRNA_5'_0
ath-MIR400	79/73/12	ath-miR400	12-32	71-91	miRNA_5'_0/miRNA*_5'_+2
ath-MIR403	103	ath-miR403	103-123	26-47	miRNA_5'_0
ath-MIR408	141/143/120/121/142/53	ath-miR408	120-140	54-74	miRNA_5'_0/miRNA_3'_0/miRNA*_5'_-1
ath-MIR414	40/43	ath-miR414	25-45	68-90	miRNA_3'_-2

ath-MIR4240	155	ath-miR4240	31-51	155-175	miRNA*_5'_0
ath-MIR4243	20	ath-miR4243	20-40	119-139	miRNA_5'_0
ath-MIR4245	104	ath-miR4245	104-124	5-25	miRNA_5'_0
ath-MIR5014a	103	ath-miR5014a-5p	22-42	102-122	miRNA*_5'_0
		ath-miR5014a-3p	103-123	21-41	miRNA_5'_0
ath-MIR5021	44	ath-miR5021	41-60	120-140	miRNA_5'_+3
ath-MIR5025	22	ath-miR5025	23-43	96-116	miRNA_5'_-1
ath-MIR5634	203	ath-miR5634	3-23	206-226	miRNA*_5'_-3
ath-MIR5642a	343/248/249/280	ath-miR5642a	343-362	15-35	miRNA_5'_0
ath-MIR5642b	341/246/278	ath-miR5642b	341-360	15-35	miRNA_5'_0
ath-MIR5643a	61	ath-miR5643a	61-81	5-27	miRNA_5'_0
ath-MIR5643b	61	ath-miR5643b	61-81	5-27	miRNA_5'_0
ath-MIR5645c	356	ath-miR5645c	356-376	11-31	miRNA_5'_0
ath-MIR5646	51	ath-miR5646	51-70	3-24	miRNA_5'_0
ath-MIR5653	62	ath-miR5653	63-86	3-23	miRNA_5'_-1
ath-MIR5656	86	ath-miR5656	89-109	47-67	miRNA_5'_-3
ath-MIR5657	59	ath-miR5657	61-81	7-28	miRNA_5'_-2
ath-MIR5658	2	ath-miR5658	3-23	74-94	miRNA_5'_-1
ath-MIR5661	3	ath-miR5661	3-23	61-81	miRNA_5'_0
ath-MIR5997	104	ath-miR5997	104-124	43-63	miRNA_5'_0
ath-MIR771	12	ath-miR771	12-33	82-102	miRNA_5'_0
ath-MIR773a	63/4	ath-miR773a	63-83	5-24	miRNA_5'_0/miRNA*_5'_-1
ath-MIR774a	11	ath-miR774a	70-90	11-31	miRNA*_5'_0
ath-MIR775	93/98	ath-miR775	95-114	11-30	miRNA_5'_-2
ath-MIR776	81/82/19	ath-miR776	82-102	20-39	miRNA_5'_0/miRNA*_5'_-1
ath-MIR777	84	ath-miR777	84-105	6-26	miRNA_5'_0
ath-MIR779	106	ath-miR779.2	106-126	44-65	miRNA_5'_0
ath-MIR780	147	ath-miR780.1	129-149	29-49	miRNA_3'_-3
		ath-miR780.2	150-170	7-28	miRNA_5'_-3
ath-MIR781a	6/70	ath-miR781a	6-26	70-91	miRNA_5'_0/miRNA*_5'_0
ath-MIR781b	3	ath-miR781b	3-23	67-87	miRNA_5'_0

ath-MIR824	649	ath-miR824	35-55	628-648	miRNA*_3'_0
ath-MIR825	115/130	ath-miR825	115-135	54-75	miRNA_5'_0
ath-MIR829	110	ath-miR829.1	110-133	75-98	miRNA_5'_0
ath-MIR839	174/50/218	ath-miR839	50-70	196-217	miRNA_5'_0/miRNA*_3'_0
ath-MIR840	206/201/179/93/180	ath-miR840	50-71	158-179	miRNA*_3'_0
ath-MIR847	54	ath-miR847	159-179	54-74	miRNA*_5'_0
ath-MIR851	138	ath-miR851-5p	50-70	117-137	miRNA*_3'_0
		ath-miR851-3p	119-139	48-68	miRNA_3'_-2
ath-MIR853	223/244	ath-miR853	50-71	223-243	miRNA*_5'_0/miRNA*_3'_0
ath-MIR858a	12	ath-miR858a	11-31	154-174	miRNA_5'_+1
ath-MIR858b	275	ath-miR858b	275-295	17-35	miRNA_5'_0
ath-MIR859	18/91	ath-miR859	18-38	91-111	miRNA_5'_0/miRNA*_5'_0
ath-MIR860	86/107/89	ath-miR860	86-106	23-43	miRNA_5'_0/miRNA_3'_0
ath-MIR864	4	ath-miR864-5p	4-24	70-91	miRNA_5'_0
		ath-miR864-3p	70-91	4-24	miRNA*_5'_0
ath-MIR867	24	ath-miR867	24-44	76-96	miRNA_5'_0
ath-MIR868	55	ath-miR868-5p	54-74	118-138	miRNA_5'_+1
		ath-miR868-3p	166-186	9-29	
ath-MIR8121	11/33	ath-miR8121	11-34	110-133	miRNA_5'_0/miRNA_3'_0
ath-MIR8166	24	ath-miR8166	24-45	50-71	miRNA_5'_0
ath-MIR8167a	27	ath-miR8167a	54-75	7-26	miRNA*_3'_0
ath-MIR8167b	27	ath-miR8167b	54-75	7-26	miRNA*_3'_0
ath-MIR8167c	27	ath-miR8167c	54-75	7-26	miRNA*_3'_0
ath-MIR8167d	27	ath-miR8167d	54-75	7-26	miRNA*_3'_0
ath-MIR8167e	27	ath-miR8167e	54-75	7-26	miRNA*_3'_0
ath-MIR8167f	27	ath-miR8167f	54-75	7-26	miRNA*_3'_0
ath-MIR8168	54	ath-miR8168	57-77	7-26	miRNA_5'_-3
ath-MIR8169	106	ath-miR8169	104-124	9-32	miRNA_5'_+2
ath-MIR8173	82	ath-miR8173	82-102	6-25	miRNA_5'_0
ath-MIR8178	137	ath-miR8178	81-101	140-160	miRNA*_3'_-3
ath-MIR161		ath-miR161.2	38-58	125-145	
ath-MIR169a	87	ath-miR169a	18-38	190-210	
ath-MIR2936	45	ath-miR2936	15-36	138-160	

ath-MIR2939	102	ath-miR2939	79-99	29-47	
ath-MIR319a	110	ath-miR319a	154-174	5-25	
ath-MIR395a	67	ath-miR395a	63-83	13-33	
ath-MIR395d	74	ath-miR395d	70-90	13-33	
ath-MIR395e	69	ath-miR395e	65-85	13-33	
ath-MIR402	287	ath-miR402	67-88	245-265	
ath-MIR405a	27	ath-miR405a	90-113	61-83	
ath-MIR405b	27	ath-miR405b	70-93	40-63	
ath-MIR405d	7/15	ath-miR405d	49-72	20-42	
ath-MIR5014b	2	ath-miR5014b	21-41	115-135	
ath-MIR5017	107	ath-miR5017	99-119	57-77	
ath-MIR5020a	45	ath-miR5020a	50-70	106-126	
ath-MIR5023	101	ath-miR5023	30-50	86-106	
ath-MIR5629	18	ath-miR5629	220-241	111-132	
ath-MIR5632	80	ath-miR5632	132-152	11-32	
ath-MIR5635a	349/348	ath-miR5635a	268-288	157-177	
ath-MIR5635c	138	ath-miR5635c	134-154	23-43	
ath-MIR5644	7	ath-miR5644	3-22	129-145	
ath-MIR5645d	300	ath-miR5645d	37-56	417-436	
ath-MIR5655	17/18	ath-miR5655	83-103	395-415	
ath-MIR5662	256	ath-miR5662	268-287	3-22	
ath-MIR5995b	117	ath-miR5995b	70-90	103-123	
ath-MIR779		ath-miR779.1	142-162	4-23	
ath-MIR822	13	ath-miR822	50-70	270-290	
ath-MIR823	168	ath-miR823	120-140	54-74	
ath-MIR828	138	ath-miR828	50-71	123-144	
ath-MIR829		ath-miR829.2	134-154	54-74	
ath-MIR833a	157/42	ath-miR833a-3p	115-136	54-75	
		ath-miR833a-5p	54-75	115-136	
ath-MIR835	280/303	ath-miR835-3p	363-383	50-70	
		ath-miR835-5p	50-70	363-383	
ath-MIR838	142	ath-miR838	136-156	54-74	
ath-MIR848	68	ath-miR848	123-144	55-75	

ath-MIR856	92	ath-miR856	28-49	230-250	
ath-MIR857	45	ath-miR857	344-364	16-36	
ath-MIR863	233	ath-miR863-5p	48-68	240-260	
		ath-miR863-3p	239-259	49-69	
ath-MIR865	4	ath-miR865-3p	108-128	17-37	
		ath-miR865-5p	9-29	116-136	
ath-MIR8170	126	ath-miR8170-5p	62-82	121-141	
ath-MIR8170	126	ath-miR8170-3p	121-141	62-82	
ath-MIR8175	135	ath-miR8175	142-161	31-50	
ath-MIR8176	59/63/67	ath-miR8176	23-43	50-70	
ath-MIR8180	45/129	ath-miR8180	133-151	67-83	
ath-MIR8181	108	ath-miR8181	1-20	114-134	
ath-MIR8183	18/43/44	ath-miR8183	13-33	87-107	
ath-MIR826a	124	ath-miR826a	50-70	111-129	
ath-MIR169g	250	ath-miR169g-3p	204-224	148-168	
		ath-miR169g-5p	144-164	208-228	

Supplementary table S2a. Discovery of known miRNAs from flowers of *Arabidopsis.*

1. Degradome supported cutsite(s) in Genome:28656

PremiRNA Locus in Genome:>Chr1(28545-28660)_plus strand

PremiRNA sequence:UGGUGUUGUGCAAGAAGGAGAAGCAAAGUCUGUCUAUGUAUUAUGAGAUAGCUACUUCUAUGGCUAGGAUAUAUGUUGUACAAGACCGGCUUUUCUUCUACUUCUUGCACAACCUG

PremiRNA secondary structure:(((.((((((((((((.(((((.((((((.((((.(((((((((...((((((......))))))...))))....))))))))).)))))).))))).)))))))))))))))

Degradome supported cutsite(s) in premiRNA:112

miRNA sequence and Count(AGO1/wt):UUUUCUUCUACUUCUUGCACA_4.76/3.83

miRNA Position in PremiRNA:91-111

miRNA* sequence and Count(AGO1/wt):UGCAAGAAGGAGAAGCAAAGU_0.25/0.81

miRNA Position in PremiRNA:9-29

miRNA annonation:ath-miR838

2. Degradome supported cutsite(s) in Genome:2165570

PremiRNA Locus in Genome:>Chr1(2165563-2165700)_plus strand

PremiRNA sequence:UCUUACAUCUUGAUGAAGAGGAAUGGGAAGCCGAAAUCUAAACUGAAACAGAGGCGUCACCCAAUUAUCAAUUGGUAAGUGAUAUCUAUGUUUCGAUCUGUAUCUCGGCCUUUCACUCCUCUUCUUCUUGAUGUAAGA

PremiRNA secondary structure:(((((((((..((.((((((((.((..(.(((((.........(((((((.((..((((((((((.....)))))...)))))..)).))))))).........))))).)..)).)))))))).))..)))))))))

Degradome supported cutsite(s) in premiRNA:8

miRNA sequence and Count(AGO1/wt):UCACUCCUCUUCUUCUUGAUG_12.77/5.24

miRNA Position in PremiRNA:113-133

miRNA* sequence and Count(AGO1/wt):UCUUGAUGAAGAGGAAUGGGA_1.25/2.01

miRNA Position in PremiRNA:8-28

miRNA annonation:ath-miR847

3. Degradome supported cutsite(s) in Genome:6220813/6220834

PremiRNA Locus in Genome:>Chr1(6220645-6220838)_plus strand

PremiRNA sequence:AGGAAGAGCUCCUUGAAGUUCAAUGGAGGGUUUAGCAGGGUGAAGUAAAGCUGCUAAGCUAUGGAUCCCAUAAGCCUUAUCAAAUUCAAUAUAAUUGAUGAUAAGGUUUUUUUUAUGGAUGCCAUAUCUCAGGAGCUUUCACUUACCCCUUUAAUGGCUUCACUCUUCUUUGGAUUGAAGGGAGCUCUUCAUCU

PremiRNA secondary
structure:(((((((((((((..(((((((.(((((((..(((((((..((((((((((.((.((.(((((...((((((((((((((...(((((...))))))))))))))).....))))))...))))).)).)).)))))).))))..))))......)))..))))))).)))))))..))))))))))))..))

Degradome supported cutsite(s) in premiRNA:169/190
miRNA sequence and Count(AGO1/wt):UUUGGAUUGAAGGGAGCUCUU_27199.01/2653.42
miRNA Position in PremiRNA:169-189
miRNA* sequence and Count(AGO1/wt):GAGCUCCUUGAAGUUCAAUGG_5.76/107.55
miRNA Position in PremiRNA:6-26
miRNA annonation:ath-miR159b

4. Degradome supported cutsite(s) in Genome:6740590
PremiRNA Locus in Genome:>Chr1(6740498-6740593)_plus strand
PremiRNA
sequence:UUGCUUCAGGUAUGAUUGACUUCAAAAAAUACCUUGAAACUAUAAACCUCAGUUUCUUUGAAUUUGAUUUUUAAAGUCAAUAAUACCUUGA
AGCAA
PremiRNA secondary structure:((((((((((((.((((((((.(((((.......((((((.........))))))...........))))).)))))))).))))).))))))))
Degradome supported cutsite(s) in premiRNA:93
miRNA sequence and Count(AGO1/wt):UCAGGUAUGAUUGACUUCAAA_40.56/12.08
miRNA Position in PremiRNA:6-26
miRNA* sequence and Count(AGO1/wt):UAAAGUCAAUAAUACCUUGAAG_0.75/0.81
miRNA Position in PremiRNA:72-93
miRNA annonation:ath-miR864-5p

5. Degradome supported cutsite(s) in Genome:9364561
PremiRNA Locus in Genome:>Chr1(9364477-9364564)_plus strand
PremiRNA
sequence:CCAUGAGUUCCCUUUAACGCUUCAUUGUUAAAUACUCAAAGCCACAUUGGUUUGUAUACAACACUGAAGUGUUUGGGGGACUCUUGG

PremiRNA secondary structure:(((.(((((((((..(((((((((.((((..((((...(((((.....)))))))))..)))).)))))))))..))))))))).)))
Degradome supported cutsite(s) in premiRNA:85
miRNA sequence and Count(AGO1/wt):CUGAAGUGUUUGGGGGACUC_65.84/4.63
miRNA Position in PremiRNA:64-84
miRNA* sequence and Count(AGO1/wt):GUUCCCUUUAACGCUUCAUUG_0/0.4
miRNA Position in PremiRNA:7-27

miRNA annonation:ath-miR395f/ath-miR395c/ath-miR395b

6. Degradome supported cutsite(s) in Genome:17825722
PremiRNA Locus in Genome:>Chr1(17825720-17825829)_plus strand
PremiRNA sequence:GAUCAAUGCAUUGAAAGUGACUACAUCGGGGUUCCGAUUUUUUUUGUUCUUCAUAUGAUGAAGCGGAAACAGUAAUCAACCCUGGUUUAGUCACUUUCACUGCAUUAAUC
PremiRNA secondary structure:(((.((((((.((((((((((((.(((((((((..((((....(((((((((((...))))))....))))).))))))))))))).))))))))))))).)))))).)))
Degradome supported cutsite(s) in premiRNA:3
miRNA sequence and Count(AGO1/wt):UCAAUGCAUUGAAAGUGACUA_4549.61/904.55
miRNA Position in PremiRNA:3-23
miRNA* sequence and Count(AGO1/wt):GUCACUUUCACUGCAUUAAUC_0.75/2.82
miRNA Position in PremiRNA:90-110
miRNA annonation:ath-miR161.2

7. Degradome supported cutsite(s) in Genome:20041586
PremiRNA Locus in Genome:>Chr1(20041581-20041665)_plus strand
PremiRNA sequence:GAGAUUGAGCCAAGGAUGACUUGCCGAUUUUCUCAACGAAUCUUACUGAUUAUGGUAUCCGGCAAGUUGACUUUGGCUCUGUUUC

PremiRNA secondary structure:(((((.(((((((((.((((((((((...(((.....)))...((((......))))..)))))))))).))))))))).)))))
Degradome supported cutsite(s) in premiRNA:6
miRNA sequence and Count(AGO1/wt):UGAGCCAAGGAUGACUUGCCG_31.79/13.49
miRNA Position in PremiRNA:6-26
miRNA* sequence and Count(AGO1/wt):GCAAGUUGACUUUGGCUCUGU_0/2.22
miRNA Position in PremiRNA:62-82
miRNA annonation:ath-miR169g-5p/ath-miR169d/ath-miR169e/ath-miR169f

8. Degradome supported cutsite(s) in Genome:22149808/22149735
PremiRNA Locus in Genome:>Chr1(22149730-22149831)_plus strand
PremiRNA sequence:CGAAAUCUCUCUGUUGUGAAGUCAAACAUGAGUAUGAAUUAACAUUAAUGGAUCUUUUUGAUUCAUAAUACUCAUGUUUGGUUUCACAGUAGAUAGAUAUCG
PremiRNA secondary structure:(((.((((.((((((((((..(((((((((((((............(((((((.....))))))).)))))))))))))..)))))))))).)))).)))

Degradome supported cutsite(s) in premiRNA:79/6
miRNA sequence and Count(AGO1/wt):UCUCUCUGUUGUGAAGUCAAA_10.01/15.31
miRNA Position in PremiRNA:6-26
miRNA* sequence and Count(AGO1/wt):UGGUUUCACAGUAGAUAGAUA_0.75/1.61
miRNA Position in PremiRNA:79-99
miRNA annonation:ath-miR859

9. Degradome supported cutsite(s) in Genome:24554011
PremiRNA Locus in Genome:>Chr1(24553982-24554031)_plus strand
PremiRNA sequence:CGUACACUUACAUCUGUACACUAAAAGUGUUGUACAAAUUUAAGUGUACG
PremiRNA secondary structure:((((((((((.((.(((((((.......).)))))).))).))))))))))
Degradome supported cutsite(s) in premiRNA:30
miRNA sequence and Count(AGO1/wt):UUGUACAAAUUUAAGUGUACG_8.26/2.01
miRNA Position in PremiRNA:30-50
miRNA* sequence and Count(AGO1/wt):UACACUUACAUCUGUACACUA_0/0
miRNA Position in PremiRNA:3-23
miRNA annonation:ath-miR5014a-3p

10. Degradome supported cutsite(s) in Genome:24884359
PremiRNA Locus in Genome:>Chr1(24884354-24884581)_plus strand
PremiRNA
sequence:AGUCGUUGAAGAGGACUUGGAACUUCGAUAUUAUCCCCCGUGUUUUGUCCAGUUUCUCUAUAGGUUGUGCCUGAAAAUAUGGGUUUCUAGG
UUUAAUUGAUGAAAAGAGAUAACGUAAGUCAUGCACAUGUAAUUAAAUUCAUGCCCCUAACUAUUAAUUCUUGCAAGUAAUUAAAAAAUAAAACGU
ACACAAAAUAUAUUUAUGAUCACUAAUCCUUUUUAACUACU
PremiRNA secondary
structure:(((.(((((((((((.((((......(((...)))....(((((((((...((((.....((((.(((((.(((...((((.((((((...((((.....)))).))))))..))))..))).))))).(((..........))).))))....((((((((....)).))))))......))))....)))))))))..........))))))))
))))))).)))
Degradome supported cutsite(s) in premiRNA:6
miRNA sequence and Count(AGO1/wt):UUGAAGAGGACUUGGAACUUCGAU_49547.99/3690.3
miRNA Position in PremiRNA:6-29
miRNA* sequence and Count(AGO1/wt):UAUCCCCCGUGUUUUGUCC_1.25/1.01
miRNA Position in PremiRNA:35-225
miRNA annonation:ath-miR163

11. Degradome supported cutsite(s) in Genome:24921104
PremiRNA Locus in Genome:>Chr1(24921099-24921200)_plus strand
PremiRNA sequence:UAGUGUUGACAGAAGAUAGAGAGCACAGAUGAUAAGAUACAAUUCCUCGCAGCUUCUUUGCAUCUUACUCCUUUGUGCUCUCUAGCCUUCUGUCAUCACCCG
PremiRNA secondary structure:(.(((.(((((((((.(((((((((((((.((((((((.(((..............))).)))))).)).)))))))))))))..))))))))).)))..)
Degradome supported cutsite(s) in premiRNA:6
miRNA sequence and Count(AGO1/wt):UUGACAGAAGAUAGAGAGCAC_167.23/89.02
miRNA Position in PremiRNA:6-26
miRNA* sequence and Count(AGO1/wt):GCUCUCUAGCCUUCUGUCAUCA_0/0.4
miRNA Position in PremiRNA:77-98
miRNA annonation:ath-miR157b/ath-miR157a/ath-miR157c

12. Degradome supported cutsite(s) in Genome:26638083
PremiRNA Locus in Genome:>Chr1(26638000-26638107)_plus strand
PremiRNA sequence:AGAGAGCAACAAAAUCAGUGUGUAUUUGUUAAGACAAAUGUUUAUAUAAGUCGUCCAUGCGUUGUACAUUUAAUCUUAACUAAUACGCAUUGAGUUUCGUUGCUUUCU
PremiRNA secondary structure:((((((((((((.((((((((((((.(((((((.((((((....((((..(((....))))))))))))))..))))))).)))))))))))).))).))))))))))
Degradome supported cutsite(s) in premiRNA:84
miRNA sequence and Count(AGO1/wt):UACGCAUUGAGUUUCGUUGCUU_36.05/22.16
miRNA Position in PremiRNA:84-105
miRNA* sequence and Count(AGO1/wt):CAACAAAAUCAGUGUGUAUU_0/0.4
miRNA Position in PremiRNA:6-26
miRNA annonation:ath-miR777

13. Degradome supported cutsite(s) in Genome:29422546
PremiRNA Locus in Genome:>Chr1(29422456-29422570)_plus strand
PremiRNA sequence:AACGUUGCACUACGUGACAUUGAAACUGUCUUUCAACAUUCCAAUAUUUCAACUUUCGAAUACCCAAUAUUUGGUUUGUUCAAAGACAUUUUCGAUGUCUAGCAGUGCCAAUGUU
PremiRNA secondary structure:((((((((((....((((((((((.(((((((.((((..((((((((.................)))).))))..)))).))))))).))))))))))....)))).)))))))
Degradome supported cutsite(s) in premiRNA:91
miRNA sequence and Count(AGO1/wt):UUCGAUGUCUAGCAGUGCCA_236.83/127.9

miRNA Position in PremiRNA:91-110
miRNA* sequence and Count(AGO1/wt):GCACUACGUGACAUUGAAAC_0.5/29.2
miRNA Position in PremiRNA:7-26
miRNA annonation:ath-miR775

14. Degradome supported cutsite(s) in Genome:22150026
PremiRNA Locus in Genome:>Chr1(22149939-22150030)_plus strand
PremiRNA
sequence:UUUCAGAUGGCUGUUUGGGUAACUAAUAUUUAAGAUUUUGGUCAAUUUAAGUUACCAAUUUAUAUAUUGGUUACCCAUAUGGCCAUCUCAA
A
PremiRNA secondary structure:(((.((((((((((.((((((((((((((.((((...(((((.(((....)))))))))))).))))))))))))))).)))))))))).)))
Degradome supported cutsite(s) in premiRNA:88
miRNA sequence and Count(AGO1/wt):UUGGUUACCCAUAUGGCCAUC_8.76/1.61
miRNA Position in PremiRNA:67-87
miRNA* sequence and Count(AGO1/wt):UGGCUGUUUGGGUAACUAAUA_0.75/0.4
miRNA Position in PremiRNA:8-28
miRNA annonation:ath-miR774a

15. Degradome supported cutsite(s) in Genome:1041012
PremiRNA Locus in Genome:>Chr2(1040937-1041037)_plus strand
PremiRNA
sequence:GUGAAAUUUCAAAGGAGUGGCAUGUGAACACAUAUCCUAUGGUUUCUUCAAAUUUCCAUUGAAACCAUUGAGUUUUGUGUUCUCAGGUCAC
CCCUUUGAAU
PremiRNA secondary structure:((......(((((((.(((((.((.(((((((..((..((((((((..............)))))))).))....))))))).)).))))).)))))))))
Degradome supported cutsite(s) in premiRNA:76
miRNA sequence and Count(AGO1/wt):UGUGUUCUCAGGUCACCCCUU_3.5/0.6
miRNA Position in PremiRNA:76-96
miRNA* sequence and Count(AGO1/wt):GGAGUGGCAUGUGAACACAUA_0/0.6
miRNA Position in PremiRNA:14-34
miRNA annonation:ath-miR398a

16. Degradome supported cutsite(s) in Genome:9560866/9560887
PremiRNA Locus in Genome:>Chr2(9560769-9560891)_plus strand

PremiRNA
sequence:UAAUAUAGUUGAAUCUUCGAAAGUAUUUCAAGAAGUCAGCUGAGCUUUCUCGUCAUCACUUAUUAGUAAAUAUAGUCUCUAUAUUUUGAU
GAGUGAUGAUUGGAAAUUUCGUUGACUCAUUA
PremiRNA secondary structure:((((....(((((....(....)...)))))..(((((((.(((.(((((.(((((((((((((((.((((((((...))))))))))))))))))))))).))))).))))))))))).))))
Degradome supported cutsite(s) in premiRNA:98/119
miRNA sequence and Count(AGO1/wt):UGAUUGGAAAUUUCGUUGACU_46.31/8.26
miRNA Position in PremiRNA:98-118
miRNA* sequence and Count(AGO1/wt):UCAGCUGAGCUUUCUCGUCAUC_58.08/10.67
miRNA Position in PremiRNA:36-57
miRNA annonation:ath-miR779.2

17. Degradome supported cutsite(s) in Genome:11159774
PremiRNA Locus in Genome:>Chr2(11159706-111159799)_plus strand
PremiRNA
sequence:GACUCGUUCAAGCACCAGCUCGAAGAAGCUUAGCUAAUUUAUCUUAGAAAAUAAUGAAAAAGCUAUGCUUCUCAAGAAGGUGCAUGAACAA
GUU
PremiRNA secondary structure:((((.(((((.(((((..((.((.(((((.(((((..(((((..(.....)..)))))..))))).)))))))).))..))))).))))).))))
Degradome supported cutsite(s) in premiRNA:69
miRNA sequence and Count(AGO1/wt):UUCUCAAGAAGGUGCAUGAAC_54.58/17.12
miRNA Position in PremiRNA:69-89
miRNA* sequence and Count(AGO1/wt):UCAAGCACCAGCUCGAAGAAGC_3.5/2.42
miRNA Position in PremiRNA:8-29
miRNA annonation:ath-miR825

18. Degradome supported cutsite(s) in Genome:15611882
PremiRNA Locus in Genome:>Chr2(15611875-15611978)_plus strand
PremiRNA
sequence:AGAGAAGUGAGAUGAAAUCUUUGAUUGGAAAUUUCAUGUAGACGAUGCUAUCACCCCAUAUGACCAAAAGAAAUUCCCAAUUAAAGAUUUC
AUCUUACUUCCCU
PremiRNA secondary structure:((.(((((((((((((((((((((((((.((((((..(((.....)))..(((.......)))......)))))).))))))))))))))))))))))))).))
Degradome supported cutsite(s) in premiRNA:8
miRNA sequence and Count(AGO1/wt):AAUUAAAGAUUUCAUCUUACU_0/3.22
miRNA Position in PremiRNA:79-99
miRNA* sequence and Count(AGO1/wt):UGAGAUGAAAUCUUUGAUUGG_70.35/20.34

miRNA Position in PremiRNA:8-28
miRNA annonation:ath-miR1886.3

19. Degradome supported cutsite(s) in Genome:16061971
PremiRNA Locus in Genome:>Chr2(16061966-16062046)_plus strand
PremiRNA sequence:CUGUAAAGCUCAGGAGGGAUAGCGCCAUGAUGAUCACAUUCGUUAUCUAUUUUUGGCGCUAUCCAUCCUGAGUUUCAUUG

PremiRNA secondary structure:(.((.((((((((((.(((((((((((.((((((.((....)).))).)))...))))))))))).))))))))))).)).)
Degradome supported cutsite(s) in premiRNA:6
miRNA sequence and Count(AGO1/wt):AAGCUCAGGAGGGAUAGCGCC_181.75/459.42
miRNA Position in PremiRNA:6-26
miRNA* sequence and Count(AGO1/wt):CGCUAUCCAUCCUGAGUUUCA_35.3/216.92
miRNA Position in PremiRNA:58-78
miRNA annonation:ath-miR390b/ath-miR390a

20. Degradome supported cutsite(s) in Genome:16340282/16340363
PremiRNA Locus in Genome:>Chr2(16340279-16340363)_plus strand
PremiRNA sequence:GUAUGCCUGGCUCCCUGUAUGCCAUAUGCUGAGCCCAUCGAGUAUCGAUGACCUCCGUGGAUGGCGUAUGAGGAGCCAUGCAUAU

PremiRNA secondary structure:((((((.(((((((..(((((((((.(((.(((..(((((.....)))))..))).))).)))))))))..))))))).))))))
Degradome supported cutsite(s) in premiRNA:4/85
miRNA sequence and Count(AGO1/wt):UGCCUGGCUCCCUGUAUGCCA_2413.62/120.24
miRNA Position in PremiRNA:4-24
miRNA* sequence and Count(AGO1/wt):GCGUAUGAGGAGCCAUGCAUA_0/16.31
miRNA Position in PremiRNA:64-84
miRNA annonation:ath-miR160a/ath-miR160c/ath-miR160b

21. Degradome supported cutsite(s) in Genome:19176240
PremiRNA Locus in Genome:>Chr2(19176122-19176265)_plus strand
PremiRNA
sequence:UUGAGGGGACUGUUGUCUGGCUCGAGGACUCUGGCUCGCUCUAUUCAUGUUGGAUCUCUUUCGAUCUAACAAUCGAAUUGAACCUUCAGAU
UUCAGAUUUGAUUAGGGUUUUAGCGUCUUCGGACCAGGCUUCAUUCCCCCCAA
PremiRNA secondary structure:(((.(((((.((..((((((..(((((((.((((...((((((.((((((((((((......)))))))))...(((((((....))))..))).....))).)))))).)))).)))))))..))))))..)).))))).)))
Degradome supported cutsite(s) in premiRNA:119

miRNA sequence and Count(AGO1/wt):UCGGACCAGGCUUCAUUCCCC_76756.02/3261.89
miRNA Position in PremiRNA:120-140
miRNA* sequence and Count(AGO1/wt):GGACUGUUGUCUGGCUCGAGG_0.25/87.82
miRNA Position in PremiRNA:7-27
miRNA annonation:ath-miR166b/ath-miR166f/ath-miR166a/ath-miR166g/ath-miR166c/ath-miR166d/ath-miR166e

22. Degradome supported cutsite(s) in Genome:19520770
PremiRNA Locus in Genome:>Chr2(19520766-19520851)_plus strand
PremiRNA
sequence:AUGUUGGAGAAGCAGGGCACGUGCAAACCAACAAACACGAAAUCCGUCUCAUUUGCUUAUUUGCACGUACUUAACUUCUCCAACAU

PremiRNA secondary structure:((((((((((((..(((.(((((((((....((((.(((.....)))....))))....))))))))).)))..))))))))))))
Degradome supported cutsite(s) in premiRNA:5
miRNA sequence and Count(AGO1/wt):UGGAGAAGCAGGGCACGUGCA_984.13/151.06
miRNA Position in PremiRNA:5-25
miRNA* sequence and Count(AGO1/wt):CACGUACUUAACUUCUCCAAC_11.77/15.91
miRNA Position in PremiRNA:64-84
miRNA annonation:ath-miR164b/ath-miR164a

23. Degradome supported cutsite(s) in Genome:6217561
PremiRNA Locus in Genome:>Chr3(6217490-6217584)_plus strand
PremiRNA
sequence:CAAGUAUGGUUUUCUUGGCCCAUCCACUUCUAAUGUGUUUUGUAACUAAUGCAAAAUGUGCAUUAGAAGUGGAUUGGUCAAGGGAAGCGUA
CUUG
PremiRNA secondary structure:((((((((.((..(((((((.(((((((((((((((((((((((.....))))))))..))))))))))))))).)))))))..)).))))))))
Degradome supported cutsite(s) in premiRNA:72
miRNA sequence and Count(AGO1/wt):UGGAUUGGUCAAGGGAAGCGU_2/0.6
miRNA Position in PremiRNA:70-90
miRNA* sequence and Count(AGO1/wt):UUUUCUUGGCCCAUCCACUUC_2.25/0.6
miRNA Position in PremiRNA:8-28
miRNA annonation:ath-miR3440b-3p

24. Degradome supported cutsite(s) in Genome:8108090/8108172/8108193
PremiRNA Locus in Genome:>Chr3(8108090-8108190)_plus strand

PremiRNA sequence:UGAAGCUGCCAGCAUGAUCUAAUUAGCUUUCUUUAUCCUUUGUUGUGUUUCAUGACGAUGGUUAAGAGAUCAGUCUCGAUUAGAUCAUGUUCGCAGUUUCA
PremiRNA secondary structure:(((((((((.((((((((((((((((((.(((((..((.((((..((...))..)))).))...)))))..)).)).))))))))))))))).)))))))))
Degradome supported cutsite(s) in premiRNA:1/83/104
miRNA sequence and Count(AGO1/wt):UGAAGCUGCCAGCAUGAUCUA_6554.16/1991.78
miRNA Position in PremiRNA:1-21
miRNA* sequence and Count(AGO1/wt):GAUCAUGUUCGCAGUUUCACC_0.25/0.2
miRNA Position in PremiRNA:83-103
miRNA annonation:ath-miR167b/ath-miR167a

25. Degradome supported cutsite(s) in Genome:8236161
PremiRNA Locus in Genome:>Chr3(8236156-8236251)_plus strand
PremiRNA sequence:GUACUUUCGCUUGCAGAGAGAAAUCACAGUGGUCAAAAAAGUUGUAGUUUUCUUAAAGUCUCUUUCCUCUGUGAUUCUCUGUGUAAGCGAAAGAGC
PremiRNA secondary structure:((.((((((((((((.((((((.((((((.((.........(((.((....)))))).........)).))))))))))))).))))))))))))).))
Degradome supported cutsite(s) in premiRNA:6
miRNA sequence and Count(AGO1/wt):UUCGCUUGCAGAGAGAAAUCAC_3485.12/859.63
miRNA Position in PremiRNA:6-27
miRNA* sequence and Count(AGO1/wt):GAUUCUCUGUGUAAGCGAAAG_1/24.98
miRNA Position in PremiRNA:73-93
miRNA annonation:ath-miR173-5p

26. Degradome supported cutsite(s) in Genome:8346589/8346610
PremiRNA Locus in Genome:>Chr3(8346411-8346612)_plus strand
PremiRNA sequence:GCUGCUCCCCUCUUUAGCUUGGAGAAGCCAGUAAAUAUCUUUCUCUUCAAGGAACUUGUGGGCCUCAAGAAAACCUGCUAUAAUUCUAGUUUUUCAACAUUCCUCCUUCUGAUAUCAGCAACAACUCCUUACCCUUAAGAAUGGAGAAUGGAACUUCCUCAAAGAUAUUUACUUGGCCUCUGCAACCGGAAAGGGGAGCAGC
PremiRNA secondary structure:(((((((((((.((..(.(((.(((.((((((((((((((((.......(((..(....)..)))...(((((...((((......)))))))))..(((((.(((..(((....))).........((((....))))...))))))))..........))))))))))).)))).))).)))).)).)))))))))))

Degradome supported cutsite(s) in premiRNA:179/200

miRNA sequence and Count(AGO1/wt):UCCCCUCUUUAGCUUGGAGAAG_14.27/5.64
miRNA Position in PremiRNA:6-27
miRNA* sequence and Count(AGO1/wt):UCUGCAACCGGAAAGGGGAGC_4.76/5.24
miRNA Position in PremiRNA:179-199
miRNA annonation:ath-miR853

27. Degradome supported cutsite(s) in Genome:19073542
PremiRNA Locus in Genome:>Chr3(19073446-19073545)_plus strand
PremiRNA
sequence:CUUUGAUAUUGGCCUGGUUCACUCAGAUCUUACCUGACCACACACGUAGAUAUACAUUAUUCUCUCUAGAUUAUCUGAUUGAGCCGCGCCA
AUAUCUCAG
PremiRNA secondary structure:((..(((((((((.(((((((.((((((.((((.((......)).))))..........(((....)))..)))))).))))))).)))))))))..))
Degradome supported cutsite(s) in premiRNA:97
miRNA sequence and Count(AGO1/wt):UGAUUGAGCCGCGCCAAUAUC_2174.29/405.65
miRNA Position in PremiRNA:76-96
miRNA* sequence and Count(AGO1/wt):UAUUGGCCUGGUUCACUCAGA_835.67/95.67
miRNA Position in PremiRNA:7-27
miRNA annonation:ath-miR171a

28. Degradome supported cutsite(s) in Genome:20587991
PremiRNA Locus in Genome:>Chr3(20587914-20588016)_plus strand
PremiRNA
sequence:UGCUAUUGCAACAUCUUCAAGAUUCAGAAAUCAGAUUCUCUUAUGGGUUUUCUUUUGAGCCUUUAUUUUUUGGUUUGAGAAUCUUGAUGAU
GCUGCAGCGGCA
PremiRNA secondary structure:(((..(((((.((((.(((((((((..((((((((......((.((((((......)))))).))...))))))))..))))))))).)))).)))))..)))
Degradome supported cutsite(s) in premiRNA:78
miRNA sequence and Count(AGO1/wt):AGAAUCUUGAUGAUGCUGCAG_2861.5/617.94
miRNA Position in PremiRNA:78-98
miRNA* sequence and Count(AGO1/wt):GCAACAUCUUCAAGAUUCAGA_0.5/51.76
miRNA Position in PremiRNA:8-28
miRNA annonation:ath-miR172d/ath-miR172c

29. Degradome supported cutsite(s) in Genome:22922301/22922321
PremiRNA Locus in Genome:>Chr3(22922206-22922325)_plus strand

PremiRNA sequence:UUGAGGGGACUGUUGUCUGGCUCGAGGACUCUUAUUCUAAUACAAUCUCAUUUGAAUACAUUCAGAUCUGAUGAUUGAUUAGGGUUUUAGUGUCGUCGGACCAGGCUUCAUUCCCCCCAA

PremiRNA secondary structure:(((.(((((.((..((((((..(((.(((.((.((((((((.((((((((((((((....))))))..))).))))))))))))...)).))).)))..))))))..)).))))).)))

Degradome supported cutsite(s) in premiRNA:96/116

miRNA sequence and Count(AGO1/wt):UCGGACCAGGCUUCAUUCCCC_76756.02/3261.89

miRNA Position in PremiRNA:96-116

miRNA* sequence and Count(AGO1/wt):GGACUGUUGUCUGGCUCGAGG_0.25/87.82

miRNA Position in PremiRNA:7-27

miRNA annonation:ath-miR166b/ath-miR166f/ath-miR166a/ath-miR166g/ath-miR166c/ath-miR166d/ath-miR166e

30. Degradome supported cutsite(s) in Genome:23406177

PremiRNA Locus in Genome:>Chr3(23406172-23406272)_plus strand

PremiRNA sequence:ACAAGUGAAGCUGCCAGCAUGAUCUAUCUUUGGUUAAGAGAUGAAUGUGGAAACAUAUUGCUUAAACCCAAGCUAGGUCAUGCUCUGACAGCCUCACUCCU

PremiRNA secondary structure:(..(((((.(((((.((((((((((((.(((.((((..(((...((((((....)))))).))).)))).))).)))))))))))..).)))).)))))..)

Degradome supported cutsite(s) in premiRNA:6

miRNA sequence and Count(AGO1/wt):UGAAGCUGCCAGCAUGAUCUA_6554.16/1991.78

miRNA Position in PremiRNA:6-26

miRNA* sequence and Count(AGO1/wt):GGUCAUGCUCUGACAGCCUCACU_0.5/13.9

miRNA Position in PremiRNA:76-98

miRNA annonation:ath-miR167b/ath-miR167a

31. Degradome supported cutsite(s) in Genome:9889047

PremiRNA Locus in Genome:>Chr4(9888982-9889070)_plus strand

PremiRNA sequence:GUCGUGCCUGGCUCCCUGUAUGCCACAAGAAAACAUCGAUUUAGUUUCAAAAUCGAUCACUAGUGGCGUACAGAGUAGUCAAGCAUGAC

PremiRNA secondary structure:(((((((.(((((.((((((((((((.((.....((((((((.......))))))))..)).))))))))))).).))))).)))))))

Degradome supported cutsite(s) in premiRNA:66

miRNA sequence and Count(AGO1/wt):UGCCUGGCUCCCUGUAUGCCA_2413.62/120.24

miRNA Position in PremiRNA:5-25

miRNA* sequence and Count(AGO1/wt):GCGUACAGAGUAGUCAAGCAUG_0/4.83

miRNA Position in PremiRNA:66-87
miRNA annonation:ath-miR160a/ath-miR160c/ath-miR160b

32. Degradome supported cutsite(s) in Genome:10578652/10578737/10578758
PremiRNA Locus in Genome:>Chr4(10578647-10578760)_plus strand
PremiRNA
sequence:CGGAUUCGCUUGGUGCAGGUCGGGAACCAAUUCGGCUGACACAGCCUCGUGACUUUUAAACCUUUAUUGGUUUGUGAGCAGGGAUUGGAUC
CCGCCUUGCAUCAACUGAAUCGG
PremiRNA secondary structure:(.((((((.((((((((((.(((((.((((((((((((...)))))...((.(((.((((((......)))))).))))).))))))).))))).)))))))))).)))))).)
Degradome supported cutsite(s) in premiRNA:6/91/112
miRNA sequence and Count(AGO1/wt):UCGCUUGGUGCAGGUCGGGAA_1012.92/266.47
miRNA Position in PremiRNA:6-26
miRNA* sequence and Count(AGO1/wt):CCCGCCUUGCAUCAACUGAAU_9.26/270.7
miRNA Position in PremiRNA:91-111
miRNA annonation:ath-miR168b/ath-miR168a

33. Degradome supported cutsite(s) in Genome:12353109/12353130
PremiRNA Locus in Genome:>Chr4(12352440-12353134)_plus strand
PremiRNA
sequence:GUUCUAAAUUUGAAGCUUAUAAAAACCCAUCACUACUUUUGCAUACUUGUAUCCGCAGUGUAUUUCCUCGCAUCUACCAUCCCUUUUCUACG
CCUCUCUCCCUCUCUCUCUUUCUCCAUCAAAUCUUGUUUUGUUCAAACUCUCUCUCUCUCAUCUAUUCUCUCCAUACAAUACAUGAAUAUACAUACAU
ACCAUCAUCUUCUUUUCCCAUCUCUAGUUUUUCAUCAAUCUUCUGAUGUUCCAAACGCUCUAUCUCUUCAUAUACAUACAUACGAAUAUAUUAUUGU
UGUCAUAGAUCCAUUUAGAAUCACUUUAGCUUUUAGAUGAGAUCUAGGGUUUCUUUGUUUUCUUUCAAAUUUUGUUGCAUAUUCUUCUAAAUCAUG
GUUUUUCGCUUGCUAGGUUAUAGAUCCAUGCAAAUAUGGAGUAGAUGUACAAACACACGCUCGGACGCAUAUUACACAUGUUCAUACACUUAAUACU
CGCUGUUUUGAAUUGAUGUUUUAGGAAUAUAUAUGUAGAGAGAGCUUCCUUGAGUCCAUUCACAGGUCGUGAUAUGAUUCAAUUAGCUUCCGACUC
AUUCAUCCAAAUACCGAGUCGCCAAAAUUCAAACUAGACUCGUUAAAUGAAUGAAUGAUGCGGUAGACAAAUUGGAUCAUUGAUUCUCUUUGAUUG
GACUGAAGGGAGCUCCCUCUCUC
PremiRNA secondary
structure:(....................................((((((..((..((((.(((((........((((((((...((((......)))).............................((((((.......((((...(((.....(((......................((((..(((((......))))).
...)))).....................(((.((((........)))))))..((((((((((((((.(......).)))))))))).)))))).))).....))).....)))).......((((((.....((((((((.((((.........)))))))).))))...)))))).)))))))))))))........)))))))))..)).........((((((..(((
.(((((..(((......)))))))))))).....)))))).))))))(((.((((((((((.((((((.(((.(((..((.(.(((((((((((.(((.(((..((((((((.((..((.((((((................)))))).))..)).))))))))..))).)).).))))))))))).))..))).))).)))))).)))))))))).)))...)

Degradome supported cutsite(s) in premiRNA:670/691
miRNA sequence and Count(AGO1/wt):UUGGACUGAAGGGAGCUCCCU_20449.58/4223.04

miRNA Position in PremiRNA:670-690
miRNA* sequence and Count(AGO1/wt):AGAGCUUCCUUGAGUCCAUUC_2.5/16.11
miRNA Position in PremiRNA:521-541
miRNA annonation:ath-miR319b/ath-miR319a

34. Degradome supported cutsite(s) in Genome:12625128/12625742
PremiRNA Locus in Genome:>Chr4(12625123-12625744)_plus strand
PremiRNA
sequence:AUGUCUAGACCAUUUGUGAGAAGGGAGUUUUUGUUUACACCAAUACCCCCCAGUCUCUAAAUUUGUAAGAAGUAUUAUGCUCAAUUAAGGA
ACAUAGCUAGUUCGACAUACCAUACUACCCUUUUUAAAACUAUCCUAUAUGUUUGAUGCUAGCAUAGCGUAAUUUUGUGUUCUCAUGGUGCAGCAGG
GUUAGUUUAUUGUGUACCUCUAGAUAUAUAUUCUCUUGCUGCGUAGGGUUCCCAAGCUGCCAAAAACUUUUAAAAAUUAGUGAUCUGUUCCCCAAAC
CCCCAUUCAAUAAGAAAGGUCUACCUGUAGCUCACAGUCACAGCUAUUAGAGCUGUCCUUGCUUCUUUGGGUUUACUGUUUUAGUUAAUUAGUUACC
CUAUGAAAGUCUGAUCCUUCAGAAGUUAGGUAUAGAAGUAAGUCGAUUAAGUUCAUUCAUGACUUUUCCAGAUGUUGCAUACAUAACUUUUUUUAU
UUGUAUUCGAACUAGUCCAAGCCGAUUCUCAAAAUCAUAUAAAAUCAUUUACACCUUGGUCAUGGUAGCUAAGAAUAUUGUAUCUAAAAUUGGGGA
GUGGGGAGAUGUUUGGUUAUAUUCCCUUCUCAUCGAUGGUCUAGAUGU
PremiRNA secondary
structure:(((((((((((((.((((((((((((((..........(((((((.((((((.(((((((.((((.....((((((..((((.(((((((......((((((((((.((((..(((((...(((((((((......((((......((((((...))))))).....))))......((((((((((((...((..(((.((((........)))
)))))..))))))))))...((....)).....)))).....))))))))).)))))......................((((((((((((.....((((((((......((((((.....)))))).(((((((((..((((..((((...........)))).)))))..(((...(((((....)))))..))).....)))))))))..((..((((((.......))))
).))..)).))))))......))))))))))))).))))))))))))))).......((((.....)))).................)))))))....).)))....))))))).....)))).))))))).))))).).)).)))))...))))))))))))).))))))))))))))
Degradome supported cutsite(s) in premiRNA:6/620
miRNA sequence and Count(AGO1/wt):UAGACCAUUUGUGAGAAGGGA_524.48/742.01
miRNA Position in PremiRNA:6-26
miRNA* sequence and Count(AGO1/wt):CCUUCUCAUCGAUGGUCUAGA_10.51/71.1
miRNA Position in PremiRNA:599-619
miRNA annonation:ath-miR824

35. Degradome supported cutsite(s) in Genome:15074945
PremiRNA Locus in Genome:>Chr4(15074940-15075033)_plus strand
PremiRNA
sequence:AAAACUGACAGAAGAGAGUGAGCACAUGCAGGCACUGUUAUGUGUCUAUAACUUUGCGUGUGCGUGCUCACCUCUCUUUCUGUCAGUUGCC
UAU
PremiRNA secondary structure:(.((((((((((((((((((((((((..(((.(((..((((((....))))))..))).)))..))))))).))))).)))))))))))).....)
Degradome supported cutsite(s) in premiRNA:6
miRNA sequence and Count(AGO1/wt):UGACAGAAGAGAGUGAGCAC_199.78/108.36

miRNA Position in PremiRNA:6-25
miRNA* sequence and Count(AGO1/wt):GCUCACCUCUCUUUCUGUCAGU_4.51/21.15
miRNA Position in PremiRNA:66-87
miRNA annonation:ath-miR156e/ath-miR156f/ath-miR156d/ath-miR156a/ath-miR156c/ath-miR156b

36. Degradome supported cutsite(s) in Genome:287586
PremiRNA Locus in Genome:>Chr5(287581-287739)_plus strand
PremiRNA
sequence:CAAGAUGGAGAAGCAGGGCACGUGCAUUACUAGCUCAUAUAUACACUCUCACCACAAAUGCGUGUAUAUAUGCGGAAUUUUGUGAUAUAGA
UGUGUGUGUGUGUUGAGUGUGAUGAUAUGGAUGAGUUAGUUCUUCAUGUGCCCAUCUUCACCAUCAUG
PremiRNA secondary structure:((.(((((.((((..(((((((((....((((((((((.(((((((.((((.((((.((((((.(((((.(((((....)))))))))).))))))...)))).)))).)))....)))).))))))))))....)))))))))..)))).))))).))

Degradome supported cutsite(s) in premiRNA:6
miRNA sequence and Count(AGO1/wt):UGGAGAAGCAGGGCACGUGCA_984.13/151.06
miRNA Position in PremiRNA:6-26
miRNA* sequence and Count(AGO1/wt):AUGUGCCCAUCUUCACCAUC_0/0
miRNA Position in PremiRNA:140-156
miRNA annonation:ath-miR164b/ath-miR164a

37. Degradome supported cutsite(s) in Genome:3867213
PremiRNA Locus in Genome:>Chr5(3867208-3867312)_plus strand
PremiRNA
sequence:GGAGGUGACAGAAGAGAGUGAGCACACAUGGUGGUUUCUUGCAUGCUUUUUUGAUUAGGGUUUCAUGCUUGAAGCUAUGUGUGCUUACUCU
CUCUCUGUCACCCC
PremiRNA secondary structure:((.((((((((((((((((((((((((((((...(((..(((((((((.......))))...)))))..))))))))))))))))))))))))).)))))))))))
Degradome supported cutsite(s) in premiRNA:6
miRNA sequence and Count(AGO1/wt):UGACAGAAGAGAGUGAGCAC_199.78/108.36
miRNA Position in PremiRNA:6-25
miRNA* sequence and Count(AGO1/wt):GCUUACUCUCUCUCUGUCACC_0/0.6
miRNA Position in PremiRNA:83-103
miRNA annonation:ath-miR156e/ath-miR156f/ath-miR156d/ath-miR156a/ath-miR156c/ath-miR156b

38. Degradome supported cutsite(s) in Genome:4691128
PremiRNA Locus in Genome:>Chr5(4691027-4691132)_plus strand

PremiRNA
sequence:CUCGACAGGGUUGAUAUGAGAACACACGAGUAAUCAACGGCUGUAAUGACGCUACGUCAUUGUUACAGCUCUCGUUUUCAUGUGUUCUCAG
GUCACCCCUGCUGAG
PremiRNA secondary structure:((((.(((((.((((.((((((((((.(((.(((....((((((((((((((...))))))...)))))))...)))))).)))))))))).)))).))))).))))
Degradome supported cutsite(s) in premiRNA:102
miRNA sequence and Count(AGO1/wt):UGUGUUCUCAGGUCACCCCUG_120.92/0.81
miRNA Position in PremiRNA:81-101
miRNA* sequence and Count(AGO1/wt):GGGUUGAUAUGAGAACACACG_0.75/3.42
miRNA Position in PremiRNA:8-28
miRNA annonation:ath-miR398c/ath-miR398b

39. Degradome supported cutsite(s) in Genome:9098879/9098897
PremiRNA Locus in Genome:>Chr5(9098806-9098901)_plus strand
PremiRNA
sequence:UCAAUAUAUAUAGUCCAAUCUAUUGAAGUACUAGUACACCAGCUCUAAUAAGCUGAUGUGGGUAAGUAGUUCAAUAGAUUGGACUAUGUAU
AUUAA
PremiRNA secondary structure:(.((((((((((((((((((((((((((.((((.(..((((((((......)))))..)))..).)))).)))))))))))))))))))))))))).)
Degradome supported cutsite(s) in premiRNA:74/92
miRNA sequence and Count(AGO1/wt):UCAAUAGAUUGGACUAUGUAU_0.25/12.49
miRNA Position in PremiRNA:71-91
miRNA* sequence and Count(AGO1/wt):AUAUAGUCCAAUCUAUUGAAG_0/0
miRNA Position in PremiRNA:8-28
miRNA annonation:ath-miR860

40. Degradome supported cutsite(s) in Genome:9136126
PremiRNA Locus in Genome:>Chr5(9136121-9136223)_plus strand
PremiRNA
sequence:GAUGGUGACAGAAGAGAGUGAGCACACAUGGUGGCUUUCUUGCAUAUUUGAAGGUUCCAUGCUUGAAGCUAUGUGUGCUCACUCUCUAUCC
GUCACCCCCUUC
PremiRNA secondary structure:((.((((((.(((((((((((((((((((((((....(((..((((............))))..)))))))))))))))))))))))))).)).))))))....))
Degradome supported cutsite(s) in premiRNA:6
miRNA sequence and Count(AGO1/wt):UGACAGAAGAGAGUGAGCAC_199.78/108.36
miRNA Position in PremiRNA:6-25
miRNA* sequence and Count(AGO1/wt):GCUCACUCUCUAUCCGUCACC_0/0.81

miRNA Position in PremiRNA:77-97
miRNA annonation:ath-miR156e/ath-miR156f/ath-miR156d/ath-miR156a/ath-miR156c/ath-miR156b

41. Degradome supported cutsite(s) in Genome:9852684
PremiRNA Locus in Genome:>Chr5(9852679-9852770)_plus strand
PremiRNA
sequence:CUUGAUGGAGAAGCAGGGCACGUGCGAACACAAAUGAAAUCGAUCGGUACUUGUUGAUCAUAUUUUCGCACGUGUUCUACUACUCCAACAC
G
PremiRNA secondary structure:(.((.(((((.((.((((((((((((((..(....).....((((((......)))))).....)))))))))))))).)).))))).)).)
Degradome supported cutsite(s) in premiRNA:6
miRNA sequence and Count(AGO1/wt):UGGAGAAGCAGGGCACGUGCG_8.01/2.82
miRNA Position in PremiRNA:6-26
miRNA* sequence and Count(AGO1/wt):CACGUGUUCUACUACUCCAAC_1.75/11.68
miRNA Position in PremiRNA:69-89
miRNA annonation:ath-miR164c

42. Degradome supported cutsite(s) in Genome:13611904
PremiRNA Locus in Genome:>Chr5(13611803-13611927)_plus strand
PremiRNA
sequence:UACUUUUCCACAGCUUUCUUGAACUUUCUUUUUCAUUUCCAUUGUUUUUUUCUUAAACAAAAGUAAGAAGAAAAAAAACUUUAAGAUUAAG
CAUUUUGGAAGCUCAAGAAAGCUGUGGGAAAACA
PremiRNA secondary structure:(..(((((((((((((((((((.(((((................(((((((((((........)))))))))))....(((.......)))......))))).)))))))))))))))))))..)
Degradome supported cutsite(s) in premiRNA:102
miRNA sequence and Count(AGO1/wt):UUCCACAGCUUUCUUGAACUU_7402.34/2931.98
miRNA Position in PremiRNA:6-26
miRNA* sequence and Count(AGO1/wt):GCUCAAGAAAGCUGUGGGAAA_71.6/68.08
miRNA Position in PremiRNA:102-122
miRNA annonation:ath-miR396b

43. Degradome supported cutsite(s) in Genome:15758111/15758189
PremiRNA Locus in Genome:>Chr5(15758106-15758191)_plus strand
PremiRNA
sequence:UUAGAAGAAGCAAAAUGACGACUCGGACAACAAUAAUACAAAACUAAUACUUAUUGUUGUCGGAGUCGUCAUUUUGCAUCUUCUAA

PremiRNA secondary structure:((((((((.(((((((((((((((.(((((((((((..............))))))))))).))))))))))))))).))))))))
Degradome supported cutsite(s) in premiRNA:6/84
miRNA sequence and Count(AGO1/wt):AGAAGCAAAAUGACGACUCGG_1.25/1.41
miRNA Position in PremiRNA:6-26
miRNA* sequence and Count(AGO1/wt):GAGUCGUCAUUUUGCAUCUUC_0.5/2.62
miRNA Position in PremiRNA:63-83
miRNA annonation:ath-miR3933

44. Degradome supported cutsite(s) in Genome:17516378/17516399
PremiRNA Locus in Genome:>Chr5(17516303-17516403)_plus strand
PremiRNA
sequence:GUUCAGGUGAAUGAUGCCUGGCUCGAGACCAUUCAAUCUCAUGAUCUCAUGAUUAUAACGAUGAUGAUGAUGAUGUCGGACCAGGCUUCAUUCCCCUCAAC
PremiRNA secondary structure:(((.(((.((((((.((((((..(((.(.((((..(((((((((((....))))))...)).)))...)))).).)))..)))))).)))))).))).)))
Degradome supported cutsite(s) in premiRNA:76/97
miRNA sequence and Count(AGO1/wt):UCGGACCAGGCUUCAUUCCCC_76756.02/3261.89
miRNA Position in PremiRNA:76-96
miRNA* sequence and Count(AGO1/wt):UGAAUGAUGCCUGGCUCGAGA_17.52/2.42
miRNA Position in PremiRNA:8-28
miRNA annonation:ath-miR166b/ath-miR166f/ath-miR166a/ath-miR166g/ath-miR166c/ath-miR166d/ath-miR166e

45. Degradome supported cutsite(s) in Genome:19567227
PremiRNA Locus in Genome:>Chr5(19567222-19567349)_plus strand
PremiRNA
sequence:UACAGUUGAAAUUGUAGAUUUCGUACAUGACAAUAAUUUGCUCGAGCAUCACUUUCAUGAUUUUGUGGUCUGUGUUGCCGUAUUUGAGAGCAUACAUUUCAUGUUCGAAAUCUACGAUUUUAACUCUA
PremiRNA secondary structure:((.((((((((((((((((((((.((((((.(((....(((((..(((.(((..((((......))))...))).)))..........)))))...)))))))).)))))))))))))))))))).))
Degradome supported cutsite(s) in premiRNA:6
miRNA sequence and Count(AGO1/wt):UUGAAAUUGUAGAUUUCGUAC_2.25/1.21
miRNA Position in PremiRNA:6-26
miRNA* sequence and Count(AGO1/wt):UCGAAAUCUACGAUUUUAACU_35.8/16.72
miRNA Position in PremiRNA:105-125
miRNA annonation:ath-miR4243

46. Degradome supported cutsite(s) in Genome:21161609
PremiRNA Locus in Genome:>Chr5(21161504-21161634)_plus strand
PremiRNA
sequence:AUGAAUUGUCAGUAUAAAUCUUUGAUCUGAUACUCUAUUAGUUAGUAUGUGAAUUUCUUUUUAACAAUUUUCUUAUUCAUGCGAGAGCUAUCAAUAAGUAUUAGAACAAAGUUUUAUACUGACAAUUCAAU
PremiRNA secondary structure:((((((((((((((((((.(((((.(((((((((....(((((.(((((.(((................)))....)))))...)))))......))))))))).))))).)))))))))))))))))).))
Degradome supported cutsite(s) in premiRNA:106
miRNA sequence and Count(AGO1/wt):ACAAAGUUUUAUACUGACAAU_15.02/19.34
miRNA Position in PremiRNA:106-126
miRNA* sequence and Count(AGO1/wt):UGUCAGUAUAAAUCUUUGAUC_21.53/7.05
miRNA Position in PremiRNA:7-27
miRNA annonation:ath-miR4245

47. Degradome supported cutsite(s) in Genome:23636961
PremiRNA Locus in Genome:>Chr5(23636956-23637057)_plus strand
PremiRNA
sequence:CUAUAAAGCUCAGGAGGGAUAGCGCCAUGGCUCACCAGUGCUGUAUGUUUUGUAUAUGCGUACAUGUAUAUCUGUUGGCGCUAUCCAUCCUGAGUUCCAUAG
PremiRNA secondary structure:((((..(((((((((.(((((((((((.((....)).(((((((((((.........))))))).)))).......))))))))))).)))))))))..))))
Degradome supported cutsite(s) in premiRNA:6
miRNA sequence and Count(AGO1/wt):AAGCUCAGGAGGGAUAGCGCC_181.75/459.42
miRNA Position in PremiRNA:6-26
miRNA* sequence and Count(AGO1/wt):CGCUAUCCAUCCUGAGUUCCA_0.25/7.05
miRNA Position in PremiRNA:79-99
miRNA annonation:ath-miR390b/ath-miR390a

48. Degradome supported cutsite(s) in Genome:24293160/24293211
PremiRNA Locus in Genome:>Chr5(24293156-24293233)_plus strand
PremiRNA sequence:UUGCUUCGCAGGAGAGAUAGCGCCAUCACCUCUUCUAAGAAGUUAACUAGUGGUGACGGUAUCUCUCCUACGUAGCAA

PremiRNA secondary structure:(((((.((.((((((((((.((.((((((..((((...))))........)))))).)).)))))))))).)).)))))
Degradome supported cutsite(s) in premiRNA:5/56
miRNA sequence and Count(AGO1/wt):UUCGCAGGAGAGAUAGCGCCA_50.82/38.27
miRNA Position in PremiRNA:5-25

miRNA* sequence and Count(AGO1/wt):ACGGUAUCUCUCCUACGUAGC_2.5/53.98
miRNA Position in PremiRNA:56-76
miRNA annonation:ath-miR391

49. Degradome supported cutsite(s) in Genome:78932/78953
PremiRNA Locus in Genome:>Chr1(79037-78927)_minus strand
PremiRNA
sequence:GUUGAGGGGAAUGUUGUCUGGAUCGAGGAUAUUAUAGAUAUAUACAUGUGUAUGUUAAUGAUUCAAGUGAUCAUAGAGAGUAUCCUCGGACCAGGCUUCAUCCCCCCCAAC
PremiRNA secondary structure:((((.((((.(((..((((((.(((((((((((...((((((((....)))))))).((((((.....))))))....))))))))))).))))))..))).)))).))))
Degradome supported cutsite(s) in premiRNA:107/86
miRNA sequence and Count(AGO1/wt):UCGGACCAGGCUUCAUCCCCC_26140.03/750.87
miRNA Position in PremiRNA:86-106
miRNA* sequence and Count(AGO1/wt):GGAAUGUUGUCUGGAUCGAGG_0.5/21.75
miRNA Position in PremiRNA:8-28
miRNA annonation:ath-miR165b/ath-miR165a

50. Degradome supported cutsite(s) in Genome:234017
PremiRNA Locus in Genome:>Chr1(234152-234013)_minus strand
PremiRNA
sequence:AUGAUGCGCAAAUGCGGAUAUCAAUGUAAAUCAGGACAACAAACCAAUAGAGGUGCUCAGCGAAAACAACAAAAUGUGAAAAGGGUUUAUCAGAAACAUUGGGGUCAUGAUAUACCUUUAUAUCCGCAUUUGCGCAUCAU
PremiRNA secondary structure:(((((((((((((((((((((.((.(((.((((.(((..(((.......((...((((.......(((......))).....))))...)).......)))..))).)))).))).)).)))))))))))))))))))))
Degradome supported cutsite(s) in premiRNA:137
miRNA sequence and Count(AGO1/wt):CUUUAUAUCCGCAUUUGCGCA_18.53/20.14
miRNA Position in PremiRNA:116-136
miRNA* sequence and Count(AGO1/wt):CGCAAAUGCGGAUAUCAAUGU_0.25/4.83
miRNA Position in PremiRNA:7-27
miRNA annonation:ath-miR2112-3p

51. Degradome supported cutsite(s) in Genome:3961367/3961388
PremiRNA Locus in Genome:>Chr1(3961454-3961365)_minus strand

PremiRNA sequence:CGCGAGAUAUUAGUGCGGUUCAAUCAAAUAGUCGUCCUCUUAACUCAUGGAGAACGGUGUUGUUCGAUUGAGCCGUGCCAAUAUCACGCG

PremiRNA secondary structure:((((.((((((.(..((((((((((.(((((((((.((((........)))).)))))..))))).))))))))))..).)))))).))))
Degradome supported cutsite(s) in premiRNA:89/68
miRNA sequence and Count(AGO1/wt):UUGAGCCGUGCCAAUAUCACG_736.28/66.47
miRNA Position in PremiRNA:68-88
miRNA* sequence and Count(AGO1/wt):AGAUAUUAGUGCGGUUCAAUC_21.78/26.99
miRNA Position in PremiRNA:5-25
miRNA annonation:ath-miR171c/ath-miR171b

52. Degradome supported cutsite(s) in Genome:4820431
PremiRNA Locus in Genome:>Chr1(4820499-4820405)_minus strand
PremiRNA sequence:UCUCCUCUUCUCCAAAUAGUUUAGGUUAGCUGACAUAUAAUAAUUCAAUAGACCCCAGUUUCUCCAGAUAACCUAACUAUUUGAGAAGAAGUGA
PremiRNA secondary structure:((.(.((((((((.((((((((((.((((((.(((............((....))............))).))))))))))))))).)))))))).).))
Degradome supported cutsite(s) in premiRNA:70
miRNA sequence and Count(AGO1/wt):UCUUCUCCAAAUAGUUUAGGUU_4.76/5.44
miRNA Position in PremiRNA:6-27
miRNA* sequence and Count(AGO1/wt):CCUAACUAUUUUGAGAAGAAG_3.76/2.42
miRNA Position in PremiRNA:72-92
miRNA annonation:ath-miR830-5p

53. Degradome supported cutsite(s) in Genome:11834118
PremiRNA Locus in Genome:>Chr1(11834159-11834089)_minus strand
PremiRNA sequence:AUUUGAACCUGUCAAUUGGUAUCAAAGCUUCUAAAUCUUUGAAGCUCUGAUACCAAAUGAUGGAAUCAAAU

PremiRNA secondary structure:((((((..((((((.((((((((((.((((((.........)))))).)))))))))).))))))..))))))
Degradome supported cutsite(s) in premiRNA:43
miRNA sequence and Count(AGO1/wt):AGCUCUGAUACCAAAUGAUGGAAU_0.75/9.67
miRNA Position in PremiRNA:43-66
miRNA* sequence and Count(AGO1/wt):CCUGUCAAUUGGUAUCAAAGCUUC_0/0.6
miRNA Position in PremiRNA:8-31

miRNA annonation:ath-miR829.1

54. Degradome supported cutsite(s) in Genome:23345389/23345408
PremiRNA Locus in Genome:>Chr1(23345506-23345382)_minus strand
PremiRNA
sequence:AGUUUUAGGGCGCCUCUCCAUUGGCAGGUCCUUUACUUCCAAAUAUACACAUACAUAUAUGAAUAUCGAAAAUUUCCGAUGAUCGAUUUAUAAAUGACCUGCCAAAGGAGAGUUGCCCUGAAACU
PremiRNA secondary structure:((((((((((((.((((((.((((((((((.((((.......(((((........)))))((.(((((........))))).))......)))).)))))))))).)))))).))))))))))))
Degradome supported cutsite(s) in premiRNA:119/100
miRNA sequence and Count(AGO1/wt):UGCCAAAGGAGAGUUGCCCUG_353.24/18.53
miRNA Position in PremiRNA:100-120
miRNA* sequence and Count(AGO1/wt):UUAGGGCGCCUCUCCAUUGGC_0.75/0
miRNA Position in PremiRNA:8-28
miRNA annonation:ath-miR399b/ath-miR399c

55. Degradome supported cutsite(s) in Genome:27713234/27713255
PremiRNA Locus in Genome:>Chr1(27713416-27713233)_minus strand
PremiRNA
sequence:GUAGAGCUCCUUAAAGUUCAAACAUGAGUUGAGCAGGGUAAAGAAAAGCUGCUAAGCUAUGGAUCCCAUAAGCCCUAAUCCUUGUAAAGUAAAAAAGGAUUUGGUUAUAUGGAUUGCAUAUCUCAGGAGCUUUAACUUGCCCUUUAAUGGCUUUUACUCUUCUUUGGAUUGAAGGGAGCUCUAC

PremiRNA secondary
structure:((((((((((((..((((((((...((((.(((((((((((...((((((.((.((.((((.(..(((((((((..(((((((..........))))))).)))).)))))..).)))).)).)).))))))...)))))))......))))..))))...))))))))..))))))))))))
Degradome supported cutsite(s) in premiRNA:184/163
miRNA sequence and Count(AGO1/wt):UUUGGAUUGAAGGGAGCUCUA_49830.64/4532.41
miRNA Position in PremiRNA:163-183
miRNA* sequence and Count(AGO1/wt):GAGCUCCUUAAAGUUCAAACA_0/2.82
miRNA Position in PremiRNA:4-24
miRNA annonation:ath-miR159a

56. Degradome supported cutsite(s) in Genome:22930095/22930116
PremiRNA Locus in Genome:>Chr1(22930184-22930090)_minus strand

PremiRNA
sequence:AAAUACGAGAUAUUGGUGCGGUUCAAUCAGAAAACCGUACUCUUUUGUUUUAAAGAUCGGUUUAUUUGAUUGAGCCGUGCCAAUAUCACGC
GUUU
PremiRNA secondary structure:(((..((.((((((((..((((((((((((((((((...(((((......))))).)))))).))))))))))))..)))))))).))..)))
Degradome supported cutsite(s) in premiRNA:91/70
miRNA sequence and Count(AGO1/wt):UUGAGCCGUGCCAAUAUCACG_736.28/66.47
miRNA Position in PremiRNA:70-90
miRNA* sequence and Count(AGO1/wt):AGAUAUUGGUGCGGUUCAAUC_23.53/22.96
miRNA Position in PremiRNA:8-28
miRNA annonation:ath-miR171c/ath-miR171b

57. Degradome supported cutsite(s) in Genome:4142352/4142464
PremiRNA Locus in Genome:>Chr2(4142468-4142328)_minus strand
PremiRNA
sequence:UAUUCUUCCACAGCUUUCUUGAACUGCAAAACUUCUUCAGAUUUUUUUUUUUUCUUUUGAUAUCUCUUACGCAUAAAAUAGUGAUUUUCU
UCAUAUCUCUGCUCGAUUGAUUUGCGGUUCAAUAAAGCUGUGGGAAGAUA
PremiRNA secondary structure:(((..((((((((((((.((((((((((((.(.((..((((............((....))....................((((......))))...))))...))..).)))))))))))).))))))))))))..)))
Degradome supported cutsite(s) in premiRNA:118/6
miRNA sequence and Count(AGO1/wt):UUCCACAGCUUUCUUGAACUG_1451.03/390.54
miRNA Position in PremiRNA:6-26
miRNA* sequence and Count(AGO1/wt):GUUCAAUAAAGCUGUGGGAAG_37.3/68.68
miRNA Position in PremiRNA:118-138
miRNA annonation:ath-miR396a

58. Degradome supported cutsite(s) in Genome:10676554
PremiRNA Locus in Genome:>Chr2(10676558-10676443)_minus strand
PremiRNA
sequence:GAAACUGACAGAAGAGAGUGAGCACACAAAGGCAAUUUGCAUAUCAUUGCACUUGCUUCUCUUGCGUGCUCACUGCUCUUUCUGUCAGAUU
CCGGUGCUGAUCUCUUUGGCCUGUC
PremiRNA secondary structure:((..(((((((((((((((((((((.(((((((((..((((......)))).))))))...))).))))))))).))).)))))))))....(((.((..(.....)..)))))))
Degradome supported cutsite(s) in premiRNA:6
miRNA sequence and Count(AGO1/wt):UGACAGAAGAGAGUGAGCAC_199.78/108.36
miRNA Position in PremiRNA:6-25
miRNA* sequence and Count(AGO1/wt):GCUCACUGCUCUUUCUGUCAGA_0/4.83

miRNA Position in PremiRNA:68-89
miRNA annonation:ath-miR156e/ath-miR156f/ath-miR156d/ath-miR156a/ath-miR156c/ath-miR156b

59. Degradome supported cutsite(s) in Genome:3366369
PremiRNA Locus in Genome:>Chr3(3366416-3366349)_minus strand
PremiRNA sequence:UGCUUCUUUGUCUACAAUUUUGGAAAAAGUGAUGACGCCAUUGCUCUUUCCCAAAUGUAGACAAAGCA
PremiRNA secondary structure:(((((...((((((((.(((.(((((.((..(((....)))..)).))))).))))))))))))))))
Degradome supported cutsite(s) in premiRNA:49
miRNA sequence and Count(AGO1/wt):UCCCAAAUGUAGACAAAGCA_41217.85/13378.09
miRNA Position in PremiRNA:49-68
miRNA* sequence and Count(AGO1/wt):CUUUGUCUACAAUUUUGGAAA_23.28/50.55
miRNA Position in PremiRNA:3-26
miRNA annonation:ath-miR158a

60. Degradome supported cutsite(s) in Genome:3599812
PremiRNA Locus in Genome:>Chr3(3599897-3599787)_minus strand
PremiRNA
sequence:GCUGUUGGAGCAUCAUCAAGAUUCACAAAUCAUCAAGUAUUCGUGUAAAUAAACCCAUUUAUGAUUAGAUUUUUGAUGUAUGUAUGAGAAU
CUUGAUGAUGCUGCAGCUGC
PremiRNA secondary structure:((.((((.((((((((((((((((.((.(((((((((...((.(((((((......)))))))....))..))))))).))...)).))))))))))))))))).)))).))
Degradome supported cutsite(s) in premiRNA:87
miRNA sequence and Count(AGO1/wt):AGAAUCUUGAUGAUGCUGCAG_2861.5/617.94
miRNA Position in PremiRNA:87-107
miRNA* sequence and Count(AGO1/wt):GGAGCAUCAUCAAGAUUCACA_0/1.41
miRNA Position in PremiRNA:7-27
miRNA annonation:ath-miR172d/ath-miR172c

61. Degradome supported cutsite(s) in Genome:4359010
PremiRNA Locus in Genome:>Chr3(4359207-4359007)_minus strand
PremiRNA
sequence:GUGUGCAGCCAAGGAUGACUUGCCGAUUUAAAUGAUCUUUCUUUAUACUCUAUUAAGACAAUUUAGUUUCAAACUUUUUUUUUUUUUUUU
UUUGAAGGAUUCAGGAAGAAAUUAGGAUAUAUUAUUCCGUAUAAAAUACAAGAUAUAUAAAACCAAAAAGAAAAAGUAACAUGAUCGGCAAGUUGU
CCUUGGCUACACGU

PremiRNA secondary structure:(((((.(((((((((..((((((((((((((((..((((...............))))..)))))........((((((((((((((((((((((((.....)))))))))))...(((........))).........................))))))))))))).....))))))))))..))))))))).)))))

Degradome supported cutsite(s) in premiRNA:199
miRNA sequence and Count(AGO1/wt):CAGCCAAGGAUGACUUGCCGA_16.27/54.18
miRNA Position in PremiRNA:6-26
miRNA* sequence and Count(AGO1/wt):CAAGUUGUCCUUGGCUACACG_0/0.6
miRNA Position in PremiRNA:178-198
miRNA annonation:ath-miR169a

62. Degradome supported cutsite(s) in Genome:6244694
PremiRNA Locus in Genome:>Chr3(6244698-6244524)_minus strand
PremiRNA sequence:GGUUGUUGACAGAAGAUAGAGAGCACUAAGGAUGACAUGCAAGUACAUACAUAUAUAUCAUCACACCGCAUGUGGAUGAUAAAAUAUGUAUAACAAAUUCAAAGAAAGAGAGGGAGAGAAAGAGAGAGAACCUGCAUCUCUACUCUUUUGUGCUCUCUAUACUUCUGUCACCACC

PremiRNA secondary structure:(((.((.((((((((((((((((((...((((.((......))..((((((((.(((((((.(((.....))))))))))..))))))))).....))))...(((((((..(((((...((.(......)))...))))).))))))))))))))))))).)))))))))).)))

Degradome supported cutsite(s) in premiRNA:6
miRNA sequence and Count(AGO1/wt):UUGACAGAAGAUAGAGAGCAC_167.23/89.02
miRNA Position in PremiRNA:6-26
miRNA* sequence and Count(AGO1/wt):GCUCUCUAUACUUCUGUCACC_0.25/23.16
miRNA Position in PremiRNA:152-172
miRNA annonation:ath-miR157b/ath-miR157a/ath-miR157c

63. Degradome supported cutsite(s) in Genome:19659579
PremiRNA Locus in Genome:>Chr3(19659671-19659576)_minus strand
PremiRNA sequence:UGUCGUCUCGGUUCGCGAUCCACAAGUAAUCUUUUGUGGAGAUUAUGAAAACAAUCAUCACGAGAGGCUACGUGUGGGUGGCAAACAAAGACGACA
PremiRNA secondary structure:((((((((..(((.((.(((((((.(((..((((((((..((((.(....).))))..)))))))).))).))))))).)).)))..))))))))
Degradome supported cutsite(s) in premiRNA:94
miRNA sequence and Count(AGO1/wt):UCUCGGUUCGCGAUCCACAAG_166.23/20.14
miRNA Position in PremiRNA:6-26

miRNA* sequence and Count(AGO1/wt):UGUGGGUGGCAAACAAAGACG_0.75/0.6
miRNA Position in PremiRNA:73-93
miRNA annonation:ath-miR851-5p

64. Degradome supported cutsite(s) in Genome:369856/369877
PremiRNA Locus in Genome:>Chr4(369996-369851)_minus strand
PremiRNA
sequence:GUUGUGGGGAAUGUUGUUUGGAUCGAGGAUAUCAUAAACGCAUACACAUGUUUAUAUGUUAUGAUGCAUUAUAUGACUGAUGUAAUGUAC
AUAUAUAUACAUACAUGCCACAUGGUAUCGUCGGACCAGGCUUCAUCCCCCUCAAC
PremiRNA secondary structure:((((.((((.(((..((((((.((((.((((((((....((((....((((..((((((.(((.((((((((((.....))))))))))))))))))))))))..))))...)))))))).)))).))))))..))).)))).))))

Degradome supported cutsite(s) in premiRNA:142/121
miRNA sequence and Count(AGO1/wt):UCGGACCAGGCUUCAUCCCCC_26140.03/750.87
miRNA Position in PremiRNA:121-141
miRNA* sequence and Count(AGO1/wt):GGAAUGUUGUUUGGAUCGAGG_0/18.13
miRNA Position in PremiRNA:8-28
miRNA annonation:ath-miR165b/ath-miR165a

65. Degradome supported cutsite(s) in Genome:1535440
PremiRNA Locus in Genome:>Chr4(1535652-1535435)_minus strand
PremiRNA
sequence:UAUACAAUACUACUUUUUCAUCCAUUAAUCCCCUUACAAUGUCGAGUAAACGAAGCAUCUGUCCCCUGGUAUUGUCUUCGAGCUUGGUGUG
UUUUUCUAGCCAGCCCCAAGUUCUCGAGUUGAUCAUUGUUUGUAUUCUGACACAUUAUUUGGGGACGAGAUGUUUUGUUGACUCGAUAUAAGAAGG
GGCUUUAUGGAAGAAAUUGUAGUAUUAUAUA
PremiRNA secondary
structure:((((.((((((((..((((.(((((.((.((((((...(((((((((.((((((((((((((((((..(((.((((...((((((((.(((((.....)))..)).))))))))..((((....((.....)).)))).))))...)))..)))))).))))))))))))).)))))))))...)))))).)).))))).))))..))))
)))).))))
Degradome supported cutsite(s) in premiRNA:214
miRNA sequence and Count(AGO1/wt):UAUGGAAGAAAUUGUAGUAUU_1983.77/666.07
miRNA Position in PremiRNA:193-213
miRNA* sequence and Count(AGO1/wt):UACUACUUUUUCAUCCAUUAA_0.25/0.4
miRNA Position in PremiRNA:8-28
miRNA annonation:ath-miR447a.2-3p

66. Degradome supported cutsite(s) in Genome:8504145/8504169
PremiRNA Locus in Genome:>Chr4(8504315-8504140)_minus strand
PremiRNA
sequence:UCAAGAUAUCAGAUAUUUCACGAAGAUAUCUGCAUAACAGCUACGAGGAAAUUGUGAUUUUAUCGUUUCACAAACAUCAACAGCUCCAUGG
AUCGUUAUGGACGGAUAAAUCACAAUCACUAUCCUUUUUCUAGCAGCUGUUGAGCAGGUUUCUUCGUGAAUAUCUGGCAUUUUGG

PremiRNA secondary structure:(((((((..((((((((.((((((((.((((((.((((((((.((((((.((((((((((.((((((.....))).......(.((((((((....)))))))).))))))))))))).....))))).......).)))))))).)))))).))))))))))))))))..)))))))

Degradome supported cutsite(s) in premiRNA:172/148
miRNA sequence and Count(AGO1/wt):UUCUUCGUGAAUAUCUGGCAU_2565.84/597.39
miRNA Position in PremiRNA:151-171
miRNA* sequence and Count(AGO1/wt):UAUCAGAUAUUUCACGAAGAUA_16.02/8.06
miRNA Position in PremiRNA:8-29
miRNA annonation:ath-miR780.2

67. Degradome supported cutsite(s) in Genome:15415511
PremiRNA Locus in Genome:>Chr4(15415511-15415427)_minus strand
PremiRNA sequence:CUGACAGAAGAGAGUGAGCACACAAAGGCACUUUGCAUGUUCGAUGCAUUUGCUUCUCUUGCGUGCUCACUGCUCUAUCUGUCAG

PremiRNA secondary structure:((((((((((((((((((((.((((((((...(((((.....)))))..)))))...))).))))))))).)))).))))))))
Degradome supported cutsite(s) in premiRNA:2
miRNA sequence and Count(AGO1/wt):UGACAGAAGAGAGUGAGCAC_199.78/108.36
miRNA Position in PremiRNA:2-21
miRNA* sequence and Count(AGO1/wt):GCUCACUGCUCUAUCUGUCAGA_0/11.28
miRNA Position in PremiRNA:65-86
miRNA annonation:ath-miR156e/ath-miR156f/ath-miR156d/ath-miR156a/ath-miR156c/ath-miR156b

68. Degradome supported cutsite(s) in Genome:1188211
PremiRNA Locus in Genome:>Chr5(1188304-1188206)_minus strand
PremiRNA
sequence:UGUAGGCGCAGCACCAUUAAGAUUCACAUGGAAAUUGAUAAAUACCCUAAAUUAGGGUUUUGAUAUGUAUAUGAGAAUCUUGAUGAUGCUG
CAUCAACA
PremiRNA secondary structure:(((.(..((((((.(((((((((((.((((...((...((((.((((((...))))))))))..))...)))).))))))))))).))))))..).)))
Degradome supported cutsite(s) in premiRNA:95

miRNA sequence and Count(AGO1/wt):AGAAUCUUGAUGAUGCUGCAU_5566.03/1154.91
miRNA Position in PremiRNA:74-94
miRNA* sequence and Count(AGO1/wt):GCAGCACCAUUAAGAUUCACA_0.25/15.91
miRNA Position in PremiRNA:8-28
miRNA annonation:ath-miR172a/ath-miR172b-3p

69. Degradome supported cutsite(s) in Genome:2634936/2634957
PremiRNA Locus in Genome:>Chr5(2635022-2634934)_minus strand
PremiRNA
sequence:CGCUGGAGGCAGCGGUUCAUCGAUCUCUUCCUGUGAACACAUUAAAAAUGUAAAAGCAUGAAUAGAUCGAUAAACCUCUGCAUCCAGCG

PremiRNA secondary structure:(((((((.((((.((((.((((((((.(((.(((....((((.....))))....))).))).)))))))).)))).)))).)))))))
Degradome supported cutsite(s) in premiRNA:88/67
miRNA sequence and Count(AGO1/wt):UCGAUAAACCUCUGCAUCCAG_1712.14/373.62
miRNA Position in PremiRNA:67-87
miRNA* sequence and Count(AGO1/wt):GGAGGCAGCGGUUCAUCGAUC_0/1.01
miRNA Position in PremiRNA:5-25
miRNA annonation:ath-miR162b/ath-miR162a

70. Degradome supported cutsite(s) in Genome:3456666/3456733
PremiRNA Locus in Genome:>Chr5(3456737-3456640)_minus strand
PremiRNA
sequence:GAAGUUGACAGAAGAGAGUGAGCACACAAAGGGGAAGUUGUAUAAAAGUUUUGUAUAUGGUUGCUUUUGCGUGCUCACUCUCUUUUUGUCA
UAACUUC
PremiRNA secondary structure:(((((((((((((((((((((((((.((((((.((...(((((((.....)))))))...)).)))))).)))))))))))).))))))))..)))))
Degradome supported cutsite(s) in premiRNA:73/6
miRNA sequence and Count(AGO1/wt):UGACAGAAGAGAGUGAGCAC_199.78/108.36
miRNA Position in PremiRNA:6-25
miRNA* sequence and Count(AGO1/wt):GCUCACUCUCUUUUUGUCAUA_3.5/31.82
miRNA Position in PremiRNA:73-93
miRNA annonation:ath-miR156e/ath-miR156f/ath-miR156d/ath-miR156a/ath-miR156c/ath-miR156b

71. Degradome supported cutsite(s) in Genome:7740610/7740631
PremiRNA Locus in Genome:>Chr5(7740705-7740605)_minus strand

PremiRNA
sequence:UGAAGUCGCUGGAGGCAGCGGUUCAUCGAUCAAUUCCUGUGAAUAUUUAUUUUUGUUUACAAAAGCAAGAAUCGAUCGAUAAACCUCUGCA
UCCAGCGCUG
PremiRNA secondary structure:(..((.(((((((.((((.((((.(((((((.((((.((((((((........))))))))).......)))).))))))).)))).)))).))))))))))
Degradome supported cutsite(s) in premiRNA:97/76
miRNA sequence and Count(AGO1/wt):UCGAUAAACCUCUGCAUCCAG_1712.14/373.62
miRNA Position in PremiRNA:76-96
miRNA* sequence and Count(AGO1/wt):GGAGGCAGCGGUUCAUCGAUC_0/1.01
miRNA Position in PremiRNA:11-31
miRNA annonation:ath-miR162b/ath-miR162a

72. Degradome supported cutsite(s) in Genome:16660490
PremiRNA Locus in Genome:>Chr5(16660998-16660464)_minus strand
PremiRNA
sequence:AAAAGCCAUAACUCCUUCAUUUUCUUUAGACAUCUCUUCUUCUCUCAUCUCUCUUUUCUUCUCUCUUUUCCUCACAUAAACUCUCUUUUUUU
ACUAUUAAAUCCAUAUGGUACCUCAAAUUAAUCUAUGGUCAUCUAGGGUUAUCUUGAAGAUUAGAAUUGAUUCUAGCACGCACAGAGAGGAAGAUC
AUUGCAUCCAGAAUCACAAACAUGGCCUAUCUUUUAUCUUUUCUUUUUGAUCUAAGUCACUGUUUUAUGCUAUAUAUAGUAUAAUCAAAUUCUUUA
CAUGUGCUUGUAUGUAUGCGUAUAUAUAGUAACGGAAUUGUUAAUAUGCUUAUAGAUGUUGAGUUGGUGGAGGAAGAGAGCUUUCUUCGGUCCACU
CAUGGAGUAAUAUGUGAGAUUUAAUUGACUCUCGACUCAUUCAUCCAAAUACCAAAUGAAAGAAUUUGUUCUCAUAUGGUAAAUGAAUGAAUGAUG
CGAGAGACAAAUUGAGUCUUCACUUCUCUAUGCUUGGACUGAAGGGAGCUCCCUAUUUU
PremiRNA secondary
structure:((((........(((((((((..(((..((((((((((((((((......((..((((..((..........................((((((.........))))))..((((..((((((.(((....)))))))))..)))).))..))))..)).....(....)..))))))))))..(((.((((((........(((((((((..(((..
...............)))....)))).))))))..(((((((((((((((...........((((......))))..))))).)))))))))))..)))..)))....))).....))))))))))..)))))))))...(((((..((((((((((..(((((((......((((((((((((..((((((..((((((((.((..(((((.((((..(((....)
)))))).)))))..)).))))))))..))))))...))))))))).))))..)))))))..))))))))))..)))))....))))
Degradome supported cutsite(s) in premiRNA:510
miRNA sequence and Count(AGO1/wt):UUGGACUGAAGGGAGCUCCCU_20449.58/4223.04
miRNA Position in PremiRNA:510-530
miRNA* sequence and Count(AGO1/wt):AGAGCUUUCUUCGGUCCACUC_48.82/53.58
miRNA Position in PremiRNA:361-381
miRNA annonation:ath-miR319b/ath-miR319a

73. Degradome supported cutsite(s) in Genome:16775545
PremiRNA Locus in Genome:>Chr5(16775662-16775520)_minus strand

PremiRNA
sequence:UUGAGGGGAAUGUUGUCUGGCACGAGGCCCUUAACUUAGAUCUAUAUUUGAUUAUAUAUAUAUGUCUCUUCUUUAUUCAUUAGUCUAUACA
UGAAUGAUCAUUUUACGGUUAAUGACGUCGGACCAGGCUUCAUUCCCCUCAA
PremiRNA secondary structure:((((((((((((..((((((..(((.(.(.((((((.((((.(((((..........))))).)))).....((((((((((.....)).))))))))).........)))))).).).)))..))))))..))))))))))))
Degradome supported cutsite(s) in premiRNA:119
miRNA sequence and Count(AGO1/wt):UCGGACCAGGCUUCAUUCCCC_76756.02/3261.89
miRNA Position in PremiRNA:119-139
miRNA* sequence and Count(AGO1/wt):GGAAUGUUGUCUGGCACGAGG_0/5.84
miRNA Position in PremiRNA:7-27
miRNA annonation:ath-miR166b/ath-miR166f/ath-miR166a/ath-miR166g/ath-miR166c/ath-miR166d/ath-miR166e

74. Degradome supported cutsite(s) in Genome:18358894
PremiRNA Locus in Genome:>SChr5(18358898-18358800)_minus strand
PremiRNA
sequence:CGGAUUCGCUUGGUGCAGGUCGGGAACUGAUUGGCUGACACCGACACGUGUCUUGUCAUGGUUGGUUUGUGAGCUCCCGUCUUGUAUCAAC
UGAAUCGG
PremiRNA secondary structure:(.((((((.((((((((((.(((((........(((.((((((((.((((......))))))))))..)).)))))))).)))))))))).)))))).)
Degradome supported cutsite(s) in premiRNA:6
miRNA sequence and Count(AGO1/wt):UCGCUUGGUGCAGGUCGGGAA_1012.92/266.47
miRNA Position in PremiRNA:6-26
miRNA* sequence and Count(AGO1/wt):CCCGUCUUGUAUCAACUGAAU_0.25/15.91
miRNA Position in PremiRNA:76-96
miRNA annonation:ath-miR168b/ath-miR168a

75. Degradome supported cutsite(s) in Genome:771384/771406
PremiRNA Locus in Genome:>Chr2(771517-771382)_minus strand
PremiRNA
sequence:GAAGACACUGAAGGACCUAAACUAACAAAGGUAAACGGCUCAGUGUGCGGGGUAUUACACUCGGUUUAAUGUCUGAAUGCGAUAAUCCGCA
CGAUGAUCUCUUUAUCUUUGUUUGUUUAGGUCCCUUAGUUUCUUC
PremiRNA secondary structure:(((((.(((((.((((((((((.(((((((((((.((.(((.(((((((..(((..((.((((........)))).))..)))..))))))).))).)).))))))))))).)))))))))).))))).)))))
Degradome supported cutsite(s) in premiRNA:135/113
miRNA sequence and Count(AGO1/wt):CACUGAAGGACCUAAACUAAC_9.26/3.22
miRNA Position in PremiRNA:6-26
miRNA* sequence and Count(AGO1/wt):UUGUUUAGGUCCCUUAGUUUC_34.05/9.47

miRNA Position in PremiRNA:113-133
miRNA annonation:ath-miR840-3p

76. Degradome supported cutsite(s) in Genome:19659579
PremiRNA Locus in Genome:>Chr3(19659673-19659574)_minus strand
PremiRNA sequence:ACUGUCGUCUCGGUUCGCGAUCCACAAGUAAUCUUUUGUGGAGAUUAUGAAAACAAUCAUCACGAGAGGCUACGUGUGGGUGGCAAACAAAGACGACAAU
PremiRNA secondary structure:(.((((((((..(((.((.(((((((.(((..((((((((..((((.(....).))))..))))))))..))).))))))).)).)))..)))))))).)
Degradome supported cutsite(s) in premiRNA:96
miRNA sequence and Count(AGO1/wt):UGUGGGUGGCAAACAAAGACG_0.75/0.6
miRNA Position in PremiRNA:75-95
miRNA* sequence and Count(AGO1/wt):UCUCGGUUCGCGAUCCACAAG_166.23/20.14
miRNA Position in PremiRNA:8-28
miRNA annonation:ath-miR851-5p

Supplementary table S2b. Discovery of known miRNAs from leaves of *Arabidopsis*.

1. Degradome supported cutsite(s) in Genome:28656
PremiRNA Locus in Genome:>Chr1(28545-28660)_plus strand
PremiRNA
sequence:UGGUGUUGUGCAAGAAGGAGAAGCAAAGUCUGUCUAUGUAUUAUGAGAUAGCUACUUCUAUGGCUAGGAUAUAUGUUGUACAAGACCGGCU
UUUCUUCUACUUCUUGCACAACCUG
PremiRNA secondary structure:(((.(((((((((((((.(((((.((((((.((((.(((((((((...((((((......))))))...))))....))))))))).)))))).))))).)))))))))))))))
Degradome supported cutsite(s) in premiRNA:112
miRNA sequence and Count(AGO1/wt):UUUUCUUCUACUUCUUGCACA_15.79/12.6
miRNA Position in PremiRNA:91-111
miRNA* sequence and Count(AGO1/wt):UGCAAGAAGGAGAAGCAAAGU_0.36/0.8
miRNA Position in PremiRNA:9-29
miRNA annonation:ath-miR838

2. Degradome supported cutsite(s) in Genome:2165570
PremiRNA Locus in Genome:>Chr1(2165563-2165700)_plus strand
PremiRNA
sequence:UCUUACAUCUUGAUGAAGAGGAAUGGGAAGCCGAAAUCUAAACUGAAACAGAGGCGUCACCCAAUUAUCAAUUGGUAAGUGAUAUCUAUGU
UUCGAUCUGUAUCUCGGCCUUUCACUCCUCUUCUUCUUGAUGUAAGA
PremiRNA secondary structure:(((((((((..((.((((((((.((..(.(((((.........(((((((.((..((((((((((.....)))))...)))))..)).))))))).........))))).)..)).)))))))).))..)))))))))
Degradome supported cutsite(s) in premiRNA:8
miRNA sequence and Count(AGO1/wt):UCACUCCUCUUCUUCUUGAUG_63.5/19.2
miRNA Position in PremiRNA:113-133
miRNA* sequence and Count(AGO1/wt):UCUUGAUGAAGAGGAAUGGGA_6.82/3.2
miRNA Position in PremiRNA:8-28
miRNA annonation:ath-miR847

3. Degradome supported cutsite(s) in Genome:6220813/6220834
PremiRNA Locus in Genome:>Chr1(6220645-6220838)_plus strand
PremiRNA
sequence:AGGAAGAGCUCCUUGAAGUUCAAUGGAGGGUUUAGCAGGGUGAAGUAAAGCUGCUAAGCUAUGGAUCCCAUAAGCCUUAUCAAAUUCAAUA
UAAUUGAUGAUAAGGUUUUUUUUAUGGAUGCCAUAUCUCAGGAGCUUUCACUUACCCCUUUAAUGGCUUCACUCUUCUUUGGAUUGAAGGGAGCUC
UUCAUCU

PremiRNA secondary structure:(((((((((((((..(((((((.(((((((..(((((((..((((((((((.((.((.(((((...((((((((((((((...(((((...))))))))))))))))).....))))))...))))).)).)).))))))).))))..))))......)))..))))))).)))))))..))))))))))))..))

Degradome supported cutsite(s) in premiRNA:169/190
miRNA sequence and Count(AGO1/wt):UUUGGAUUGAAGGGAGCUCUU_110106.13/5624.98
miRNA Position in PremiRNA:169-189
miRNA* sequence and Count(AGO1/wt):GAGCUCCUUGAAGUUCAAUGG_22.24/157.84
miRNA Position in PremiRNA:6-26
miRNA annonation:ath-miR159b

4. Degradome supported cutsite(s) in Genome:6740590
PremiRNA Locus in Genome:>Chr1(6740498-6740593)_plus strand
PremiRNA sequence:UUGCUUCAGGUAUGAUUGACUUCAAAAAAUACCUUGAAACUAUAAACCUCAGUUUCUUUGAAUUUGAUUUUUAAAGUCAAUAAUACCUUGAAGCAA
PremiRNA secondary structure:((((((((((((.((((((((.(((((.......((((((.........))))))...........))))).)))))))).))))).))))))))
Degradome supported cutsite(s) in premiRNA:93
miRNA sequence and Count(AGO1/wt):UCAGGUAUGAUUGACUUCAAA_47/16.8
miRNA Position in PremiRNA:6-26
miRNA* sequence and Count(AGO1/wt):UAAAGUCAAUAAUACCUUGAAG_1.79/1.2
miRNA Position in PremiRNA:72-93
miRNA annonation:ath-miR864-5p

5. Degradome supported cutsite(s) in Genome:9364561
PremiRNA Locus in Genome:>Chr1(9364477-9364564)_plus strand
PremiRNA sequence:CCAUGAGUUCCCUUUAACGCUUCAUUGUUAAAUACUCAAAGCCACAUUGGUUUGUAUACAACACUGAAGUGUUUGGGGGACUCUUGG

PremiRNA secondary structure:(((.(((((((((..(((((((((.((((..((((...(((((.....)))))))))..)))).)))))))))..))))))))).)))
Degradome supported cutsite(s) in premiRNA:85
miRNA sequence and Count(AGO1/wt):CUGAAGUGUUUGGGGGACUC_357.69/20
miRNA Position in PremiRNA:64-84
miRNA* sequence and Count(AGO1/wt):GUUCCCUUUAACGCUUCAUUG_0/0.6
miRNA Position in PremiRNA:7-27

miRNA annonation:ath-miR395f/ath-miR395c/ath-miR395b

6. Degradome supported cutsite(s) in Genome:17825722
PremiRNA Locus in Genome:>Chr1(17825720-17825829)_plus strand
PremiRNA
sequence:GAUCAAUGCAUUGAAAGUGACUACAUCGGGGUUCCGAUUUUUUUUGUUCUUCAUAUGAUGAAGCGGAAACAGUAAUCAACCCUGGUUUAGUCACUUUCACUGCAUUAAUC
PremiRNA secondary structure:(((.((((((.((((((((((((.(((((((((..((((....((((((((((((...))))))....))))).)))))))))))))).)))))))))))).)))))).)))
Degradome supported cutsite(s) in premiRNA:3
miRNA sequence and Count(AGO1/wt):UCAAUGCAUUGAAAGUGACUA_2939/2140.73
miRNA Position in PremiRNA:3-23
miRNA* sequence and Count(AGO1/wt):GUCACUUUCACUGCAUUAAUC_5.74/10.4
miRNA Position in PremiRNA:90-110
miRNA annonation:ath-miR161.2

7. Degradome supported cutsite(s) in Genome:20041586
PremiRNA Locus in Genome:>Chr1(20041581-20041665)_plus strand
PremiRNA
sequence:GAGAUUGAGCCAAGGAUGACUUGCCGAUUUUCUCAACGAAUCUUACUGAUUAUGGUAUCCGGCAAGUUGACUUUGGCUCUGUUUC

PremiRNA secondary structure:(((((.(((((((((.((((((((((...(((.....)))...((((......))))..)))))))))).))))))))).)))))
Degradome supported cutsite(s) in premiRNA:6
miRNA sequence and Count(AGO1/wt):UGAGCCAAGGAUGACUUGCCG_31.21/9.2
miRNA Position in PremiRNA:6-26
miRNA* sequence and Count(AGO1/wt):GCAAGUUGACUUUGGCUCUGU_0/8
miRNA Position in PremiRNA:62-82
miRNA annonation:ath-miR169g-5p/ath-miR169d/ath-miR169e/ath-miR169f

8. Degradome supported cutsite(s) in Genome:22149808/22149735
PremiRNA Locus in Genome:>Chr1(22149730-22149831)_plus strand
PremiRNA
sequence:CGAAAUCUCUCUGUUGUGAAGUCAAACAUGAGUAUGAAUUAACAUUAAUGGAUCUUUUUGAUUCAUAAUACUCAUGUUUGGUUUCACAGUAGAUAGAUAUCG
PremiRNA secondary structure:(((.((((.(((((((((((..(((((((((((((............(((((((.....))))))).)))))))))))))..))))))))))).)))).)))

Degradome supported cutsite(s) in premiRNA:79/6
miRNA sequence and Count(AGO1/wt):UCUCUCUGUUGUGAAGUCAAA_1.44/1.4
miRNA Position in PremiRNA:6-26
miRNA* sequence and Count(AGO1/wt):UGGUUUCACAGUAGAUAGAUA_0/0
miRNA Position in PremiRNA:79-99
miRNA annonation:ath-miR859

9. Degradome supported cutsite(s) in Genome:24554011
PremiRNA Locus in Genome:>Chr1(24553982-24554031)_plus strand
PremiRNA sequence:CGUACACUUACAUCUGUACACUAAAAGUGUUGUACAAAUUUAAGUGUACG
PremiRNA secondary structure:((((((((((.((.(((((((.......).)))))).)).))))))))))
Degradome supported cutsite(s) in premiRNA:30
miRNA sequence and Count(AGO1/wt):UUGUACAAAUUUAAGUGUACG_17.22/4.4
miRNA Position in PremiRNA:30-50
miRNA* sequence and Count(AGO1/wt):UACACUUACAUCUGUACACUA_0/0
miRNA Position in PremiRNA:3-23
miRNA annonation:ath-miR5014a-3p

10. Degradome supported cutsite(s) in Genome:24884359
PremiRNA Locus in Genome:>Chr1(24884354-24884581)_plus strand
PremiRNA
sequence:AGUCGUUGAAGAGGACUUGGAACUUCGAUAUUAUCCCCCGUGUUUUGUCCAGUUUCUCUAUAGGUUGUGCCUGAAAAUAUGGGUUUCUAGG
UUUAAUUGAUGAAAAGAGAUAACGUAAGUCAUGCACAUGUAAUUAAAUUCAUGCCCCUAACUAUUAAUUCUUGCAAGUAAUUAAAAAAUAAAACGU
ACACAAAAUAUAUUUAUGAUCACUAAUCCUUUUUAACUACU
PremiRNA secondary
structure:(((.(((((((((((.((((......(((...)))....(((((((((...((((.....((((.(((((.(((...((((.((((((...((((.....)))).))))))..))))..))).))))).(((..........))).))))....((((((((....)).))))))......))))....)))))))))..........))))))))
))))))).)))
Degradome supported cutsite(s) in premiRNA:6
miRNA sequence and Count(AGO1/wt):UUGAAGAGGACUUGGAACUUCGAU_50470.38/8029.77
miRNA Position in PremiRNA:6-29
miRNA* sequence and Count(AGO1/wt):UAUCCCCCGUGUUUUGUCC_0.36/0.6
miRNA Position in PremiRNA:35-225
miRNA annonation:ath-miR163

11. Degradome supported cutsite(s) in Genome:24921104
PremiRNA Locus in Genome:>Chr1(24921099-24921200)_plus strand
PremiRNA sequence:UAGUGUUGACAGAAGAUAGAGAGCACAGAUGAUAAGAUACAAUUCCUCGCAGCUUCUUUGCAUCUUACUCCUUUGUGCUCUCUAGCCUUCU GUCAUCACCCG
PremiRNA secondary structure:(.(((.(((((((((.(((((((((((((.((((((((.(((...............))).)))))).)).))))))))))))..))))))))).)))..)
Degradome supported cutsite(s) in premiRNA:6
miRNA sequence and Count(AGO1/wt):UUGACAGAAGAUAGAGAGCAC_1637.4/715.58
miRNA Position in PremiRNA:6-26
miRNA* sequence and Count(AGO1/wt):GCUCUCUAGCCUUCUGUCAUCA_0/0.8
miRNA Position in PremiRNA:77-98
miRNA annonation:ath-miR157b/ath-miR157a/ath-miR157c

12. Degradome supported cutsite(s) in Genome:26638083
PremiRNA Locus in Genome:>Chr1(26638000-26638107)_plus strand
PremiRNA sequence:AGAGAGCAACAAAAUCAGUGUGUAUUUGUUAAGACAAAUGUUUAUAUAAGUCGUCCAUGCGUUGUACAUUUAAUCUUAACUAAUACGCAUU GAGUUUCGUUGCUUUCU
PremiRNA secondary structure:((((((((((((.((((((((((((.(((((((.((((((....((((..(((....)))))))))))))..))))))).))))))))))))).))).))))))))))
Degradome supported cutsite(s) in premiRNA:84
miRNA sequence and Count(AGO1/wt):UACGCAUUGAGUUUCGUUGCUU_45.56/18.8
miRNA Position in PremiRNA:84-105
miRNA* sequence and Count(AGO1/wt):CAACAAAAUCAGUGUGUAUU_0/0
miRNA Position in PremiRNA:6-26
miRNA annonation:ath-miR777

13. Degradome supported cutsite(s) in Genome:29422546
PremiRNA Locus in Genome:>Chr1(29422456-29422570)_plus strand
PremiRNA sequence:AACGUUGCACUACGUGACAUUGAAACUGUCUUUCAACAUUCCAAUAUUUCAACUUUCGAAUACCCAAUAUUUGGUUUGUUCAAAGACAUUU UCGAUGUCUAGCAGUGCCAAUGUU
PremiRNA secondary structure:(((((((((((....((((((((((.(((((((.((((..((((((((.................)))).))))..)))).))))))).))))))))))....)))).)))))))
Degradome supported cutsite(s) in premiRNA:91
miRNA sequence and Count(AGO1/wt):UUCGAUGUCUAGCAGUGCCA_334.37/124.83

miRNA Position in PremiRNA:91-110
miRNA* sequence and Count(AGO1/wt):GCACUACGUGACAUUGAAAC_1.08/29.01
miRNA Position in PremiRNA:7-26
miRNA annonation:ath-miR775

14. Degradome supported cutsite(s) in Genome:22150026
PremiRNA Locus in Genome:>Chr1(22149939-22150030)_plus strand
PremiRNA
sequence:UUUCAGAUGGCUGUUUGGGUAACUAAUAUUUAAGAUUUUGGUCAAUUUAAGUUACCAAUUUAUAUAUUGGUUACCCAUAUGGCCAUCUCAA
A
PremiRNA secondary structure:(((.((((((((((.((((((((((((((.((((...(((((.(((....)))))))))))).)))))))))))))).)))))))))).)))
Degradome supported cutsite(s) in premiRNA:88
miRNA sequence and Count(AGO1/wt):UUGGUUACCCAUAUGGCCAUC_0.72/0.2
miRNA Position in PremiRNA:67-87
miRNA* sequence and Count(AGO1/wt):UGGCUGUUUGGGUAACUAAUA_0/0
miRNA Position in PremiRNA:8-28
miRNA annonation:ath-miR774a

15. Degradome supported cutsite(s) in Genome:1041012
PremiRNA Locus in Genome:>Chr2(1040937-1041037)_plus strand
PremiRNA
sequence:GUGAAAUUUCAAAGGAGUGGCAUGUGAACACAUAUCCUAUGGUUUCUUCAAAUUUCCAUUGAAACCAUUGAGUUUUGUGUUCUCAGGUCAC
CCCUUUGAAU
PremiRNA secondary structure:((......(((((((.(((((.((.((((((((..((..(((((((((.............)))))))).))....))))))).)).))))).)))))))))
Degradome supported cutsite(s) in premiRNA:76
miRNA sequence and Count(AGO1/wt):UGUGUUCUCAGGUCACCCCUU_18.3/0.4
miRNA Position in PremiRNA:76-96
miRNA* sequence and Count(AGO1/wt):GGAGUGGCAUGUGAACACAUA_0.36/2.6
miRNA Position in PremiRNA:14-34
miRNA annonation:ath-miR398a

16. Degradome supported cutsite(s) in Genome:9560866/9560887
PremiRNA Locus in Genome:>Chr2(9560769-9560891)_plus strand

PremiRNA
sequence:UAAUAUAGUUGAAUCUUCGAAAGUAUUUCAAGAAGUCAGCUGAGCUUUCUCGUCAUCACUUAUUAGUAAAUAUAGUCUCUAUAUUUUGAUGAGUGAUGAUUGGAAAUUUCGUUGACUCAUUA
PremiRNA secondary structure:((((....(((((....(....)...)))))..(((((((.(((.(((((.((((((((((((((((.((((((((...))))))))))))))))))))))))).))))).))))))))))).))))
Degradome supported cutsite(s) in premiRNA:98/119
miRNA sequence and Count(AGO1/wt):UGAUUGGAAAUUUCGUUGACU_25.47/8.6
miRNA Position in PremiRNA:98-118
miRNA* sequence and Count(AGO1/wt):UCAGCUGAGCUUUCUCGUCAUC_67.09/15
miRNA Position in PremiRNA:36-57
miRNA annonation:ath-miR779.2

17. Degradome supported cutsite(s) in Genome:11159774
PremiRNA Locus in Genome:>Chr2(11159706-111159799)_plus strand
PremiRNA
sequence:GACUCGUUCAAGCACCAGCUCGAAGAAGCUUAGCUAAUUUAUCUUAGAAAAUAAUGAAAAAGCUAUGCUUCUCAAGAAGGUGCAUGAACAAGUU
PremiRNA secondary structure:((((.(((((.(((((..((.((.(((((.(((((..(((((..(.....)..)))))..))))).))))))).))..))))).))))).))))
Degradome supported cutsite(s) in premiRNA:69
miRNA sequence and Count(AGO1/wt):UUCUCAAGAAGGUGCAUGAAC_237.86/79.42
miRNA Position in PremiRNA:69-89
miRNA* sequence and Count(AGO1/wt):UCAAGCACCAGCUCGAAGAAGC_19.73/13.8
miRNA Position in PremiRNA:8-29
miRNA annonation:ath-miR825

18. Degradome supported cutsite(s) in Genome:15611882
PremiRNA Locus in Genome:>Chr2(15611875-15611978)_plus strand
PremiRNA
sequence:AGAGAAGUGAGAUGAAAUCUUUGAUUGGAAAUUUCAUGUAGACGAUGCUAUCACCCCAUAUGACCAAAAGAAAUUCCCAAUUAAAGAUUUCAUCUUACUUCCCU
PremiRNA secondary structure:((.(((((((((((((((((((((((((.((((((..(((.....)))..(((.......)))......)))))).))))))))))))))))))))))))).))
Degradome supported cutsite(s) in premiRNA:8
miRNA sequence and Count(AGO1/wt):AAUUAAAGAUUUCAUCUUACU_0/4.8
miRNA Position in PremiRNA:79-99
miRNA* sequence and Count(AGO1/wt):UGAGAUGAAAUCUUUGAUUGG_54.53/17.8

miRNA Position in PremiRNA:8-28
miRNA annonation:ath-miR1886.3

19. Degradome supported cutsite(s) in Genome:16061971
PremiRNA Locus in Genome:>Chr2(16061966-16062046)_plus strand
PremiRNA sequence:CUGUAAAGCUCAGGAGGGAUAGCGCCAUGAUGAUCACAUUCGUUAUCUAUUUUUGGCGCUAUCCAUCCUGAGUUUCAUUG

PremiRNA secondary structure:(.((.((((((((((.(((((((((((.((((((.((....)).))).)))...))))))))))).)))))))))).)).)
Degradome supported cutsite(s) in premiRNA:6
miRNA sequence and Count(AGO1/wt):AAGCUCAGGAGGGAUAGCGCC_87.9/375.29
miRNA Position in PremiRNA:6-26
miRNA* sequence and Count(AGO1/wt):CGCUAUCCAUCCUGAGUUUCA_8.97/208.65
miRNA Position in PremiRNA:58-78
miRNA annonation:ath-miR390b/ath-miR390a

20. Degradome supported cutsite(s) in Genome:16340282/16340363
PremiRNA Locus in Genome:>Chr2(16340279-16340363)_plus strand
PremiRNA sequence:GUAUGCCUGGCUCCCUGUAUGCCAUAUGCUGAGCCCAUCGAGUAUCGAUGACCUCCGUGGAUGGCGUAUGAGGAGCCAUGCAUAU

PremiRNA secondary structure:((((((.(((((((..(((((((((.(((.(((..(((((.....)))))..))).))).)))))))))..))))))).))))))
Degradome supported cutsite(s) in premiRNA:4/85
miRNA sequence and Count(AGO1/wt):UGCCUGGCUCCCUGUAUGCCA_2840.7/218.45
miRNA Position in PremiRNA:4-24
miRNA* sequence and Count(AGO1/wt):GCGUAUGAGGAGCCAUGCAUA_0/14.6
miRNA Position in PremiRNA:64-84
miRNA annonation:ath-miR160a/ath-miR160c/ath-miR160b

21. Degradome supported cutsite(s) in Genome:19176240
PremiRNA Locus in Genome:>Chr2(19176122-19176265)_plus strand
PremiRNA
sequence:UUGAGGGGACUGUUGUCUGGCUCGAGGACUCUGGCUCGCUCUAUUCAUGUUGGAUCUCUUUCGAUCUAACAAUCGAAUUGAACCUUCAGAU
UUCAGAUUUGAUUAGGGUUUUAGCGUCUUCGGACCAGGCUUCAUUCCCCCCAA
PremiRNA secondary structure:(((.(((((.((..((((((..(((((((.((((...((((((.((((((((((((......)))))))))...(((((((....))))..))).....))).)))))).)))).)))))))..))))))..)).))))).)))
Degradome supported cutsite(s) in premiRNA:119

miRNA sequence and Count(AGO1/wt):UCGGACCAGGCUUCAUUCCCC_56156.81/2154.13
miRNA Position in PremiRNA:120-140
miRNA* sequence and Count(AGO1/wt):GGACUGUUGUCUGGCUCGAGG_0/85.62
miRNA Position in PremiRNA:7-27
miRNA annonation:ath-miR166b/ath-miR166f/ath-miR166a/ath-miR166g/ath-miR166c/ath-miR166d/ath-miR166e

22. Degradome supported cutsite(s) in Genome:19520770
PremiRNA Locus in Genome:>Chr2(19520766-19520851)_plus strand
PremiRNA
sequence:AUGUUGGAGAAGCAGGGCACGUGCAAACCAACAAACACGAAAUCCGUCUCAUUUGCUUAUUUGCACGUACUUAACUUCUCCAACAU

PremiRNA secondary structure:((((((((((((..(((.(((((((((....((((.(((.....)))....))))....))))))))).)))..))))))))))))
Degradome supported cutsite(s) in premiRNA:5
miRNA sequence and Count(AGO1/wt):UGGAGAAGCAGGGCACGUGCA_798.61/107.83
miRNA Position in PremiRNA:5-25
miRNA* sequence and Count(AGO1/wt):CACGUACUUAACUUCUCCAAC_1.44/1
miRNA Position in PremiRNA:64-84
miRNA annonation:ath-miR164b/ath-miR164a

23. Degradome supported cutsite(s) in Genome:6217561
PremiRNA Locus in Genome:>Chr3(6217490-6217584)_plus strand
PremiRNA
sequence:CAAGUAUGGUUUUCUUGGCCCAUCCACUUCUAAUGUGUUUUGUAACUAAUGCAAAAUGUGCAUUAGAAGUGGAUUGGUCAAGGGAAGCGUA
CUUG
PremiRNA secondary structure:((((((((.((..(((((((.(((((((((((((((((((((((.....))))))))..))))))))))))))).)))))))..)).))))))))
Degradome supported cutsite(s) in premiRNA:72
miRNA sequence and Count(AGO1/wt):UGGAUUGGUCAAGGGAAGCGU_0/0.2
miRNA Position in PremiRNA:70-90
miRNA* sequence and Count(AGO1/wt):UUUUCUUGGCCCAUCCACUUC_1.79/0.6
miRNA Position in PremiRNA:8-28
miRNA annonation:ath-miR3440b-3p

24. Degradome supported cutsite(s) in Genome:8108090/8108172/8108193
PremiRNA Locus in Genome:>Chr3(8108090-8108190)_plus strand

PremiRNA
sequence:UGAAGCUGCCAGCAUGAUCUAAUUAGCUUUCUUUAUCCUUUGUUGUGUUUCAUGACGAUGGUUAAGAGAUCAGUCUCGAUUAGAUCAUGUU
CGCAGUUUCA
PremiRNA secondary structure:(((((((((.((((((((((((((((((.(((((..((.((((..((...))..)))).))...)))))..)).)).))))))))))))))).)))))))))
Degradome supported cutsite(s) in premiRNA:1/83/104
miRNA sequence and Count(AGO1/wt):UGAAGCUGCCAGCAUGAUCUA_6224.22/3575.48
miRNA Position in PremiRNA:1-21
miRNA* sequence and Count(AGO1/wt):GAUCAUGUUCGCAGUUUCACC_0/1.2
miRNA Position in PremiRNA:83-103
miRNA annonation:ath-miR167b/ath-miR167a

25. Degradome supported cutsite(s) in Genome:8236161
PremiRNA Locus in Genome:>Chr3(8236156-8236251)_plus strand
PremiRNA
sequence:GUACUUUCGCUUGCAGAGAGAAAUCACAGUGGUCAAAAAAGUUGUAGUUUUCUUAAAGUCUCUUUCCUCUGUGAUUCUCUGUGUAAGCGAA
AGAGC
PremiRNA secondary structure:((.((((((((((((.((((((.((((((.((.........(((.((....))))).........)).)))))))))))).)))))))))))).))
Degradome supported cutsite(s) in premiRNA:6
miRNA sequence and Count(AGO1/wt):UUCGCUUGCAGAGAGAAAUCAC_4397.39/1264.11
miRNA Position in PremiRNA:6-27
miRNA* sequence and Count(AGO1/wt):GAUUCUCUGUGUAAGCGAAAG_3.23/41.61
miRNA Position in PremiRNA:73-93
miRNA annonation:ath-miR173-5p

26. Degradome supported cutsite(s) in Genome:8346589/8346610
PremiRNA Locus in Genome:>Chr3(8346411-8346612)_plus strand
PremiRNA
sequence:GCUGCUCCCCUCUUUAGCUUGGAGAAGCCAGUAAAUAUCUUUCUCUUCAAGGAACUUGUGGGCCUCAAGAAAACCUGCUAUAAUUCUAGUU
UUUCAACAUUCCUCCUUCUGAUAUCAGCAACAACUCCUUACCCUUAAGAAUGGAGAAUGGAACUUCCUCAAAGAUAUUUACUUGGCCUCUGCAACCG
GAAAGGGGAGCAGC
PremiRNA secondary
structure:((((((((((.((..(.(((.(((.((((((((((((((((.......(((..(....)..)))...(((((...((((......)))))))))..(((((.(((..(((....))).........((((....))))...))))))))..........))))))))))).)))).))).)))).)).))))))))))

Degradome supported cutsite(s) in premiRNA:179/200

miRNA sequence and Count(AGO1/wt):UCCCCUCUUUAGCUUGGAGAAG_22.6/7.2
miRNA Position in PremiRNA:6-27
miRNA* sequence and Count(AGO1/wt):UCUGCAACCGGAAAGGGGAGC_9.33/3.6
miRNA Position in PremiRNA:179-199
miRNA annonation:ath-miR853

27. Degradome supported cutsite(s) in Genome:19073542
PremiRNA Locus in Genome:>Chr3(19073446-19073545)_plus strand
PremiRNA
sequence:CUUUGAUAUUGGCCUGGUUCACUCAGAUCUUACCUGACCACACACGUAGAUAUACAUUAUUCUCUCUAGAUUAUCUGAUUGAGCCGCGCCA
AUAUCUCAG
PremiRNA secondary structure:((..(((((((((.(((((((.((((((.((((.((......)).))))...........(((....)))..)))))).))))))).)))))))))..))
Degradome supported cutsite(s) in premiRNA:97
miRNA sequence and Count(AGO1/wt):UGAUUGAGCCGCGCCAAUAUC_370.25/257.86
miRNA Position in PremiRNA:76-96
miRNA* sequence and Count(AGO1/wt):UAUUGGCCUGGUUCACUCAGA_336.16/56.01
miRNA Position in PremiRNA:7-27
miRNA annonation:ath-miR171a

28. Degradome supported cutsite(s) in Genome:20587991
PremiRNA Locus in Genome:>Chr3(20587914-20588016)_plus strand
PremiRNA
sequence:UGCUAUUGCAACAUCUUCAAGAUUCAGAAAUCAGAUUCUCUUAUGGGUUUUCUUUUGAGCCUUUAUUUUUGGUUUGAGAAUCUUGAUGAU
GCUGCAGCGGCA
PremiRNA secondary structure:(((..(((((.((((.(((((((((..((((((((......((.((((((......)))))).))...))))))))..))))))))).)))).)))))..)))
Degradome supported cutsite(s) in premiRNA:78
miRNA sequence and Count(AGO1/wt):AGAAUCUUGAUGAUGCUGCAG_42.33/6.8
miRNA Position in PremiRNA:78-98
miRNA* sequence and Count(AGO1/wt):GCAACAUCUUCAAGAUUCAGA_0/1.4
miRNA Position in PremiRNA:8-28
miRNA annonation:ath-miR172d/ath-miR172c

29. Degradome supported cutsite(s) in Genome:22922301/22922321
PremiRNA Locus in Genome:>Chr3(22922206-22922325)_plus strand

PremiRNA
sequence:UUGAGGGGACUGUUGUCUGGCUCGAGGACUCUUAUUCUAAUACAAUCUCAUUUGAAUACAUUCAGAUCUGAUGAUUGAUUAGGGUUUUAGU GUCGUCGGACCAGGCUUCAUUCCCCCCAA
PremiRNA secondary structure:(((.(((((.((..((((((..(((.(((.((.((((((((.(((((((((((((....))))))..))).)))))))))))))...)).))).))).))..))))))..)).))))).)))
Degradome supported cutsite(s) in premiRNA:96/116
miRNA sequence and Count(AGO1/wt):UCGGACCAGGCUUCAUUCCCC_56156.81/2154.13
miRNA Position in PremiRNA:96-116
miRNA* sequence and Count(AGO1/wt):GGACUGUUGUCUGGCUCGAGG_0/85.62
miRNA Position in PremiRNA:7-27
miRNA annonation:ath-miR166b/ath-miR166f/ath-miR166a/ath-miR166g/ath-miR166c/ath-miR166d/ath-miR166e

30. Degradome supported cutsite(s) in Genome:23406177
PremiRNA Locus in Genome:>Chr3(23406172-23406272)_plus strand
PremiRNA
sequence:ACAAGUGAAGCUGCCAGCAUGAUCUAUCUUUGGUUAAGAGAUGAAUGUGGAAACAUAUUGCUUAAACCCAAGCUAGGUCAUGCUCUGACAG CCUCACUCCU
PremiRNA secondary structure:(..(((((.(((((.(((((((((((.(((.((((..(((...((((((....)))))).))).)))).))).)))))))))))..).)))).)))))..)
Degradome supported cutsite(s) in premiRNA:6
miRNA sequence and Count(AGO1/wt):UGAAGCUGCCAGCAUGAUCUA_6224.22/3575.48
miRNA Position in PremiRNA:6-26
miRNA* sequence and Count(AGO1/wt):GGUCAUGCUCUGACAGCCUCACU_0/14
miRNA Position in PremiRNA:76-98
miRNA annonation:ath-miR167b/ath-miR167a

31. Degradome supported cutsite(s) in Genome:9889047
PremiRNA Locus in Genome:>Chr4(9888982-9889070)_plus strand
PremiRNA
sequence:GUCGUGCCUGGCUCCCUGUAUGCCACAAGAAAACAUCGAUUUAGUUUCAAAAUCGAUCACUAGUGGCGUACAGAGUAGUCAAGCAUGAC

PremiRNA secondary structure:(((((((.(((((.((((((((((((.((.....((((((((.......))))))))..)).)))))))))))).).))))).)))))))
Degradome supported cutsite(s) in premiRNA:66
miRNA sequence and Count(AGO1/wt):UGCCUGGCUCCCUGUAUGCCA_2840.7/218.45
miRNA Position in PremiRNA:5-25
miRNA* sequence and Count(AGO1/wt):GCGUACAGAGUAGUCAAGCAUG_0/6.8

miRNA Position in PremiRNA:66-87
miRNA annonation:ath-miR160a/ath-miR160c/ath-miR160b

32. Degradome supported cutsite(s) in Genome:10578652/10578737/10578758
PremiRNA Locus in Genome:>Chr4(10578647-10578760)_plus strand
PremiRNA
sequence:CGGAUUCGCUUGGUGCAGGUCGGGAACCAAUUCGGCUGACACAGCCUCGUGACUUUUAAACCUUUAUUGGUUUGUGAGCAGGGAUUGGAUC
CCGCCUUGCAUCAACUGAAUCGG
PremiRNA secondary structure:(.((((((.((((((((((.(((((.((((((((((((...)))))...((.(((.((((((......)))))).))))).))))))).))))).)))))))))).)))))).)
Degradome supported cutsite(s) in premiRNA:6/91/112
miRNA sequence and Count(AGO1/wt):UCGCUUGGUGCAGGUCGGGAA_1608.34/435.31
miRNA Position in PremiRNA:6-26
miRNA* sequence and Count(AGO1/wt):CCCGCCUUGCAUCAACUGAAU_6.82/471.12
miRNA Position in PremiRNA:91-111
miRNA annonation:ath-miR168b/ath-miR168a

33. Degradome supported cutsite(s) in Genome:12353109/12353130
PremiRNA Locus in Genome:>Chr4(12352440-12353134)_plus strand
PremiRNA
sequence:GUUCUAAAUUUGAAGCUUAUAAAAACCCAUCACUACUUUUGCAUACUUGUAUCCGCAGUGUAUUUCCUCGCAUCUACCAUCCCUUUUCUACG
CCUCUCUCCCUCUCUCUCUUUCUCCAUCAAAUCUUGUUUUGUUCAAACUCUCUCUCUCUCAUCUAUUCUCUCCAUACAAUACAUGAAUAUACAUACAU
ACCAUCAUCUUCUUUUCCCAUCUCUAGUUUUUCAUCAAUCUUCUGAUGUUCCAAACGCUCUAUCUCUUCAUAUACAUACAUACGAAUAUAUUAUUGU
UGUCAUAGAUCCAUUUAGAAUCACUUUAGCUUUUAGAUGAGAUCUAGGGUUUCUUUGUUUUCUUUCAAAUUUUGUUGCAUAUUCUUCUAAAUCAUG
GUUUUUCGCUUGCUAGGUUAUAGAUCCAUGCAAAUAUGGAGUAGAUGUACAAACACACGCUCGGACGCAUAUUACACAUGUUCAUACACUUAAUACU
CGCUGUUUUGAAUUGAUGUUUUAGGAAUAUAUAUGUAGAGAGAGCUUCCUUGAGUCCAUUCACAGGUCGUGAUAUGAUUCAAUUAGCUUCCGACUC
AUUCAUCCAAAUACCGAGUCGCCAAAAUUCAAACUAGACUCGUUAAAUGAAUGAAUGAUGCGGUAGACAAAUUGGAUCAUUGAUUCUCUUUGAUUG
GACUGAAGGGAGCUCCCUCUCUC
PremiRNA secondary
structure:(...................................((((((..((..((((.(((((........((((((((...((((......)))).............................((((((.......((((...(((.....(((.......................((((..(((((......))))).
...))))....................(((.((((........)))))))..(((((((((((((((.(......).))))))))).)))))).))).....))).....)))).......((((((.....((((((((.((((.........)))))))).))))...)))))).))))))))))))))........)))))))))..))........((((((..(((
.(((((..(((......))))))))))).....)))))).))))))(((.((((((((((.((((((.(((.(((..((.(.(((((((((((.(((.(((..((((((((.((..((.((((((................)))))).))..)).))))))))..))).)).).))))))))))).))..))).))).)))))).))))))))).)))...)

Degradome supported cutsite(s) in premiRNA:670/691
miRNA sequence and Count(AGO1/wt):UUGGACUGAAGGGAGCUCCCU_5570.55/779.19

miRNA Position in PremiRNA:670-690

miRNA* sequence and Count(AGO1/wt):AGAGCUUCCUUGAGUCCAUUC_2.15/11.8

miRNA Position in PremiRNA:521-541

miRNA annonation:ath-miR319b/ath-miR319a

34. Degradome supported cutsite(s) in Genome:12625128/12625742

PremiRNA Locus in Genome:>Chr4(12625123-12625744)_plus strand

PremiRNA sequence:AUGUCUAGACCAUUUGUGAGAAGGGAGUUUUUGUUUACACCAAUACCCCCAGUCUCUAAAUUUGUAAGAAGUAUUAUGCUCAAUUAAGGAACAUAGCUAGUUCGACAUACCAUACUACCCUUUUUAAAACUAUCCUAUAUGUUUGAUGCUAGCAUAGCGUAAUUUUGUGUUCUCAUGGUGCAGCAGGGUUAGUUUAUUGUGUACCUCUAGAUAUAUAUUCUCUUGCUGCGUAGGGUUCCCAAGCUGCCAAAAACUUUUAAAAAUUAGUGAUCUGUUCCCCAAACCCCCAUUCAAUAAGAAAGGUCUACCUGUAGCUCACAGUCACAGCUAUUAGAGCUGUCCUUGCUUCUUUGGGUUUACUGUUUUAGUUAAUUAGUUACCCUAUGAAAGUCUGAUCCUUCAGAAGUUAGGUAUAGAAGUAAGUCGAUUAAGUUCAUUCAUGACUUUUCCAGAUGUUGCAUACAUAACUUUUUUUAUUUGUAUUCGAACUAGUCCAAGCCGAUUCUCAAAAUCAUAUAAAAUCAUUUACACCUUGGUCAUGGUAGCUAAGAAUAUUGUAUCUAAAAUUGGGGAGUGGGGAGAUGUUUGGUUAUAUUCCCUUCUCAUCGAUGGUCUAGAUGU

PremiRNA secondary structure:(((((((((((((.((((((((((((..........(((((((.((((((.(((((((.((((.....((((((..((((.(((((((......((((((((((.((((..(((((...(((((((((......((((......((((((...))))))).....)))).......(((((((((((...((..(((.((((........)))))))..))))))))))...((....)).....)))).....))))))))).))))).......................(((((((((((.....((((((((......((((((.....)))))).(((((((((..((((..((((...........)))).)))))..(((...(((((....)))))..))).....)))))))))..((..((((((.......))))).))..)).))))))......))))))))))))).))))))))))))))).......((((.....)))).................)))))))....).)))....))))))).....)))).))))))).))))).).)).))))))...)))))))))))).))))))))))))))

Degradome supported cutsite(s) in premiRNA:6/620

miRNA sequence and Count(AGO1/wt):UAGACCAUUUGUGAGAAGGGA_659.77/505.92

miRNA Position in PremiRNA:6-26

miRNA* sequence and Count(AGO1/wt):CCUUCUCAUCGAUGGUCUAGA_7.18/94.82

miRNA Position in PremiRNA:599-619

miRNA annonation:ath-miR824

35. Degradome supported cutsite(s) in Genome:15074945

PremiRNA Locus in Genome:>Chr4(15074940-15075033)_plus strand

PremiRNA sequence:AAAACUGACAGAAGAGAGUGAGCACAUGCAGGCACUGUUAUGUGUCUAUAACUUUGCGUGUGCGUGCUCACCUCUCUUUCUGUCAGUUGCCUAU

PremiRNA secondary structure:(.(((((((((((((((((((((((..(((.(((..((((((....))))))..))).)))..))))))).))))).))))))))))).....)

Degradome supported cutsite(s) in premiRNA:6

miRNA sequence and Count(AGO1/wt):UGACAGAAGAGAGUGAGCAC_341.54/238.06

miRNA Position in PremiRNA:6-25
miRNA* sequence and Count(AGO1/wt):GCUCACCUCUCUUUCUGUCAGU_7.18/45.01
miRNA Position in PremiRNA:66-87
miRNA annonation:ath-miR156e/ath-miR156f/ath-miR156d/ath-miR156a/ath-miR156c/ath-miR156b

36. Degradome supported cutsite(s) in Genome:287586
PremiRNA Locus in Genome:>Chr5(287581-287739)_plus strand
PremiRNA
sequence:CAAGAUGGAGAAGCAGGGCACGUGCAUUACUAGCUCAUAUAUACACUCUCACCACAAAUGCGUGUAUAUAUGCGGAAUUUUGUGAUAUAGA
UGUGUGUGUGUGUUGAGUGUGAUGAUAUGGAUGAGUUAGUUCUUCAUGUGCCCAUCUUCACCAUCAUG
PremiRNA secondary structure:((.(((((.((((..(((((((((....((((((((((.(((((((.((((.((((.((((((.(((((.(((((....)))))))))).))))))...)))).)))).)))....)))).))))))))))....)))))))))..)))).))))).))

Degradome supported cutsite(s) in premiRNA:6
miRNA sequence and Count(AGO1/wt):UGGAGAAGCAGGGCACGUGCA_798.61/107.83
miRNA Position in PremiRNA:6-26
miRNA* sequence and Count(AGO1/wt):AUGUGCCCAUCUUCACCAUC_0/0
miRNA Position in PremiRNA:140-156
miRNA annonation:ath-miR164b/ath-miR164a

37. Degradome supported cutsite(s) in Genome:3867213
PremiRNA Locus in Genome:>Chr5(3867208-3867312)_plus strand
PremiRNA
sequence:GGAGGUGACAGAAGAGAGUGAGCACACAUGGUGGUUUCUUGCAUGCUUUUUUGAUUAGGGUUUCAUGCUUGAAGCUAUGUGUGCUUACUCU
CUCUCUGUCACCCC
PremiRNA secondary structure:((.(((((((((((((((((((((((((((((...(((..(((((((((.......))))...)))))..))))))))))))))))))))))).)))))))))))
Degradome supported cutsite(s) in premiRNA:6
miRNA sequence and Count(AGO1/wt):UGACAGAAGAGAGUGAGCAC_341.54/238.06
miRNA Position in PremiRNA:6-25
miRNA* sequence and Count(AGO1/wt):GCUUACUCUCUCUCUGUCACC_0/0
miRNA Position in PremiRNA:83-103
miRNA annonation:ath-miR156e/ath-miR156f/ath-miR156d/ath-miR156a/ath-miR156c/ath-miR156b

38. Degradome supported cutsite(s) in Genome:4691128
PremiRNA Locus in Genome:>Chr5(4691027-4691132)_plus strand

PremiRNA
sequence:CUCGACAGGGUUGAUAUGAGAACACACGAGUAAUCAACGGCUGUAAUGACGCUACGUCAUUGUUACAGCUCUCGUUUUCAUGUGUUCUCAGGUCACCCCUGCUGAG
PremiRNA secondary structure:((((.(((((.((((.((((((((((.(((.(((....(((((((((((((...))))))...)))))))...)))))).)))))))))).)))).))))).))))
Degradome supported cutsite(s) in premiRNA:102
miRNA sequence and Count(AGO1/wt):UGUGUUCUCAGGUCACCCCUG_84.31/0.2
miRNA Position in PremiRNA:81-101
miRNA* sequence and Count(AGO1/wt):GGGUUGAUAUGAGAACACACG_0.72/1
miRNA Position in PremiRNA:8-28
miRNA annonation:ath-miR398c/ath-miR398b

39. Degradome supported cutsite(s) in Genome:9098879/9098897
PremiRNA Locus in Genome:>Chr5(9098806-9098901)_plus strand
PremiRNA
sequence:UCAAUAUAUAUAGUCCAAUCUAUUGAAGUACUAGUACACCAGCUCUAAUAAGCUGAUGUGGGUAAGUAGUUCAAUAGAUUGGACUAUGUAUAUUAA
PremiRNA secondary structure:(.(((((((((((((((((((((((((.((((.(..((((((((......)))))..)))..).)))).))))))))))))))))))))))))).)
Degradome supported cutsite(s) in premiRNA:74/92
miRNA sequence and Count(AGO1/wt):UCAAUAGAUUGGACUAUGUAU_0.36/8
miRNA Position in PremiRNA:71-91
miRNA* sequence and Count(AGO1/wt):AUAUAGUCCAAUCUAUUGAAG_0/0
miRNA Position in PremiRNA:8-28
miRNA annonation:ath-miR860

40. Degradome supported cutsite(s) in Genome:9136126
PremiRNA Locus in Genome:>Chr5(9136121-9136223)_plus strand
PremiRNA
sequence:GAUGGUGACAGAAGAGAGUGAGCACACAUGGUGGCUUUCUUGCAUAUUUGAAGGUUCCAUGCUUGAAGCUAUGUGUGCUCACUCUCUAUCCGUCACCCCCUUC
PremiRNA secondary structure:((.((((((.((((((((((((((((((((((....(((..((((.............))))..))))))))))))))))))))))))).)).))))))....))
Degradome supported cutsite(s) in premiRNA:6
miRNA sequence and Count(AGO1/wt):UGACAGAAGAGAGUGAGCAC_341.54/238.06
miRNA Position in PremiRNA:6-25
miRNA* sequence and Count(AGO1/wt):GCUCACUCUCUAUCCGUCACC_0/0

miRNA Position in PremiRNA:77-97
miRNA annonation:ath-miR156e/ath-miR156f/ath-miR156d/ath-miR156a/ath-miR156c/ath-miR156b

41. Degradome supported cutsite(s) in Genome:9852684
PremiRNA Locus in Genome:>Chr5(9852679-9852770)_plus strand
PremiRNA
sequence:CUUGAUGGAGAAGCAGGGCACGUGCGAACACAAAUGAAAUCGAUCGGUACUUGUUGAUCAUAUUUUCGCACGUGUUCUACUACUCCAACAC
G
PremiRNA secondary structure:(.((.(((((.((.((((((((((((((..(....).....((((((......)))))).....)))))))))))))).)).))))).)).)
Degradome supported cutsite(s) in premiRNA:6
miRNA sequence and Count(AGO1/wt):UGGAGAAGCAGGGCACGUGCG_20.45/4
miRNA Position in PremiRNA:6-26
miRNA* sequence and Count(AGO1/wt):CACGUGUUCUACUACUCCAAC_1.08/14.4
miRNA Position in PremiRNA:69-89
miRNA annonation:ath-miR164c

42. Degradome supported cutsite(s) in Genome:13611904
PremiRNA Locus in Genome:>Chr5(13611803-13611927)_plus strand
PremiRNA
sequence:UACUUUUCCACAGCUUUCUUGAACUUUCUUUUUCAUUUCCAUUGUUUUUUUCUUAAACAAAAGUAAGAAGAAAAAAAACUUUAAGAUUAAG
CAUUUUGGAAGCUCAAGAAAGCUGUGGGAAAACA
PremiRNA secondary structure:(..((((((((((((((((((.(((((................(((((((((((........)))))))))))....(((.......)))......))))).))))))))))))))))))..)
Degradome supported cutsite(s) in premiRNA:102
miRNA sequence and Count(AGO1/wt):UUCCACAGCUUUCUUGAACUU_8813.79/3823.74
miRNA Position in PremiRNA:6-26
miRNA* sequence and Count(AGO1/wt):GCUCAAGAAAGCUGUGGGAAA_71.75/183.04
miRNA Position in PremiRNA:102-122
miRNA annonation:ath-miR396b

43. Degradome supported cutsite(s) in Genome:17516378/17516399
PremiRNA Locus in Genome:>Chr5(17516303-17516403)_plus strand
PremiRNA
sequence:GUUCAGGUGAAUGAUGCCUGGCUCGAGACCAUUCAAUCUCAUGAUCUCAUGAUUAUAACGAUGAUGAUGAUGAUGUCGGACCAGGCUUCAU
UCCCCUCAAC

PremiRNA secondary structure:(((.(((.((((((.((((((..(((.(.((((..(((((((((((....))))))...)).)))...)))).).)))..)))))).)))))).))).)))
Degradome supported cutsite(s) in premiRNA:76/97
miRNA sequence and Count(AGO1/wt):UCGGACCAGGCUUCAUUCCCC_56156.81/2154.13
miRNA Position in PremiRNA:76-96
miRNA* sequence and Count(AGO1/wt):UGAAUGAUGCCUGGCUCGAGA_1.44/0.2
miRNA Position in PremiRNA:8-28
miRNA annonation:ath-miR166b/ath-miR166f/ath-miR166a/ath-miR166g/ath-miR166c/ath-miR166d/ath-miR166e

44. Degradome supported cutsite(s) in Genome:19567227
PremiRNA Locus in Genome:>Chr5(19567222-19567349)_plus strand
PremiRNA
sequence:UACAGUUGAAAUUGUAGAUUUCGUACAUGACAAUAAUUUGCUCGAGCAUCACUUUCAUGAUUUUGUGGUCUGUGUUGCCGUAUUUGAGAGC
AUACAUUUCAUGUUCGAAAUCUACGAUUUUAACUCUA
PremiRNA secondary structure:((.(((((((((((((((((((((.((((((.(((....(((((..(((.(((..((((......))))...))).))).........)))))...))))))))).)))))))))))))))))))).))
Degradome supported cutsite(s) in premiRNA:6
miRNA sequence and Count(AGO1/wt):UUGAAAUUGUAGAUUUCGUAC_0/0
miRNA Position in PremiRNA:6-26
miRNA* sequence and Count(AGO1/wt):UCGAAAUCUACGAUUUUAACU_0.36/0
miRNA Position in PremiRNA:105-125
miRNA annonation:ath-miR4243

45. Degradome supported cutsite(s) in Genome:21161609
PremiRNA Locus in Genome:>Chr5(21161504-21161634)_plus strand
PremiRNA
sequence:AUGAAUUGUCAGUAUAAAUCUUUGAUCUGAUACUCUAUUAGUUAGUAUGUGAAUUUCUUUUUAACAAUUUUCUUAUUCAUGCGAGAGCUA
UCAAUAAGUAUUAGAACAAAGUUUUAUACUGACAAUUCAAU
PremiRNA secondary structure:((((((((((((((((((.(((((.(((((((((....(((((.(((((.(((................)))......)))))...)))))......))))))))).))))).))))))))))))))))).))
Degradome supported cutsite(s) in premiRNA:106
miRNA sequence and Count(AGO1/wt):ACAAAGUUUUAUACUGACAAU_6.82/29.41
miRNA Position in PremiRNA:106-126
miRNA* sequence and Count(AGO1/wt):UGUCAGUAUAAAUCUUUGAUC_13.27/3.8
miRNA Position in PremiRNA:7-27
miRNA annonation:ath-miR4245

46. Degradome supported cutsite(s) in Genome:23636961
PremiRNA Locus in Genome:>Chr5(23636956-23637057)_plus strand
PremiRNA
sequence:CUAUAAAGCUCAGGAGGGAUAGCGCCAUGGCUCACCAGUGCUGUAUGUUUUGUAUAUGCGUACAUGUAUAUCUGUUGGCGCUAUCCAUCCU
GAGUUCCAUAG
PremiRNA secondary structure:((((..(((((((((.(((((((((((.((....)).((((((((((.........))))))).))))......))))))))))).)))))))))..))))
Degradome supported cutsite(s) in premiRNA:6
miRNA sequence and Count(AGO1/wt):AAGCUCAGGAGGGAUAGCGCC_87.9/375.29
miRNA Position in PremiRNA:6-26
miRNA* sequence and Count(AGO1/wt):CGCUAUCCAUCCUGAGUUCCA_0/6
miRNA Position in PremiRNA:79-99
miRNA annonation:ath-miR390b/ath-miR390a

47. Degradome supported cutsite(s) in Genome:24293160/24293211
PremiRNA Locus in Genome:>Chr5(24293156-24293233)_plus strand
PremiRNA sequence:UUGCUUCGCAGGAGAGAUAGCGCCAUCACCUCUUCUAAGAAGUUAACUAGUGGUGACGGUAUCUCUCCUACGUAGCAA

PremiRNA secondary structure:(((((.((.((((((((((.((.((((((..((((...)))).......)))))).)).)))))))))).)).)))))
Degradome supported cutsite(s) in premiRNA:5/56
miRNA sequence and Count(AGO1/wt):UUCGCAGGAGAGAUAGCGCCA_96.51/30.21
miRNA Position in PremiRNA:5-25
miRNA* sequence and Count(AGO1/wt):ACGGUAUCUCUCCUACGUAGC_7.53/590.74
miRNA Position in PremiRNA:56-76
miRNA annonation:ath-miR391

48. Degradome supported cutsite(s) in Genome:78932/78953
PremiRNA Locus in Genome:>Chr1(79037-78927)_minus strand
PremiRNA
sequence:GUUGAGGGGAAUGUUGUCUGGAUCGAGGAUAUUAUAGAUAUAUACAUGUGUAUGUUAAUGAUUCAAGUGAUCAUAGAGAGUAUCCUCGGA
CCAGGCUUCAUCCCCCCCAAC
PremiRNA secondary structure:((((.((((.(((..((((((.(((((((((((...((((((((....)))))))).((((((.....))))))....))))))))))).))))))..))).)))).))))
Degradome supported cutsite(s) in premiRNA:107/86
miRNA sequence and Count(AGO1/wt):UCGGACCAGGCUUCAUCCCCC_17565.86/412.3
miRNA Position in PremiRNA:86-106

miRNA* sequence and Count(AGO1/wt):GGAAUGUUGUCUGGAUCGAGG_0/30.21
miRNA Position in PremiRNA:8-28
miRNA annonation:ath-miR165b/ath-miR165a

49. Degradome supported cutsite(s) in Genome:234017
PremiRNA Locus in Genome:>Chr1(234152-234013)_minus strand
PremiRNA
sequence:AUGAUGCGCAAAUGCGGAUAUCAAUGUAAAUCAGGACAACAAACCAAUAGAGGUGCUCAGCGAAAACAACAAAAUGUGAAAGGGUUUAUC
AGAAACAUUGGGGUCAUGAUAUACCUUUAUAUCCGCAUUUGCGCAUCAU
PremiRNA secondary structure:(((((((((((((((((((((.((.(((.((((.(((..(((.......((...((((.......(((......))).....))))...)).......)))..))).)))).))).)).)))))))))))))))))))))
Degradome supported cutsite(s) in premiRNA:137
miRNA sequence and Count(AGO1/wt):CUUUAUAUCCGCAUUUGCGCA_10.05/15.2
miRNA Position in PremiRNA:116-136
miRNA* sequence and Count(AGO1/wt):CGCAAAUGCGGAUAUCAAUGU_0/1.6
miRNA Position in PremiRNA:7-27
miRNA annonation:ath-miR2112-3p

50. Degradome supported cutsite(s) in Genome:3961367/3961388
PremiRNA Locus in Genome:>Chr1(3961454-3961365)_minus strand
PremiRNA
sequence:CGCGAGAUAUUAGUGCGGUUCAAUCAAAUAGUCGUCCUCUUAACUCAUGGAGAACGGUGUUGUUCGAUUGAGCCGUGCCAAUAUCACGCG

PremiRNA secondary structure:((((.((((((.(..((((((((((.(((((((((.((((........)))).))))..))))).))))))))))..).)))))).))))
Degradome supported cutsite(s) in premiRNA:89/68
miRNA sequence and Count(AGO1/wt):UUGAGCCGUGCCAAUAUCACG_318.94/40.81
miRNA Position in PremiRNA:68-88
miRNA* sequence and Count(AGO1/wt):AGAUAUUAGUGCGGUUCAAUC_5.38/25.21
miRNA Position in PremiRNA:5-25
miRNA annonation:ath-miR171c/ath-miR171b

51. Degradome supported cutsite(s) in Genome:4820431
PremiRNA Locus in Genome:>Chr1(4820499-4820405)_minus strand

PremiRNA sequence:UCUCCUCUUCUCCAAAUAGUUUAGGUUAGCUGACAUAUAAUAAUUCAAUAGACCCCAGUUUCUCCAGAUAACCUAACUAUUUUGAGAAGAAGUGA
PremiRNA secondary structure:((.(.(((((((.((((((((.((((((.(((............((....))............))).)))))))))))))).))))))).).))
Degradome supported cutsite(s) in premiRNA:70
miRNA sequence and Count(AGO1/wt):UCUUCUCCAAAUAGUUUAGGUU_14.71/6.6
miRNA Position in PremiRNA:6-27
miRNA* sequence and Count(AGO1/wt):CCUAACUAUUUUGAGAAGAAG_4.66/2.2
miRNA Position in PremiRNA:72-92
miRNA annonation:ath-miR830-5p

52. Degradome supported cutsite(s) in Genome:11834118
PremiRNA Locus in Genome:>Chr1(11834159-11834089)_minus strand
PremiRNA sequence:AUUUGAACCUGUCAAUUGGUAUCAAAGCUUCUAAAUCUUUGAAGCUCUGAUACCAAAUGAUGGAAUCAAAU

PremiRNA secondary structure:((((((..((((((.(((((((((.((((((.........)))))).))))))))).))))))..))))))
Degradome supported cutsite(s) in premiRNA:43
miRNA sequence and Count(AGO1/wt):AGCUCUGAUACCAAAUGAUGGAAU_0.36/3.6
miRNA Position in PremiRNA:43-66
miRNA* sequence and Count(AGO1/wt):CCUGUCAAUUGGUAUCAAAGCUUC_0/0.6
miRNA Position in PremiRNA:8-31
miRNA annonation:ath-miR829.1

53. Degradome supported cutsite(s) in Genome:23345389/23345408
PremiRNA Locus in Genome:>Chr1(23345506-23345382)_minus strand
PremiRNA sequence:AGUUUUAGGGCGCCUCUCCAUUGGCAGGUCCUUUACUUCCAAAUAUACACAUACAUAUAUGAAUAUCGAAAAUUUCCGAUGAUCGAUUUAUAAAUGACCUGCCAAAGGAGAGUUGCCCUGAAACU
PremiRNA secondary structure:((((((((((((.((((((.((((((((((.((((.......(((((........)))))((.(((((........))))).))......)))).)))))))))).)))))).))))))))))))
Degradome supported cutsite(s) in premiRNA:119/100
miRNA sequence and Count(AGO1/wt):UGCCAAAGGAGAGUUGCCCUG_33.37/9.4
miRNA Position in PremiRNA:100-120
miRNA* sequence and Count(AGO1/wt):UUAGGGCGCCUCUCCAUUGGC_0.36/0
miRNA Position in PremiRNA:8-28

miRNA annonation:ath-miR399b/ath-miR399c

54. Degradome supported cutsite(s) in Genome:27713234/27713255
PremiRNA Locus in Genome:>Chr1(27713416-27713233)_minus strand
PremiRNA
sequence:GUAGAGCUCCUUAAAGUUCAAACAUGAGUUGAGCAGGGUAAAGAAAAGCUGCUAAGCUAUGGAUCCCAUAAGCCCUAAUCCUUGUAAAGUA
AAAAAGGAUUUGGUUAUAUGGAUUGCAUAUCUCAGGAGCUUUAACUUGCCCUUUAAUGGCUUUUACUCUUCUUUGGAUUGAAGGGAGCUCUAC

PremiRNA secondary
structure:((((((((((((..((((((((...((((.(((((((((((...((((((.((.((.((((.(..(((((((((..(((((((...........))))))).)))).)))))..).)))).)).)).))))))...)))))))......))))..))))...))))))))..))))))))))))

Degradome supported cutsite(s) in premiRNA:184/163
miRNA sequence and Count(AGO1/wt):UUUGGAUUGAAGGGAGCUCUA_74556.8/4894.6
miRNA Position in PremiRNA:163-183
miRNA* sequence and Count(AGO1/wt):GAGCUCCUUAAAGUUCAAACA_0/3.4
miRNA Position in PremiRNA:4-24
miRNA annonation:ath-miR159a

55. Degradome supported cutsite(s) in Genome:22930095/22930116
PremiRNA Locus in Genome:>Chr1(22930184-22930090)_minus strand
PremiRNA
sequence:AAAUACGAGAUAUUGGUGCGGUUCAAUCAGAAAACCGUACUCUUUUGUUUUAAAGAUCGGUUUAUUUGAUUGAGCCGUGCCAAUAUCACGC
GUUU
PremiRNA secondary structure:(((..((.((((((((..(((((((((((((((((((...(((((......))))).)))))).)))))))))))))..)))))))).))..)))
Degradome supported cutsite(s) in premiRNA:91/70
miRNA sequence and Count(AGO1/wt):UUGAGCCGUGCCAAUAUCACG_318.94/40.81
miRNA Position in PremiRNA:70-90
miRNA* sequence and Count(AGO1/wt):AGAUAUUGGUGCGGUUCAAUC_10.05/49.81
miRNA Position in PremiRNA:8-28
miRNA annonation:ath-miR171c/ath-miR171b

56. Degradome supported cutsite(s) in Genome:4142352/4142464
PremiRNA Locus in Genome:>Chr2(4142468-4142328)_minus strand

PremiRNA sequence:UAUUCUUCCACAGCUUUCUUGAACUGCAAAACUUCUUCAGAUUUUUUUUUUUUUCUUUUGAUAUCUCUUACGCAUAAAAUAGUGAUUUUCUUCAUAUCUCUGCUCGAUUGAUUUGCGGUUCAAUAAAGCUGUGGGAAGAUA

PremiRNA secondary structure:(((..((((((((((((.((((((((((((.(.((..((((............((....))....................((((......))))...))))...))..).)))))))))))).))))))))))))..)))

Degradome supported cutsite(s) in premiRNA:118/6

miRNA sequence and Count(AGO1/wt):UUCCACAGCUUUCUUGAACUG_6282.7/1100.87

miRNA Position in PremiRNA:6-26

miRNA* sequence and Count(AGO1/wt):GUUCAAUAAAGCUGUGGGAAG_187.28/274.07

miRNA Position in PremiRNA:118-138

miRNA annonation:ath-miR396a

57. Degradome supported cutsite(s) in Genome:10676554

PremiRNA Locus in Genome:>Chr2(10676558-10676443)_minus strand

PremiRNA sequence:GAAACUGACAGAAGAGAGUGAGCACACAAAGGCAAUUUGCAUAUCAUUGCACUUGCUUCUCUUGCGUGCUCACUGCUCUUUCUGUCAGAUUCCGGUGCUGAUCUCUUUGGCCUGUC

PremiRNA secondary structure:((..(((((((((((((((((((((.(((((((((..((((......)))).))))))...))).))))))))).))).)))))))))....(((.((..(.....)..)))))))

Degradome supported cutsite(s) in premiRNA:6

miRNA sequence and Count(AGO1/wt):UGACAGAAGAGAGUGAGCAC_341.54/238.06

miRNA Position in PremiRNA:6-25

miRNA* sequence and Count(AGO1/wt):GCUCACUGCUCUUUCUGUCAGA_0/12.8

miRNA Position in PremiRNA:68-89

miRNA annonation:ath-miR156e/ath-miR156f/ath-miR156d/ath-miR156a/ath-miR156c/ath-miR156b

58. Degradome supported cutsite(s) in Genome:3366369

PremiRNA Locus in Genome:>Chr3(3366416-3366349)_minus strand

PremiRNA sequence:UGCUUCUUUGUCUACAAUUUUGGAAAAAGUGAUGACGCCAUUGCUCUUUCCCAAAUGUAGACAAAGCA

PremiRNA secondary structure:(((((...((((((((.(((.(((((.((..(((....)))..)).))))).))))))))))))))))

Degradome supported cutsite(s) in premiRNA:49

miRNA sequence and Count(AGO1/wt):UCCCAAAUGUAGACAAAGCA_143709.88/25016.54

miRNA Position in PremiRNA:49-68

miRNA* sequence and Count(AGO1/wt):CUUUGUCUACAAUUUUGGAAA_19.37/76.62

miRNA Position in PremiRNA:3-26

miRNA annonation:ath-miR158a

59. Degradome supported cutsite(s) in Genome:3599812
PremiRNA Locus in Genome:>Chr3(3599897-3599787)_minus strand
PremiRNA
sequence:GCUGUUGGAGCAUCAUCAAGAUUCACAAAUCAUCAAGUAUUCGUGUAAAUAAACCCAUUUAUGAUUAGAUUUUUGAUGUAUGUAUGAGAAU
CUUGAUGAUGCUGCAGCUGC
PremiRNA secondary structure:((.((((.((((((((((((((((.((.(((((((((...((.(((((((......)))))))....))..))))))).))...)).)))))))))))))))).)))).))
Degradome supported cutsite(s) in premiRNA:87
miRNA sequence and Count(AGO1/wt):AGAAUCUUGAUGAUGCUGCAG_42.33/6.8
miRNA Position in PremiRNA:87-107
miRNA* sequence and Count(AGO1/wt):GGAGCAUCAUCAAGAUUCACA_0/0
miRNA Position in PremiRNA:7-27
miRNA annonation:ath-miR172d/ath-miR172c

60. Degradome supported cutsite(s) in Genome:4359010
PremiRNA Locus in Genome:>Chr3(4359207-4359007)_minus strand
PremiRNA
sequence:GUGUGCAGCCAAGGAUGACUUGCCGAUUUAAAUGAUCUUUCUUUAUACUCUAUUAAGACAAUUUAGUUUCAAACUUUUUUUUUUUUUUUU
UUUGAAGGAUUCAGGAAGAAAUUAGGAUAUAUUAUUCCGUAUAAAAUACAAGAUAUAUAAAACCAAAAAGAAAAAGUAACAUGAUCGGCAAGUUGU
CCUUGGCUACACGU
PremiRNA secondary
structure:(((((.(((((((((..((((((((((((((((..((((...............))))..))))).......((((((((((((((((((((((((.....)))))))))))...(((........)))........................)))))))))))))).....)))))))))))..))))))))).)))))

Degradome supported cutsite(s) in premiRNA:199
miRNA sequence and Count(AGO1/wt):CAGCCAAGGAUGACUUGCCGA_85.74/254.06
miRNA Position in PremiRNA:6-26
miRNA* sequence and Count(AGO1/wt):CAAGUUGUCCUUGGCUACACG_1.08/1
miRNA Position in PremiRNA:178-198
miRNA annonation:ath-miR169a

61. Degradome supported cutsite(s) in Genome:6244694
PremiRNA Locus in Genome:>Chr3(6244698-6244524)_minus strand
PremiRNA
sequence:GGUUGUUGACAGAAGAUAGAGAGCACUAAGGAUGACAUGCAAGUACAUACAUAUAUAUCAUCACACCGCAUGUGGAUGAUAAAAUAUGUAU

AACAAAUUCAAAGAAAGAGAGGGAGAGAAAGAGAGAGAACCUGCAUCUCUACUCUUUUGUGCUCUCUAUACUUCUGUCACCACC

PremiRNA secondary structure:(((.((.((((((((((((((((((...((((.((......))..((((((((.(((((((.(((.....))))))))))..))))))))).....))))...(((((((..(((((...((.(......)))...))))).))))))))))))))))).)))))))))).)))

Degradome supported cutsite(s) in premiRNA:6
miRNA sequence and Count(AGO1/wt):UUGACAGAAGAUAGAGAGCAC_1637.4/715.58
miRNA Position in PremiRNA:6-26
miRNA* sequence and Count(AGO1/wt):GCUCUCUAUACUUCUGUCACC_8.25/230.46
miRNA Position in PremiRNA:152-172
miRNA annonation:ath-miR157b/ath-miR157a/ath-miR157c

62. Degradome supported cutsite(s) in Genome:19659579
PremiRNA Locus in Genome:>Chr3(19659671-19659576)_minus strand
PremiRNA sequence:UGUCGUCUCGGUUCGCGAUCCACAAGUAAUCUUUUGUGGAGAUUAUGAAAACAAUCAUCACGAGAGGCUACGUGUGGGUGGCAAACAAAGACGACA
PremiRNA secondary structure:((((((((..(((.((.((((((((.(((..((((((((..((((.(....).))))..))))))))..))).))))))).)).)))..))))))))
Degradome supported cutsite(s) in premiRNA:94
miRNA sequence and Count(AGO1/wt):UCUCGGUUCGCGAUCCACAAG_4.66/1
miRNA Position in PremiRNA:6-26
miRNA* sequence and Count(AGO1/wt):UGUGGGUGGCAAACAAAGACG_0/0
miRNA Position in PremiRNA:73-93
miRNA annonation:ath-miR851-5p

63. Degradome supported cutsite(s) in Genome:369856/369877
PremiRNA Locus in Genome:>Chr4(369996-369851)_minus strand
PremiRNA sequence:GUUGUGGGGAAUGUUGUUUGGAUCGAGGAUAUCAUAAACGCAUACACAUGUUUAUAUGUUAUGAUGCAUUAUAUGACUGAUGUAAUGUACAUAUAUAUACAUACAUGCCACAUGGUAUCGUCGGACCAGGCUUCAUCCCCCUCAAC
PremiRNA secondary structure:((((.((((.(((..((((((.((((.((((((((....((((....((((..((((((.(((.((((((((((.....)))))))))))))))))))))))..))))...)))))))).)))).))))))..))).)))).))))

Degradome supported cutsite(s) in premiRNA:142/121
miRNA sequence and Count(AGO1/wt):UCGGACCAGGCUUCAUCCCCC_17565.86/412.3
miRNA Position in PremiRNA:121-141

miRNA* sequence and Count(AGO1/wt):GGAAUGUUGUUUGGAUCGAGG_0.36/3.4
miRNA Position in PremiRNA:8-28
miRNA annonation:ath-miR165b/ath-miR165a

64. Degradome supported cutsite(s) in Genome:1535440
PremiRNA Locus in Genome:>Chr4(1535652-1535435)_minus strand
PremiRNA
sequence:UAUACAAUACUACUUUUUCAUCCAUUAAUCCCCUUACAAUGUCGAGUAAACGAAGCAUCUGUCCCCUGGUAUUGUCUUCGAGCUUGGUGUG
UUUUUCUAGCCAGCCCCAAGUUCUCGAGUUGAUCAUUGUUUGUAUUCUGACACAUUAUUUGGGGACGAGAUGUUUUGUUGACUCGAUAUAAGAAGG
GGCUUUAUGGAAGAAAUUGUAGUAUUAUAUA
PremiRNA secondary
structure:((((.((((((((..((((.(((((.((.((((((...((((((((.(((((((((((((((((..(((.((((...((((((((.(((((.....)))..)).))))))))..((((....((.....)).)))).))))...)))..)))))).))))))))))))).)))))))))...)))))).)).))))).))))..))))
))))).)))))
Degradome supported cutsite(s) in premiRNA:214
miRNA sequence and Count(AGO1/wt):UAUGGAAGAAAUUGUAGUAUU_1074.14/218.45
miRNA Position in PremiRNA:193-213
miRNA* sequence and Count(AGO1/wt):UACUACUUUUUCAUCCAUUAA_0/0
miRNA Position in PremiRNA:8-28
miRNA annonation:ath-miR447a.2-3p

65. Degradome supported cutsite(s) in Genome:8504145/8504169
PremiRNA Locus in Genome:>Chr4(8504315-8504140)_minus strand
PremiRNA
sequence:UCAAGAUAUCAGAUAUUUCACGAAGAUAUCUGCAUAACAGCUACGAGGAAAUUGUGAUUUUAUCGUUUCACAAACAUCAACAGCUCCAUGG
AUCGUUAUGGACGGAUAAAUCACAAUCACUAUCCUUUUUCUAGCAGCUGUUGAGCAGGUUUCUUCGUGAAUAUCUGGCAUUUUGG

PremiRNA secondary structure:(((((((..((((((((.((((((((.((((((.((((((((.((((((.((((((((((.((((((.....)))........(.((((((((....)))))))).)))))))))))))).....)))))......).)))))))).)))))).))))))))))))))))..)))))))

Degradome supported cutsite(s) in premiRNA:172/148
miRNA sequence and Count(AGO1/wt):UUCUUCGUGAAUAUCUGGCAU_6.82/1
miRNA Position in PremiRNA:151-171
miRNA* sequence and Count(AGO1/wt):UAUCAGAUAUUUCACGAAGAUA_0/0
miRNA Position in PremiRNA:8-29
miRNA annonation:ath-miR780.2

66. Degradome supported cutsite(s) in Genome:15415511
PremiRNA Locus in Genome:>Chr4(15415511-15415427)_minus strand
PremiRNA sequence:CUGACAGAAGAGAGUGAGCACACAAAGGCACUUUGCAUGUUCGAUGCAUUUGCUUCUCUUGCGUGCUCACUGCUCUAUCUGUCAG

PremiRNA secondary structure:(((((((((((((((((((((.((((((((...(((((.....)))))..)))))...))).))))))))).)))).))))))))
Degradome supported cutsite(s) in premiRNA:2
miRNA sequence and Count(AGO1/wt):UGACAGAAGAGAGUGAGCAC_341.54/238.06
miRNA Position in PremiRNA:2-21
miRNA* sequence and Count(AGO1/wt):GCUCACUGCUCUAUCUGUCAGA_0.36/96.42
miRNA Position in PremiRNA:65-86
miRNA annonation:ath-miR156e/ath-miR156f/ath-miR156d/ath-miR156a/ath-miR156c/ath-miR156b

67. Degradome supported cutsite(s) in Genome:1188211
PremiRNA Locus in Genome:>Chr5(1188304-1188206)_minus strand
PremiRNA
sequence:UGUAGGCGCAGCACCAUUAAGAUUCACAUGGAAAUUGAUAAAUACCCUAAAUUAGGGUUUUGAUAUGUAUAUGAGAAUCUUGAUGAUGCUG
CAUCAACA
PremiRNA secondary structure:(((.(..((((((.(((((((((((.((((...((...((((.((((((...))))))))))..))...)))).))))))))))).))))))..).)))
Degradome supported cutsite(s) in premiRNA:95
miRNA sequence and Count(AGO1/wt):AGAAUCUUGAUGAUGCUGCAU_2707.96/2035.1
miRNA Position in PremiRNA:74-94
miRNA* sequence and Count(AGO1/wt):GCAGCACCAUUAAGAUUCACA_0.36/35.41
miRNA Position in PremiRNA:8-28
miRNA annonation:ath-miR172a/ath-miR172b-3p

68. Degradome supported cutsite(s) in Genome:2634936/2634957
PremiRNA Locus in Genome:>Chr5(2635022-2634934)_minus strand
PremiRNA
sequence:CGCUGGAGGCAGCGGUUCAUCGAUCUCUUCCUGUGAACACAUUAAAAAUGUAAAAGCAUGAAUAGAUCGAUAAACCUCUGCAUCCAGCG

PremiRNA secondary structure:(((((((.((((.((((.((((((((.(((.(((....((((.....))))....))).))).)))))))).)))).)))).)))))))
Degradome supported cutsite(s) in premiRNA:88/67
miRNA sequence and Count(AGO1/wt):UCGAUAAACCUCUGCAUCCAG_3161.44/389.9

miRNA Position in PremiRNA:67-87

miRNA* sequence and Count(AGO1/wt):GGAGGCAGCGGUUCAUCGAUC_0.36/0.2

miRNA Position in PremiRNA:5-25

miRNA annonation:ath-miR162b/ath-miR162a

69. Degradome supported cutsite(s) in Genome:3456666/3456733

PremiRNA Locus in Genome:>Chr5(3456737-3456640)_minus strand

PremiRNA sequence:GAAGUUGACAGAAGAGAGUGAGCACACAAAGGGGAAGUUGUAUAAAAGUUUUGUAUAUGGUUGCUUUUGCGUGCUCACUCUCUUUUUGUCAUAACUUC

PremiRNA secondary structure:(((((((((((((((((((((((((.((((((.((...(((((((.....)))))))...)).)))))).)))))))))))).))))))))..)))))

Degradome supported cutsite(s) in premiRNA:73/6

miRNA sequence and Count(AGO1/wt):UGACAGAAGAGAGUGAGCAC_341.54/238.06

miRNA Position in PremiRNA:6-25

miRNA* sequence and Count(AGO1/wt):GCUCACUCUCUUUUUGUCAUA_2.15/34.81

miRNA Position in PremiRNA:73-93

miRNA annonation:ath-miR156e/ath-miR156f/ath-miR156d/ath-miR156a/ath-miR156c/ath-miR156b

70. Degradome supported cutsite(s) in Genome:7740610/7740631

PremiRNA Locus in Genome:>Chr5(7740705-7740605)_minus strand

PremiRNA sequence:UGAAGUCGCUGGAGGCAGCGGUUCAUCGAUCAAUUCCUGUGAAUAUUUAUUUUUGUUUACAAAAGCAAGAAUCGAUCGAUAAACCUCUGCAUCCAGCGCUG

PremiRNA secondary structure:(..((.(((((((.((((.((((.(((((((.((((.((((((((........)))))))).......)))).))))))).)))).)))).))))))))))

Degradome supported cutsite(s) in premiRNA:97/76

miRNA sequence and Count(AGO1/wt):UCGAUAAACCUCUGCAUCCAG_3161.44/389.9

miRNA Position in PremiRNA:76-96

miRNA* sequence and Count(AGO1/wt):GGAGGCAGCGGUUCAUCGAUC_0.36/0.2

miRNA Position in PremiRNA:11-31

miRNA annonation:ath-miR162b/ath-miR162a

71. Degradome supported cutsite(s) in Genome:16660490

PremiRNA Locus in Genome:>Chr5(16660998-16660464)_minus strand

PremiRNA sequence:AAAAGCCAUAACUCCUUCAUUUUCUUUAGACAUCUCUUCUUCUCUCAUCUCUCUUUUCUUCUCUCUUUUCCUCACAUAAACUCUCUUUUUUUACUAUUAAAUCCAUAUGGUACCUCAAAUUAAUCUAUGGUCAUCUAGGGUUAUCUUGAAGAUUAGAAUUGAUUCUAGCACGCACAGAGAGGAAGAUCAUUGCAUCCAGAAUCACAAACAUGGCCUAUCUUUUAUCUUUUCUUUUUGAUCUAAGUCACUGUUUUAUGCUAUAUAUAGUAUAAUCAAAUUCUUUACAUGUGCUUGUAUGUAUGCGUAUAUAUAGUAACGGAAUUGUUAAUAUGCUUAUAGAUGUUGAGUUGGUGGAGGAAGAGAGCUUUCUUCGGUCCACUCAUGGAGUAAUAUGUGAGAUUUAAUUGACUCUCGACUCAUUCAUCCAAAUACCAAAUGAAAGAAUUUGUUCUCAUAUGGUAAAUGAAUGAAUGAUGCGAGAGACAAAUUGAGUCUUCACUUCUCUAUGCUUGGACUGAAGGGAGCUCCCUAUUUU

PremiRNA secondary structure:((((........(((((((((..(((..((((((((((((((((......((..((((..((..........................((((((.........))))))..((((..((((((.(((....)))))))))..)))).))..))))..)).....(....)..))))))))))..(((.((((((........(((((((((..(((................)))....)))).)))))..(((((((((((((((...........((((......))))..))))).)))))))))))..)))..)))....))).....))))))))))..)))))))))...(((((..((((((((((..(((((((......((((((((((((..((((((..((((((((.((..(((((.((((..(((....)))))))).)))))..)).))))))))..))))))...)))))))))).))))..)))))))..))))))))))..)))))....))))

Degradome supported cutsite(s) in premiRNA:510

miRNA sequence and Count(AGO1/wt):UUGGACUGAAGGGAGCUCCCU_5570.55/779.19

miRNA Position in PremiRNA:510-530

miRNA* sequence and Count(AGO1/wt):AGAGCUUUCUUCGGUCCACUC_22.6/31.01

miRNA Position in PremiRNA:361-381

miRNA annonation:ath-miR319b/ath-miR319a

72. Degradome supported cutsite(s) in Genome:16775545

PremiRNA Locus in Genome:>Chr5(16775662-16775520)_minus strand

PremiRNA sequence:UUGAGGGGAAUGUUGUCUGGCACGAGGCCCUUAACUUAGAUCUAUAUUUGAUUAUAUAUAUAUGUCUCUUCUUUAUUCAUUAGUCUAUACAUGAAUGAUCAUUUUACGGUUAAUGACGUCGGACCAGGCUUCAUUCCCCUCAA

PremiRNA secondary structure:((((((((((((..((((((..(((.(.(.((((((.((((.(((((..........))))).)))).....((((((((((.....)).))))))))).........)))))).).).)))..))))))..))))))))))))

Degradome supported cutsite(s) in premiRNA:119

miRNA sequence and Count(AGO1/wt):UCGGACCAGGCUUCAUUCCCC_56156.81/2154.13

miRNA Position in PremiRNA:119-139

miRNA* sequence and Count(AGO1/wt):GGAAUGUUGUCUGGCACGAGG_0.36/35.41

miRNA Position in PremiRNA:7-27

miRNA annonation:ath-miR166b/ath-miR166f/ath-miR166a/ath-miR166g/ath-miR166c/ath-miR166d/ath-miR166e

73. Degradome supported cutsite(s) in Genome:18358894

PremiRNA Locus in Genome:>SChr5(18358898-18358800)_minus strand

PremiRNA sequence:CGGAUUCGCUUGGUGCAGGUCGGGAACUGAUUGGCUGACACCGACACGUGUCUUGUCAUGGUUGGUUUGUGAGCUCCCGUCUUGUAUCAACUGAAUCGG

PremiRNA secondary structure:(.((((((.((((((((((.(((((........(((.((((((((.((((......))))))))))..)).)))))))).))))))))))).)))))).)

Degradome supported cutsite(s) in premiRNA:6

miRNA sequence and Count(AGO1/wt):UCGCUUGGUGCAGGUCGGGAA_1608.34/435.31

miRNA Position in PremiRNA:6-26

miRNA* sequence and Count(AGO1/wt):CCCGUCUUGUAUCAACUGAAU_2.15/98.42

miRNA Position in PremiRNA:76-96

miRNA annonation:ath-miR168b/ath-miR168a

74. Degradome supported cutsite(s) in Genome:771384/771406

PremiRNA Locus in Genome:>Chr2(771517-771382)_minus strand

PremiRNA sequence:GAAGACACUGAAGGACCUAAACUAACAAAGGUAAACGGCUCAGUGUGCGGGGUAUUACACUCGGUUUAAUGUCUGAAUGCGAUAAUCCGCACGAUGAUCUCUUUAUCUUUGUUUGUUUAGGUCCCUUAGUUUCUUC

PremiRNA secondary structure:(((((.(((((.((((((((((.((((((((((((.((.(((.(((((((..(((..((.((((........)))).))..)))..))))))).))).)).))))))))))))).)))))))))).))))).)))))

Degradome supported cutsite(s) in premiRNA:135/113

miRNA sequence and Count(AGO1/wt):CACUGAAGGACCUAAACUAAC_15.43/7.6

miRNA Position in PremiRNA:6-26

miRNA* sequence and Count(AGO1/wt):UUGUUUAGGUCCCUUAGUUUC_17.58/12.2

miRNA Position in PremiRNA:113-133

miRNA annonation:ath-miR840-3p

Supplementary table S2c. Discovery of known miRNAs from roots of *Arabidopsis*.

1. Degradome supported cutsite(s) in Genome:28656
PremiRNA Locus in Genome:>Chr1(28545-28660)_plus strand
PremiRNA
sequence:UGGUGUUGUGCAAGAAGGAGAAGCAAAGUCUGUCUAUGUAUUAUGAGAUAGCUACUUCUAUGGCUAGGAUAUAUGUUGUACAAGACCGGCU UUUCUUCUACUUCUUGCACAACCUG
PremiRNA secondary structure:(((.((((((((((((.(((((.((((((.((((.(((((((((...((((((......))))))...))))....))))))))).)))))).))))).)))))))))))))))
Degradome supported cutsite(s) in premiRNA:112
miRNA sequence and Count(AGO1/wt):UUUUCUUCUACUUCUUGCACA_10.22/4.03
miRNA Position in PremiRNA:91-111
miRNA* sequence and Count(AGO1/wt):UGCAAGAAGGAGAAGCAAAGU_0/0
miRNA Position in PremiRNA:9-29
miRNA annonation:ath-miR838

2. Degradome supported cutsite(s) in Genome:6220813/6220834
PremiRNA Locus in Genome:>Chr1(6220645-6220838)_plus strand
PremiRNA
sequence:AGGAAGAGCUCCUUGAAGUUCAAUGGAGGGUUUAGCAGGGUGAAGUAAAGCUGCUAAGCUAUGGAUCCCAUAAGCCUUAUCAAAUUCAAUA UAAUUGAUGAUAAGGUUUUUUUUAUGGAUGCCAUAUCUCAGGAGCUUUCACUUACCCCUUUAAUGGCUUCACUCUUCUUUGGAUUGAAGGGAGCUC UUCAUCU
PremiRNA secondary
structure:((((((((((((((..(((((((.(((((((..(((((((..((((((((((.((.((.(((((...((((((((((((((...(((((...)))))))))))))))))).....))))))...))))).)).)).)))))).))))..))))......)))..)))))))).)))))))..))))))))))))..))
Degradome supported cutsite(s) in premiRNA:169/190
miRNA sequence and Count(AGO1/wt):UUUGGAUUGAAGGGAGCUCUU_13623.99/1256.02
miRNA Position in PremiRNA:169-189
miRNA* sequence and Count(AGO1/wt):GAGCUCCUUGAAGUUCAAUGG_38.12/44.04
miRNA Position in PremiRNA:6-26
miRNA annonation:ath-miR159b

3. Degradome supported cutsite(s) in Genome:6740590
PremiRNA Locus in Genome:>Chr1(6740498-6740593)_plus strand
PremiRNA
sequence:UUGCUUCAGGUAUGAUUGACUUCAAAAAAUACCUUGAAACUAUAAACCUCAGUUUCUUUGAAUUUGAUUUUUAAAGUCAAUAAUACCUUGA AGCAA

PremiRNA secondary structure:(((((((((((((.((((((((.(((((.......((((((.........))))))...........))))).)))))))).))))).))))))))
Degradome supported cutsite(s) in premiRNA:93
miRNA sequence and Count(AGO1/wt):UCAGGUAUGAUUGACUUCAAA_7.07/6.04
miRNA Position in PremiRNA:6-26
miRNA* sequence and Count(AGO1/wt):UAAAGUCAAUAAUACCUUGAAG_0/0.29
miRNA Position in PremiRNA:72-93
miRNA annonation:ath-miR864-5p

4. Degradome supported cutsite(s) in Genome:9364561
PremiRNA Locus in Genome:>Chr1(9364477-9364564)_plus strand
PremiRNA
sequence:CCAUGAGUUCCCUUUAACGCUUCAUUGUUAAAUACUCAAAGCCACAUUGGUUUGUAUACAACACUGAAGUGUUUGGGGGACUCUUGG

PremiRNA secondary structure:(((.(((((((((..(((((((((.((((..((((...(((((.....)))))))))..)))).)))))))))..))))))))).)))
Degradome supported cutsite(s) in premiRNA:85
miRNA sequence and Count(AGO1/wt):CUGAAGUGUUUGGGGGACUC_393.02/60.44
miRNA Position in PremiRNA:64-84
miRNA* sequence and Count(AGO1/wt):GUUCCCUUUAACGCUUCAUUG_0.39/1.73
miRNA Position in PremiRNA:7-27
miRNA annonation:ath-miR395f/ath-miR395c/ath-miR395b

5. Degradome supported cutsite(s) in Genome:17825722
PremiRNA Locus in Genome:>Chr1(17825720-17825829)_plus strand
PremiRNA
sequence:GAUCAAUGCAUUGAAAGUGACUACAUCGGGGUUCCGAUUUUUUUUGUUCUUCAUAUGAUGAAGCGGAAACAGUAAUCAACCCUGGUUUAGU
CACUUUCACUGCAUUAAUC
PremiRNA secondary structure:(((.((((((.(((((((((((((.(((((((((..((((....(((((((((((...))))))....))))).))))))))))))).))))))))))).)))))).)))
Degradome supported cutsite(s) in premiRNA:3
miRNA sequence and Count(AGO1/wt):UCAAUGCAUUGAAAGUGACUA_2979.47/1615.79
miRNA Position in PremiRNA:3-23
miRNA* sequence and Count(AGO1/wt):GUCACUUUCACUGCAUUAAUC_12.18/8.06
miRNA Position in PremiRNA:90-110
miRNA annonation:ath-miR161.2

6. Degradome supported cutsite(s) in Genome:20041586
PremiRNA Locus in Genome:>Chr1(20041581-20041665)_plus strand
PremiRNA
sequence:GAGAUUGAGCCAAGGAUGACUUGCCGAUUUUCUCAACGAAUCUUACUGAUUAUGGUAUCCGGCAAGUUGACUUUGGCUCUGUUUC

PremiRNA secondary structure:(((((.(((((((((.((((((((((...(((.....)))...((((......))))..)))))))))).))))))))).)))))
Degradome supported cutsite(s) in premiRNA:6
miRNA sequence and Count(AGO1/wt):UGAGCCAAGGAUGACUUGCCG_29.48/10.94
miRNA Position in PremiRNA:6-26
miRNA* sequence and Count(AGO1/wt):GCAAGUUGACUUUGGCUCUGU_0/36.55
miRNA Position in PremiRNA:62-82
miRNA annonation:ath-miR169g-5p/ath-miR169d/ath-miR169e/ath-miR169f

7. Degradome supported cutsite(s) in Genome:22149808/22149735
PremiRNA Locus in Genome:>Chr1(22149730-22149831)_plus strand
PremiRNA
sequence:CGAAAUCUCUCUGUUGUGAAGUCAAACAUGAGUAUGAAUUAACAUUAAUGGAUCUUUUUGAUUCAUAAUACUCAUGUUUGGUUUCACAGUA
GAUAGAUAUCG
PremiRNA secondary structure:(((.((((.(((((((((((..(((((((((((((............(((((((.....))))))).)))))))))))))..))))))))))).)))).)))
Degradome supported cutsite(s) in premiRNA:79/6
miRNA sequence and Count(AGO1/wt):UCUCUCUGUUGUGAAGUCAAA_9.83/4.32
miRNA Position in PremiRNA:6-26
miRNA* sequence and Count(AGO1/wt):UGGUUUCACAGUAGAUAGAUA_0/0.86
miRNA Position in PremiRNA:79-99
miRNA annonation:ath-miR859

8. Degradome supported cutsite(s) in Genome:24554011
PremiRNA Locus in Genome:>Chr1(24553982-24554031)_plus strand
PremiRNA sequence:CGUACACUUACAUCUGUACACUAAAAGUGUUGUACAAAUUUAAGUGUACG
PremiRNA secondary structure:((((((((((.((.(((((((.......).)))))).)).))))))))))
Degradome supported cutsite(s) in premiRNA:30
miRNA sequence and Count(AGO1/wt):UUGUACAAAUUUAAGUGUACG_1.57/1.73
miRNA Position in PremiRNA:30-50
miRNA* sequence and Count(AGO1/wt):UACACUUACAUCUGUACACUA_0/0

miRNA Position in PremiRNA:3-23
miRNA annonation:ath-miR5014a-3p

9. Degradome supported cutsite(s) in Genome:24884359
PremiRNA Locus in Genome:>Chr1(24884354-24884581)_plus strand
PremiRNA
sequence:AGUCGUUGAAGAGGACUUGGAACUUCGAUAUUAUCCCCCGUGUUUUGUCCAGUUUCUCUAUAGGUUGUGCCUGAAAAUAUGGGUUUCUAGG
UUUAAUUGAUGAAAAGAGAUAACGUAAGUCAUGCACAUGUAAUUAAAUUCAUGCCCCUAACUAUUAAUUCUUGCAAGUAAUUAAAAAAUAAAACGU
ACACAAAAUAUAUUUAUGAUCACUAAUCCUUUUUAACUACU
PremiRNA secondary
structure:(((.((((((((((.((((......(((...)))....((((((((...((((.....((((.(((((.(((...((((.((((((...((((.....)))).)))))))..)))))..))).))))).(((..........))).))))....((((((((....)).))))))......))))....)))))))))..........))))))))
))))))).)))
Degradome supported cutsite(s) in premiRNA:6
miRNA sequence and Count(AGO1/wt):UUGAAGAGGACUUGGAACUUCGAU_6225.81/2072.83
miRNA Position in PremiRNA:6-29
miRNA* sequence and Count(AGO1/wt):UAUCCCCCGUGUUUUGUCC_0.39/0
miRNA Position in PremiRNA:35-225
miRNA annonation:ath-miR163

10. Degradome supported cutsite(s) in Genome:24921104
PremiRNA Locus in Genome:>Chr1(24921099-24921200)_plus strand
PremiRNA
sequence:UAGUGUUGACAGAAGAUAGAGAGCACAGAUGAUAAGAUACAAUUCCUCGCAGCUUCUUUGCAUCUUACUCCUUUGUGCUCUCUAGCCUUCU
GUCAUCACCCG
PremiRNA secondary structure:(.(((.(((((((((.(((((((((((((.((((((((.(((...............))).))))))).)).)))))))))))))..))))))))).)))..)
Degradome supported cutsite(s) in premiRNA:6
miRNA sequence and Count(AGO1/wt):UUGACAGAAGAUAGAGAGCAC_3.14/2.59
miRNA Position in PremiRNA:6-26
miRNA* sequence and Count(AGO1/wt):GCUCUCUAGCCUUCUGUCAUCA_0/0
miRNA Position in PremiRNA:77-98
miRNA annonation:ath-miR157b/ath-miR157a/ath-miR157c

11. Degradome supported cutsite(s) in Genome:26638083
PremiRNA Locus in Genome:>Chr1(26638000-26638107)_plus strand

PremiRNA
sequence:AGAGAGCAACAAAAUCAGUGUGUAUUUGUUAAGACAAAUGUUUAUAUAAGUCGUCCAUGCGUUGUACAUUUAAUCUUAACUAAUACGCAUU
GAGUUUCGUUGCUUCU
PremiRNA secondary structure:(((((((((((((.((((((((((((.(((((((.((((((....((((..(((....)))))))))))))..))))))).)))))))))))).))).))))))))))
Degradome supported cutsite(s) in premiRNA:84
miRNA sequence and Count(AGO1/wt):UACGCAUUGAGUUUCGUUGCUU_13.36/11.8
miRNA Position in PremiRNA:84-105
miRNA* sequence and Count(AGO1/wt):CAACAAAAUCAGUGUGUAUU_0/0
miRNA Position in PremiRNA:6-26
miRNA annonation:ath-miR777

12. Degradome supported cutsite(s) in Genome:29422546
PremiRNA Locus in Genome:>Chr1(29422456-29422570)_plus strand
PremiRNA
sequence:AACGUUGCACUACGUGACAUUGAAACUGUCUUUCAACAUUCCAAUAUUUCAACUUUCGAAUACCCAAUAUUUGGUUUGUUCAAAGACAUUU
UCGAUGUCUAGCAGUGCCAAUGUU
PremiRNA secondary structure:(((((((((((....((((((((((.(((((((.((((..((((((((.................)))).))))..)))).))))))).))))))))))....)))).)))))))
Degradome supported cutsite(s) in premiRNA:91
miRNA sequence and Count(AGO1/wt):UUCGAUGUCUAGCAGUGCCA_226.77/108.22
miRNA Position in PremiRNA:91-110
miRNA* sequence and Count(AGO1/wt):GCACUACGUGACAUUGAAAC_0.79/13.53
miRNA Position in PremiRNA:7-26
miRNA annonation:ath-miR775

13. Degradome supported cutsite(s) in Genome:22150026
PremiRNA Locus in Genome:>Chr1(22149939-22150030)_plus strand
PremiRNA
sequence:UUUCAGAUGGCUGUUUGGGUAACUAAUAUUUAAGAUUUUGGUCAAUUUAAGUUACCAAUUUAUAUAUUGGUUACCCAUAUGGCCAUCUCAA
A
PremiRNA secondary structure:(((.((((((((((.((((((((((((((.((((...(((((.(((....))))))))))))).))))))))))))))).)))))))))).)))
Degradome supported cutsite(s) in premiRNA:88
miRNA sequence and Count(AGO1/wt):UUGGUUACCCAUAUGGCCAUC_1.97/2.01
miRNA Position in PremiRNA:67-87
miRNA* sequence and Count(AGO1/wt):UGGCUGUUUGGGUAACUAAUA_0/0

miRNA Position in PremiRNA:8-28
miRNA annonation:ath-miR774a

14. Degradome supported cutsite(s) in Genome:1041012
PremiRNA Locus in Genome:>Chr2(1040937-1041037)_plus strand
PremiRNA sequence:GUGAAAUUUCAAAGGAGUGGCAUGUGAACACAUAUCCUAUGGUUUCUUCAAAUUUCCAUUGAAACCAUUGAGUUUUGUGUUCUCAGGUCACCCCUUUGAAU
PremiRNA secondary structure:((......(((((((.(((((.((.(((((((..((..((((((((.............)))))))).))....))))))).)).))))).)))))))))
Degradome supported cutsite(s) in premiRNA:76
miRNA sequence and Count(AGO1/wt):UGUGUUCUCAGGUCACCCCUU_2.36/0.86
miRNA Position in PremiRNA:76-96
miRNA* sequence and Count(AGO1/wt):GGAGUGGCAUGUGAACACAUA_0/0.29
miRNA Position in PremiRNA:14-34
miRNA annonation:ath-miR398a

15. Degradome supported cutsite(s) in Genome:9560866/9560887
PremiRNA Locus in Genome:>Chr2(9560769-9560891)_plus strand
PremiRNA sequence:UAAUAUAGUUGAAUCUUCGAAAGUAUUUCAAGAAGUCAGCUGAGCUUUCUCGUCAUCACUUAUUAGUAAAUAUAGUCUCUAUAUUUUUGAUGAGUGAUGAUUGGAAAUUUCGUUGACUCAUUA
PremiRNA secondary structure:((((....(((((....(....)...)))))..(((((((.(((.(((((.(((((((((((((((.((((((((...))))))))))))))))))))))).))))).)))))))))).))))
Degradome supported cutsite(s) in premiRNA:98/119
miRNA sequence and Count(AGO1/wt):UGAUUGGAAAUUUCGUUGACU_26.73/14.68
miRNA Position in PremiRNA:98-118
miRNA* sequence and Count(AGO1/wt):UCAGCUGAGCUUUCUCGUCAUC_64.06/25.9
miRNA Position in PremiRNA:36-57
miRNA annonation:ath-miR779.2

16. Degradome supported cutsite(s) in Genome:11159774
PremiRNA Locus in Genome:>Chr2(11159706-111159799)_plus strand
PremiRNA sequence:GACUCGUUCAAGCACCAGCUCGAAGAAGCUUAGCUAAUUUAUCUUAGAAAAUAAUGAAAAAGCUAUGCUUCUCAAGAAGGUGCAUGAACAAGUU

PremiRNA secondary structure:((((.(((((.(((((..((.((.(((((.(((((..(((((..(.....)..)))))..))))).))))))).))..))))).))))).))))
Degradome supported cutsite(s) in premiRNA:69
miRNA sequence and Count(AGO1/wt):UUCUCAAGAAGGUGCAUGAAC_1.97/0.29
miRNA Position in PremiRNA:69-89
miRNA* sequence and Count(AGO1/wt):UCAAGCACCAGCUCGAAGAAGC_0/0
miRNA Position in PremiRNA:8-29
miRNA annonation:ath-miR825

17. Degradome supported cutsite(s) in Genome:15611882
PremiRNA Locus in Genome:>Chr2(15611875-15611978)_plus strand
PremiRNA
sequence:AGAGAAGUGAGAUGAAAUCUUUGAUUGGAAAUUUCAUGUAGACGAUGCUAUCACCCCAUAUGACCAAAAGAAAUUCCCAAUUAAAGAUUUC
AUCUUACUUCCCU
PremiRNA secondary structure:((.((((((((((((((((((((((((.((((((..(((.....)))..(((.......)))......)))))).))))))))))))))))))))))))).))
Degradome supported cutsite(s) in premiRNA:8
miRNA sequence and Count(AGO1/wt):AAUUAAAGAUUUCAUCUUACU_0/1.44
miRNA Position in PremiRNA:79-99
miRNA* sequence and Count(AGO1/wt):UGAGAUGAAAUCUUUGAUUGG_36.16/9.21
miRNA Position in PremiRNA:8-28
miRNA annonation:ath-miR1886.3

18. Degradome supported cutsite(s) in Genome:16061971
PremiRNA Locus in Genome:>Chr2(16061966-16062046)_plus strand
PremiRNA sequence:CUGUAAAGCUCAGGAGGGAUAGCGCCAUGAUGAUCACAUUCGUUAUCUAUUUUUGGCGCUAUCCAUCCUGAGUUUCAUUG

PremiRNA secondary structure:(.((.((((((((((.((((((((((((.((((((.((....)).))).)))...)))))))))))).)))))))))).)).)
Degradome supported cutsite(s) in premiRNA:6
miRNA sequence and Count(AGO1/wt):AAGCUCAGGAGGGAUAGCGCC_121.05/98.14
miRNA Position in PremiRNA:6-26
miRNA* sequence and Count(AGO1/wt):CGCUAUCCAUCCUGAGUUUCA_4.32/40.87
miRNA Position in PremiRNA:58-78
miRNA annonation:ath-miR390b/ath-miR390a

19. Degradome supported cutsite(s) in Genome:16340282/16340363

PremiRNA Locus in Genome:>Chr2(16340279-16340363)_plus strand
PremiRNA sequence:GUAUGCCUGGCUCCCUGUAUGCCAUAUGCUGAGCCCAUCGAGUAUCGAUGACCUCCGUGGAUGGCGUAUGAGGAGCCAUGCAUAU

PremiRNA secondary structure:((((((.(((((((..(((((((((.(((.(((..(((((.....)))))..))).))).)))))))))..))))))).))))))
Degradome supported cutsite(s) in premiRNA:4/85
miRNA sequence and Count(AGO1/wt):UGCCUGGCUCCCUGUAUGCCA_1765.83/263.06
miRNA Position in PremiRNA:4-24
miRNA* sequence and Count(AGO1/wt):GCGUAUGAGGAGCCAUGCAUA_0.79/23.89
miRNA Position in PremiRNA:64-84
miRNA annonation:ath-miR160a/ath-miR160c/ath-miR160b

20. Degradome supported cutsite(s) in Genome:19176240
PremiRNA Locus in Genome:>Chr2(19176122-19176265)_plus strand
PremiRNA
sequence:UUGAGGGGACUGUUGUCUGGCUCGAGGACUCUGGCUCGCUCUAUUCAUGUUGGAUCUCUUUCGAUCUAACAAUCGAAUUGAACCUUCAGAU
UUCAGAUUUGAUUAGGGUUUUAGCGUCUUCGGACCAGGCUUCAUUCCCCCCAA
PremiRNA secondary structure:(((.(((((.((..((((((..(((((((.((((...((((((.((((((((((((......)))))))))...(((((((....))))..))).....))).)))))).)))).)))))))..))))))..)).))))).)))
Degradome supported cutsite(s) in premiRNA:119
miRNA sequence and Count(AGO1/wt):UCGGACCAGGCUUCAUUCCCC_14661.96/1228.68
miRNA Position in PremiRNA:120-140
miRNA* sequence and Count(AGO1/wt):GGACUGUUGUCUGGCUCGAGG_1.57/56.99
miRNA Position in PremiRNA:7-27
miRNA annonation:ath-miR166b/ath-miR166f/ath-miR166a/ath-miR166g/ath-miR166c/ath-miR166d/ath-miR166e

21. Degradome supported cutsite(s) in Genome:19520770
PremiRNA Locus in Genome:>Chr2(19520766-19520851)_plus strand
PremiRNA
sequence:AUGUUGGAGAAGCAGGGCACGUGCAAACCAACAAACACGAAAUCCGUCUCAUUUGCUUAUUUGCACGUACUUAACUUCUCCAACAU

PremiRNA secondary structure:((((((((((((..(((.(((((((((....((((.(((.....)))....))))....))))))))).)))..))))))))))))
Degradome supported cutsite(s) in premiRNA:5
miRNA sequence and Count(AGO1/wt):UGGAGAAGCAGGGCACGUGCA_400.49/85.19
miRNA Position in PremiRNA:5-25
miRNA* sequence and Count(AGO1/wt):CACGUACUUAACUUCUCCAAC_0.39/0.29

miRNA Position in PremiRNA:64-84
miRNA annonation:ath-miR164b/ath-miR164a

22. Degradome supported cutsite(s) in Genome:6217561
PremiRNA Locus in Genome:>Chr3(6217490-6217584)_plus strand
PremiRNA sequence:CAAGUAUGGUUUUCUUGGCCCAUCCACUUCUAAUGUGUUUUGUAACUAAUGCAAAAUGUGCAUUAGAAGUGGAUUGGUCAAGGGAAGCGUACUUG
PremiRNA secondary structure:((((((((.((..(((((((.(((((((((((((((((((((((.....))))))))..))))))))))))))).)))))))..)).))))))))
Degradome supported cutsite(s) in premiRNA:72
miRNA sequence and Count(AGO1/wt):UGGAUUGGUCAAGGGAAGCGU_0.79/0.86
miRNA Position in PremiRNA:70-90
miRNA* sequence and Count(AGO1/wt):UUUUCUUGGCCCAUCCACUUC_1.57/0.29
miRNA Position in PremiRNA:8-28
miRNA annonation:ath-miR3440b-3p

23. Degradome supported cutsite(s) in Genome:8108090/8108172/8108193
PremiRNA Locus in Genome:>Chr3(8108090-8108190)_plus strand
PremiRNA sequence:UGAAGCUGCCAGCAUGAUCUAAUUAGCUUUCUUUAUCCUUUGUUGUGUUUCAUGACGAUGGUUAAGAGAUCAGUCUCGAUUAGAUCAUGUUCGCAGUUUCA
PremiRNA secondary structure:(((((((((.((((((((((((((((((.(((((..((.((((..((...))..)))).))...)))))..)).)).)))))))))))))).)))))))))
Degradome supported cutsite(s) in premiRNA:1/83/104
miRNA sequence and Count(AGO1/wt):UGAAGCUGCCAGCAUGAUCUA_921.24/505.4
miRNA Position in PremiRNA:1-21
miRNA* sequence and Count(AGO1/wt):GAUCAUGUUCGCAGUUUCACC_0/0
miRNA Position in PremiRNA:83-103
miRNA annonation:ath-miR167b/ath-miR167a

24. Degradome supported cutsite(s) in Genome:8236161
PremiRNA Locus in Genome:>Chr3(8236156-8236251)_plus strand
PremiRNA sequence:GUACUUUCGCUUGCAGAGAGAAAUCACAGUGGUCAAAAAAGUUGUAGUUUUCUUAAAGUCUCUUUCCUCUGUGAUUCUCUGUGUAAGCGAAAGAGC

PremiRNA secondary structure:((.(((((((((((.((((((.((((((.((.........(((.((....))))).........)).))))))))))).))))))))))).))
Degradome supported cutsite(s) in premiRNA:6
miRNA sequence and Count(AGO1/wt):UUCGCUUGCAGAGAGAAAUCAC_3875.95/1120.46
miRNA Position in PremiRNA:6-27
miRNA* sequence and Count(AGO1/wt):GAUUCUCUGUGUAAGCGAAAG_2.75/25.62
miRNA Position in PremiRNA:73-93
miRNA annonation:ath-miR173-5p

25. Degradome supported cutsite(s) in Genome:8346589/8346610
PremiRNA Locus in Genome:>Chr3(8346411-8346612)_plus strand
PremiRNA
sequence:GCUGCUCCCCUCUUUAGCUUGGAGAAGCCAGUAAAUAUCUUUCUCUUCAAGGAACUUGUGGGCCUCAAGAAAACCUGCUAUAAUUCUAGUU
UUUCAACAUUCCUCCUUCUGAUAUCAGCAACAACUCCUUACCCUUAAGAAUGGAGAAUGGAACUUCCUCAAAGAUAUUUACUUGGCCUCUGCAACCG
GAAAGGGGAGCAGC
PremiRNA secondary
structure:((((((((((.((..(.(((.(((.((((((((((((((((.......(((..(....)..)))...(((((...((((......)))))))))..(((((.(((..(((....)))..........((((....))))...))))))))..........)))))))))))).)))).))).))))..)).)))))))))))

Degradome supported cutsite(s) in premiRNA:179/200
miRNA sequence and Count(AGO1/wt):UCCCCUCUUUAGCUUGGAGAAG_10.22/4.89
miRNA Position in PremiRNA:6-27
miRNA* sequence and Count(AGO1/wt):UCUGCAACCGGAAAGGGGAGC_3.93/2.88
miRNA Position in PremiRNA:179-199
miRNA annonation:ath-miR853

26. Degradome supported cutsite(s) in Genome:19073542
PremiRNA Locus in Genome:>Chr3(19073446-19073545)_plus strand
PremiRNA
sequence:CUUUGAUAUUGGCCUGGUUCACUCAGAUCUUACCUGACCACACACGUAGAUAUACAUUAUUCUCUCUAGAUUAUCUGAUUGAGCCGCGCCA
AUAUCUCAG
PremiRNA secondary structure:((..(((((((((.(((((((.((((((.((((.((......)).))))...........(((....)))..)))))).))))))).)))))))))..))
Degradome supported cutsite(s) in premiRNA:97
miRNA sequence and Count(AGO1/wt):UGAUUGAGCCGCGCCAAUAUC_1.18/0.58
miRNA Position in PremiRNA:76-96
miRNA* sequence and Count(AGO1/wt):UAUUGGCCUGGUUCACUCAGA_2.75/0.29

miRNA Position in PremiRNA:7-27
miRNA annonation:ath-miR171a

27. Degradome supported cutsite(s) in Genome:20587991
PremiRNA Locus in Genome:>Chr3(20587914-20588016)_plus strand
PremiRNA
sequence:UGCUAUUGCAACAUCUUCAAGAUUCAGAAAUCAGAUUCUCUUAUGGGUUUUCUUUUGAGCCUUUAUUUUUUGGUUUGAGAAUCUUGAUGAU
GCUGCAGCGGCA
PremiRNA secondary structure:(((..(((((.((((.(((((((((..((((((((......((.((((((......)))))).))...))))))))..))))))))).)))).)))))..)))
Degradome supported cutsite(s) in premiRNA:78
miRNA sequence and Count(AGO1/wt):AGAAUCUUGAUGAUGCUGCAG_863.46/562.68
miRNA Position in PremiRNA:78-98
miRNA* sequence and Count(AGO1/wt):GCAACAUCUUCAAGAUUCAGA_0/77.13
miRNA Position in PremiRNA:8-28
miRNA annonation:ath-miR172d/ath-miR172c

28. Degradome supported cutsite(s) in Genome:22922301/22922321
PremiRNA Locus in Genome:>Chr3(22922206-22922325)_plus strand
PremiRNA
sequence:UUGAGGGGACUGUUGUCUGGCUCGAGGACUCUUAUUCUAAUACAAUCUCAUUUGAAUACAUUCAGAUCUGAUGAUUGAUUAGGGUUUUAGU
GUCGUCGGACCAGGCUUCAUUCCCCCCAA
PremiRNA secondary structure:(((.(((((.((..((((((..(((.(((.((.((((((((.((((((((((((((....))))))..))).)))))))))))))...)).))).))).))..)))))).)).))))).)))
Degradome supported cutsite(s) in premiRNA:96/116
miRNA sequence and Count(AGO1/wt):UCGGACCAGGCUUCAUUCCCC_14661.96/1228.68
miRNA Position in PremiRNA:96-116
miRNA* sequence and Count(AGO1/wt):GGACUGUUGUCUGGCUCGAGG_1.57/56.99
miRNA Position in PremiRNA:7-27
miRNA annonation:ath-miR166b/ath-miR166f/ath-miR166a/ath-miR166g/ath-miR166c/ath-miR166d/ath-miR166e

29. Degradome supported cutsite(s) in Genome:23406177
PremiRNA Locus in Genome:>Chr3(23406172-23406272)_plus strand
PremiRNA
sequence:ACAAGUGAAGCUGCCAGCAUGAUCUAUCUUUGGUUAAGAGAUGAAUGUGGAAACAUAUUGCUUAAACCCAAGCUAGGUCAUGCUCUGACAG
CCUCACUCCU

PremiRNA secondary structure:(..(((((.(((((.(((((((((((.(((.((((..(((...((((((....)))))).))).)))).))).)))))))))))..).)))).)))))..)
Degradome supported cutsite(s) in premiRNA:6
miRNA sequence and Count(AGO1/wt):UGAAGCUGCCAGCAUGAUCUA_921.24/505.4
miRNA Position in PremiRNA:6-26
miRNA* sequence and Count(AGO1/wt):GGUCAUGCUCUGACAGCCUCACU_0/0
miRNA Position in PremiRNA:76-98
miRNA annonation:ath-miR167b/ath-miR167a

30. Degradome supported cutsite(s) in Genome:9889047
PremiRNA Locus in Genome:>Chr4(9888982-9889070)_plus strand
PremiRNA
sequence:GUCGUGCCUGGCUCCCUGUAUGCCACAAGAAAACAUCGAUUUAGUUUCAAAAUCGAUCACUAGUGGCGUACAGAGUAGUCAAGCAUGAC

PremiRNA secondary structure:(((((((.(((((.((((((((((((.((.....((((((((.......))))))))..)).))))))))))).).))))).)))))))
Degradome supported cutsite(s) in premiRNA:66
miRNA sequence and Count(AGO1/wt):UGCCUGGCUCCCUGUAUGCCA_1765.83/263.06
miRNA Position in PremiRNA:5-25
miRNA* sequence and Count(AGO1/wt):GCGUACAGAGUAGUCAAGCAUG_1.18/10.94
miRNA Position in PremiRNA:66-87
miRNA annonation:ath-miR160a/ath-miR160c/ath-miR160b

31. Degradome supported cutsite(s) in Genome:10578652/10578737/10578758
PremiRNA Locus in Genome:>Chr4(10578647-10578760)_plus strand
PremiRNA
sequence:CGGAUUCGCUUGGUGCAGGUCGGGAACCAAUUCGGCUGACACAGCCUCGUGACUUUUAAACCUUUAUUGGUUUGUGAGCAGGGAUUGGAUC
CCGCCUUGCAUCAACUGAAUCGG
PremiRNA secondary structure:(.((((((.((((((((((.(((((.((((((((((((...)))))...((.(((.((((((......)))))).))))).))))))).))))).)))))))))).)))))).)
Degradome supported cutsite(s) in premiRNA:6/91/112
miRNA sequence and Count(AGO1/wt):UCGCUUGGUGCAGGUCGGGAA_349.79/174.99
miRNA Position in PremiRNA:6-26
miRNA* sequence and Count(AGO1/wt):CCCGCCUUGCAUCAACUGAAU_3.14/191.4
miRNA Position in PremiRNA:91-111
miRNA annonation:ath-miR168b/ath-miR168a

32. Degradome supported cutsite(s) in Genome:12353109/12353130

PremiRNA Locus in Genome:>Chr4(12352440-12353134)_plus strand

PremiRNA
sequence:GUUCUAAAUUUGAAGCUUAUAAAAACCCAUCACUACUUUUGCAUACUUGUAUCCGCAGUGUAUUUCCUCGCAUCUACCAUCCCUUUUCUACG
CCUCUCUCCCUCUCUCUCUUUCUCCAUCAAAUCUUGUUUUGUUCAAACUCUCUCUCUCUCAUCUAUUCUCUCCAUACAAUACAUGAAUAUACAUACAU
ACCAUCAUCUUCUUUUCCCAUCUCUAGUUUUUCAUCAAUCUUCUGAUGUUCCAAACGCUCUAUCUCUUCAUAUACAUACAUACGAAUAUAUUAUUGU
UGUCAUAGAUCCAUUUAGAAUCACUUUAGCUUUUAGAUGAGAUCUAGGGUUUCUUUGUUUUCUUUCAAAUUUUGUUGCAUAUUCUUCUAAAUCAUG
GUUUUUCGCUUGCUAGGUUAUAGAUCCAUGCAAAUAUGGAGUAGAUGUACAAACACACGCUCGGACGCAUAUUACACAUGUUCAUACACUUAAUACU
CGCUGUUUUGAAUUGAUGUUUUAGGAAUAUAUAUGUAGAGAGAGCUUCCUUGAGUCCAUUCACAGGUCGUGAUAUGAUUCAAUUAGCUUCCGACUC
AUUCAUCCAAAUACCGAGUCGCCAAAAUUCAAACUAGACUCGUUAAAUGAAUGAAUGAUGCGGUAGACAAAUUGGAUCAUUGAUUCUCUUUGAUUG
GACUGAAGGGAGCUCCCUCUCUC

PremiRNA secondary
structure:(.......................................((((((..((..((((.(((((........((((((((...((((......))))............................((((((.......((((...(((.....(((......................((((..(((((......))))).
...)))).....................(((.((((........)))))))..(((((((((((((((.(......).)))))))))).)))))).))).....))).....)))).......((((((.....((((((((.((((.........)))))))).))))...)))))).))))))))))))))........)))))))))..)).......((((((..(((
.(((((..(((......)))))))))))).....)))))).))))))(((.((((((((((.((((((.(((.(((..((.(.(((((((((((.(((.(((..((((((((.((..((.((((((...............)))))).))..)).))))))))..))).)).).))))))))))).))..))).))).)))))).))))))))))).)))...)

Degradome supported cutsite(s) in premiRNA:670/691

miRNA sequence and Count(AGO1/wt):UUGGACUGAAGGGAGCUCCCU_702.32/207.51

miRNA Position in PremiRNA:670-690

miRNA* sequence and Count(AGO1/wt):AGAGCUUCCUUGAGUCCAUUC_1.57/0.29

miRNA Position in PremiRNA:521-541

miRNA annonation:ath-miR319b/ath-miR319a

33. Degradome supported cutsite(s) in Genome:12625128/12625742

PremiRNA Locus in Genome:>Chr4(12625123-12625744)_plus strand

PremiRNA
sequence:AUGUCUAGACCAUUUGUGAGAAGGGAGUUUUUGUUUACACCAAUACCCCCCAGUCUCUAAAUUUGUAAGAAGUAUUAUGCUCAAUUAAGGA
ACAUAGCUAGUUCGACAUACCAUACUACCCUUUUUAAAACUAUCCUAUAUGUUUGAUGCUAGCAUAGCGUAAUUUUGUGUUCUCAUGGUGCAGCAGG
GUUAGUUUAUUGUGUACCUCUAGAUAUAUAUUCUCUUGCUGCGUAGGGUUCCCAAGCUGCCAAAAACUUUUAAAAAUUAGUGAUCUGUUCCCCAAAC
CCCCAUUCAAUAAGAAAGGUCUACCUGUAGCUCACAGUCACAGCUAUUAGAGCUGUCCUUGCUUCUUUGGGUUUACUGUUUUAGUUAAUUAGUUACC
CUAUGAAAGUCUGAUCCUUCAGAAGUUAGGUAUAGAAGUAAGUCGAUUAAGUUCAUUCAUGACUUUUCCAGAUGUUGCAUACAUAACUUUUUUUAU
UUGUAUUCGAACUAGUCCAAGCCGAUUCUCAAAAUCAUAUAAAAUCAUUUACACCUUGGUCAUGGUAGCUAAGAAUAUUGUAUCUAAAAUUGGGGA
GUGGGGAGAUGUUUGGUUAUAUUCCCUUCUCAUCGAUGGUCUAGAUGU

PremiRNA secondary
structure:(((((((((((((.((((((((((((..........((((((.((((((.(((((((.((((.....((((((..((((.((((((......(((((((((.((((..(((((...((((((((......((((......((((((...))))))).....))))......(((((((((((...((..(((.((((........)))
))))..))))))))))...((....)).....)))).....)))))))))).)))))......................(((((((((((.....(((((((......((((((.....)))))).(((((((((..((((..((((...........)))).)))))..(((...(((((....)))))..))).....)))))))))..((..((((((.......))))
).))..)).))))))......)))))))))))).))))))))))))).......((((.....)))).................)))))))....).)))....))))))).....)))).))))))).))))).).)).)))))...))))))))))))).)))))))))))))
Degradome supported cutsite(s) in premiRNA:6/620
miRNA sequence and Count(AGO1/wt):UAGACCAUUUGUGAGAAGGGA_746.34/1807.47
miRNA Position in PremiRNA:6-26
miRNA* sequence and Count(AGO1/wt):CCUUCUCAUCGAUGGUCUAGA_9.04/123.76
miRNA Position in PremiRNA:599-619
miRNA annonation:ath-miR824

34. Degradome supported cutsite(s) in Genome:15074945
PremiRNA Locus in Genome:>Chr4(15074940-15075033)_plus strand
PremiRNA
sequence:AAAACUGACAGAAGAGAGUGAGCACAUGCAGGCACUGUUAUGUGUCUAUAACUUUGCGUGUGCGUGCUCACCUCUCUUUCUGUCAGUUGCC
UAU
PremiRNA secondary structure:(.((((((((((((((((((((((..(((.(((..((((((....))))))..))).))).)))))))).))))).)))))))))))).....)
Degradome supported cutsite(s) in premiRNA:6
miRNA sequence and Count(AGO1/wt):UGACAGAAGAGAGUGAGCAC_46.38/51.23
miRNA Position in PremiRNA:6-25
miRNA* sequence and Count(AGO1/wt):GCUCACCUCUCUUUCUGUCAGU_4.72/38.57
miRNA Position in PremiRNA:66-87
miRNA annonation:ath-miR156e/ath-miR156f/ath-miR156d/ath-miR156a/ath-miR156c/ath-miR156b

35. Degradome supported cutsite(s) in Genome:287586
PremiRNA Locus in Genome:>Chr5(287581-287739)_plus strand
PremiRNA
sequence:CAAGAUGGAGAAGCAGGGCACGUGCAUUACUAGCUCAUAUAUACACUCUCACCACAAAUGCGUGUAUAUAUGCGGAAUUUUGUGAUAUAGA
UGUGUGUGUGUGUUGAGUGUGAUGAUAUGGAUGAGUUAGUUCUUCAUGUGCCCAUCUUCACCAUCAUG
PremiRNA secondary structure:((.(((((.((((..(((((((((....((((((((((.(((((((.((((.((((.((((((.(((((.(((((....)))))))))))).))))))...)))).)))).))).....)))).))))))))))....))))))))..)))).))))).))

Degradome supported cutsite(s) in premiRNA:6
miRNA sequence and Count(AGO1/wt):UGGAGAAGCAGGGCACGUGCA_400.49/85.19
miRNA Position in PremiRNA:6-26

miRNA* sequence and Count(AGO1/wt):AUGUGCCCAUCUUCACCAUC_0.39/0.58
miRNA Position in PremiRNA:140-156
miRNA annonation:ath-miR164b/ath-miR164a

36. Degradome supported cutsite(s) in Genome:3867213
PremiRNA Locus in Genome:>Chr5(3867208-3867312)_plus strand
PremiRNA sequence:GGAGGUGACAGAAGAGAGUGAGCACACAUGGUGGUUUCUUGCAUGCUUUUUUGAUUAGGGUUUCAUGCUUGAAGCUAUGUGUGCUUACUCUCUCUCUGUCACCCC
PremiRNA secondary structure:((.(((((((((((((((((((((((((((((...(((..(((((((((.......))))...)))))..))))))))))))))))))))))).)))))))))))
Degradome supported cutsite(s) in premiRNA:6
miRNA sequence and Count(AGO1/wt):UGACAGAAGAGAGUGAGCAC_46.38/51.23
miRNA Position in PremiRNA:6-25
miRNA* sequence and Count(AGO1/wt):GCUUACUCUCUCUCUGUCACC_0/0.58
miRNA Position in PremiRNA:83-103
miRNA annonation:ath-miR156e/ath-miR156f/ath-miR156d/ath-miR156a/ath-miR156c/ath-miR156b

37. Degradome supported cutsite(s) in Genome:4691128
PremiRNA Locus in Genome:>Chr5(4691027-4691132)_plus strand
PremiRNA sequence:CUCGACAGGGUUGAUAUGAGAACACACGAGUAAUCAACGGCUGUAAUGACGCUACGUCAUUGUUACAGCUCUCGUUUUCAUGUGUUCUCAGGUCACCCCUGCUGAG
PremiRNA secondary structure:((((.(((((.((((.((((((((((.(((.(((....(((((((((((((...))))))...)))))))...)))))).)))))))))).)))).))))).))))
Degradome supported cutsite(s) in premiRNA:102
miRNA sequence and Count(AGO1/wt):UGUGUUCUCAGGUCACCCCUG_1.97/0
miRNA Position in PremiRNA:81-101
miRNA* sequence and Count(AGO1/wt):GGGUUGAUAUGAGAACACACG_0.79/0.86
miRNA Position in PremiRNA:8-28
miRNA annonation:ath-miR398c/ath-miR398b

38. Degradome supported cutsite(s) in Genome:9098879/9098897
PremiRNA Locus in Genome:>Chr5(9098806-9098901)_plus strand

PremiRNA sequence:UCAAUAUAUAUAGUCCAAUCUAUUGAAGUACUAGUACACCAGCUCUAAUAAGCUGAUGUGGGUAAGUAGUUCAAUAGAUUGGACUAUGUAUAUUAA

PremiRNA secondary structure:(.(((((((((((((((((((((((((.((((.(..((((((((......)))))..)))..).)))).))))))))))))))))))))))))).)

Degradome supported cutsite(s) in premiRNA:74/92

miRNA sequence and Count(AGO1/wt):UCAAUAGAUUGGACUAUGUAU_19.65/16.69

miRNA Position in PremiRNA:71-91

miRNA* sequence and Count(AGO1/wt):AUAUAGUCCAAUCUAUUGAAG_0/0

miRNA Position in PremiRNA:8-28

miRNA annonation:ath-miR860

39. Degradome supported cutsite(s) in Genome:9136126

PremiRNA Locus in Genome:>Chr5(9136121-9136223)_plus strand

PremiRNA sequence:GAUGGUGACAGAAGAGAGUGAGCACACAUGGUGGCUUUCUUGCAUAUUUGAAGGUUCCAUGCUUGAAGCUAUGUGUGCUCACUCUCUAUCCGUCACCCCCUUC

PremiRNA secondary structure:((.((((((.((((((((((((((((((((((....(((..((((............))))..))))))))))))))))))))))).)).))))))....))

Degradome supported cutsite(s) in premiRNA:6

miRNA sequence and Count(AGO1/wt):UGACAGAAGAGAGUGAGCAC_46.38/51.23

miRNA Position in PremiRNA:6-25

miRNA* sequence and Count(AGO1/wt):GCUCACUCUCUAUCCGUCACC_0/0.58

miRNA Position in PremiRNA:77-97

miRNA annonation:ath-miR156e/ath-miR156f/ath-miR156d/ath-miR156a/ath-miR156c/ath-miR156b

40. Degradome supported cutsite(s) in Genome:9852684

PremiRNA Locus in Genome:>Chr5(9852679-9852770)_plus strand

PremiRNA sequence:CUUGAUGGAGAAGCAGGGCACGUGCGAACACAAAUGAAAUCGAUCGGUACUUGUUGAUCAUAUUUUCGCACGUGUUCUACUACUCCAACACG

PremiRNA secondary structure:(.((.(((((.((.((((((((((((((..(....).....((((((......)))))).....)))))))))))))).)).))))).)).)

Degradome supported cutsite(s) in premiRNA:6

miRNA sequence and Count(AGO1/wt):UGGAGAAGCAGGGCACGUGCG_4.72/1.15

miRNA Position in PremiRNA:6-26

miRNA* sequence and Count(AGO1/wt):CACGUGUUCUACUACUCCAAC_0/3.45

miRNA Position in PremiRNA:69-89
miRNA annonation:ath-miR164c

41. Degradome supported cutsite(s) in Genome:13611904
PremiRNA Locus in Genome:>Chr5(13611803-13611927)_plus strand
PremiRNA sequence:UACUUUUCCACAGCUUUCUUGAACUUUCUUUUUCAUUUCCAUUGUUUUUUUCUUAAACAAAAGUAAGAAGAAAAAAAACUUUAAGAUUAAGCAUUUUGGAAGCUCAAGAAAGCUGUGGGAAAACA
PremiRNA secondary structure:(..(((((((((((((((((((.(((((................(((((((((((........)))))))))))....(((.......)))......))))).)))))))))))))))))))..)
Degradome supported cutsite(s) in premiRNA:102
miRNA sequence and Count(AGO1/wt):UUCCACAGCUUUCUUGAACUU_2035.84/1539.8
miRNA Position in PremiRNA:6-26
miRNA* sequence and Count(AGO1/wt):GCUCAAGAAAGCUGUGGGAAA_41.27/28.49
miRNA Position in PremiRNA:102-122
miRNA annonation:ath-miR396b

42. Degradome supported cutsite(s) in Genome:17516378/17516399
PremiRNA Locus in Genome:>Chr5(17516303-17516403)_plus strand
PremiRNA sequence:GUUCAGGUGAAUGAUGCCUGGCUCGAGACCAUUCAAUCUCAUGAUCUCAUGAUUAUAACGAUGAUGAUGAUGAUGUCGGACCAGGCUUCAUUCCCCUCAAC
PremiRNA secondary structure:(((.(((.((((((.((((((..(((.(.((((..(((((((((((....))))))...)).)))...)))).).)))..)))))).)))))).))).)))
Degradome supported cutsite(s) in premiRNA:76/97
miRNA sequence and Count(AGO1/wt):UCGGACCAGGCUUCAUUCCCC_14661.96/1228.68
miRNA Position in PremiRNA:76-96
miRNA* sequence and Count(AGO1/wt):UGAAUGAUGCCUGGCUCGAGA_0.39/0
miRNA Position in PremiRNA:8-28
miRNA annonation:ath-miR166b/ath-miR166f/ath-miR166a/ath-miR166g/ath-miR166c/ath-miR166d/ath-miR166e

43. Degradome supported cutsite(s) in Genome:21161609
PremiRNA Locus in Genome:>Chr5(21161504-21161634)_plus strand
PremiRNA sequence:AUGAAUUGUCAGUAUAAAUCUUUGAUCUGAUACUCUAUUAGUUAGUAUGUGAAUUUCUUUUUAACAAUUUUCUUAUUCAUGCGAGAGCUAUCAAUAAGUAUUAGAACAAAGUUUUAUACUGACAAUUCAAU

PremiRNA secondary structure:((((((((((((((((((.(((((.(((((((((....(((((.(((((.(((...............))).....)))))...)))))......))))))))).))))).))))))))))))))))).))
Degradome supported cutsite(s) in premiRNA:106
miRNA sequence and Count(AGO1/wt):ACAAAGUUUUAUACUGACAAU_4.72/33.1
miRNA Position in PremiRNA:106-126
miRNA* sequence and Count(AGO1/wt):UGUCAGUAUAAAUCUUUGAUC_6.68/3.45
miRNA Position in PremiRNA:7-27
miRNA annonation:ath-miR4245

44. Degradome supported cutsite(s) in Genome:23636961
PremiRNA Locus in Genome:>Chr5(23636956-23637057)_plus strand
PremiRNA
sequence:CUAUAAAGCUCAGGAGGGAUAGCGCCAUGGCUCACCAGUGCUGUAUGUUUUGUAUAUGCGUACAUGUAUAUCUGUUGGCGCUAUCCAUCCU
GAGUUCCAUAG
PremiRNA secondary structure:((((..(((((((((.(((((((((((.((....)).(((((((((((.........))))))).))))......))))))))))).)))))))))..))))
Degradome supported cutsite(s) in premiRNA:6
miRNA sequence and Count(AGO1/wt):AAGCUCAGGAGGGAUAGCGCC_121.05/98.14
miRNA Position in PremiRNA:6-26
miRNA* sequence and Count(AGO1/wt):CGCUAUCCAUCCUGAGUUCCA_0.39/2.01
miRNA Position in PremiRNA:79-99
miRNA annonation:ath-miR390b/ath-miR390a

45. Degradome supported cutsite(s) in Genome:78932/78953
PremiRNA Locus in Genome:>Chr1(79037-78927)_minus strand
PremiRNA
sequence:GUUGAGGGGAAUGUUGUCUGGAUCGAGGAUAUUAUAGAUAUAUACAUGUGUAUGUUAAUGAUUCAAGUGAUCAUAGAGAGUAUCCUCGGA
CCAGGCUUCAUCCCCCCCAAC
PremiRNA secondary structure:((((.((((.(((..((((((.(((((((((((...((((((((....)))))))).((((((.....))))))....))))))))))).))))))..))).)))).))))
Degradome supported cutsite(s) in premiRNA:107/86
miRNA sequence and Count(AGO1/wt):UCGGACCAGGCUUCAUCCCCC_6791.36/474.6
miRNA Position in PremiRNA:86-106
miRNA* sequence and Count(AGO1/wt):GGAAUGUUGUCUGGAUCGAGG_1.97/56.41
miRNA Position in PremiRNA:8-28
miRNA annonation:ath-miR165b/ath-miR165a

46. Degradome supported cutsite(s) in Genome:234017
PremiRNA Locus in Genome:>Chr1(234152-234013)_minus strand
PremiRNA
sequence:AUGAUGCGCAAAUGCGGAUAUCAAUGUAAAUCAGGACAACAAACCAAUAGAGGUGCUCAGCGAAAACAACAAAAUGUGAAAAGGGUUUAUC
AGAAACAUUGGGGUCAUGAUAUACCUUUAUAUCCGCAUUUGCGCAUCAU
PremiRNA secondary structure:((((((((((((((((((((.((.(((.((((.(((..(((.......((...((((.......(((......))).....))))...)).......)))..))).)))).))).)).))))))))))))))))))))
Degradome supported cutsite(s) in premiRNA:137
miRNA sequence and Count(AGO1/wt):CUUUAUAUCCGCAUUUGCGCA_20.44/11.8
miRNA Position in PremiRNA:116-136
miRNA* sequence and Count(AGO1/wt):CGCAAAUGCGGAUAUCAAUGU_0/1.44
miRNA Position in PremiRNA:7-27
miRNA annonation:ath-miR2112-3p

47. Degradome supported cutsite(s) in Genome:3961367/3961388
PremiRNA Locus in Genome:>Chr1(3961454-3961365)_minus strand
PremiRNA
sequence:CGCGAGAUAUUAGUGCGGUUCAAUCAAAUAGUCGUCCUCUUAACUCAUGGAGAACGGUGUUGUUCGAUUGAGCCGUGCCAAUAUCACGCG

PremiRNA secondary structure:((((.((((((.(..((((((((((.(((((((((.((((........)))).)))))..)))).))))))))))..).)))))).))))
Degradome supported cutsite(s) in premiRNA:89/68
miRNA sequence and Count(AGO1/wt):UUGAGCCGUGCCAAUAUCACG_148.17/37.13
miRNA Position in PremiRNA:68-88
miRNA* sequence and Count(AGO1/wt):AGAUAUUAGUGCGGUUCAAUC_1.57/6.91
miRNA Position in PremiRNA:5-25
miRNA annonation:ath-miR171c/ath-miR171b

48. Degradome supported cutsite(s) in Genome:4820431
PremiRNA Locus in Genome:>Chr1(4820499-4820405)_minus strand
PremiRNA
sequence:UCUCCUCUUCUCCAAAUAGUUUAGGUUAGCUGACAUAUAAUAAUUCAAUAGACCCCAGUUUCUCCAGAUAACCUAACUAUUUGAGAAGAA
GUGA
PremiRNA secondary structure:((.(.(((((((.((((((((.((((((.(((............((....))............))).))))))))))))))).))))))).).))
Degradome supported cutsite(s) in premiRNA:70
miRNA sequence and Count(AGO1/wt):UCUUCUCCAAAUAGUUUAGGUU_2.75/3.45

miRNA Position in PremiRNA:6-27
miRNA* sequence and Count(AGO1/wt):CCUAACUAUUUGAGAAGAAG_0.39/1.44
miRNA Position in PremiRNA:72-92
miRNA annonation:ath-miR830-5p

49. Degradome supported cutsite(s) in Genome:11834118
PremiRNA Locus in Genome:>Chr1(11834159-11834089)_minus strand
PremiRNA sequence:AUUUGAACCUGUCAAUUGGUAUCAAAGCUUCUAAAUCUUUGAAGCUCUGAUACCAAAUGAUGGAAUCAAAU

PremiRNA secondary structure:((((((..((((((.(((((((((.((((((.........)))))).))))))))).))))))..))))))
Degradome supported cutsite(s) in premiRNA:43
miRNA sequence and Count(AGO1/wt):AGCUCUGAUACCAAAUGAUGGAAU_38.91/94.12
miRNA Position in PremiRNA:43-66
miRNA* sequence and Count(AGO1/wt):CCUGUCAAUUGGUAUCAAAGCUUC_1.57/16.69
miRNA Position in PremiRNA:8-31
miRNA annonation:ath-miR829.1

50. Degradome supported cutsite(s) in Genome:23345389/23345408
PremiRNA Locus in Genome:>Chr1(23345506-23345382)_minus strand
PremiRNA
sequence:AGUUUUAGGGCGCCUCUCCAUUGGCAGGUCCUUUACUUCCAAAUAUACACAUACAUAUAUGAAUAUCGAAAAUUUCCGAUGAUCGAUUUAU
AAAUGACCUGCCAAAGGAGAGUUGCCCUGAAACU
PremiRNA secondary structure:((((((((((((.((((((.((((((((((.((((.......(((((........)))))((.(((((........))))).))......)))).)))))))))).)))))).))))))))))))
Degradome supported cutsite(s) in premiRNA:119/100
miRNA sequence and Count(AGO1/wt):UGCCAAAGGAGAGUUGCCCUG_36.16/4.61
miRNA Position in PremiRNA:100-120
miRNA* sequence and Count(AGO1/wt):UUAGGGCGCCUCUCCAUUGGC_0/0
miRNA Position in PremiRNA:8-28
miRNA annonation:ath-miR399b/ath-miR399c

51. Degradome supported cutsite(s) in Genome:27713234/27713255
PremiRNA Locus in Genome:>Chr1(27713416-27713233)_minus strand
PremiRNA
sequence:GUAGAGCUCCUUAAAGUUCAAACAUGAGUUGAGCAGGGUAAAGAAAAGCUGCUAAGCUAUGGAUCCCAUAAGCCCUAAUCCUUGUAAAGUA

AAAAAGGAUUUGGUUAUAUGGAUUGCAUAUCUCAGGAGCUUUAACUUGCCCUUUAAUGGCUUUUACUCUUCUUUGGAUUGAAGGGAGCUCUAC

PremiRNA secondary structure:(((((((((((..((((((((...((((.(((((((((((...((((((.((.((.((((.(..(((((((((..(((((((..........))))))).)))).)))))..).)))).)).)).))))))...)))))))......))))..))))...))))))))..)))))))))))

Degradome supported cutsite(s) in premiRNA:184/163
miRNA sequence and Count(AGO1/wt):UUUGGAUUGAAGGGAGCUCUA_18349.26/2550.03
miRNA Position in PremiRNA:163-183
miRNA* sequence and Count(AGO1/wt):GAGCUCCUUAAAGUUCAAACA_0.79/0.86
miRNA Position in PremiRNA:4-24
miRNA annonation:ath-miR159a

52. Degradome supported cutsite(s) in Genome:22930095/22930116
PremiRNA Locus in Genome:>Chr1(22930184-22930090)_minus strand
PremiRNA sequence:AAAUACGAGAUAUUGGUGCGGUUCAAUCAGAAAACCGUACUCUUUUGUUUUAAAGAUCGGUUUAUUUGAUUGAGCCGUGCCAAUAUCACGCGUUU
PremiRNA secondary structure:(((..((.((((((((..((((((((((((((((((...(((((......))))).)))))).))))))))))))..)))))))).))..)))
Degradome supported cutsite(s) in premiRNA:91/70
miRNA sequence and Count(AGO1/wt):UUGAGCCGUGCCAAUAUCACG_148.17/37.13
miRNA Position in PremiRNA:70-90
miRNA* sequence and Count(AGO1/wt):AGAUAUUGGUGCGGUUCAAUC_16.51/37.13
miRNA Position in PremiRNA:8-28
miRNA annonation:ath-miR171c/ath-miR171b

53. Degradome supported cutsite(s) in Genome:4142352/4142464
PremiRNA Locus in Genome:>Chr2(4142468-4142328)_minus strand
PremiRNA sequence:UAUUCUUCCACAGCUUUCUUGAACUGCAAAACUUCUUCAGAUUUUUUUUUUUUCUUUUGAUAUCUCUUACGCAUAAAAUAGUGAUUUUCUUCAUAUCUCUGCUCGAUUGAUUUGCGGUUCAAUAAAGCUGUGGGAAGAUA
PremiRNA secondary structure:(((..(((((((((((.((((((((((((.(.((..((((...........((....))..................((((......))))...))))...))..).))))))))))))).))))))))))))..)))
Degradome supported cutsite(s) in premiRNA:118/6
miRNA sequence and Count(AGO1/wt):UUCCACAGCUUUCUUGAACUG_2063.35/773.07
miRNA Position in PremiRNA:6-26

miRNA* sequence and Count(AGO1/wt):GUUCAAUAAAGCUGUGGGAAG_119.48/140.74
miRNA Position in PremiRNA:118-138
miRNA annonation:ath-miR396a

54. Degradome supported cutsite(s) in Genome:10676554
PremiRNA Locus in Genome:>Chr2(10676558-10676443)_minus strand
PremiRNA
sequence:GAAACUGACAGAAGAGAGUGAGCACACAAAGGCAAUUUGCAUAUCAUUGCACUUGCUUCUCUUGCGUGCUCACUGCUCUUUCUGUCAGAUU
CCGGUGCUGAUCUCUUUGGCCUGUC
PremiRNA secondary structure:((..((((((((((((((((((((.(((((((((..((((......)))).))))))...))).)))))))))).))).)))))))))....(((.((..(.....)..)))))))
Degradome supported cutsite(s) in premiRNA:6
miRNA sequence and Count(AGO1/wt):UGACAGAAGAGAGUGAGCAC_46.38/51.23
miRNA Position in PremiRNA:6-25
miRNA* sequence and Count(AGO1/wt):GCUCACUGCUCUUUCUGUCAGA_0/3.45
miRNA Position in PremiRNA:68-89
miRNA annonation:ath-miR156e/ath-miR156f/ath-miR156d/ath-miR156a/ath-miR156c/ath-miR156b

55. Degradome supported cutsite(s) in Genome:3366369
PremiRNA Locus in Genome:>Chr3(3366416-3366349)_minus strand
PremiRNA sequence:UGCUUCUUUGUCUACAAUUUUGGAAAAAGUGAUGACGCCAUUGCUCUUUCCCAAAUGUAGACAAAGCA
PremiRNA secondary structure:(((((...((((((((.(((.(((((.((..(((....)))..)).))))).))))))))))))))))
Degradome supported cutsite(s) in premiRNA:49
miRNA sequence and Count(AGO1/wt):UCCCAAAUGUAGACAAAGCA_63787.33/11683.21
miRNA Position in PremiRNA:49-68
miRNA* sequence and Count(AGO1/wt):CUUUGUCUACAAUUUUGGAAA_12.97/67.06
miRNA Position in PremiRNA:3-26
miRNA annonation:ath-miR158a

56. Degradome supported cutsite(s) in Genome:3599812
PremiRNA Locus in Genome:>Chr3(3599897-3599787)_minus strand
PremiRNA
sequence:GCUGUUGGAGCAUCAUCAAGAUUCACAAAUCAUCAAGUAUUCGUGUAAAUAAACCCAUUUAUGAUUAGAUUUUUGAUGUAUGUAUGAGAAU
CUUGAUGAUGCUGCAGCUGC
PremiRNA secondary structure:((.((((.(((((((((((((((.((.(((((((((...((.(((((((......)))))))....))..))))))).))...)).))))))))))))))).)))).))

Degradome supported cutsite(s) in premiRNA:87
miRNA sequence and Count(AGO1/wt):AGAAUCUUGAUGAUGCUGCAG_863.46/562.68
miRNA Position in PremiRNA:87-107
miRNA* sequence and Count(AGO1/wt):GGAGCAUCAUCAAGAUUCACA_0/3.17
miRNA Position in PremiRNA:7-27
miRNA annonation:ath-miR172d/ath-miR172c

57. Degradome supported cutsite(s) in Genome:4359010
PremiRNA Locus in Genome:>Chr3(4359207-4359007)_minus strand
PremiRNA
sequence:GUGUGCAGCCAAGGAUGACUUGCCGAUUUAAAUGAUCUUUCUUUAUACUCUAUUAAGACAAUUUAGUUUCAAACUUUUUUUUUUUUUUUUUUUGAAGGAUUCAGGAAGAAAUUAGGAUAUAUUAUUCCGUAUAAAAUACAAGAUAUAUAAAACCAAAAAGAAAAAGUAACAUGAUCGGCAAGUUGUCCUUGGCUACACGU
PremiRNA secondary
structure:(((((.(((((((((..((((((((((((((((..((((...............))))..))))).......(((((((((((((((((((((((((.....)))))))))))...(((.........)))..........................))))))))))))).....))))))))))..))))))))).)))))

Degradome supported cutsite(s) in premiRNA:199
miRNA sequence and Count(AGO1/wt):CAGCCAAGGAUGACUUGCCGA_92.36/225.36
miRNA Position in PremiRNA:6-26
miRNA* sequence and Count(AGO1/wt):CAAGUUGUCCUUGGCUACACG_2.75/2.88
miRNA Position in PremiRNA:178-198
miRNA annonation:ath-miR169a

58. Degradome supported cutsite(s) in Genome:6244694
PremiRNA Locus in Genome:>Chr3(6244698-6244524)_minus strand
PremiRNA
sequence:GGUUGUUGACAGAAGAUAGAGAGCACUAAGGAUGACAUGCAAGUACAUACAUAUAUAUCAUCACACCGCAUGUGGAUGAUAAAAUAUGUAUAACAAAUUCAAAGAAAGAGAGGGAGAGAAAGAGAGAGAACCUGCAUCUCUACUCUUUUGUGCUCUCUAUACUUCUGUCACCACC

PremiRNA secondary structure:(((.(((.(((((((((((((((((((...((((.((......))..((((((((.(((((((.(((.....))))))))))..))))))))).....))))...(((((((..(((((...((.(......)))...))))).))))))))))))))))))).))))))))))).)))

Degradome supported cutsite(s) in premiRNA:6
miRNA sequence and Count(AGO1/wt):UUGACAGAAGAUAGAGAGCAC_3.14/2.59
miRNA Position in PremiRNA:6-26

miRNA* sequence and Count(AGO1/wt):GCUCUCUAUACUUCUGUCACC_0.39/0
miRNA Position in PremiRNA:152-172
miRNA annonation:ath-miR157b/ath-miR157a/ath-miR157c

59. Degradome supported cutsite(s) in Genome:19659579
PremiRNA Locus in Genome:>Chr3(19659671-19659576)_minus strand
PremiRNA
sequence:UGUCGUCUCGGUUCGCGAUCCACAAGUAAUCUUUUGUGGAGAUUAUGAAAACAAUCAUCACGAGAGGCUACGUGUGGGUGGCAAACAAAGA
CGACA
PremiRNA secondary structure:((((((((..(((.((.((((((((.(((..((((((((..((((.(....).))))..))))))))..))).))))))).)).)))..))))))))
Degradome supported cutsite(s) in premiRNA:94
miRNA sequence and Count(AGO1/wt):UCUCGGUUCGCGAUCCACAAG_1.18/0
miRNA Position in PremiRNA:6-26
miRNA* sequence and Count(AGO1/wt):UGUGGGUGGCAAACAAAGACG_0/0
miRNA Position in PremiRNA:73-93
miRNA annonation:ath-miR851-5p

60. Degradome supported cutsite(s) in Genome:369856/369877
PremiRNA Locus in Genome:>Chr4(369996-369851)_minus strand
PremiRNA
sequence:GUUGUGGGGAAUGUUGUUUGGAUCGAGGAUAUCAUAAACGCAUACACAUGUUUAUAUGUUAUGAUGCAUUAUAUGACUGAUGUAAUGUAC
AUAUAUAUACAUACAUGCCACAUGGUAUCGUCGGACCAGGCUUCAUCCCCCUCAAC
PremiRNA secondary structure:((((.((((.(((..((((((.((((.((((((((....((((....((((..((((((.(((.((((((((((.....)))))))))))))))))))))))..))))...)))))))).)))).))))))..))).)))).))))

Degradome supported cutsite(s) in premiRNA:142/121
miRNA sequence and Count(AGO1/wt):UCGGACCAGGCUUCAUCCCCC_6791.36/474.6
miRNA Position in PremiRNA:121-141
miRNA* sequence and Count(AGO1/wt):GGAAUGUUGUUUGGAUCGAGG_0/2.88
miRNA Position in PremiRNA:8-28
miRNA annonation:ath-miR165b/ath-miR165a

61. Degradome supported cutsite(s) in Genome:1535440
PremiRNA Locus in Genome:>Chr4(1535652-1535435)_minus strand

PremiRNA
sequence:UAUACAAUACUACUUUUUCAUCCAUUAAUCCCCUUACAAUGUCGAGUAAACGAAGCAUCUGUCCCCUGGUAUUGUCUUCGAGCUUGGUGUG
UUUUUCUAGCCAGCCCCAAGUUCUCGAGUUGAUCAUUGUUUGUAUUCUGACACAUUAUUUGGGGACGAGAUGUUUUGUUGACUCGAUAUAAGAAGG
GGCUUUAUGGAAGAAAUUGUAGUAUUAUAUA
PremiRNA secondary
structure:((((.((((((((..((((.(((((.((.((((((...((((((((.((((((((((((((((..(((.((((...(((((((.(((((.....)))..)).)))))))))..((((....((.....)).)))).))))...)))..)))))).))))))))))).)))))))))...)))))).)).))))).))))..))))
)))).)))))
Degradome supported cutsite(s) in premiRNA:214
miRNA sequence and Count(AGO1/wt):UAUGGAAGAAAUUGUAGUAUU_269.22/96.13
miRNA Position in PremiRNA:193-213
miRNA* sequence and Count(AGO1/wt):UACUACUUUUUCAUCCAUUAA_0/0
miRNA Position in PremiRNA:8-28
miRNA annonation:ath-miR447a.2-3p

62. Degradome supported cutsite(s) in Genome:8504145/8504169
PremiRNA Locus in Genome:>Chr4(8504315-8504140)_minus strand
PremiRNA
sequence:UCAAGAUAUCAGAUAUUUCACGAAGAUAUCUGCAUAACAGCUACGAGGAAAUUGUGAUUUUAUCGUUUCACAAACAUCAACAGCUCCAUGG
AUCGUUAUGGACGGAUAAAUCACAAUCACUAUCCUUUUUCUAGCAGCUGUUGAGCAGGUUUCUUCGUGAAUAUCUGGCAUUUUGG

PremiRNA secondary structure:(((((((..((((((((.((((((((.((((((.((((((((.((((((.((((((((((.((((((.....)))).......(.((((((((....)))))))).))))))))))))).....))))).......).)))))))).))))))).))))))))))))))))..)))))))

Degradome supported cutsite(s) in premiRNA:172/148
miRNA sequence and Count(AGO1/wt):UUCUUCGUGAAUAUCUGGCAU_4.32/2.59
miRNA Position in PremiRNA:151-171
miRNA* sequence and Count(AGO1/wt):UAUCAGAUAUUUCACGAAGAUA_0.39/0.29
miRNA Position in PremiRNA:8-29
miRNA annonation:ath-miR780.2

63. Degradome supported cutsite(s) in Genome:15415511
PremiRNA Locus in Genome:>Chr4(15415511-15415427)_minus strand
PremiRNA sequence:CUGACAGAAGAGAGUGAGCACACAAAGGCACUUUGCAUGUUCGAUGCAUUUGCUUCUCUUGCGUGCUCACUGCUCUAUCUGUCAG

PremiRNA secondary structure:((((((((((((((((((((.((((((((...(((((.....)))))..)))))...))).))))))))).)))).))))))))

Degradome supported cutsite(s) in premiRNA:2
miRNA sequence and Count(AGO1/wt):UGACAGAAGAGAGUGAGCAC_46.38/51.23
miRNA Position in PremiRNA:2-21
miRNA* sequence and Count(AGO1/wt):GCUCACUGCUCUAUCUGUCAGA_0/2.59
miRNA Position in PremiRNA:65-86
miRNA annonation:ath-miR156e/ath-miR156f/ath-miR156d/ath-miR156a/ath-miR156c/ath-miR156b

64. Degradome supported cutsite(s) in Genome:1188211
PremiRNA Locus in Genome:>Chr5(1188304-1188206)_minus strand
PremiRNA
sequence:UGUAGGCGCAGCACCAUUAAGAUUCACAUGGAAAUUGAUAAAUACCCUAAAUUAGGGUUUUGAUAUGUAUAUGAGAAUCUUGAUGAUGCUG
CAUCAACA
PremiRNA secondary structure:(((.(..((((((.(((((((((((.((((...((...((((.((((((...)))))))))))..))...)))).)))))))))))).))))))..).)))
Degradome supported cutsite(s) in premiRNA:95
miRNA sequence and Count(AGO1/wt):AGAAUCUUGAUGAUGCUGCAU_540.01/1466.7
miRNA Position in PremiRNA:74-94
miRNA* sequence and Count(AGO1/wt):GCAGCACCAUUAAGAUUCACA_1.57/7.77
miRNA Position in PremiRNA:8-28
miRNA annonation:ath-miR172a/ath-miR172b-3p

65. Degradome supported cutsite(s) in Genome:2634936/2634957
PremiRNA Locus in Genome:>Chr5(2635022-2634934)_minus strand
PremiRNA
sequence:CGCUGGAGGCAGCGGUUCAUCGAUCUCUUCCUGUGAACACAUUAAAAAUGUAAAAGCAUGAAUAGAUCGAUAAACCUCUGCAUCCAGCG

PremiRNA secondary structure:(((((((.((((.((((.((((((((.(((.(((....((((.....))))....))).))).)))))))).)))).)))).)))))))
Degradome supported cutsite(s) in premiRNA:88/67
miRNA sequence and Count(AGO1/wt):UCGAUAAACCUCUGCAUCCAG_996.7/219.89
miRNA Position in PremiRNA:67-87
miRNA* sequence and Count(AGO1/wt):GGAGGCAGCGGUUCAUCGAUC_0/0
miRNA Position in PremiRNA:5-25
miRNA annonation:ath-miR162b/ath-miR162a

66. Degradome supported cutsite(s) in Genome:3456666/3456733

PremiRNA Locus in Genome:>Chr5(3456737-3456640)_minus strand
PremiRNA sequence:GAAGUUGACAGAAGAGAGUGAGCACACAAAGGGGAAGUUGUAUAAAAGUUUUGUAUAUGGUUGCUUUUGCGUGCUCACUCUCUUUUUGUCAUAACUUC
PremiRNA secondary structure:(((((((((((((((((((((((((.((((((.((...(((((((.....)))))))...)).)))))).))))))))))))).))))))))..)))))
Degradome supported cutsite(s) in premiRNA:73/6
miRNA sequence and Count(AGO1/wt):UGACAGAAGAGAGUGAGCAC_46.38/51.23
miRNA Position in PremiRNA:6-25
miRNA* sequence and Count(AGO1/wt):GCUCACUCUCUUUUUGUCAUA_0/2.01
miRNA Position in PremiRNA:73-93
miRNA annonation:ath-miR156e/ath-miR156f/ath-miR156d/ath-miR156a/ath-miR156c/ath-miR156b

67. Degradome supported cutsite(s) in Genome:7740610/7740631
PremiRNA Locus in Genome:>Chr5(7740705-7740605)_minus strand
PremiRNA sequence:UGAAGUCGCUGGAGGCAGCGGUUCAUCGAUCAAUUCCUGUGAAUAUUUAUUUUUGUUUACAAAAGCAAGAAUCGAUCGAUAAACCUCUGCAUCCAGCGCUG
PremiRNA secondary structure:(..((.(((((((.((((.((((.(((((((.((((.((((((((........))))))))......)))).))))))).)))).)))).))))))))))
Degradome supported cutsite(s) in premiRNA:97/76
miRNA sequence and Count(AGO1/wt):UCGAUAAACCUCUGCAUCCAG_996.7/219.89
miRNA Position in PremiRNA:76-96
miRNA* sequence and Count(AGO1/wt):GGAGGCAGCGGUUCAUCGAUC_0/0
miRNA Position in PremiRNA:11-31
miRNA annonation:ath-miR162b/ath-miR162a

68. Degradome supported cutsite(s) in Genome:16660490
PremiRNA Locus in Genome:>Chr5(16660998-16660464)_minus strand
PremiRNA sequence:AAAAGCCAUAACUCCUUCAUUUUCUUUAGACAUCUCUUCUUCUCUCAUCUCUCUUUUCUUCUCUCUUUUCCUCACAUAAACUCUCUUUUUUUACUAUUAAAUCCAUAUGGUACCUCAAAUUAAUCUAUGGUCAUCUAGGGUUAUCUUGAAGAUUAGAAUUGAUUCUAGCACGCACAGAGAGGAAGAUCAUUGCAUCCAGAAUCACAAACAUGGCCUAUCUUUUAUCUUUUCUUUUUGAUCUAAGUCACUGUUUUAUGCUAUAUAUAGUAUAAUCAAAUUCUUUACAUGUGCUUGUAUGUAUGCGUAUAUAUAGUAACGGAAUUGUUAAUAUGCUUAUAGAUGUUGAGUUGGUGGAGGAAGAGAGCUUUCUUCGGUCCACUCAUGGAGUAAUAUGUGAGAUUUAAUUGACUCUCGACUCAUUCAUCCAAAUACCAAAUGAAAGAAUUUGUUCUCAUAUGGUAAAUGAAUGAAUGAUGCGAGAGACAAAUUGAGUCUUCACUUCUCUAUGCUUGGACUGAAGGGAGCUCCCUAUUUU

PremiRNA secondary
structure:((((........((((((((..(((..((((((((((((((((......((..((((..((...........................((((((.........))))))..((((..((((((.(((....)))))))))..)))).))..))))..)).....(....)..))))))))))..(((.((((((........((((((((..(((..
...............)))....)))).))))))..((((((((((((((((...........((((......))))..))))).))))))))))..)))..)))....))).....))))))))))..)))))))))...(((((..((((((((((..(((((((......((((((((((((..((((((..((((((((.((..(((((.((((..(((....)
))))))).)))))..)).))))))))..))))))...))))))))).))))..)))))))..))))))))))..)))))....))))
Degradome supported cutsite(s) in premiRNA:510
miRNA sequence and Count(AGO1/wt):UUGGACUGAAGGGAGCUCCCU_702.32/207.51
miRNA Position in PremiRNA:510-530
miRNA* sequence and Count(AGO1/wt):AGAGCUUUCUUCGGUCCACUC_3.54/12.09
miRNA Position in PremiRNA:361-381
miRNA annonation:ath-miR319b/ath-miR319a

69. Degradome supported cutsite(s) in Genome:16775545
PremiRNA Locus in Genome:>Chr5(16775662-16775520)_minus strand
PremiRNA
sequence:UUGAGGGGAAUGUUGUCUGGCACGAGGCCCUUAACUUAGAUCUAUAUUUGAUUAUAUAUAUAUGUCUCUUCUUUAUUCAUUAGUCUAUACA
UGAAUGAUCAUUUUACGGUUAAUGACGUCGGACCAGGCUUCAUUCCCCUCAA
PremiRNA secondary structure:((((((((((((..((((((..(((.(.(.((((((.((((.(((((..........))))).)))).....((((((((((.....)).))))))))).........)))))).).).)))..))))))..))))))))))))
Degradome supported cutsite(s) in premiRNA:119
miRNA sequence and Count(AGO1/wt):UCGGACCAGGCUUCAUUCCCC_14661.96/1228.68
miRNA Position in PremiRNA:119-139
miRNA* sequence and Count(AGO1/wt):GGAAUGUUGUCUGGCACGAGG_0/18.71
miRNA Position in PremiRNA:7-27
miRNA annonation:ath-miR166b/ath-miR166f/ath-miR166a/ath-miR166g/ath-miR166c/ath-miR166d/ath-miR166e

70. Degradome supported cutsite(s) in Genome:18358894
PremiRNA Locus in Genome:>SChr5(18358898-18358800)_minus strand
PremiRNA
sequence:CGGAUUCGCUUGGUGCAGGUCGGGAACUGAUUGGCUGACACCGACACGUGUCUUGUCAUGGUUGGUUUGUGAGCUCCCGUCUUGUAUCAAC
UGAAUCGG
PremiRNA secondary structure:(.((((((.((((((((((.(((((........(((.((((((((.((((......))))))))))..)).)))))))).)))))))))).)))))).)
Degradome supported cutsite(s) in premiRNA:6
miRNA sequence and Count(AGO1/wt):UCGCUUGGUGCAGGUCGGGAA_349.79/174.99
miRNA Position in PremiRNA:6-26
miRNA* sequence and Count(AGO1/wt):CCCGUCUUGUAUCAACUGAAU_0.79/89.51

miRNA Position in PremiRNA:76-96
miRNA annonation:ath-miR168b/ath-miR168a

71. Degradome supported cutsite(s) in Genome:771384/771406
PremiRNA Locus in Genome:>Chr2(771517-771382)_minus strand
PremiRNA sequence:GAAGACACUGAAGGACCUAAACUAACAAAGGUAAACGGCUCAGUGUGCGGGGUAUUACACUCGGUUUAAUGUCUGAAUGCGAUAAUCCGCACGAUGAUCUCUUUAUCUUUGUUUGUUUAGGUCCCUUAGUUUCUUC
PremiRNA secondary structure:(((((.(((((.((((((((((.((((((((((((.((.(((.(((((((..(((..((.((((........)))).))..)))..))))))).))).)).)))))))))))).)))))))))).))))).)))))
Degradome supported cutsite(s) in premiRNA:135/113
miRNA sequence and Count(AGO1/wt):CACUGAAGGACCUAAACUAAC_1.97/2.59
miRNA Position in PremiRNA:6-26
miRNA* sequence and Count(AGO1/wt):UUGUUUAGGUCCCUUAGUUUC_20.44/14.97
miRNA Position in PremiRNA:113-133
miRNA annonation:ath-miR840-3p

Supplementary table S2d. Discovery of known miRNAs from seedling of *Arabidopsis*.

1. Degradome supported cutsite(s) in Genome:28656
PremiRNA Locus in Genome:>Chr1(28545-28660)_plus strand
PremiRNA sequence:UGGUGUUGUGCAAGAAGGAGAAGCAAAGUCUGUCUAUGUAUUAUGAGAUAGCUACUUCUAUGGCUAGGAUAUAUGUUGUACAAGACCGGCUUUUCUUCUACUUCUUGCACAACCUG
PremiRNA secondary structure:(((.((((((((((((.(((((.((((((.((((.(((((((((...((((((......))))))...))))....))))))))).)))))).))))).)))))))))))))))
Degradome supported cutsite(s) in premiRNA:112
miRNA sequence and Count(AGO1/wt):UUUUCUUCUACUUCUUGCACA_32.65/7.58
miRNA Position in PremiRNA:91-111
miRNA* sequence and Count(AGO1/wt):UGCAAGAAGGAGAAGCAAAGU_0.62/0.55
miRNA Position in PremiRNA:9-29
miRNA annonation:ath-miR838

2. Degradome supported cutsite(s) in Genome:2165570
PremiRNA Locus in Genome:>Chr1(2165563-2165700)_plus strand
PremiRNA sequence:UCUUACAUCUUGAUGAAGAGGAAUGGGAAGCCGAAAUCUAAACUGAAACAGAGGCGUCACCCAAUUAUCAAUUGGUAAGUGAUAUCUAUGUUUCGAUCUGUAUCUCGGCCUUUCACUCCUCUUCUUCUUGAUGUAAGA
PremiRNA secondary structure:(((((((((..((.((((((((.((..(.(((((.........(((((((.((..((((((((((.....)))))...)))))..)).))))))).........))))).)..)).)))))))).))..)))))))))
Degradome supported cutsite(s) in premiRNA:8
miRNA sequence and Count(AGO1/wt):UCACUCCUCUUCUUCUUGAUG_60.95/5.92
miRNA Position in PremiRNA:113-133
miRNA* sequence and Count(AGO1/wt):UCUUGAUGAAGAGGAAUGGGA_9.3/0.74
miRNA Position in PremiRNA:8-28
miRNA annonation:ath-miR847

3. Degradome supported cutsite(s) in Genome:6220813/6220834
PremiRNA Locus in Genome:>Chr1(6220645-6220838)_plus strand
PremiRNA sequence:AGGAAGAGCUCCUUGAAGUUCAAUGGAGGGUUUAGCAGGGUGAAGUAAAGCUGCUAAGCUAUGGAUCCCAUAAGCCUUAUCAAAUUCAAUAUAAUUGAUGAUAAGGUUUUUUUUAUGGAUGCCAUAUCUCAGGAGCUUUCACUUACCCCUUUAAUGGCUUCACUCUUCUUUGGAUUGAAGGGAGCUCUUCAUCU

PremiRNA secondary structure:(((((((((((((..(((((((.(((((((..(((((((..((((((((((.((.((.(((((...((((((((((((((...(((((...))))))))))))))))).....))))))...))))).)).)).))))))).))))..))))......)))..))))))).)))))))..))))))))))))..))

Degradome supported cutsite(s) in premiRNA:169/190

miRNA sequence and Count(AGO1/wt):UUUGGAUUGAAGGGAGCUCUU_24839.19/3434.76

miRNA Position in PremiRNA:169-189

miRNA* sequence and Count(AGO1/wt):GAGCUCCUUGAAGUUCAAUGG_12.19/86.93

miRNA Position in PremiRNA:6-26

miRNA annonation:ath-miR159b

4. Degradome supported cutsite(s) in Genome:6740590

PremiRNA Locus in Genome:>Chr1(6740498-6740593)_plus strand

PremiRNA sequence:UUGCUUCAGGUAUGAUUGACUUCAAAAAAUACCUUGAAACUAUAAACCUCAGUUUCUUUGAAUUUGAUUUUUAAAGUCAAUAAUACCUUGAAGCAA

PremiRNA secondary structure:(((((((((((((.((((((((.(((((.......((((((.........))))))...........))))).)))))))).))))).))))))))

Degradome supported cutsite(s) in premiRNA:93

miRNA sequence and Count(AGO1/wt):UCAGGUAUGAUUGACUUCAAA_81.82/11.47

miRNA Position in PremiRNA:6-26

miRNA* sequence and Count(AGO1/wt):UAAAGUCAAUAAUACCUUGAAG_2.89/0.37

miRNA Position in PremiRNA:72-93

miRNA annonation:ath-miR864-5p

5. Degradome supported cutsite(s) in Genome:9364561

PremiRNA Locus in Genome:>Chr1(9364477-9364564)_plus strand

PremiRNA sequence:CCAUGAGUUCCCUUUAACGCUUCAUUGUUAAAUACUCAAAGCCACAUUGGUUUGUAUACAACACUGAAGUGUUUGGGGGACUCUUGG

PremiRNA secondary structure:(((.(((((((((..(((((((((.((((..((((...(((((.....)))))))))..)))).)))))))))..))))))))).)))

Degradome supported cutsite(s) in premiRNA:85

miRNA sequence and Count(AGO1/wt):CUGAAGUGUUUGGGGGACUC_87.4/10.73

miRNA Position in PremiRNA:64-84

miRNA* sequence and Count(AGO1/wt):GUUCCCUUUAACGCUUCAUUG_0/0.55

miRNA Position in PremiRNA:7-27

miRNA annonation:ath-miR395f/ath-miR395c/ath-miR395b

6. Degradome supported cutsite(s) in Genome:17825722
PremiRNA Locus in Genome:>Chr1(17825720-17825829)_plus strand
PremiRNA
sequence:GAUCAAUGCAUUGAAAGUGACUACAUCGGGGUUCCGAUUUUUUUUGUUCUUCAUAUGAUGAAGCGGAAACAGUAAUCAACCCUGGUUUAGU
CACUUUCACUGCAUUAAUC
PremiRNA secondary structure:(((.((((((.((((((((((((.(((((((((..((((....(((((((((((...))))))....))))).))))))))))))).)))))))))))).)))))).)))
Degradome supported cutsite(s) in premiRNA:3
miRNA sequence and Count(AGO1/wt):UCAAUGCAUUGAAAGUGACUA_12175.67/955.65
miRNA Position in PremiRNA:3-23
miRNA* sequence and Count(AGO1/wt):GUCACUUUCACUGCAUUAAUC_8.27/6.47
miRNA Position in PremiRNA:90-110
miRNA annonation:ath-miR161.2

7. Degradome supported cutsite(s) in Genome:20041586
PremiRNA Locus in Genome:>Chr1(20041581-20041665)_plus strand
PremiRNA
sequence:GAGAUUGAGCCAAGGAUGACUUGCCGAUUUUCUCAACGAAUCUUACUGAUUAUGGUAUCCGGCAAGUUGACUUUGGCUCUGUUUC

PremiRNA secondary structure:(((((.(((((((((.((((((((((...(((.....)))...((((......))))..)))))))))).))))))))).)))))
Degradome supported cutsite(s) in premiRNA:6
miRNA sequence and Count(AGO1/wt):UGAGCCAAGGAUGACUUGCCG_28.31/3.7
miRNA Position in PremiRNA:6-26
miRNA* sequence and Count(AGO1/wt):GCAAGUUGACUUUGGCUCUGU_0.21/15.72
miRNA Position in PremiRNA:62-82
miRNA annonation:ath-miR169g-5p/ath-miR169d/ath-miR169e/ath-miR169f

8. Degradome supported cutsite(s) in Genome:22149808/22149735
PremiRNA Locus in Genome:>Chr1(22149730-22149831)_plus strand
PremiRNA
sequence:CGAAAUCUCUCUGUUGUGAAGUCAAACAUGAGUAUGAAUUAACAUUAAUGGAUCUUUUUGAUUCAUAAUACUCAUGUUUGGUUUCACAGUA
GAUAGAUAUCG
PremiRNA secondary structure:(((.((((.(((((((((((..(((((((((((((............(((((((.....))))))).)))))))))))))..))))))))))).)))).)))

Degradome supported cutsite(s) in premiRNA:79/6
miRNA sequence and Count(AGO1/wt):UCUCUCUGUUGUGAAGUCAAA_3.51/0.92
miRNA Position in PremiRNA:6-26
miRNA* sequence and Count(AGO1/wt):UGGUUUCACAGUAGAUAGAUA_0.41/0.55
miRNA Position in PremiRNA:79-99
miRNA annonation:ath-miR859

9. Degradome supported cutsite(s) in Genome:24554011
PremiRNA Locus in Genome:>Chr1(24553982-24554031)_plus strand
PremiRNA sequence:CGUACACUUACAUCUGUACACUAAAAGUGUUGUACAAAUUUAAGUGUACG
PremiRNA secondary structure:((((((((((.((.(((((((.......).)))))).)).))))))))))
Degradome supported cutsite(s) in premiRNA:30
miRNA sequence and Count(AGO1/wt):UUGUACAAAUUUAAGUGUACG_14.26/0.92
miRNA Position in PremiRNA:30-50
miRNA* sequence and Count(AGO1/wt):UACACUUACAUCUGUACACUA_0.21/0
miRNA Position in PremiRNA:3-23
miRNA annonation:ath-miR5014a-3p

10. Degradome supported cutsite(s) in Genome:24884359
PremiRNA Locus in Genome:>Chr1(24884354-24884581)_plus strand
PremiRNA
sequence:AGUCGUUGAAGAGGACUUGGAACUUCGAUAUUAUCCCCCGUGUUUUGUCCAGUUUCUCUAUAGGUUGUGCCUGAAAAUAUGGGUUUCUAGG
UUUAAUUGAUGAAAAGAGAUAACGUAAGUCAUGCACAUGUAAUUAAAUUCAUGCCCCUAACUAUUAAUUCUUGCAAGUAAUUAAAAAAUAAAACGU
ACACAAAAUAUAUUUAUGAUCACUAAUCCUUUUUAACUACU
PremiRNA secondary
structure:(((.((((((((((.((((......(((...)))....(((((((((...((((.....((((.(((((.(((...((((.((((((...((((.....)))).))))))..))))..))).))))).(((..........))).))))....((((((((....)).))))))......))))....)))))))))..........))))))))
))))))).)))
Degradome supported cutsite(s) in premiRNA:6
miRNA sequence and Count(AGO1/wt):UUGAAGAGGACUUGGAACUUCGAU_35596.78/2218.51
miRNA Position in PremiRNA:6-29
miRNA* sequence and Count(AGO1/wt):UAUCCCCCGUGUUUUGUCC_1.86/0.18
miRNA Position in PremiRNA:35-225
miRNA annonation:ath-miR163

11. Degradome supported cutsite(s) in Genome:24921104

PremiRNA Locus in Genome:>Chr1(24921099-24921200)_plus strand

PremiRNA sequence:UAGUGUUGACAGAAGAUAGAGAGCACAGAUGAUAAGAUACAAUUCCUCGCAGCUUCUUUGCAUCUUACUCCUUUGUGCUCUCUAGCCUUCUGUCAUCACCCG

PremiRNA secondary structure:(.(((.(((((((((.((((((((((((((.((((((((.(((..............))).))))))).)).)))))))))))))..))))))))).)))..)

Degradome supported cutsite(s) in premiRNA:6

miRNA sequence and Count(AGO1/wt):UUGACAGAAGAUAGAGAGCAC_0/1656.07

miRNA Position in PremiRNA:6-26

miRNA* sequence and Count(AGO1/wt):GCUCUCUAGCCUUCUGUCAUCA_1.24/13.13

miRNA Position in PremiRNA:77-98

miRNA annonation:ath-miR157b/ath-miR157a/ath-miR157c

12. Degradome supported cutsite(s) in Genome:26638083

PremiRNA Locus in Genome:>Chr1(26638000-26638107)_plus strand

PremiRNA sequence:AGAGAGCAACAAAAUCAGUGUGUAUUUGUUAAGACAAAUGUUUAUAUAAGUCGUCCAUGCGUUGUACAUUUAAUCUUAACUAAUACGCAUUGAGUUUCGUUGCUUUCU

PremiRNA secondary structure:((((((((((((.((((((((((((.(((((((.((((((....((((..(((....)))))))))))))..))))))).))))))))))))).))).))))))))))

Degradome supported cutsite(s) in premiRNA:84

miRNA sequence and Count(AGO1/wt):UACGCAUUGAGUUUCGUUGCUU_47.73/15.35

miRNA Position in PremiRNA:84-105

miRNA* sequence and Count(AGO1/wt):CAACAAAAUCAGUGUGUAUU_0.62/0

miRNA Position in PremiRNA:6-26

miRNA annonation:ath-miR777

13. Degradome supported cutsite(s) in Genome:29422546

PremiRNA Locus in Genome:>Chr1(29422456-29422570)_plus strand

PremiRNA sequence:AACGUUGCACUACGUGACAUUGAAACUGUCUUUCAACAUUCCAAUAUUUCAACUUUCGAAUACCCAAUAUUUGGUUUGUUCAAAGACAUUUUCGAUGUCUAGCAGUGCCAAUGUU

PremiRNA secondary structure:(((((((((((....((((((((((.(((((((.((((..((((((((................)))).))))..)))).))))))).))))))))))....)))).))))))))

Degradome supported cutsite(s) in premiRNA:91

miRNA sequence and Count(AGO1/wt):UUCGAUGUCUAGCAGUGCCA_781.46/57.52

miRNA Position in PremiRNA:91-110
miRNA* sequence and Count(AGO1/wt):GCACUACGUGACAUUGAAAC_0.83/11.1
miRNA Position in PremiRNA:7-26
miRNA annonation:ath-miR775

14. Degradome supported cutsite(s) in Genome:22150026
PremiRNA Locus in Genome:>Chr1(22149939-22150030)_plus strand
PremiRNA
sequence:UUUCAGAUGGCUGUUUGGGUAACUAAUAUUUAAGAUUUUGGUCAAUUUAAGUUACCAAUUUAUAUAUUGGUUACCCAUAUGGCCAUCUCAA
A
PremiRNA secondary structure:(((.((((((((((.((((((((((((((.((((...(((((.(((....)))))))))))).))))))))))))))).))))))))))).)))
Degradome supported cutsite(s) in premiRNA:88
miRNA sequence and Count(AGO1/wt):UUGGUUACCCAUAUGGCCAUC_1.03/0.37
miRNA Position in PremiRNA:67-87
miRNA* sequence and Count(AGO1/wt):UGGCUGUUUGGGUAACUAAUA_0/0
miRNA Position in PremiRNA:8-28
miRNA annonation:ath-miR774a

15. Degradome supported cutsite(s) in Genome:1041012
PremiRNA Locus in Genome:>Chr2(1040937-1041037)_plus strand
PremiRNA
sequence:GUGAAAUUUCAAAGGAGUGGCAUGUGAACACAUAUCCUAUGGUUUCUUCAAAUUUCCAUUGAAACCAUUGAGUUUUGUGUUCUCAGGUCAC
CCCUUUGAAU
PremiRNA secondary structure:((......(((((((.(((((.((.(((((((..((..((((((((..............)))))))).))....))))))).)).))))).)))))))))
Degradome supported cutsite(s) in premiRNA:76
miRNA sequence and Count(AGO1/wt):UGUGUUCUCAGGUCACCCCUU_7.85/2.77
miRNA Position in PremiRNA:76-96
miRNA* sequence and Count(AGO1/wt):GGAGUGGCAUGUGAACACAUA_0/12.39
miRNA Position in PremiRNA:14-34
miRNA annonation:ath-miR398a

16. Degradome supported cutsite(s) in Genome:9560866/9560887
PremiRNA Locus in Genome:>Chr2(9560769-9560891)_plus strand

PremiRNA
sequence:UAAUAUAGUUGAAUCUUCGAAAGUAUUUCAAGAAGUCAGCUGAGCUUUCUCGUCAUCACUUAUUAGUAAAUAUAGUCUCUAUAUUUUGAUGAGUGAUGAUUGGAAAUUUCGUUGACUCAUUA

PremiRNA secondary structure:((((....(((((....(....)...)))))..(((((((.(((.(((((.((((((((((((((.((((((((...)))))))))))))))))))))).))))).)))))))))).))))

Degradome supported cutsite(s) in premiRNA:98/119

miRNA sequence and Count(AGO1/wt):UGAUUGGAAAUUUCGUUGACU_9.5/2.96

miRNA Position in PremiRNA:98-118

miRNA* sequence and Count(AGO1/wt):UCAGCUGAGCUUUCUCGUCAUC_71.29/6.29

miRNA Position in PremiRNA:36-57

miRNA annonation:ath-miR779.2

17. Degradome supported cutsite(s) in Genome:11159774

PremiRNA Locus in Genome:>Chr2(11159706-111159799)_plus strand

PremiRNA
sequence:GACUCGUUCAAGCACCAGCUCGAAGAAGCUUAGCUAAUUUAUCUUAGAAAAUAAUGAAAAAGCUAUGCUUCUCAAGAAGGUGCAUGAACAAGUU

PremiRNA secondary structure:((((.(((((.(((((..((.((.(((((.(((((..(((((..(.....)..)))))..))))).))))))).))..))))).))))).))))

Degradome supported cutsite(s) in premiRNA:69

miRNA sequence and Count(AGO1/wt):UUCUCAAGAAGGUGCAUGAAC_310.15/46.24

miRNA Position in PremiRNA:69-89

miRNA* sequence and Count(AGO1/wt):UCAAGCACCAGCUCGAAGAAGC_40.5/10.91

miRNA Position in PremiRNA:8-29

miRNA annonation:ath-miR825

18. Degradome supported cutsite(s) in Genome:15611882

PremiRNA Locus in Genome:>Chr2(15611875-15611978)_plus strand

PremiRNA
sequence:AGAGAAGUGAGAUGAAAUCUUUGAUUGGAAAUUUCAUGUAGACGAUGCUAUCACCCCAUAUGACCAAAAGAAAUUCCCAAUUAAAGAUUUCAUCUUACUUCCCU

PremiRNA secondary structure:((.(((((((((((((((((((((((((.((((((..(((.....)))..(((.......)))......)))))).)))))))))))))))))))))))))).))

Degradome supported cutsite(s) in premiRNA:8

miRNA sequence and Count(AGO1/wt):AAUUAAAGAUUUCAUCUUACU_1.03/4.99

miRNA Position in PremiRNA:79-99

miRNA* sequence and Count(AGO1/wt):UGAGAUGAAAUCUUUGAUUGG_114.88/15.35

miRNA Position in PremiRNA:8-28
miRNA annonation:ath-miR1886.3

19. Degradome supported cutsite(s) in Genome:16061971
PremiRNA Locus in Genome:>Chr2(16061966-16062046)_plus strand
PremiRNA sequence:CUGUAAAGCUCAGGAGGGAUAGCGCCAUGAUGAUCACAUUCGUUAUCUAUUUUUGGCGCUAUCCAUCCUGAGUUUCAUUG

PremiRNA secondary structure:(.((.((((((((((.(((((((((((.((((((.((....)).))).)))...))))))))))).)))))))))).)).)
Degradome supported cutsite(s) in premiRNA:6
miRNA sequence and Count(AGO1/wt):AAGCUCAGGAGGGAUAGCGCC_408.29/331.25
miRNA Position in PremiRNA:6-26
miRNA* sequence and Count(AGO1/wt):CGCUAUCCAUCCUGAGUUUCA_14.26/103.2
miRNA Position in PremiRNA:58-78
miRNA annonation:ath-miR390b/ath-miR390a

20. Degradome supported cutsite(s) in Genome:16340282/16340363
PremiRNA Locus in Genome:>Chr2(16340279-16340363)_plus strand
PremiRNA sequence:GUAUGCCUGGCUCCCUGUAUGCCAUAUGCUGAGCCCAUCGAGUAUCGAUGACCUCCGUGGAUGGCGUAUGAGGAGCCAUGCAUAU

PremiRNA secondary structure:((((((.(((((((..(((((((((.(((.(((..(((((.....)))))..))).))).)))))))))..))))))).))))))
Degradome supported cutsite(s) in premiRNA:4/85
miRNA sequence and Count(AGO1/wt):UGCCUGGCUCCCUGUAUGCCA_4143.89/231.56
miRNA Position in PremiRNA:4-24
miRNA* sequence and Count(AGO1/wt):GCGUAUGAGGAGCCAUGCAUA_0.21/30.33
miRNA Position in PremiRNA:64-84
miRNA annonation:ath-miR160a/ath-miR160c/ath-miR160b

21. Degradome supported cutsite(s) in Genome:19176240
PremiRNA Locus in Genome:>Chr2(19176122-19176265)_plus strand
PremiRNA
sequence:UUGAGGGGACUGUUGUCUGGCUCGAGGACUCUGGCUCGCUCUAUUCAUGUUGGAUCUCUUUCGAUCUAACAAUCGAAUUGAACCUUCAGAU
UUCAGAUUUGAUUAGGGUUUUAGCGUCUUCGGACCAGGCUUCAUUCCCCCCAA
PremiRNA secondary structure:(((.(((((.((..((((((..(((((((.((((...((((((.((((((((((((......)))))))))...(((((((....))))..))).....))).)))))).)))).)))))))..))))))..)).))))).)))
Degradome supported cutsite(s) in premiRNA:119

miRNA sequence and Count(AGO1/wt):UCGGACCAGGCUUCAUUCCCC_42640.47/1448.18
miRNA Position in PremiRNA:120-140
miRNA* sequence and Count(AGO1/wt):GGACUGUUGUCUGGCUCGAGG_0/110.05
miRNA Position in PremiRNA:7-27
miRNA annonation:ath-miR166b/ath-miR166f/ath-miR166a/ath-miR166g/ath-miR166c/ath-miR166d/ath-miR166e

22. Degradome supported cutsite(s) in Genome:19520770
PremiRNA Locus in Genome:>Chr2(19520766-19520851)_plus strand
PremiRNA
sequence:AUGUUGGAGAAGCAGGGCACGUGCAAACCAACAAACACGAAAUCCGUCUCAUUUGCUUAUUUGCACGUACUUAACUUCUCCAACAU

PremiRNA secondary structure:((((((((((((..(((.(((((((((....((((.(((.....)))....))))....))))))))).)))..))))))))))))
Degradome supported cutsite(s) in premiRNA:5
miRNA sequence and Count(AGO1/wt):UGGAGAAGCAGGGCACGUGCA_968.05/196.6
miRNA Position in PremiRNA:5-25
miRNA* sequence and Count(AGO1/wt):CACGUACUUAACUUCUCCAAC_1.65/2.22
miRNA Position in PremiRNA:64-84
miRNA annonation:ath-miR164b/ath-miR164a

23. Degradome supported cutsite(s) in Genome:6217561
PremiRNA Locus in Genome:>Chr3(6217490-6217584)_plus strand
PremiRNA
sequence:CAAGUAUGGUUUUCUUGGCCCAUCCACUUCUAAUGUGUUUUGUAACUAAUGCAAAAUGUGCAUUAGAAGUGGAUUGGUCAAGGGAAGCGUA
CUUG
PremiRNA secondary structure:((((((((.((..(((((((.(((((((((((((((((((((((.....))))))))..))))))))))))))).)))))))..)).))))))))
Degradome supported cutsite(s) in premiRNA:72
miRNA sequence and Count(AGO1/wt):UGGAUUGGUCAAGGGAAGCGU_0.62/0.18
miRNA Position in PremiRNA:70-90
miRNA* sequence and Count(AGO1/wt):UUUUCUUGGCCCAUCCACUUC_4.96/1.29
miRNA Position in PremiRNA:8-28
miRNA annonation:ath-miR3440b-3p

24. Degradome supported cutsite(s) in Genome:8108090/8108172/8108193
PremiRNA Locus in Genome:>Chr3(8108090-8108190)_plus strand

PremiRNA sequence:UGAAGCUGCCAGCAUGAUCUAAUUAGCUUUCUUUAUCCUUUGUUGUGUUUCAUGACGAUGGUUAAGAGAUCAGUCUCGAUUAGAUCAUGUUCGCAGUUUCA
PremiRNA secondary structure:(((((((((.((((((((((((((((((.(((((..((.((((..((...))..)))).))...)))))..)).)).))))))))))))).)))))))))
Degradome supported cutsite(s) in premiRNA:1/83/104
miRNA sequence and Count(AGO1/wt):UGAAGCUGCCAGCAUGAUCUA_13857.2/2368.5
miRNA Position in PremiRNA:1-21
miRNA* sequence and Count(AGO1/wt):GAUCAUGUUCGCAGUUUCACC_0/0.55
miRNA Position in PremiRNA:83-103
miRNA annonation:ath-miR167b/ath-miR167a

25. Degradome supported cutsite(s) in Genome:8236161
PremiRNA Locus in Genome:>Chr3(8236156-8236251)_plus strand
PremiRNA sequence:GUACUUUCGCUUGCAGAGAGAAAUCACAGUGGUCAAAAAAGUUGUAGUUUUCUUAAAGUCUCUUUCCUCUGUGAUUCUCUGUGUAAGCGAAAGAGC
PremiRNA secondary structure:((.((((((((((((.((((((.((((((.((.........(((.((....))))).........)).)))))))))))).)))))))))))).))
Degradome supported cutsite(s) in premiRNA:6
miRNA sequence and Count(AGO1/wt):UUCGCUUGCAGAGAGAAAUCAC_9068.22/1310.58
miRNA Position in PremiRNA:6-27
miRNA* sequence and Count(AGO1/wt):GAUUCUCUGUGUAAGCGAAAG_7.44/25.15
miRNA Position in PremiRNA:73-93
miRNA annonation:ath-miR173-5p

26. Degradome supported cutsite(s) in Genome:8346589/8346610
PremiRNA Locus in Genome:>Chr3(8346411-8346612)_plus strand
PremiRNA sequence:GCUGCUCCCCUCUUUAGCUUGGAGAAGCCAGUAAAUAUCUUUCUCUUCAAGGAACUUGUGGGCCUCAAGAAAACCUGCUAUAAUUCUAGUUUUUCAACAUUCCUCCUUCUGAUAUCAGCAACAACUCCUUACCCUUAAGAAUGGAGAAUGGAACUUCCUCAAAGAUAUUUACUUGGCCUCUGCAACCGGAAAGGGGAGCAGC
PremiRNA secondary structure:(((((((((((.((..(.(((.(((.(((((((((((((((.......(((..(....)..)))...(((((...((((......)))))))))..(((((.(((..(((....))).........((((....))))...))))))))..........)))))))))))).)))).))).)))).)).)))))))))))

Degradome supported cutsite(s) in premiRNA:179/200

miRNA sequence and Count(AGO1/wt):UCCCCUCUUUAGCUUGGAGAAG_29.34/2.77
miRNA Position in PremiRNA:6-27
miRNA* sequence and Count(AGO1/wt):UCUGCAACCGGAAAGGGGAGC_7.85/1.29
miRNA Position in PremiRNA:179-199
miRNA annonation:ath-miR853

27. Degradome supported cutsite(s) in Genome:19073542
PremiRNA Locus in Genome:>Chr3(19073446-19073545)_plus strand
PremiRNA
sequence:CUUUGAUAUUGGCCUGGUUCACUCAGAUCUUACCUGACCACACACGUAGAUAUACAUUAUUCUCUCUAGAUUAUCUGAUUGAGCCGCGCCAAUAUCUCAG
PremiRNA secondary structure:((..(((((((((.(((((((.((((((.((((.((......)).))))...........(((....)))..)))))).))))))).)))))))))..))
Degradome supported cutsite(s) in premiRNA:97
miRNA sequence and Count(AGO1/wt):UGAUUGAGCCGCGCCAAUAUC_1323.03/352.7
miRNA Position in PremiRNA:76-96
miRNA* sequence and Count(AGO1/wt):UAUUGGCCUGGUUCACUCAGA_594.67/40.69
miRNA Position in PremiRNA:7-27
miRNA annonation:ath-miR171a

28. Degradome supported cutsite(s) in Genome:20587991
PremiRNA Locus in Genome:>Chr3(20587914-20588016)_plus strand
PremiRNA
sequence:UGCUAUUGCAACAUCUUCAAGAUUCAGAAAUCAGAUUCUCUUAUGGGUUUUCUUUUGAGCCUUUAUUUUUUGGUUUGAGAAUCUUGAUGAUGCUGCAGCGGCA
PremiRNA secondary structure:(((..(((((.((((.(((((((((..((((((((......((.((((((......)))))).))...))))))))..))))))))).)))).)))))..)))
Degradome supported cutsite(s) in premiRNA:78
miRNA sequence and Count(AGO1/wt):AGAAUCUUGAUGAUGCUGCAG_36.99/16.28
miRNA Position in PremiRNA:78-98
miRNA* sequence and Count(AGO1/wt):GCAACAUCUUCAAGAUUCAGA_0/2.03
miRNA Position in PremiRNA:8-28
miRNA annonation:ath-miR172d/ath-miR172c

29. Degradome supported cutsite(s) in Genome:22922301/22922321
PremiRNA Locus in Genome:>Chr3(22922206-22922325)_plus strand

PremiRNA
sequence:UUGAGGGGACUGUUGUCUGGCUCGAGGACUCUUAUUCUAAUACAAUCUCAUUUGAAUACAUUCAGAUCUGAUGAUUGAUUAGGGUUUUAGU
GUCGUCGGACCAGGCUUCAUUCCCCCCAA
PremiRNA secondary structure:(((.(((((.((..((((((..(((.(((.((.((((((((.(((((((((((((((....))))))..))).)))))))))))))...)).))).)))..))))))..)).))))).)))
Degradome supported cutsite(s) in premiRNA:96/116
miRNA sequence and Count(AGO1/wt):UCGGACCAGGCUUCAUUCCCC_42640.47/1448.18
miRNA Position in PremiRNA:96-116
miRNA* sequence and Count(AGO1/wt):GGACUGUUGUCUGGCUCGAGG_0/110.05
miRNA Position in PremiRNA:7-27
miRNA annonation:ath-miR166b/ath-miR166f/ath-miR166a/ath-miR166g/ath-miR166c/ath-miR166d/ath-miR166e

30. Degradome supported cutsite(s) in Genome:23406177
PremiRNA Locus in Genome:>Chr3(23406172-23406272)_plus strand
PremiRNA
sequence:ACAAGUGAAGCUGCCAGCAUGAUCUAUCUUUGGUUAAGAGAUGAAUGUGGAAACAUAUUGCUUAAACCCAAGCUAGGUCAUGCUCUGACAG
CCUCACUCCU
PremiRNA secondary structure:(..(((((.(((((.(((((((((((.(((.((((..(((...((((((....)))))).))).)))).))).)))))))))))..).)))).)))))..)
Degradome supported cutsite(s) in premiRNA:6
miRNA sequence and Count(AGO1/wt):UGAAGCUGCCAGCAUGAUCUA_13857.2/2368.5
miRNA Position in PremiRNA:6-26
miRNA* sequence and Count(AGO1/wt):GGUCAUGCUCUGACAGCCUCACU_0.62/13.5
miRNA Position in PremiRNA:76-98
miRNA annonation:ath-miR167b/ath-miR167a

31. Degradome supported cutsite(s) in Genome:9889047
PremiRNA Locus in Genome:>Chr4(9888982-9889070)_plus strand
PremiRNA
sequence:GUCGUGCCUGGCUCCCUGUAUGCCACAAGAAAACAUCGAUUUAGUUUCAAAAUCGAUCACUAGUGGCGUACAGAGUAGUCAAGCAUGAC

PremiRNA secondary structure:(((((((.(((((.((((((((((((.((.....((((((((.......))))))))..)).)))))))))))).).))))).)))))))
Degradome supported cutsite(s) in premiRNA:66
miRNA sequence and Count(AGO1/wt):UGCCUGGCUCCCUGUAUGCCA_4143.89/231.56
miRNA Position in PremiRNA:5-25
miRNA* sequence and Count(AGO1/wt):GCGUACAGAGUAGUCAAGCAUG_0.41/14.98

miRNA Position in PremiRNA:66-87
miRNA annonation:ath-miR160a/ath-miR160c/ath-miR160b

32. Degradome supported cutsite(s) in Genome:10578652/10578737/10578758
PremiRNA Locus in Genome:>Chr4(10578647-10578760)_plus strand
PremiRNA
sequence:CGGAUUCGCUUGGUGCAGGUCGGGAACCAAUUCGGCUGACACAGCCUCGUGACUUUUAAACCUUUAUUGGUUUGUGAGCAGGGAUUGGAUC
CCGCCUUGCAUCAACUGAAUCGG
PremiRNA secondary structure:(.((((((.((((((((((.(((((.((((((((((((...)))))...((.(((.((((((......)))))).))))).))))))).))))).)))))))))).)))))).)
Degradome supported cutsite(s) in premiRNA:6/91/112
miRNA sequence and Count(AGO1/wt):UCGCUUGGUGCAGGUCGGGAA_1250.3/166.64
miRNA Position in PremiRNA:6-26
miRNA* sequence and Count(AGO1/wt):CCCGCCUUGCAUCAACUGAAU_23.56/236.37
miRNA Position in PremiRNA:91-111
miRNA annonation:ath-miR168b/ath-miR168a

33. Degradome supported cutsite(s) in Genome:12353109/12353130
PremiRNA Locus in Genome:>Chr4(12352440-12353134)_plus strand
PremiRNA
sequence:GUUCUAAAUUUGAAGCUUAUAAAAACCCAUCACUACUUUUGCAUACUUGUAUCCGCAGUGUAUUUCCUCGCAUCUACCAUCCCUUUUCUACG
CCUCUCUCCCUCUCUCUCUUUCUCCAUCAAAUCUUGUUUUGUUCAAACUCUCUCUCUCUCAUCUAUUCUCUCCAUACAAUACAUGAAUAUACAUACAU
ACCAUCAUCUUCUUUUCCCAUCUCUAGUUUUUCAUCAAUCUUCUGAUGUUCCAAACGCUCUAUCUCUUCAUAUACAUACAUACGAAUAUAUUAUUGU
UGUCAUAGAUCCAUUUAGAAUCACUUUAGCUUUUAGAUGAGAUCUAGGGUUUCUUUGUUUUCUUUCAAAUUUUGUUGCAUAUUCUUCUAAAUCAUG
GUUUUUCGCUUGCUAGGUUAUAGAUCCAUGCAAAUAUGGAGUAGAUGUACAAACACACGCUCGGACGCAUAUUACACAUGUUCAUACACUUAAUACU
CGCUGUUUUGAAUUGAUGUUUUAGGAAUAUAUAUGUAGAGAGAGCUUCCUUGAGUCCAUUCACAGGUCGUGAUAUGAUUCAAUUAGCUUCCGACUC
AUUCAUCCAAAUACCGAGUCGCCAAAAUUCAAACUAGACUCGUUAAAUGAAUGAAUGAUGCGGUAGACAAAUUGGAUCAUUGAUUCUCUUUGAUUG
GACUGAAGGGAGCUCCCUCUCUC
PremiRNA secondary
structure:(.....................................((((((..((..((((.(((((........((((((((...((((......))))...........................((((((.......((((...(((.....(((.......................((((..(((((......))))).
...))))....................(((.((((........)))))))..((((((((((((((.(......).))))))))).)))))).))).....))).....)))).......((((((.....((((((((.((((.........)))))))).))))...)))))).)))))))))))))........)))))))))..))........((((((..(((
.(((((..(((......)))))))))))).....)))))).))))))(((.((((((((((.((((((.(((.(((..((.(.(((((((((((.(((.(((..((((((((.((..((.((((((................)))))).))..)).))))))))..))).)).).))))))))))).))..))).))).)))))).)))))))))).)))...)

Degradome supported cutsite(s) in premiRNA:670/691
miRNA sequence and Count(AGO1/wt):UUGGACUGAAGGGAGCUCCCU_4553.01/736.11

miRNA Position in PremiRNA:670-690
miRNA* sequence and Count(AGO1/wt):AGAGCUUCCUUGAGUCCAUUC_3.31/9.43
miRNA Position in PremiRNA:521-541
miRNA annonation:ath-miR319b/ath-miR319a

34. Degradome supported cutsite(s) in Genome:12625128/12625742
PremiRNA Locus in Genome:>Chr4(12625123-12625744)_plus strand
PremiRNA
sequence:AUGUCUAGACCAUUUGUGAGAAGGGAGUUUUUGUUUACACCAAUACCCCCAGUCUCUAAAUUUGUAAGAAGUAUUAUGCUCAAUUAAGGAACAUAGCUAGUUCGACAUACCAUACUACCCUUUUUAAAACUAUCCUAUAUGUUUGAUGCUAGCAUAGCGUAAUUUUGUGUUCUCAUGGUGCAGCAGGGUUAGUUUAUUGUGUACCUCUAGAUAUAUAUUCUCUUGCUGCGUAGGGUUCCCAAGCUGCCAAAAACUUUUAAAAAUUAGUGAUCUGUUCCCCAAACCCCCAUUCAAUAAGAAAGGUCUACCUGUAGCUCACAGUCACAGCUAUUAGAGCUGUCCUUGCUUCUUUGGGUUUACUGUUUUAGUUAAUUAGUUACCCUAUGAAAGUCUGAUCCUUCAGAAGUUAGGUAUAGAAGUAAGUCGAUUAAGUUCAUUCAUGACUUUUCCAGAUGUUGCAUACAUAACUUUUUUUAUUUGUAUUCGAACUAGUCCAAGCCGAUUCUCAAAAUCAUAUAAAAUCAUUUACACCUUGGUCAUGGUAGCUAAGAAUAUUGUAUCUAAAAUUGGGGAGUGGGGAGAUGUUUGGUUAUAUUCCCUUCUCAUCGAUGGUCUAGAUGU
PremiRNA secondary
structure:(((((((((((((.(((((((((((((..........(((((((.((((((.(((((((.((((.....((((((..((((.(((((((......((((((((((.((((..(((((...(((((((((......((((......((((((...)))))).....)))).......(((((((((((...((..(((.((((........))))))..))))))))))...((....)).....)))).....))))))))).)))))......................((((((((((((.....((((((((......((((((.....)))))).((((((((((..((((..((((...........)))).))))..(((...((((((....)))))..))).....)))))))))..((..((((((.......)))).))..)).))))))......)))))))))))).))))))))))))))).......((((.....)))).................)))))))....).)))....))))))).....)))).))))))).))))).).)).)))))...))))))))))))).))))))))))))))
Degradome supported cutsite(s) in premiRNA:6/620
miRNA sequence and Count(AGO1/wt):UAGACCAUUUGUGAGAAGGGA_1973.7/308.13
miRNA Position in PremiRNA:6-26
miRNA* sequence and Count(AGO1/wt):CCUUCUCAUCGAUGGUCUAGA_48.76/76.39
miRNA Position in PremiRNA:599-619
miRNA annonation:ath-miR824

35. Degradome supported cutsite(s) in Genome:15074945
PremiRNA Locus in Genome:>Chr4(15074940-15075033)_plus strand
PremiRNA
sequence:AAAACUGACAGAAGAGAGUGAGCACAUGCAGGCACUGUUAUGUGUCUAUAACUUUGCGUGUGCGUGCUCACCUCUCUUUCUGUCAGUUGCCUAU
PremiRNA secondary structure:(.((((((((((((((((((((((..(((.(((..((((((....))))))..))).)))..))))))).))))).)))))))))))).....)
Degradome supported cutsite(s) in premiRNA:6
miRNA sequence and Count(AGO1/wt):UGACAGAAGAGAGUGAGCAC_5994.85/1495.53

miRNA Position in PremiRNA:6-25
miRNA* sequence and Count(AGO1/wt):GCUCACCUCUCUUUCUGUCAGU_18.39/91
miRNA Position in PremiRNA:66-87
miRNA annonation:ath-miR156e/ath-miR156f/ath-miR156d/ath-miR156a/ath-miR156c/ath-miR156b

36. Degradome supported cutsite(s) in Genome:287586
PremiRNA Locus in Genome:>Chr5(287581-287739)_plus strand
PremiRNA
sequence:CAAGAUGGAGAAGCAGGGCACGUGCAUUACUAGCUCAUAUAUACACUCUCACCACAAAUGCGUGUAUAUAUGCGGAAUUUUGUGAUAUAGA
UGUGUGUGUGUGUUGAGUGUGAUGAUAUGGAUGAGUUAGUUCUUCAUGUGCCCAUCUUCACCAUCAUG
PremiRNA secondary structure:((.(((((.((((..(((((((((....((((((((((.(((((((.((((.((((.((((((.(((((.(((((....)))))))))).))))))...)))).)))).)))....)))).))))))))))....)))))))))..)))).))))).))

Degradome supported cutsite(s) in premiRNA:6
miRNA sequence and Count(AGO1/wt):UGGAGAAGCAGGGCACGUGCA_968.05/196.6
miRNA Position in PremiRNA:6-26
miRNA* sequence and Count(AGO1/wt):AUGUGCCCAUCUUCACCAUC_0.83/0.18
miRNA Position in PremiRNA:140-156
miRNA annonation:ath-miR164b/ath-miR164a

37. Degradome supported cutsite(s) in Genome:3867213
PremiRNA Locus in Genome:>Chr5(3867208-3867312)_plus strand
PremiRNA
sequence:GGAGGUGACAGAAGAGAGUGAGCACACAUGGUGGUUUCUUGCAUGCUUUUUUGAUUAGGGUUUCAUGCUUGAAGCUAUGUGUGCUUACUCU
CUCUCUGUCACCCC
PremiRNA secondary structure:((.(((((((((((((((((((((((((((((...(((..(((((((((.......))))...)))))..))))))))))))))))))))))).)))))))))))
Degradome supported cutsite(s) in premiRNA:6
miRNA sequence and Count(AGO1/wt):UGACAGAAGAGAGUGAGCAC_5994.85/1495.53
miRNA Position in PremiRNA:6-25
miRNA* sequence and Count(AGO1/wt):GCUUACUCUCUCUCUGUCACC_0.21/8.88
miRNA Position in PremiRNA:83-103
miRNA annonation:ath-miR156e/ath-miR156f/ath-miR156d/ath-miR156a/ath-miR156c/ath-miR156b

38. Degradome supported cutsite(s) in Genome:4691128
PremiRNA Locus in Genome:>Chr5(4691027-4691132)_plus strand

PremiRNA
sequence:CUCGACAGGGUUGAUAUGAGAACACACGAGUAAUCAACGGCUGUAAUGACGCUACGUCAUUGUUACAGCUCUCGUUUUCAUGUGUUCUCAG
GUCACCCCUGCUGAG
PremiRNA secondary structure:((((.(((((.((((.((((((((((.(((.(((....(((((((((((((...))))))...)))))))...)))))).)))))))))).)))).))))).))))
Degradome supported cutsite(s) in premiRNA:102
miRNA sequence and Count(AGO1/wt):UGUGUUCUCAGGUCACCCCUG_206.21/40.69
miRNA Position in PremiRNA:81-101
miRNA* sequence and Count(AGO1/wt):GGGUUGAUAUGAGAACACACG_7.23/183.66
miRNA Position in PremiRNA:8-28
miRNA annonation:ath-miR398c/ath-miR398b

39. Degradome supported cutsite(s) in Genome:9098879/9098897
PremiRNA Locus in Genome:>Chr5(9098806-9098901)_plus strand
PremiRNA
sequence:UCAAUAUAUAUAGUCCAAUCUAUUGAAGUACUAGUACACCAGCUCUAAUAAGCUGAUGUGGGUAAGUAGUUCAAUAGAUUGGACUAUGUAU
AUUAA
PremiRNA secondary structure:(.((((((((((((((((((((((((((.((((.(..((((((((......)))))..)))..).)))).))))))))))))))))))))))))))).)
Degradome supported cutsite(s) in premiRNA:74/92
miRNA sequence and Count(AGO1/wt):UCAAUAGAUUGGACUAUGUAU_42.77/39.76
miRNA Position in PremiRNA:71-91
miRNA* sequence and Count(AGO1/wt):AUAUAGUCCAAUCUAUUGAAG_1.45/2.77
miRNA Position in PremiRNA:8-28
miRNA annonation:ath-miR860

40. Degradome supported cutsite(s) in Genome:9136126
PremiRNA Locus in Genome:>Chr5(9136121-9136223)_plus strand
PremiRNA
sequence:GAUGGUGACAGAAGAGAGUGAGCACACAUGGUGGCUUUCUUGCAUAUUUGAAGGUUCCAUGCUUGAAGCUAUGUGUGCUCACUCUCUAUCC
GUCACCCCCUUC
PremiRNA secondary structure:((.((((((.((((((((((((((((((((((....(((..((((............))))..))))))))))))))))))))))))).)).))))))....))
Degradome supported cutsite(s) in premiRNA:6
miRNA sequence and Count(AGO1/wt):UGACAGAAGAGAGUGAGCAC_5994.85/1495.53
miRNA Position in PremiRNA:6-25
miRNA* sequence and Count(AGO1/wt):GCUCACUCUCUAUCCGUCACC_0.62/10.73

miRNA Position in PremiRNA:77-97
miRNA annonation:ath-miR156e/ath-miR156f/ath-miR156d/ath-miR156a/ath-miR156c/ath-miR156b

41. Degradome supported cutsite(s) in Genome:9852684
PremiRNA Locus in Genome:>Chr5(9852679-9852770)_plus strand
PremiRNA
sequence:CUUGAUGGAGAAGCAGGGCACGUGCGAACACAAAUGAAAUCGAUCGGUACUUGUUGAUCAUAUUUUCGCACGUGUUCUACUACUCCAACAC
G
PremiRNA secondary structure:(.((.(((((.((.((((((((((((((..(....).....((((((......)))))).....)))))))))))))).)).))))).)).)
Degradome supported cutsite(s) in premiRNA:6
miRNA sequence and Count(AGO1/wt):UGGAGAAGCAGGGCACGUGCG_27.07/5.36
miRNA Position in PremiRNA:6-26
miRNA* sequence and Count(AGO1/wt):CACGUGUUCUACUACUCCAAC_1.45/27.37
miRNA Position in PremiRNA:69-89
miRNA annonation:ath-miR164c

42. Degradome supported cutsite(s) in Genome:13611904
PremiRNA Locus in Genome:>Chr5(13611803-13611927)_plus strand
PremiRNA
sequence:UACUUUUCCACAGCUUUCUUGAACUUUCUUUUUCAUUUCCAUUGUUUUUUUCUUAAACAAAAGUAAGAAGAAAAAAAACUUUAAGAUUAAG
CAUUUUGGAAGCUCAAGAAAGCUGUGGGAAAACA
PremiRNA secondary structure:(..(((((((((((((((((((.(((((................(((((((((((........)))))))))))....(((.......)))......))))).)))))))))))))))))))..)
Degradome supported cutsite(s) in premiRNA:102
miRNA sequence and Count(AGO1/wt):UUCCACAGCUUUCUUGAACUU_8517.76/1911.49
miRNA Position in PremiRNA:6-26
miRNA* sequence and Count(AGO1/wt):GCUCAAGAAAGCUGUGGGAAA_46.28/145.56
miRNA Position in PremiRNA:102-122
miRNA annonation:ath-miR396b

43. Degradome supported cutsite(s) in Genome:17516378/17516399
PremiRNA Locus in Genome:>Chr5(17516303-17516403)_plus strand
PremiRNA
sequence:GUUCAGGUGAAUGAUGCCUGGCUCGAGACCAUUCAAUCUCAUGAUCUCAUGAUUAUAACGAUGAUGAUGAUGAUGUCGGACCAGGCUUCAU
UCCCCUCAAC

PremiRNA secondary structure:(((.(((.((((((.((((((..(((.(.((((..(((((((((((....))))))...)).)))...)))).).)))..)))))).)))))).))).)))

Degradome supported cutsite(s) in premiRNA:76/97

miRNA sequence and Count(AGO1/wt):UCGGACCAGGCUUCAUUCCCC_42640.47/1448.18

miRNA Position in PremiRNA:76-96

miRNA* sequence and Count(AGO1/wt):UGAAUGAUGCCUGGCUCGAGA_1.03/0.55

miRNA Position in PremiRNA:8-28

miRNA annonation:ath-miR166b/ath-miR166f/ath-miR166a/ath-miR166g/ath-miR166c/ath-miR166d/ath-miR166e

44. Degradome supported cutsite(s) in Genome:19567227

PremiRNA Locus in Genome:>Chr5(19567222-19567349)_plus strand

PremiRNA sequence:UACAGUUGAAAUUGUAGAUUUCGUACAUGACAAUAAUUUGCUCGAGCAUCACUUUCAUGAUUUUGUGGUCUGUGUUGCCGUAUUUGAGAGCAUACAUUUCAUGUUCGAAAUCUACGAUUUUAACUCUA

PremiRNA secondary structure:((.((((((((((((((((((.((((((.(((....(((((..(((.(((..((((......))))...))).))).........)))))...)))))))).))))))))))))))))))).))

Degradome supported cutsite(s) in premiRNA:6

miRNA sequence and Count(AGO1/wt):UUGAAAUUGUAGAUUUCGUAC_0/0

miRNA Position in PremiRNA:6-26

miRNA* sequence and Count(AGO1/wt):UCGAAAUCUACGAUUUUAACU_0.21/0

miRNA Position in PremiRNA:105-125

miRNA annonation:ath-miR4243

45. Degradome supported cutsite(s) in Genome:21161609

PremiRNA Locus in Genome:>Chr5(21161504-21161634)_plus strand

PremiRNA sequence:AUGAAUUGUCAGUAUAAAUCUUUGAUCUGAUACUCUAUUAGUUAGUAUGUGAAUUUCUUUUUAACAAUUUUCUUAUUCAUGCGAGAGCUAUCAAUAAGUAUUAGAACAAAGUUUUAUACUGACAAUUCAAU

PremiRNA secondary structure:((((((((((((((((.(((((.(((((((((....(((((.(((((.(((................)))....)))))...)))))......))))))))).))))).))))))))))))))).))

Degradome supported cutsite(s) in premiRNA:106

miRNA sequence and Count(AGO1/wt):ACAAAGUUUUAUACUGACAAU_11.36/10.73

miRNA Position in PremiRNA:106-126

miRNA* sequence and Count(AGO1/wt):UGUCAGUAUAAAUCUUUGAUC_10.33/2.03

miRNA Position in PremiRNA:7-27

miRNA annonation:ath-miR4245

46. Degradome supported cutsite(s) in Genome:23636961
PremiRNA Locus in Genome:>Chr5(23636956-23637057)_plus strand
PremiRNA sequence:CUAUAAAGCUCAGGAGGGAUAGCGCCAUGGCUCACCAGUGCUGUAUGUUUUGUAUAUGCGUACAUGUAUAUCUGUUGGCGCUAUCCAUCCUGAGUUCCAUAG
PremiRNA secondary structure:((((..(((((((((.(((((((((((.((....)).((((((((((.........))))))).))))......))))))))))).)))))))))..))))
Degradome supported cutsite(s) in premiRNA:6
miRNA sequence and Count(AGO1/wt):AAGCUCAGGAGGGAUAGCGCC_408.29/331.25
miRNA Position in PremiRNA:6-26
miRNA* sequence and Count(AGO1/wt):CGCUAUCCAUCCUGAGUUCCA_0.41/2.4
miRNA Position in PremiRNA:79-99
miRNA annonation:ath-miR390b/ath-miR390a

47. Degradome supported cutsite(s) in Genome:24293160/24293211
PremiRNA Locus in Genome:>Chr5(24293156-24293233)_plus strand
PremiRNA sequence:UUGCUUCGCAGGAGAGAUAGCGCCAUCACCUCUUCUAAGAAGUUAACUAGUGGUGACGGUAUCUCUCCUACGUAGCAA

PremiRNA secondary structure:(((((.((.((((((((((.((.((((((..((((...)))).......)))))).)).)))))))))).)).)))))
Degradome supported cutsite(s) in premiRNA:5/56
miRNA sequence and Count(AGO1/wt):UUCGCAGGAGAGAUAGCGCCA_41.95/6.47
miRNA Position in PremiRNA:5-25
miRNA* sequence and Count(AGO1/wt):ACGGUAUCUCUCCUACGUAGC_3.72/40.5
miRNA Position in PremiRNA:56-76
miRNA annonation:ath-miR391

48. Degradome supported cutsite(s) in Genome:78932/78953
PremiRNA Locus in Genome:>Chr1(79037-78927)_minus strand
PremiRNA sequence:GUUGAGGGGAAUGUUGUCUGGAUCGAGGAUAUUAUAGAUAUAUACAUGUGUAUGUUAAUGAUUCAAGUGAUCAUAGAGAGUAUCCUCGGACCAGGCUUCAUCCCCCCCAAC
PremiRNA secondary structure:((((.((((.(((..((((((.(((((((((((...((((((((....)))))))).((((((.....))))))....))))))))))).))))))..))).)))).))))
Degradome supported cutsite(s) in premiRNA:107/86
miRNA sequence and Count(AGO1/wt):UCGGACCAGGCUUCAUCCCCC_19209.65/332.55
miRNA Position in PremiRNA:86-106

miRNA* sequence and Count(AGO1/wt):GGAAUGUUGUCUGGAUCGAGG_0.21/23.67
miRNA Position in PremiRNA:8-28
miRNA annonation:ath-miR165b/ath-miR165a

49. Degradome supported cutsite(s) in Genome:234017
PremiRNA Locus in Genome:>Chr1(234152-234013)_minus strand
PremiRNA
sequence:AUGAUGCGCAAAUGCGGAUAUCAAUGUAAAUCAGGACAACAAACCAAUAGAGGUGCUCAGCGAAAACAACAAAAUGUGAAAAGGGUUUAUC
AGAAACAUUGGGGUCAUGAUAUACCUUUAUAUCCGCAUUUGCGCAUCAU
PremiRNA secondary structure:(((((((((((((((((((((.((.(((.((((.(((..(((.......((...((((.......(((......))).....))))...)).......)))..))).)))).))).)).)))))))))))))))))))))
Degradome supported cutsite(s) in premiRNA:137
miRNA sequence and Count(AGO1/wt):CUUUAUAUCCGCAUUUGCGCA_44.63/9.8
miRNA Position in PremiRNA:116-136
miRNA* sequence and Count(AGO1/wt):CGCAAAUGCGGAUAUCAAUGU_0.41/1.11
miRNA Position in PremiRNA:7-27
miRNA annonation:ath-miR2112-3p

50. Degradome supported cutsite(s) in Genome:3961367/3961388
PremiRNA Locus in Genome:>Chr1(3961454-3961365)_minus strand
PremiRNA
sequence:CGCGAGAUAUUAGUGCGGUUCAAUCAAAUAGUCGUCCUCUUAACUCAUGGAGAACGGUGUUGUUCGAUUGAGCCGUGCCAAUAUCACGCG

PremiRNA secondary structure:((((.((((((.(..((((((((((.(((((((((.((((........)))).))))..))))).))))))))))..).)))))).))))
Degradome supported cutsite(s) in premiRNA:89/68
miRNA sequence and Count(AGO1/wt):UUGAGCCGUGCCAAUAUCACG_466.77/47.35
miRNA Position in PremiRNA:68-88
miRNA* sequence and Count(AGO1/wt):AGAUAUUAGUGCGGUUCAAUC_16.74/34.4
miRNA Position in PremiRNA:5-25
miRNA annonation:ath-miR171c/ath-miR171b

51. Degradome supported cutsite(s) in Genome:4820431
PremiRNA Locus in Genome:>Chr1(4820499-4820405)_minus strand

PremiRNA sequence:UCUCCUCUUCUCCAAAUAGUUUAGGUUAGCUGACAUAUAAUAAUUCAAUAGACCCCAGUUUCUCCAGAUAACCUAACUAUUUUGAGAAGAAGUGA
PremiRNA secondary structure:((.(.(((((((.((((((((.((((((.(((............((....))............))).))))))))))))))).))))))).).))
Degradome supported cutsite(s) in premiRNA:70
miRNA sequence and Count(AGO1/wt):UCUUCUCCAAAUAGUUUAGGUU_14.05/3.33
miRNA Position in PremiRNA:6-27
miRNA* sequence and Count(AGO1/wt):CCUAACUAUUUUGAGAAGAAG_12.19/0.92
miRNA Position in PremiRNA:72-92
miRNA annonation:ath-miR830-5p

52. Degradome supported cutsite(s) in Genome:11834118
PremiRNA Locus in Genome:>Chr1(11834159-11834089)_minus strand
PremiRNA sequence:AUUUGAACCUGUCAAUUGGUAUCAAAGCUUCUAAAUCUUUGAAGCUCUGAUACCAAAUGAUGGAAUCAAAU

PremiRNA secondary structure:((((((..((((((.(((((((((.((((((.........)))))).))))))))).))))))..))))))
Degradome supported cutsite(s) in premiRNA:43
miRNA sequence and Count(AGO1/wt):AGCUCUGAUACCAAAUGAUGGAAU_6.82/21.45
miRNA Position in PremiRNA:43-66
miRNA* sequence and Count(AGO1/wt):CCUGUCAAUUGGUAUCAAAGCUUC_0.62/2.96
miRNA Position in PremiRNA:8-31
miRNA annonation:ath-miR829.1

53. Degradome supported cutsite(s) in Genome:23345389/23345408
PremiRNA Locus in Genome:>Chr1(23345506-23345382)_minus strand
PremiRNA sequence:AGUUUUAGGGCGCCUCUCCAUUGGCAGGUCCUUUACUUCCAAAUAUACACAUACAUAUAUGAAUAUCGAAAAUUUCCGAUGAUCGAUUUAUAAAUGACCUGCCAAAGGAGAGUUGCCCUGAAACU
PremiRNA secondary structure:(((((((((((.((((((.((((((((((.((((.......(((((........)))))((.(((((........))))).))......)))).))))))))))).)))))).)))))))))))
Degradome supported cutsite(s) in premiRNA:119/100
miRNA sequence and Count(AGO1/wt):UGCCAAAGGAGAGUUGCCCUG_115.3/61.4
miRNA Position in PremiRNA:100-120
miRNA* sequence and Count(AGO1/wt):UUAGGGCGCCUCUCCAUUGGC_0/0
miRNA Position in PremiRNA:8-28

miRNA annonation:ath-miR399b/ath-miR399c

54. Degradome supported cutsite(s) in Genome:27713234/27713255
PremiRNA Locus in Genome:>Chr1(27713416-27713233)_minus strand
PremiRNA
sequence:GUAGAGCUCCUUAAAGUUCAAACAUGAGUUGAGCAGGGUAAAGAAAAGCUGCUAAGCUAUGGAUCCCAUAAGCCCUAAUCCUUGUAAAGUA
AAAAAGGAUUUGGUUAUAUGGAUUGCAUAUCUCAGGAGCUUUAACUUGCCCUUUAAUGGCUUUUACUCUUCUUUGGAUUGAAGGGAGCUCUAC

PremiRNA secondary
structure:((((((((((((..((((((((...((((.(((((((((((...((((((.((.((.((((.(..(((((((((..(((((((..........))))))).)))).)))))..).)))).)).)).))))))...)))))))......))))..))))...))))))))..))))))))))))

Degradome supported cutsite(s) in premiRNA:184/163
miRNA sequence and Count(AGO1/wt):UUUGGAUUGAAGGGAGCUCUA_25792.98/2736.01
miRNA Position in PremiRNA:163-183
miRNA* sequence and Count(AGO1/wt):GAGCUCCUUAAAGUUCAAACA_1.03/0.92
miRNA Position in PremiRNA:4-24
miRNA annonation:ath-miR159a

55. Degradome supported cutsite(s) in Genome:22930095/22930116
PremiRNA Locus in Genome:>Chr1(22930184-22930090)_minus strand
PremiRNA
sequence:AAAUACGAGAUAUUGGUGCGGUUCAAUCAGAAAACCGUACUCUUUUGUUUUAAAGAUCGGUUUAUUUGAUUGAGCCGUGCCAAUAUCACGC
GUUU
PremiRNA secondary structure:(((..((.((((((((..((((((((((((((((((((...(((((......))))).))))))).)))))))))))))..)))))))).))..)))
Degradome supported cutsite(s) in premiRNA:91/70
miRNA sequence and Count(AGO1/wt):UUGAGCCGUGCCAAUAUCACG_466.77/47.35
miRNA Position in PremiRNA:70-90
miRNA* sequence and Count(AGO1/wt):AGAUAUUGGUGCGGUUCAAUC_42.15/40.32
miRNA Position in PremiRNA:8-28
miRNA annonation:ath-miR171c/ath-miR171b

56. Degradome supported cutsite(s) in Genome:4142352/4142464
PremiRNA Locus in Genome:>Chr2(4142468-4142328)_minus strand

PremiRNA sequence:UAUUCUUCCACAGCUUUCUUGAACUGCAAAACUUCUUCAGAUUUUUUUUUUUUCUUUUGAUAUCUCUUACGCAUAAAAUAGUGAUUUUCUUCAUAUCUCUGCUCGAUUGAUUUGCGGUUCAAUAAAGCUGUGGGAAGAUA

PremiRNA secondary structure:(((..((((((((((((.((((((((((((.(.((..((((...........((....))...................((((......))))...))))...))..).)))))))))))).))))))))))))..)))

Degradome supported cutsite(s) in premiRNA:118/6

miRNA sequence and Count(AGO1/wt):UUCCACAGCUUUCUUGAACUG_4774.31/692.09

miRNA Position in PremiRNA:6-26

miRNA* sequence and Count(AGO1/wt):GUUCAAUAAAGCUGUGGGAAG_92.78/224.72

miRNA Position in PremiRNA:118-138

miRNA annonation:ath-miR396a

57. Degradome supported cutsite(s) in Genome:10676554

PremiRNA Locus in Genome:>Chr2(10676558-10676443)_minus strand

PremiRNA sequence:GAAACUGACAGAAGAGAGUGAGCACACAAAGGCAAUUUGCAUAUCAUUGCACUUGCUUCUCUUGCGUGCUCACUGCUCUUUCUGUCAGAUUCCGGUGCUGAUCUCUUUGGCCUGUC

PremiRNA secondary structure:((..((((((((((((((((((((.(((((((((..((((......)))).))))))...))).))))))))).))).)))))))))....(((.((..(.....)..)))))))

Degradome supported cutsite(s) in premiRNA:6

miRNA sequence and Count(AGO1/wt):UGACAGAAGAGAGUGAGCAC_5994.85/1495.53

miRNA Position in PremiRNA:6-25

miRNA* sequence and Count(AGO1/wt):GCUCACUGCUCUUUCUGUCAGA_6.2/132.06

miRNA Position in PremiRNA:68-89

miRNA annonation:ath-miR156e/ath-miR156f/ath-miR156d/ath-miR156a/ath-miR156c/ath-miR156b

58. Degradome supported cutsite(s) in Genome:3366369

PremiRNA Locus in Genome:>Chr3(3366416-3366349)_minus strand

PremiRNA sequence:UGCUUCUUUGUCUACAAUUUUGGAAAAAGUGAUGACGCCAUUGCUCUUUCCCAAAUGUAGACAAAGCA

PremiRNA secondary structure:(((((...((((((((.(((.(((((.((..(((....)))..)).))))).))))))))))))))))

Degradome supported cutsite(s) in premiRNA:49

miRNA sequence and Count(AGO1/wt):UCCCAAAUGUAGACAAAGCA_235267.58/12735.1

miRNA Position in PremiRNA:49-68

miRNA* sequence and Count(AGO1/wt):CUUUGUCUACAAUUUUGGAAA_25/47.35

miRNA Position in PremiRNA:3-26

miRNA annonation:ath-miR158a

59. Degradome supported cutsite(s) in Genome:3599812
PremiRNA Locus in Genome:>Chr3(3599897-3599787)_minus strand
PremiRNA
sequence:GCUGUUGGAGCAUCAUCAAGAUUCACAAAUCAUCAAGUAUUCGUGUAAAUAAACCCAUUUAUGAUUAGAUUUUUGAUGUAUGUAUGAGAAU
CUUGAUGAUGCUGCAGCUGC
PremiRNA secondary structure:((.((((.((((((((((((((((.((.(((((((((...((.(((((((......)))))))....))..))))))).))...)).)))))))))))))))).)))).))
Degradome supported cutsite(s) in premiRNA:87
miRNA sequence and Count(AGO1/wt):AGAAUCUUGAUGAUGCUGCAG_36.99/16.28
miRNA Position in PremiRNA:87-107
miRNA* sequence and Count(AGO1/wt):GGAGCAUCAUCAAGAUUCACA_0.21/0.37
miRNA Position in PremiRNA:7-27
miRNA annonation:ath-miR172d/ath-miR172c

60. Degradome supported cutsite(s) in Genome:4359010
PremiRNA Locus in Genome:>Chr3(4359207-4359007)_minus strand
PremiRNA
sequence:GUGUGCAGCCAAGGAUGACUUGCCGAUUUAAAUGAUCUUUCUUUAUACUCUAUUAAGACAAUUUAGUUUCAAACUUUUUUUUUUUUUUUU
UUUGAAGGAUUCAGGAAGAAAUUAGGAUAUAUUAUUCCGUAUAAAAUACAAGAUAUAUAAAACCAAAAAGAAAAAGUAACAUGAUCGGCAAGUUGU
CCUUGGCUACACGU
PremiRNA secondary
structure:(((((.(((((((((..((((((((((((((((..((((...............))))..))))).......((((((((((((((((((((((((.....)))))))))))...(((........)))........................))))))))))))).....)))))))))))..))))))))).)))))

Degradome supported cutsite(s) in premiRNA:199
miRNA sequence and Count(AGO1/wt):CAGCCAAGGAUGACUUGCCGA_387.84/50.86
miRNA Position in PremiRNA:6-26
miRNA* sequence and Count(AGO1/wt):CAAGUUGUCCUUGGCUACACG_2.27/0
miRNA Position in PremiRNA:178-198
miRNA annonation:ath-miR169a

61. Degradome supported cutsite(s) in Genome:6244694
PremiRNA Locus in Genome:>Chr3(6244698-6244524)_minus strand
PremiRNA
sequence:GGUUGUUGACAGAAGAUAGAGAGCACUAAGGAUGACAUGCAAGUACAUACAUAUAUAUCAUCACACCGCAUGUGGAUGAUAAAAUAUGUAU

AACAAAUUCAAAGAAAGAGAGGGAGAGAAAGAGAGAGAACCUGCAUCUCUACUCUUUUGUGCUCUCUAUACUUCUGUCACCACC

PremiRNA secondary structure:(((.((.((((((((((((((((((...((((.((......))..((((((((.(((((((.(((.....))))))))))..)))))))).....))))...(((((((..(((((...((.(......)))...))))).)))))))))))))))))).)))))))))).)))

Degradome supported cutsite(s) in premiRNA:6
miRNA sequence and Count(AGO1/wt):UUGACAGAAGAUAGAGAGCAC_0/1656.07
miRNA Position in PremiRNA:6-26
miRNA* sequence and Count(AGO1/wt):GCUCUCUAUACUUCUGUCACC_28.31/379.15
miRNA Position in PremiRNA:152-172
miRNA annonation:ath-miR157b/ath-miR157a/ath-miR157c

62. Degradome supported cutsite(s) in Genome:19659579
PremiRNA Locus in Genome:>Chr3(19659671-19659576)_minus strand
PremiRNA
sequence:UGUCGUCUCGGUUCGCGAUCCACAAGUAAUCUUUUGUGGAGAUUAUGAAAACAAUCAUCACGAGAGGCUACGUGUGGGUGGCAAACAAAGA
CGACA
PremiRNA secondary structure:((((((((..(((.((.(((((((.(((..((((((((..((((.(....).))))..))))))))..))).))))))).)).)))..))))))))
Degradome supported cutsite(s) in premiRNA:94
miRNA sequence and Count(AGO1/wt):UCUCGGUUCGCGAUCCACAAG_2.27/0.18
miRNA Position in PremiRNA:6-26
miRNA* sequence and Count(AGO1/wt):UGUGGGUGGCAAACAAAGACG_0/0
miRNA Position in PremiRNA:73-93
miRNA annonation:ath-miR851-5p

63. Degradome supported cutsite(s) in Genome:369856/369877
PremiRNA Locus in Genome:>Chr4(369996-369851)_minus strand
PremiRNA
sequence:GUUGUGGGGAAUGUUGUUUGGAUCGAGGAUAUCAUAAACGCAUACACAUGUUUAUAUGUUAUGAUGCAUUAUAUGACUGAUGUAAUGUAC
AUAUAUAUACAUACAUGCCACAUGGUAUCGUCGGACCAGGCUUCAUCCCCCUCAAC
PremiRNA secondary structure:((((.((((.(((..((((((.((((.((((((((....((((....((((..((((((.(((.((((((((((.....)))))))))))))))))))))))..))))...)))))))).)))).))))))..))).)))).))))

Degradome supported cutsite(s) in premiRNA:142/121
miRNA sequence and Count(AGO1/wt):UCGGACCAGGCUUCAUCCCCC_19209.65/332.55
miRNA Position in PremiRNA:121-141

miRNA* sequence and Count(AGO1/wt):GGAAUGUUGUUUGGAUCGAGG_0/0.74
miRNA Position in PremiRNA:8-28
miRNA annonation:ath-miR165b/ath-miR165a

64. Degradome supported cutsite(s) in Genome:1535440
PremiRNA Locus in Genome:>Chr4(1535652-1535435)_minus strand
PremiRNA
sequence:UAUACAAUACUACUUUUUCAUCCAUUAAUCCCCUUACAAUGUCGAGUAAACGAAGCAUCUGUCCCCUGGUAUUGUCUUCGAGCUUGGUGUG
UUUUUCUAGCCAGCCCCAAGUUCUCGAGUUGAUCAUUGUUUGUAUUCUGACACAUUAUUUGGGGACGAGAUGUUUUGUUGACUCGAUAUAAGAAGG
GGCUUUAUGGAAGAAAUUGUAGUAUUAUAUA
PremiRNA secondary
structure:((((.((((((((..((((.(((((.((.((((((...((((((((.((((((((((((((((((..(((.((((...((((((((.(((((.....)))..)).))))))))..((((....((.....)).)))).))))...)))..)))))).))))))))))))).)))))))))...)))))).)).))))).))))..))))
))))).))))
Degradome supported cutsite(s) in premiRNA:214
miRNA sequence and Count(AGO1/wt):UAUGGAAGAAAUUGUAGUAUU_711/102.46
miRNA Position in PremiRNA:193-213
miRNA* sequence and Count(AGO1/wt):UACUACUUUUUCAUCCAUUAA_0/0
miRNA Position in PremiRNA:8-28
miRNA annonation:ath-miR447a.2-3p

65. Degradome supported cutsite(s) in Genome:8504145/8504169
PremiRNA Locus in Genome:>Chr4(8504315-8504140)_minus strand
PremiRNA
sequence:UCAAGAUAUCAGAUAUUUCACGAAGAUAUCUGCAUAACAGCUACGAGGAAAUUGUGAUUUUAUCGUUUCACAAACAUCAACAGCUCCAUGG
AUCGUUAUGGACGGAUAAAUCACAAUCACUAUCCUUUUUCUAGCAGCUGUUGAGCAGGUUUCUUCGUGAAUAUCUGGCAUUUUGG

PremiRNA secondary structure:(((((((..((((((((.((((((((.((((((.((((((((.((((((.((((((((((.((((((.....))))).......(.((((((((....)))))))).))))))))))))).....)))))......).)))))))).)))))).))))))))))))))..)))))))

Degradome supported cutsite(s) in premiRNA:172/148
miRNA sequence and Count(AGO1/wt):UUCUUCGUGAAUAUCUGGCAU_2.69/0.37
miRNA Position in PremiRNA:151-171
miRNA* sequence and Count(AGO1/wt):UAUCAGAUAUUUCACGAAGAUA_0/0
miRNA Position in PremiRNA:8-29
miRNA annonation:ath-miR780.2

66. Degradome supported cutsite(s) in Genome:15415511
PremiRNA Locus in Genome:>Chr4(15415511-15415427)_minus strand
PremiRNA sequence:CUGACAGAAGAGAGUGAGCACACAAAGGCACUUUGCAUGUUCGAUGCAUUUGCUUCUCUUGCGUGCUCACUGCUCUAUCUGUCAG

PremiRNA secondary structure:(((((((((((((((((((((.((((((((...(((((.....)))))..)))))...))).))))))))).)))).))))))))
Degradome supported cutsite(s) in premiRNA:2
miRNA sequence and Count(AGO1/wt):UGACAGAAGAGAGUGAGCAC_5994.85/1495.53
miRNA Position in PremiRNA:2-21
miRNA* sequence and Count(AGO1/wt):GCUCACUGCUCUAUCUGUCAGA_6.41/337.72
miRNA Position in PremiRNA:65-86
miRNA annonation:ath-miR156e/ath-miR156f/ath-miR156d/ath-miR156a/ath-miR156c/ath-miR156b

67. Degradome supported cutsite(s) in Genome:1188211
PremiRNA Locus in Genome:>Chr5(1188304-1188206)_minus strand
PremiRNA
sequence:UGUAGGCGCAGCACCAUUAAGAUUCACAUGGAAAUUGAUAAAUACCCUAAAUUAGGGUUUUGAUAUGUAUAUGAGAAUCUUGAUGAUGCUG
CAUCAACA
PremiRNA secondary structure:(((.(..((((((.(((((((((((.((((...((...((((.((((((...))))))))))..))...)))).)))))))))))).))))))..).)))
Degradome supported cutsite(s) in premiRNA:95
miRNA sequence and Count(AGO1/wt):AGAAUCUUGAUGAUGCUGCAU_1100.08/929.76
miRNA Position in PremiRNA:74-94
miRNA* sequence and Count(AGO1/wt):GCAGCACCAUUAAGAUUCACA_1.45/13.13
miRNA Position in PremiRNA:8-28
miRNA annonation:ath-miR172a/ath-miR172b-3p

68. Degradome supported cutsite(s) in Genome:2634936/2634957
PremiRNA Locus in Genome:>Chr5(2635022-2634934)_minus strand
PremiRNA
sequence:CGCUGGAGGCAGCGGUUCAUCGAUCUCUUCCUGUGAACACAUUAAAAAUGUAAAAGCAUGAAUAGAUCGAUAAACCUCUGCAUCCAGCG

PremiRNA secondary structure:(((((((.((((.((((.((((((((.(((.(((....((((.....))))....))).))).)))))))).)))).)))).)))))))
Degradome supported cutsite(s) in premiRNA:88/67
miRNA sequence and Count(AGO1/wt):UCGAUAAACCUCUGCAUCCAG_1405.06/136.5

miRNA Position in PremiRNA:67-87
miRNA* sequence and Count(AGO1/wt):GGAGGCAGCGGUUCAUCGAUC_0.21/0.18
miRNA Position in PremiRNA:5-25
miRNA annonation:ath-miR162b/ath-miR162a

69. Degradome supported cutsite(s) in Genome:3456666/3456733
PremiRNA Locus in Genome:>Chr5(3456737-3456640)_minus strand
PremiRNA sequence:GAAGUUGACAGAAGAGAGUGAGCACACAAAGGGGAAGUUGUAUAAAAGUUUUGUAUAUGGUUGCUUUUGCGUGCUCACUCUCUUUUUGUCAUAACUUC
PremiRNA secondary structure:(((((((((((((((((((((((((.((((((.((...(((((((.....)))))))...)).)))))).)))))))))))).))))))))..)))))
Degradome supported cutsite(s) in premiRNA:73/6
miRNA sequence and Count(AGO1/wt):UGACAGAAGAGAGUGAGCAC_5994.85/1495.53
miRNA Position in PremiRNA:6-25
miRNA* sequence and Count(AGO1/wt):GCUCACUCUCUUUUUGUCAUA_1.03/13.69
miRNA Position in PremiRNA:73-93
miRNA annonation:ath-miR156e/ath-miR156f/ath-miR156d/ath-miR156a/ath-miR156c/ath-miR156b

70. Degradome supported cutsite(s) in Genome:7740610/7740631
PremiRNA Locus in Genome:>Chr5(7740705-7740605)_minus strand
PremiRNA sequence:UGAAGUCGCUGGAGGCAGCGGUUCAUCGAUCAAUUCCUGUGAAUAUUUAUUUUUGUUUACAAAAGCAAGAAUCGAUCGAUAAACCUCUGCAUCCAGCGCUG
PremiRNA secondary structure:(..((.(((((((.((((.((((.(((((((.((((.((((((((........)))))))).......)))).))))))).)))).)))).))))))))))
Degradome supported cutsite(s) in premiRNA:97/76
miRNA sequence and Count(AGO1/wt):UCGAUAAACCUCUGCAUCCAG_1405.06/136.5
miRNA Position in PremiRNA:76-96
miRNA* sequence and Count(AGO1/wt):GGAGGCAGCGGUUCAUCGAUC_0.21/0.18
miRNA Position in PremiRNA:11-31
miRNA annonation:ath-miR162b/ath-miR162a

71. Degradome supported cutsite(s) in Genome:16660490
PremiRNA Locus in Genome:>Chr5(16660998-16660464)_minus strand

PremiRNA
sequence:AAAAGCCAUAACUCCUUCAUUUUCUUUAGACAUCUCUUCUUCUCUCAUCUCUCUUUUCUUCUCUCUUUUCCUCACAUAAACUCUCUUUUUUU
ACUAUUAAAUCCAUAUGGUACCUCAAAUUAAUCUAUGGUCAUCUAGGGUUAUCUUGAAGAUUAGAAUUGAUUCUAGCACGCACAGAGAGGAAGAUC
AUUGCAUCCAGAAUCACAAACAUGGCCUAUCUUUUAUCUUUUCUUUUUGAUCUAAGUCACUGUUUUAUGCUAUAUAUAGUAUAAUCAAAUUCUUUA
CAUGUGCUUGUAUGUAUGCGUAUAUAUAGUAACGGAAUUGUUAAUAUGCUUAUAGAUGUUGAGUUGGUGGAGGAAGAGAGCUUUCUUCGGUCCACU
CAUGGAGUAAUAUGUGAGAUUUAAUUGACUCUCGACUCAUUCAUCCAAAUACCAAAUGAAAGAAUUUGUUCUCAUAUGGUAAAUGAAUGAAUGAUG
CGAGAGACAAAUUGAGUCUUCACUUCUCUAUGCUUGGACUGAAGGGAGCUCCCUAUUUU

PremiRNA secondary
structure:((((........((((((((..(((..((((((((((((((((......((..((((..((..........................((((((.........))))))..((((..((((((.(((....)))))))))..)))).))..))))..)).....(....)..))))))))))..(((.((((((........((((((((..(((..
...............)))....)))).))))))..((((((((((((((((...........((((......))))..))))).)))))))))))..)))..)))....))).....))))))))))..)))))))))...(((((..((((((((((..(((((((......((((((((((((..((((((..((((((((.((..(((((.((((..(((....)
))))))).)))))..)).)))))))))..))))))...)))))))))).))))..)))))))..)))))))))))..)))))....))))

Degradome supported cutsite(s) in premiRNA:510

miRNA sequence and Count(AGO1/wt):UUGGACUGAAGGGAGCUCCCU_4553.01/736.11

miRNA Position in PremiRNA:510-530

miRNA* sequence and Count(AGO1/wt):AGAGCUUUCUUCGGUCCACUC_28.93/15.91

miRNA Position in PremiRNA:361-381

miRNA annonation:ath-miR319b/ath-miR319a

72. Degradome supported cutsite(s) in Genome:16775545

PremiRNA Locus in Genome:>Chr5(16775662-16775520)_minus strand

PremiRNA
sequence:UUGAGGGGAAUGUUGUCUGGCACGAGGCCCUUAACUUAGAUCUAUAUUUGAUUAUAUAUAUAUGUCUCUUCUUUAUUCAUUAGUCUAUACA
UGAAUGAUCAUUUUACGGUUAAUGACGUCGGACCAGGCUUCAUUCCCCUCAA

PremiRNA secondary structure:((((((((((((..((((((..(((.(.(.((((((.((((.(((((..........))))).)))).....((((((((((.....)).))))))))).........)))))).).).)))..))))))..))))))))))))

Degradome supported cutsite(s) in premiRNA:119

miRNA sequence and Count(AGO1/wt):UCGGACCAGGCUUCAUUCCCC_42640.47/1448.18

miRNA Position in PremiRNA:119-139

miRNA* sequence and Count(AGO1/wt):GGAAUGUUGUCUGGCACGAGG_0.21/13.69

miRNA Position in PremiRNA:7-27

miRNA annonation:ath-miR166b/ath-miR166f/ath-miR166a/ath-miR166g/ath-miR166c/ath-miR166d/ath-miR166e

73. Degradome supported cutsite(s) in Genome:18358894

PremiRNA Locus in Genome:>SChr5(18358898-18358800)_minus strand

PremiRNA
sequence:CGGAUUCGCUUGGUGCAGGUCGGGAACUGAUUGGCUGACACCGACACGUGUCUUGUCAUGGUUGGUUUGUGAGCUCCCGUCUUGUAUCAAC
UGAAUCGG
PremiRNA secondary structure:(.((((((.((((((((((.(((((........(((.((((((((.((((......))))))))))..)).)))))))).)))))))))).)))))).)
Degradome supported cutsite(s) in premiRNA:6
miRNA sequence and Count(AGO1/wt):UCGCUUGGUGCAGGUCGGGAA_1250.3/166.64
miRNA Position in PremiRNA:6-26
miRNA* sequence and Count(AGO1/wt):CCCGUCUUGUAUCAACUGAAU_0.62/46.05
miRNA Position in PremiRNA:76-96
miRNA annonation:ath-miR168b/ath-miR168a

74. Degradome supported cutsite(s) in Genome:771384/771406
PremiRNA Locus in Genome:>Chr2(771517-771382)_minus strand
PremiRNA
sequence:GAAGACACUGAAGGACCUAAACUAACAAAGGUAAACGGCUCAGUGUGCGGGGUAUUACACUCGGUUUAAUGUCUGAAUGCGAUAAUCCGCA
CGAUGAUCUCUUUAUCUUUGUUUGUUUAGGUCCCUUAGUUUCUUC
PremiRNA secondary structure:(((((.(((((.((((((((((.(((((((((((((.((.(((.(((((((..(((..((.((((........)))).))..)))..))))))).))).)).))))))))))))).)))))))))).))))).)))))
Degradome supported cutsite(s) in premiRNA:135/113
miRNA sequence and Count(AGO1/wt):CACUGAAGGACCUAAACUAAC_11.57/1.66
miRNA Position in PremiRNA:6-26
miRNA* sequence and Count(AGO1/wt):UUGUUUAGGUCCCUUAGUUUC_17.36/5.92
miRNA Position in PremiRNA:113-133
miRNA annonation:ath-miR840-5p

Supplementary table S3a. Discovery of novel miRNAs in flowers of *Arabidopsis*

1. Degradome supported cutsite(s) in Genome:17825730/17825821
PremiRNA Locus in Genome:>Chr1(17825725-17825824)_plus strand
PremiRNA
sequence:AUGCAUUGAAAGUGACUACAUCGGGGUUCCGAUUUUUUUUGUUCUUCAUAUGAUGAAGCGGAAACAGUAAUCAACCCUGGUUUAGUCACUU
UCACUGCAU
PremiRNA secondary structure:(((((.((((((((((((.(((((((((..((((....((((((((((...))))))....))))).)))))))))))).))))))))))).)))))
Degradome supported cutsite(s) in premiRNA:6/97
miRNA sequence and Count(AGO1/wt):UUGAAAGUGACUACAUCGGGG_4064.43/935.77
miRNA Position in PremiRNA:6-26
miRNA* sequence and Count(AGO1/wt):UGGUUUAGUCACUUUCACUGC_1.25/0.2
miRNA Position in PremiRNA:77-97
miRNA annonation:miRNA-1

2. Degradome supported cutsite(s) in Genome:19415175
PremiRNA Locus in Genome:>Chr2(19415069-19415180)_plus strand
PremiRNA
sequence:GUAUUACAUGUUUUGUGCUUGAAUCUAAUUCAACAGGCUUUAUGUAAGAGAUUCUUUAACAAUUCCUAUAAUCUUUGUUGUUGGAUUAGA
UUCACGCACAAACUCGUAAUCU
PremiRNA secondary structure:(.(((((..((((.((((.((((((((((((((((.((.....)).(((((((................)))))))..)))))))))))))))).))))))))..))))).)
Degradome supported cutsite(s) in premiRNA:107
miRNA sequence and Count(AGO1/wt):UUAGAUUCACGCACAAACUCGU_558.78/194.36
miRNA Position in PremiRNA:86-107
miRNA* sequence and Count(AGO1/wt):AUGUUUUGUGCUUGAAUCUAAUU_0/0
miRNA Position in PremiRNA:8-30
miRNA annonation:miRNA-2

3. Degradome supported cutsite(s) in Genome:15371933
PremiRNA Locus in Genome:>Chr3(15371931-15372073)_plus strand
PremiRNA
sequence:UCAAAUGAGUUGAUGGGUCAAAUGAGUUAUUGGGUCAAUUGGUUUGAUGAAUAAAAUGAGUUGGGUUGUAAUGAUUAAUGGUUUCAAUGG
UUUACCCAAUUAACCCAUUAACCCAUAACUCAUUUAACCCUAAACUCAUUUGA
PremiRNA secondary structure:((((((((((...((((.(((((((((((.((((.(((.((((.............((((((((...(((.(((.........))).))).))))))))))))).))).))))))))))))))))).)))))..))))))))))
Degradome supported cutsite(s) in premiRNA:3

miRNA sequence and Count(AGO1/wt):AAAUGAGUUGAUGGGUCAAAUGAG_0.25/1.61
miRNA Position in PremiRNA:3-26
miRNA* sequence and Count(AGO1/wt):UUUAACCCUAAACUCAUUUGAUU_0.5/0.81
miRNA Position in PremiRNA:121-143
miRNA annonation:miRNA-3

4. Degradome supported cutsite(s) in Genome:12138948
PremiRNA Locus in Genome:>Chr5(12138921-12138980)_plus strand
PremiRNA sequence:GGAGUCUUCUUUUCUUCCUUGUGCUCGAGUGUCUGGUUGAGUGCGGAAUGAAGAAGGCUCU
PremiRNA secondary structure:((((((((((((..((((..(..(((((.(....).)))))..))))).))))))))))))
Degradome supported cutsite(s) in premiRNA:28
miRNA sequence and Count(AGO1/wt):UUGAGUGCGGAAUGAAGAAGGC_2/0.2
miRNA Position in PremiRNA:37-58
miRNA* sequence and Count(AGO1/wt):UCUUUUCUUCCUUGUGCUCGAG_0/0
miRNA Position in PremiRNA:6-30
miRNA annonation:miRNA-4

5. Degradome supported cutsite(s) in Genome:29911709
PremiRNA Locus in Genome:>Chr1(29911713-29911101)_minus strand
PremiRNA
sequence:UACUUCGGCGAUGAUGAUGAAACAAGAUUACCAUCAAACUUUGAGAGGUUUUUCGCUCGUUGAUGUAAACUUCUUUUUAUAAUCUGAGCCU
AGUUUGUUUGUUUGAAAUCUUAUGUGUUACAUGUUAUAUGAAAUCGUUUUGGUGAUAGGAAAGGCAACAAAUUAGAGUCUCUGAUUCACUUAUGUC
AAUAGUGGAUAAAGCGCUGUACGUUGCCGAGAUAGAUAUGGCGAUAUUCUGGUACUUGGACUAGUUUUGAUAGUUCCAUGUGCAUUCAAAAGCCCU
UACAUGUUUCUCUCAACUUAUUCCCAUUAGUAUAUGUAUUUUGAUUUGGUAAAGGCAACAAAUUAGAGUCUCUGAUUCACUUAUUCAAUAGUGGAU
AAAGCGCUGUACGUUGCCAAUAAUUACUUGGUCCAAAGAUCUCCCAAUCGGCUUUCAGUCUUAUUUUAGCCGAUAUGAUCGAUGUUCCUAAAUUAUA
GAUUUUGUUACGUGUUUUCAUAUAGCUGAGCUGACCAUAUCAUGCUAUUUACCAAAUCUUUUUGAUCAAUUACCUGACACAUUGACAGACGAUCGAU
UUUUAGACACUGAAAUAUGGGCCGAAAUUUUGAAGGAGUA
PremiRNA secondary
structure:((((((..(((.(((.((((((.(((.((((.(((((....((((.((....)).))))))))))))).)))..)))))).))).(.(((((..(((.(((((((...((..((((((((...((((((((((.((.....((((((((..((((((.((((((((.((((((((..........((((((.......))))))..((
((((((((.(((((((((.........((((.(((((((.......))).)))).))))........)))....))))))).))))........(((..((((....))))......)))....)))))).)))))))).))))))))..........((((((.......))))))..)))))).((((((....((..((..((((.....(((((((............
..)))))))..))))..))..))..))))))...))))))))).)).)))))))))))..((((((.....))).)))......((((....))))..........)))))))).))))))))).))).((((((...))))))..))))))......)))..))))))
Degradome supported cutsite(s) in premiRNA:6
miRNA sequence and Count(AGO1/wt):CGGCGAUGAUGAUGAAACAAGAU_5.76/0
miRNA Position in PremiRNA:6-28

miRNA* sequence and Count(AGO1/wt):CUUCUUUUAUAAUCUGAGCCUA_0/0
miRNA Position in PremiRNA:70-610
miRNA annonation:miRNA-5

6. Degradome supported cutsite(s) in Genome:23988505
PremiRNA Locus in Genome:>Chr5(23988591-23988477)_minus strand
PremiRNA sequence:UGCUGAUGCAGCAUCAUCAAGAUUCCCACUCGAUAAGGAACCCUUUUCUGCGGAUCGAGAGGCGAAAGCAAAGGGAACCACAUCUCUUGUGAAUCUUAAUGGUGCUGCAUCUGCG
PremiRNA secondary structure:(((.(((((((((((((.(((((((((.((((((.(((((....)))))....))))))..((....))...))))..((((.....)))).))))).))))))))))))).)))
Degradome supported cutsite(s) in premiRNA:88
miRNA sequence and Count(AGO1/wt):UGAAUCUUAAUGGUGCUGCAU_2/0.6
miRNA Position in PremiRNA:90-110
miRNA* sequence and Count(AGO1/wt):UGCAGCAUCAUCAAGAUUCCC_2.25/0.2
miRNA Position in PremiRNA:8-82
miRNA annonation:miRNA-6

7. Degradome supported cutsite(s) in Genome:6220763/6220784
PremiRNA Locus in Genome:>Chr1(6220685-6220789)_plus strand
PremiRNA sequence:UGAAGUAAAGCUGCUAAGCUAUGGAUCCCAUAAGCCUUAUCAAAUUCAAUAUAAUUGAUGAUAAGGUUUUUUUUAUGGAUGCCAUAUCUCAGGAGCUUUCACUUA
PremiRNA secondary structure:(((.((((((((.((.((.(((((...((((((((((((((((...(((((...)))))))))))))))))))).....))))))...))))).)).)).)))))).)))))
Degradome supported cutsite(s) in premiRNA:79/100
miRNA sequence and Count(AGO1/wt):UGCCAUAUCUCAGGAGCUUUC_0.25/0.2
miRNA Position in PremiRNA:80-100
miRNA* sequence and Count(AGO1/wt):AAGCUGCUAAGCUAUGGAUCC_0.25/0.6
miRNA Position in PremiRNA:8-28
miRNA annonation:miRNA-7

8. Degradome supported cutsite(s) in Genome:8770553
PremiRNA Locus in Genome:>Chr5(8770544-8770622)_plus strand
PremiRNA sequence:UCAAAUUAAAUGAGUUAUGGGUUGACCCAACUCAUUUUGUUAAAUGGGUUGGGUCAACCCAUAACUCAUUUAACCCUAA

PremiRNA secondary structure:.....((((((((((((((((((((((((((((((((....))))))))))))))))))))))))))))))))......
Degradome supported cutsite(s) in premiRNA:10
miRNA sequence and Count(AGO1/wt):UAAAUGAGUUAUGGGUUGACCC_1/0
miRNA Position in PremiRNA:2-28
miRNA* sequence and Count(AGO1/wt):UCAACCCAUAACUCAUUUAAC_0.5/0.2
miRNA Position in PremiRNA:54-74
miRNA annonation:miRNA-8

9. Degradome supported cutsite(s) in Genome:20772272
PremiRNA Locus in Genome:>Chr1(20772225-20772297)_plus strand
PremiRNA sequence:UGCUUCUUUGUCUACACUUUUGGAAAAGGUGAUGAUAUCAUUGCUUUUCCCCAAAUGUAGACAAAGCAAUACC

PremiRNA secondary structure:))...(((((((((((..(((((.(((((..(((....)))..)))))..)))))))))))))))).....))
Degradome supported cutsite(s) in premiRNA:48
miRNA sequence and Count(AGO1/wt):UCCCCAAAUGUAGACAAAGCA_84.62/20.75
miRNA Position in PremiRNA:48-68
miRNA* sequence and Count(AGO1/wt):CUUUGUCUACACUUUUGGAAA_0.25/0.4
miRNA Position in PremiRNA:6-26
miRNA annonation:miRNA-9

10. Degradome supported cutsite(s) in Genome:12767160
PremiRNA Locus in Genome:>Chr2(12767153-12767272)_plus strand
PremiRNA
sequence:CUUAUACAUAUCGAAACCAAAUGAGGUCAAACUUAUUUUUUUAUUUUCUUUUUCUUUUUUUUUUGUUGCCCAAAAAAAAUAAAUAUAAGUU
UGACCUCAUUUGGUUUCGAUAUGUAUAAG
PremiRNA secondary structure:((((((((((((((((((((((((((((((((((((...((((((((.............(((((.....))))))))))))).))))))))))))))))))))))))))))))))))))
Degradome supported cutsite(s) in premiRNA:8
miRNA sequence and Count(AGO1/wt):CCUCAUUUGGUUUCGAUAUGU_0/1.21
miRNA Position in PremiRNA:95-115
miRNA* sequence and Count(AGO1/wt):AUAUCGAAACCAAAUGAGGUC_21.78/16.31
miRNA Position in PremiRNA:8-28
miRNA annonation:miRNA-10

11. Degradome supported cutsite(s) in Genome:687897

PremiRNA Locus in Genome:>Chr4(687895-687964)_plus strand
PremiRNA sequence:UGAAUAGCCUCCGAAUAACGGGAAUGUGAAAUAUAGGAACACCUUCUUGUUCAGACAGAGGCUUAUAUCA

PremiRNA secondary structure:(((..((((((.(..(((((((((.(((...........))).)))))))).)..).))))))....)))
Degradome supported cutsite(s) in premiRNA:3
miRNA sequence and Count(AGO1/wt):AUAGCCUCCGAAUAACGGGAA_0.25/0
miRNA Position in PremiRNA:4-24
miRNA* sequence and Count(AGO1/wt):CUUGUUCAGACAGAGGCUUAUA_0/0
miRNA Position in PremiRNA:46-67
miRNA annonation:miRNA-11

12. Degradome supported cutsite(s) in Genome:23988566
PremiRNA Locus in Genome:>Chr5(23988478-23988590)_plus strand
PremiRNA
sequence:GCAGAUGCAGCACCAUUAAGAUUCACAAGAGAUGUGGUUCCCUUUGCUUUCGCCUCUCGAUCCGCAGAAAAGGGUUCCUUAUCGAGUGGGA
AUCUUGAUGAUGCUGCAUCAGC
PremiRNA secondary structure:((.(((((((((.(((((((((((.((...((((.((..((((((((..(((.....)))...)))...)))))..)).))))...)).))))))))))).))))))))).))
Degradome supported cutsite(s) in premiRNA:89
miRNA sequence and Count(AGO1/wt):UGCAGCACCAUUAAGAUUCAC_2.5/0.2
miRNA Position in PremiRNA:6-26
miRNA* sequence and Count(AGO1/wt):GAAUCUUGAUGAUGCUGCAU_79.86/12.69
miRNA Position in PremiRNA:90-110
miRNA annonation:miRNA-12

13. Degradome supported cutsite(s) in Genome:16432049
PremiRNA Locus in Genome:>Chr1(16432023-16432076)_plus strand
PremiRNA sequence:CCCAUCAGAACUCCGCAGUUAAGCGCGCUUGGGCGAGAGUAGUACUAGAAUGGG
PremiRNA secondary structure:((((((((.(((((((...((((....)))).))).))))....)).).)))))
Degradome supported cutsite(s) in premiRNA:27
miRNA sequence and Count(AGO1/wt):CAGAACUCCGCAGUUAAGC_0/1.81
miRNA Position in PremiRNA:6-24
miRNA* sequence and Count(AGO1/wt):CUUGGGCGAGAGUAGUACUAG_2.5/3.22
miRNA Position in PremiRNA:29-50
miRNA annonation:miRNA-13

14. Degradome supported cutsite(s) in Genome:22165414
PremiRNA Locus in Genome:>Chr1(22165407-22165553)_plus strand
PremiRNA
sequence:AGAGUUUUAAAAGAUUUACAAGGGAUUUUAAAAAGUUUUAAAAAGAUUUACAAGAGUUUUUAAAGGGUCUUCUUGUAAAUUCUUUAAGAA
ACUCUUGUACAUCUUUUAAAACCCUUUUAAAAUCUCUUGUAAAUCUUUUAAAACCCU
PremiRNA secondary structure:((.((((((((((((((((((((((((((((((.(((((((((.(((.((((((((((((((((((((..........))))))).))))))))))))).))))))))))))..)))))))))))))))))))))))))))))).))

Degradome supported cutsite(s) in premiRNA:8
miRNA sequence and Count(AGO1/wt):UUUUAAAAGAUUUACAAGGGAUUU_3/1.61
miRNA Position in PremiRNA:5-28
miRNA* sequence and Count(AGO1/wt):AUCUCUUGUAAAUCUUUUAAAAC_0/0.2
miRNA Position in PremiRNA:122-145
miRNA annonation:miRNA-14

15. Degradome supported cutsite(s) in Genome:16102366
PremiRNA Locus in Genome:>Chr3(16102362-16102418)_plus strand
PremiRNA sequence:AUGAUUUACAAGGGAUUUUAAAAGUGUCUACAUGGUUUACAAUCGUUUUUAAAGCAU
PremiRNA secondary structure:(((.((((.(((.((((.((((.(((....)))..)))).)))).))).)))).)))
Degradome supported cutsite(s) in premiRNA:5
miRNA sequence and Count(AGO1/wt):UUUACAAUCGUUUUUAAAGCA_0.5/0.2
miRNA Position in PremiRNA:36-56
miRNA* sequence and Count(AGO1/wt):UUUACAAGGGAUUUUAAAA_0.75/0
miRNA Position in PremiRNA:4-24
miRNA annonation:miRNA-15

16. Degradome supported cutsite(s) in Genome:16159377
PremiRNA Locus in Genome:>Chr3(16159338-16159398)_plus strand
PremiRNA sequence:AGCGGUUGAGUUCCCGAAGUUGUCUCUUGUAGUUGCUGAUGACUUUUAGAACACGAUAGCU
PremiRNA secondary structure:(((.((((.((((..(((((..((....((....)).))..)))))..)))).)))).)))
Degradome supported cutsite(s) in premiRNA:40
miRNA sequence and Count(AGO1/wt):UGAUGACUUUUAGAACACGAU_7.26/2.01
miRNA Position in PremiRNA:37-57
miRNA* sequence and Count(AGO1/wt):UGAGUUCCCGAAGUUGUCUCU_0.5/0.2

miRNA Position in PremiRNA:7-27
miRNA annonation:miRNA-16

17. Degradome supported cutsite(s) in Genome:8858956
PremiRNA Locus in Genome:>Chr4(8858895-8858978)_plus strand
PremiRNA sequence:CAACUUUAUUAGUCAAGUUUUAAAUUGUGGCCAUUUUAAACAUCAAGUUCAUAUGUGGCCAUUUAAAACAUGAACUAAACGUUG

PremiRNA secondary structure:((((....(((((((.(((((((...((((((((....(((.....))).....))))))))))))))).)).)))))..))))
Degradome supported cutsite(s) in premiRNA:62
miRNA sequence and Count(AGO1/wt):UUUAAAACAUGAACUAAACGU_1.25/0.6
miRNA Position in PremiRNA:62-82
miRNA* sequence and Count(AGO1/wt):UUUAUUAGUCAAGUUUUAAAUUGU_0.25/0.2
miRNA Position in PremiRNA:5-29
miRNA annonation:miRNA-17

18. Degradome supported cutsite(s) in Genome:3752550
PremiRNA Locus in Genome:>Chr5(3752520-3752633)_plus strand
PremiRNA
sequence:AGCUUCAUCGCAAUGGCUAUGGAUCUUCUUCCUCAGUUUCUGCAUACUGACAUGUCAUGCAUCGGUAUGUGAGGAUUUCUCAUGAGGGACU
CUUUCAUUGGUUGGUGAUAGGCU
PremiRNA secondary structure:(((((.(((((...(((((((((.....(((((((..((((((((((((..((.....)))))))))))))).)))).......)))))))....)))).))))).))))))))))
Degradome supported cutsite(s) in premiRNA:31
miRNA sequence and Count(AGO1/wt):UCUUUCAUUGGUUGGUGAUAG_1.75/0
miRNA Position in PremiRNA:91-111
miRNA* sequence and Count(AGO1/wt):AUCGCAAUGGCUAUGGAUCUUC_1.5/1.01
miRNA Position in PremiRNA:7-28
miRNA annonation:miRNA-18

19. Degradome supported cutsite(s) in Genome:8770553
PremiRNA Locus in Genome:>Chr5(8770549-8770616)_plus strand
PremiRNA sequence:UUAAAUGAGUUAUGGGUUGACCCAACUCAUUUUGUUAAAUGGGUUGGGUCAACCCAUAACUCAUUUAA
PremiRNA secondary structure:((((((((((((((((((((((((((((((((....))))))))))))))))))))))))))))))))
Degradome supported cutsite(s) in premiRNA:5
miRNA sequence and Count(AGO1/wt):UAAAUGAGUUAUGGGUUGACCC_1/0

miRNA Position in PremiRNA:2-23
miRNA* sequence and Count(AGO1/wt):UCAACCCAUAACUCAUUUAAC_0.5/0.2
miRNA Position in PremiRNA:48-69
miRNA annonation:miRNA-19

20. Degradome supported cutsite(s) in Genome:12295343
PremiRNA Locus in Genome:>Chr5(12295232-12295369)_plus strand
PremiRNA
sequence:GAGUGCAAAGGGUAGAGUUAAGUUCUUGUAUUGGGACUGGAGUUGGGAUUACCAUUACAGCUUCAUUGUUAUACUCUGGGUAGAUGGGAU
CUUAUCUCUGUAUGCAUAGCUUGGGACUUACCUUUAGCAUUCUACUAA
PremiRNA secondary structure:((((((.((((((.((((((((((..(((((....((.((((.(((((((.(((((...(((.((..(.....)..))))).))))))))))))).)))))))))))).))))).)))))..))))))))))).))))))......
Degradome supported cutsite(s) in premiRNA:112
miRNA sequence and Count(AGO1/wt):UGGGACUUACCUUUAGCAUUCU_2.75/0.81
miRNA Position in PremiRNA:112-133
miRNA* sequence and Count(AGO1/wt):UGCAAAGGGUAGAGUUAAGUUC_0.25/0
miRNA Position in PremiRNA:2-22
miRNA annonation:miRNA-20

21. Degradome supported cutsite(s) in Genome:16661145
PremiRNA Locus in Genome:>Chr5(16661213-16661123)_minus strand
PremiRNA
sequence:UAAUUGACUCUCGACUCAUUCAUCCAAAUACCAAAUGAAAGAAUUUGUUCUCAUAUGGUAAAUGAAUGAAUGAUGCGAGAGACAAAUUGA

PremiRNA secondary structure:...(((.((((((..((((((((.((..(((((.((((..(((....))))))).)))))..)).))))))))..)))))).)))....)
Degradome supported cutsite(s) in premiRNA:69
miRNA sequence and Count(AGO1/wt):AAUGAAUGAUGCGAGAGACAA_40.56/20.95
miRNA Position in PremiRNA:69-86
miRNA* sequence and Count(AGO1/wt):GACUCUCGACUCAUUCAUCCA_1.25/7.45
miRNA Position in PremiRNA:6-22
miRNA annonation:miRNA-21

22. Degradome supported cutsite(s) in Genome:22322724
PremiRNA Locus in Genome:>Chr5(22322903-22322720)_minus strand

PremiRNA sequence:AAGCCUUGAUGAUUCGACAAAGUGAAGGGUUUGGUCUUCAGAAAGUUUUCUUGCAAGGUUCAAGAACGGAUCCUCAAAAUUCAAACUGUUUCUCACCAAUUUGAAUUGUGAAGAUCCGUUCUUGAACCUUGGAAGAAAACUUUCUGGAGACCAAACCCUUCACUUUGUCGAGUCACCAAGGCUU

PremiRNA secondary structure:(((((((.((((((((((((((((((((((((((((((((((((((.((((((((((((((((((.(((.((((((((.((........)).)))))))).))).))))))))))))))))).)))))))))))))))))))))))))))))))))))))).)))))))

Degradome supported cutsite(s) in premiRNA:181

miRNA sequence and Count(AGO1/wt):UUGAUGAUUCGACAAAGUGAA_492.94/77.34

miRNA Position in PremiRNA:6-26

miRNA* sequence and Count(AGO1/wt):CACUUUGUCGAGUCACCAAGG_0/3.02

miRNA Position in PremiRNA:161-181

miRNA annonation:miRNA-22

23. Degradome supported cutsite(s) in Genome:771406

PremiRNA Locus in Genome:>Chr2(771517-771382)_minus strand

PremiRNA sequence:GAAGACACUGAAGGACCUAAACUAACAAAGGUAAACGGCUCAGUGUGCGGGGUAUUACACUCGGUUUAAUGUCUGAAUGCGAUAAUCCGCACGAUGAUCUCUUUAUCUUUGUUUGUUUAGGUCCCUUAGUUUCUUC

PremiRNA secondary structure:(((((.(((((.((((((((((.(((((((((((.((.(((.(((((((..(((..((.((((........)))).))..)))..))))))).))).)).))))))))))).)))))))))).))))).)))))

Degradome supported cutsite(s) in premiRNA:113

miRNA sequence and Count(AGO1/wt):CACUGAAGGACCUAAACUAAC_9.26/3.22

miRNA Position in PremiRNA:6-26

miRNA* sequence and Count(AGO1/wt):UUGUUUAGGUCCCUUAGUUUC_34.05/9.47

miRNA Position in PremiRNA:113-133

miRNA annonation:miRNA-23

24. Degradome supported cutsite(s) in Genome:25784115

PremiRNA Locus in Genome:>Chr5(25784115-25784187)_plus strand

PremiRNA sequence:GGUUUCGUGGUGUAGUUGGUUAUCACGUCAGUCUAACACACUGAAGGUCUCCGGUUCGAACCCGGGCGAAGCC

PremiRNA secondary structure:(((((((((((..((..((..(((...(((((.......))))).)))..))..))...)))...))))))))

Degradome supported cutsite(s) in premiRNA:1

miRNA sequence and Count(AGO1/wt):GGUUUCGUGGUGUAGUUGGU_0/1.01

miRNA Position in PremiRNA:1-20

miRNA* sequence and Count(AGO1/wt):UCCGGUUCGAACCCGGGCGAAGCC_0.25/0

miRNA Position in PremiRNA:52-75

miRNA annonation:miRNA-24

Supplementary table S3b. Discovery of novel miRNAs in leaves of *Arabidopsis*

1. Degradome supported cutsite(s) in Genome:17825730/17825821

PremiRNA Locus in Genome:>Chr1(17825725-17825824)_plus strand

PremiRNA sequence:AUGCAUUGAAAGUGACUACAUCGGGGUUCCGAUUUUUUUUGUUCUUCAUAUGAUGAAGCGGAAACAGUAAUCAACCCUGGUUUAGUCACUUUCACUGCAU

PremiRNA secondary structure:(((((.((((((((((((.(((((((((..((((....(((((((((((...))))))....))))).))))))))))))).)))))))))))).)))))

Degradome supported cutsite(s) in premiRNA:6/97

miRNA sequence and Count(AGO1/wt):UUGAAAGUGACUACAUCGGGG_12450.23/1338.33

miRNA Position in PremiRNA:6-26

miRNA* sequence and Count(AGO1/wt):UGGUUUAGUCACUUUCACUGC_2.51/0.6

miRNA Position in PremiRNA:77-97

miRNA annonation:miRNA-1

2. Degradome supported cutsite(s) in Genome:19415175

PremiRNA Locus in Genome:>Chr2(19415069-19415180)_plus strand

PremiRNA sequence:GUAUUACAUGUUUUGUGCUUGAAUCUAAUUCAACAGGCUUUAUGUAAGAGAUUCUUUAACAAUUCCUAUAAUCUUUGUUGUUGGAUUAGAUUCACGCACAAACUCGUAAUCU

PremiRNA secondary structure:(.(((((..((((.((((.(((((((((((((((((.((.....)).(((((((................)))))))..))))))))))))))))).))))))))..))))).)

Degradome supported cutsite(s) in premiRNA:107

miRNA sequence and Count(AGO1/wt):UUAGAUUCACGCACAAACUCGU_2233.67/382.29

miRNA Position in PremiRNA:86-107

miRNA* sequence and Count(AGO1/wt):AUGUUUUGUGCUUGAAUCUAAUU_0/0

miRNA Position in PremiRNA:8-30

miRNA annonation:miRNA-2

3. Degradome supported cutsite(s) in Genome:15371933

PremiRNA Locus in Genome:>Chr3(15371931-15372073)_plus strand

PremiRNA sequence:UCAAAUGAGUUGAUGGGUCAAAUGAGUUAUUGGGUCAAUUGGUUUGAUGAAUAAAAUGAGUUGGGUUGUAAUGAUUAAUGGUUUCAAUGGUUUACCCAAUUAACCCAUUAACCCAUAACUCAUUUAACCCUAAACUCAUUUGA

PremiRNA secondary structure:((((((((((...((((.(((((((((((.((((.(((.((((.............((((((((...(((.(((.........))).))).))))))))))).))).)))))))))))))))).))))..))))))))))

Degradome supported cutsite(s) in premiRNA:3

miRNA sequence and Count(AGO1/wt):AAAUGAGUUGAUGGGUCAAAUGAG_0/2.6
miRNA Position in PremiRNA:3-26
miRNA* sequence and Count(AGO1/wt):UUUAACCCUAAACUCAUUUGAUU_0.72/0.4
miRNA Position in PremiRNA:121-143
miRNA annonation:miRNA-3

4. Degradome supported cutsite(s) in Genome:12138948
PremiRNA Locus in Genome:>Chr5(12138921-12138980)_plus strand
PremiRNA sequence:GGAGUCUUCUUUCUUCCUUGUGCUCGAGUGUCUGGUUGAGUGCGGAAUGAAGAAGGCUCU
PremiRNA secondary structure:((((((((((((..((((..(..(((((.(....).)))))..))))).))))))))))))
Degradome supported cutsite(s) in premiRNA:28
miRNA sequence and Count(AGO1/wt):UUGAGUGCGGAAUGAAGAAGGC_1.44/0
miRNA Position in PremiRNA:37-58
miRNA* sequence and Count(AGO1/wt):UCUUUUCUUCCUUGUGCUCGAG_0.36/0.2
miRNA Position in PremiRNA:6-30
miRNA annonation:miRNA-4

5. Degradome supported cutsite(s) in Genome:29911709
PremiRNA Locus in Genome:>Chr1(29911713-29911101)_minus strand
PremiRNA
sequence:UACUUCGGCGAUGAUGAUGAAACAAGAUUACCAUCAAACUUUGAGAGGUUUUUCGCUCGUUGAUGUAAACUUCUUUUUAUAAUCUGAGCCU
AGUUUGUUUGUUUGAAAUCUUAUGUGUUACAUGUUAUAUGAAAUCGUUUUGGUGAUAGGAAAGGCAACAAAUUAGAGUCUCUGAUUCACUUAUGUC
AAUAGUGGAUAAAGCGCUGUACGUUGCCGAGAUAGAUAUGGCGAUAUUCUGGUACUUGGACUAGUUUUGAUAGUUCCAUGUGCAUUCAAAAGCCCU
UACAUGUUUCUCUCAACUUAUUCCCAUUAGUAUAUGUAUUUUGAUUUGGUAAAGGCAACAAAUUAGAGUCUCUGAUUCACUUAUUCAAUAGUGGAU
AAAGCGCUGUACGUUGCCAAUAAUUACUUGGUCCAAAGAUCUCCCAAUCGGCUUUCAGUCUUAUUUUAGCCGAUAUGAUCGAUGUUCCUAAAUUAUA
GAUUUUGUUACGUGUUUUCAUAUAGCUGAGCUGACCAUAUCAUGCUAUUUACCAAAUCUUUUUGAUCAAUUACCUGACACAUUGACAGACGAUCGAU
UUUUAGACACUGAAAUAUGGGCCGAAAUUUUGAAGGAGUA
PremiRNA secondary
structure:((((((..(((.(((.((((((.(((.((((.(((((....((((.((....)).)))))))))))))).)))..)))))).))).(.(((((..(((.(((((((...((..((((((((...((((((((((.((.....((((((((..((((((.((((((((.((((((((...........((((((.......))))))..((
((((((((.(((((((((.........((((.(((((((.......))).)))).))))........))).....)))))).)))).........(((..((((....))))......)))....)))))).)))))))).))))))))..........((((((.......))))))..)))))).((((((.....((..((..((((.....(((((((............
..))))))..))))..))..))..))))))...)))))))))).)).)))))))))))..((((((.....))).)))......((((....))))..........))))))).))))))))).))).((((((...))))))..))))))......)))..))))))
Degradome supported cutsite(s) in premiRNA:6
miRNA sequence and Count(AGO1/wt):CGGCGAUGAUGAUGAAACAAGAU_0.36/0.2
miRNA Position in PremiRNA:6-28

miRNA* sequence and Count(AGO1/wt):CUUCUUUUUAUAAUCUGAGCCUA_0/0.2
miRNA Position in PremiRNA:70-610
miRNA annonation:miRNA-5

6. Degradome supported cutsite(s) in Genome:23988505
PremiRNA Locus in Genome:>Chr5(23988591-23988477)_minus strand
PremiRNA
sequence:UGCUGAUGCAGCAUCAUCAAGAUUCCCACUCGAUAAGGAACCCUUUUCUGCGGAUCGAGAGGCGAAAGCAAAGGGAACCACAUCUCUUGUG
AAUCUUAAUGGUGCUGCAUCUGCG
PremiRNA secondary structure:(((.(((((((((((((.(((((((((.((((((.(((((....)))))....))))))..((....))...))))..((((.....)))).))))).))))))))))))).)))
Degradome supported cutsite(s) in premiRNA:88
miRNA sequence and Count(AGO1/wt):UGAAUCUUAAUGGUGCUGCAU_0.36/0.2
miRNA Position in PremiRNA:90-110
miRNA* sequence and Count(AGO1/wt):UGCAGCAUCAUCAAGAUUCCC_0/0
miRNA Position in PremiRNA:8-82
miRNA annonation:miRNA-6

7. Degradome supported cutsite(s) in Genome:6220763/6220784
PremiRNA Locus in Genome:>Chr1(6220685-6220789)_plus strand
PremiRNA
sequence:UGAAGUAAAGCUGCUAAGCUAUGGAUCCCAUAAGCCUUAUCAAAUUCAAUAUAAUUGAUGAUAAGGUUUUUUUUAUGGAUGCCAUAUCUCA
GGAGCUUUCACUUA
PremiRNA secondary structure:(((.((((((((.((.((.(((((...((((((((((((((...(((((...))))))))))))))).....))))))...))))).)).)).))))))).)))))
Degradome supported cutsite(s) in premiRNA:79/100
miRNA sequence and Count(AGO1/wt):UGCCAUAUCUCAGGAGCUUUC_0.36/0
miRNA Position in PremiRNA:80-100
miRNA* sequence and Count(AGO1/wt):AAGCUGCUAAGCUAUGGAUCC_0/0.2
miRNA Position in PremiRNA:8-28
miRNA annonation:miRNA-7

8. Degradome supported cutsite(s) in Genome:8770553
PremiRNA Locus in Genome:>Chr5(8770544-8770622)_plus strand
PremiRNA sequence:UCAAAUUAAAUGAGUUAUGGGUUGACCCAACUCAUUUUGUUAAAUGGGUUGGGUCAACCCAUAACUCAUUUAACCCUAA

PremiRNA secondary structure:.....((((((((((((((((((((((((((((((((....)))))))))))))))))))))))))))))))).......
Degradome supported cutsite(s) in premiRNA:10
miRNA sequence and Count(AGO1/wt):UAAAUGAGUUAUGGGUUGACCC_1.44/0.2
miRNA Position in PremiRNA:2-28
miRNA* sequence and Count(AGO1/wt):UCAACCCAUAACUCAUUUAAC_0/0
miRNA Position in PremiRNA:54-74
miRNA annonation:miRNA-8

9. Degradome supported cutsite(s) in Genome:20772272
PremiRNA Locus in Genome:>Chr1(20772225-20772297)_plus strand
PremiRNA sequence:UGCUUCUUUGUCUACACUUUUGGAAAAGGUGAUGAUAUCAUUGCUUUUCCCCAAAUGUAGACAAAGCAAUACC

PremiRNA secondary structure:))...(((((((((((..(((((.(((((..(((....)))..)))))..)))))))))))))))).....))
Degradome supported cutsite(s) in premiRNA:48
miRNA sequence and Count(AGO1/wt):UCCCCAAAUGUAGACAAAGCA_84.62/20.75
miRNA Position in PremiRNA:48-68
miRNA* sequence and Count(AGO1/wt):CUUUGUCUACACUUUUGGAAA_0.25/0.4
miRNA Position in PremiRNA:6-26
miRNA annonation:miRNA-9

10. Degradome supported cutsite(s) in Genome:23988566
PremiRNA Locus in Genome:>Chr5(23988478-23988590)_plus strand
PremiRNA
sequence:GCAGAUGCAGCACCAUUAAGAUUCACAAGAGAUGUGGUUCCCUUUGCUUUCGCCUCUCGAUCCGCAGAAAAGGGUUCCUUAUCGAGUGGGA
AUCUUGAUGAUGCUGCAUCAGC
PremiRNA secondary structure:((.(((((((((.(((((((((((.((...((((.((..((((((((..(((.....)))...)))...)))))..)).))))...)).))))))))))).))))))))).))
Degradome supported cutsite(s) in premiRNA:89
miRNA sequence and Count(AGO1/wt):UGCAGCACCAUUAAGAUUCAC_1.08/0
miRNA Position in PremiRNA:6-26
miRNA* sequence and Count(AGO1/wt):GAAUCUUGAUGAUGCUGCAU_43.77/23.01
miRNA Position in PremiRNA:90-110
miRNA annonation:miRNA-12

11. Degradome supported cutsite(s) in Genome:16432049

PremiRNA Locus in Genome:>Chr1(16432023-16432076)_plus strand
PremiRNA sequence:CCCAUCAGAACUCCGCAGUUAAGCGCGCUUGGGCGAGAGUAGUACUAGAAUGGG
PremiRNA secondary structure:((((((((.(((((((...((((....)))).))).))))....)).).)))))
Degradome supported cutsite(s) in premiRNA:27
miRNA sequence and Count(AGO1/wt):CAGAACUCCGCAGUUAAGC_0/1.8
miRNA Position in PremiRNA:6-24
miRNA* sequence and Count(AGO1/wt):CUUGGGCGAGAGUAGUACUAG_4.66/3.2
miRNA Position in PremiRNA:29-50
miRNA annonation:miRNA-13

12. Degradome supported cutsite(s) in Genome:22165414
PremiRNA Locus in Genome:>Chr1(22165407-22165553)_plus strand
PremiRNA
sequence:AGAGUUUUAAAAGAUUUACAAGGGAUUUUAAAAAGUUUUAAAAAGAUUUACAAGAGUUUUUAAAGGGUCUUCUUGUAAAUUCUUUAAGAA
ACUCUUGUACAUCUUUUAAAACCCUUUUAAAAUCUCUUGUAAAUCUUUUAAAACCCU
PremiRNA secondary structure:((.((((((((((((((((((((((((((((((.(((((((((.(((.((((((((((((((((((((..........))))))).))))))))))))).))))))))))))..)))))))))))))))))))))))))))))).))

Degradome supported cutsite(s) in premiRNA:8
miRNA sequence and Count(AGO1/wt):UUUUAAAAGAUUUACAAGGGAUUU_3.59/1
miRNA Position in PremiRNA:5-28
miRNA* sequence and Count(AGO1/wt):AUCUCUUGUAAAUCUUUUAAAAC_0/0.2
miRNA Position in PremiRNA:122-145
miRNA annonation:miRNA-14

13. Degradome supported cutsite(s) in Genome:16102366
PremiRNA Locus in Genome:>Chr3(16102362-16102418)_plus strand
PremiRNA sequence:AUGAUUUACAAGGGAUUUUAAAAGUGUCUACAUGGUUUACAAUCGUUUUUAAAGCAU
PremiRNA secondary structure:(((.((((.(((.((((.((((.(((....)))..)))).)))).))).)))).)))
Degradome supported cutsite(s) in premiRNA:5
miRNA sequence and Count(AGO1/wt):UUUACAAUCGUUUUUAAAGCA_0.72/0
miRNA Position in PremiRNA:36-56
miRNA* sequence and Count(AGO1/wt):UUUACAAGGGAUUUUAAAA_0/0
miRNA Position in PremiRNA:4-24
miRNA annonation:miRNA-15

14. Degradome supported cutsite(s) in Genome:16159377
PremiRNA Locus in Genome:>Chr3(16159338-16159398)_plus strand
PremiRNA sequence:AGCGGUUGAGUUCCCGAAGUUGUCUCUUGUAGUUGCUGAUGACUUUUAGAACACGAUAGCU
PremiRNA secondary structure:(((.((((.((((..(((((..((....((....)).))..)))))..)))).)))).)))
Degradome supported cutsite(s) in premiRNA:40
miRNA sequence and Count(AGO1/wt):UGAUGACUUUUAGAACACGAU_6.82/1
miRNA Position in PremiRNA:37-57
miRNA* sequence and Count(AGO1/wt):UGAGUUCCCGAAGUUGUCUCU_1.08/0.4
miRNA Position in PremiRNA:7-27
miRNA annonation:miRNA-16

15. Degradome supported cutsite(s) in Genome:8858956
PremiRNA Locus in Genome:>Chr4(8858895-8858978)_plus strand
PremiRNA sequence:CAACUUUAUUAGUCAAGUUUUAAAUUGUGGCCAUUUUAAACAUCAAGUUCAUAUGUGGCCAUUUAAAACAUGAACUAAACGUUG

PremiRNA secondary structure:((((....(((((((.(((((((...((((((((....(((.....))).....))))))))))))))).)).)))))..))))
Degradome supported cutsite(s) in premiRNA:62
miRNA sequence and Count(AGO1/wt):UUUAAAACAUGAACUAAACGU_7.89/1.4
miRNA Position in PremiRNA:62-82
miRNA* sequence and Count(AGO1/wt):UUUAUUAGUCAAGUUUUAAAUUGU_0/0.2
miRNA Position in PremiRNA:5-29
miRNA annonation:miRNA-17

16. Degradome supported cutsite(s) in Genome:8770553
PremiRNA Locus in Genome:>Chr5(8770549-8770616)_plus strand
PremiRNA sequence:UUAAAUGAGUUAUGGGUUGACCCAACUCAUUUUGUUAAAUGGGUUGGGUCAACCCAUAACUCAUUUAA
PremiRNA secondary structure:((((((((((((((((((((((((((((((((....))))))))))))))))))))))))))))))))
Degradome supported cutsite(s) in premiRNA:5
miRNA sequence and Count(AGO1/wt):UAAAUGAGUUAUGGGUUGACCC_1.44/0.2
miRNA Position in PremiRNA:2-23
miRNA* sequence and Count(AGO1/wt):UCAACCCAUAACUCAUUUAAC_0/0
miRNA Position in PremiRNA:48-69
miRNA annonation:miRNA-19

17. Degradome supported cutsite(s) in Genome:16661145
PremiRNA Locus in Genome:>Chr5(16661213-16661123)_minus strand
PremiRNA
sequence:UAAUUGACUCUCGACUCAUUCAUCCAAAUACCAAAUGAAAGAAUUUGUUCUCAUAUGGUAAAUGAAUGAAUGAUGCGAGAGACAAAUUGA

PremiRNA secondary structure:...(((.((((((..((((((((.((..(((((.((((..(((....))))))).)))))..)).))))))))..)))))).)))....)
Degradome supported cutsite(s) in premiRNA:69
miRNA sequence and Count(AGO1/wt):AAUGAAUGAUGCGAGAGACAA_40.56/20.95
miRNA Position in PremiRNA:69-86
miRNA* sequence and Count(AGO1/wt):GACUCUCGACUCAUUCAUCCA_1.25/7.45
miRNA Position in PremiRNA:6-22
miRNA annonation:miRNA-21

18. Degradome supported cutsite(s) in Genome:22322724
PremiRNA Locus in Genome:>Chr5(22322903-22322720)_minus strand
PremiRNA
sequence:AAGCCUUGAUGAUUCGACAAAGUGAAGGGUUUGGUCUUCAGAAAGUUUUCUUGCAAGGUUCAAGAACGGAUCCUCAAAAUUCAAACUGUUU
CUCACCAAUUUGAAUUGUGAAGAUCCGUUCUUGAACCUUGGAAGAAAACUUUCUGGAGACCAAACCCUUCACUUUGUCGAGUCACCAAGGCUU

PremiRNA secondary
structure:((((((((.((.((((((((((((((((((.(((.((((((((.((........)).)))))))).))).)))))))))))))))))).))).))))))))

Degradome supported cutsite(s) in premiRNA:181
miRNA sequence and Count(AGO1/wt):UUGAUGAUUCGACAAAGUGAA_329.71/118.23
miRNA Position in PremiRNA:6-26
miRNA* sequence and Count(AGO1/wt):CACUUUGUCGAGUCACCAAGG_0/1.8
miRNA Position in PremiRNA:161-181
miRNA annonation:miRNA-22

19. Degradome supported cutsite(s) in Genome:771406
PremiRNA Locus in Genome:>Chr2(771517-771382)_minus strand

PremiRNA
sequence:GAAGACACUGAAGGACCUAAACUAACAAAGGUAAACGGCUCAGUGUGCGGGGUAUUACACUCGGUUUAAUGUCUGAAUGCGAUAAUCCGCA
CGAUGAUCUCUUUAUCUUUGUUUGUUUAGGUCCCUUAGUUUCUUC
PremiRNA secondary structure:(((((.(((((.((((((((((.(((((((((((((.((.(((.(((((((..(((..((.((((........)))).)).)))..))))))).))).)).))))))))))))).)))))))))).))))).)))))
Degradome supported cutsite(s) in premiRNA:113
miRNA sequence and Count(AGO1/wt):CACUGAAGGACCUAAACUAAC_15.43/7.6
miRNA Position in PremiRNA:6-26
miRNA* sequence and Count(AGO1/wt):UUGUUUAGGUCCCUUAGUUUC_17.58/12.2
miRNA Position in PremiRNA:113-133
miRNA annonation:miRNA-23

20. Degradome supported cutsite(s) in Genome:16859141
PremiRNA Locus in Genome:>Chr1(16859099-16859159)_plus strand
PremiRNA sequence:AGCCAAUGGCAGCCAUUGUAAAUAAGUCCUAACUCCAGGAUUUAUUUCACAAAAUGGCUGC
PremiRNA secondary structure:........((((((((((((((((((((((......))))))))))).)))..))))))))
Degradome supported cutsite(s) in premiRNA:43
miRNA sequence and Count(AGO1/wt):AUGGCAGCCAUUGUAAAUAA_0/0
miRNA Position in PremiRNA:6-25
miRNA* sequence and Count(AGO1/wt):UUUAUUUCACAAAAUGGCUGCA_0.36/0.2
miRNA Position in PremiRNA:44-66
miRNA annonation:miRNA-25

21. Degradome supported cutsite(s) in Genome:8046230
PremiRNA Locus in Genome:>Chr2(8045548-8046257)_plus strand
PremiRNA
sequence:AUGAGGCUUGUUAAUGGAUUUAGAACAUUCCUUAUUUUGCUUCUAUAAAUAAAGAGAAGAAAAUAAUUACUAGCCAAAAUGUUUGCUCAA
UCUCUAACAAUUUUUUUUAUUGUUAAGUUCUUUCAAUGUAACAGAAUAAUAAUAAGUAGAUUAUAACCCGCGGUACACCGCAGAAAUAAUUGUUUU
UUUAAAAUUAGUAUAUAUAAAAGUCUGUAAAUUAUAUUUAUUUAUAAAAUAUUUAUAUUUUAUAGUUUACAAGUAACAUCCUUCCAAACCCGUUCU
ACCAAACCCGUCCCGUUAAAAAAAUCUGCAGUACACCGCUAAUUUAAAAAAAUAUUUUACGGGAUUGCAAUUAUAUUUUAGUUUGUCAAACAAAUU
UUUGUUUUAAAUAGUUGUCGAAAUCUAAUUCAAAUAUUGGAGAAAUAAGAUUUAGUGAACUGAUAAGAAAUGUAUUUAGGUGCGUUUUGCUCAUUU
UAAGGGAUUGCAAUUGUAUUUUGGUUUGUCAAAUAUUUUUUGUUUUAAAUAGUUUCCGAAAUCUAGUUCAAAUUUUGGAGAAAUUUGCGUGAUAUA
UGGGAUUAGAAUAUAUUUGAAAUAUUUUUUGUUUGUUAAUCAAUUUAUUGCCAAAAGAAAAUCAAUAAUUAAAAGCUAGUGGCAGCCAUUGUAAAU
AAGUUACAACUCCAGGAUUUAUUUCACAAAAUGGCUGCAAAAAU

PremiRNA secondary structure:((..(((((.(((((..((((((((((....(((((((.(((((..........))))))))))))..((.((((((((((.((((....((((((((.........(((((((((..((((.......).)))..))))))))....((((((((((..((((....)))).(((((((..((((.(((.(((((((...((((((.....(((((((((((......(((((.....)))))...)))))))))))...............................(((((((.((((((((..((........))..)))).........)))))))))))).....)))))).)))))))...))).))))..)))))))).....)))))...))))))........))))))).....(((((..((((.(((...(((((((((....)))))))))..))).))))..)))))))))...))))))))))).)).........))))))))))..((((((((((.........))))))))))..((((.(..(((.((..((((((...(((.............)))...)))))).)).)))..)))))...........))))))))))....(((((((((((((((((((..... .....))))))))).)))..))))))))....))

Degradome supported cutsite(s) in premiRNA:683

miRNA sequence and Count(AGO1/wt):UUUAUUUCACAAAAUGGCUGCA_0.36/0.2

miRNA Position in PremiRNA:684-705

miRNA* sequence and Count(AGO1/wt):CAGCCAUUGUAAAUAAGUU_0/0

miRNA Position in PremiRNA:653-671

miRNA annonation:miRNA-26

22. Degradome supported cutsite(s) in Genome:9979134

PremiRNA Locus in Genome:>Chr5(9979099-9979161)_plus strand

PremiRNA sequence:AGUGACAGCCAUUGUAAAUAAGUUCCAACUCCAAGAUUUAUUUCACAAAAUGGCUGCAAAAAU

PremiRNA secondary structure:(.((.((((((((((((((((((..........))))))))).)))..)))))))))....)

Degradome supported cutsite(s) in premiRNA:36

miRNA sequence and Count(AGO1/wt):UUUAUUUCACAAAAUGGCUGCA_0.36/0.2

miRNA Position in PremiRNA:37-58

miRNA* sequence and Count(AGO1/wt):CAGCCAUUGUAAAUAAGUU_0/0

miRNA Position in PremiRNA:6-24

miRNA annonation:miRNA-27

23. Degradome supported cutsite(s) in Genome:8277186

PremiRNA Locus in Genome:>Chr1(8277133-8277214)_plus strand

PremiRNA sequence:GUUUCAUCGCUUGUAUAUUUAAGAAAUUUGGAAAAAUAUUGAUUUUAUUAAAUUGGUUUAGAUGUGUUGGUUAAACUUAAAU

PremiRNA secondary structure:((((....(((..((((((((((.(((((((.(((((....))))).)))))))..))))))))))..))).))))......

Degradome supported cutsite(s) in premiRNA:54

miRNA sequence and Count(AGO1/wt):UUUAGAUGUGUUGGUUAAACU_3.23/0

miRNA Position in PremiRNA:57-77

miRNA* sequence and Count(AGO1/wt):UUUCAUCGCUUGUAUAUUUAAGAA_0.72/0

miRNA Position in PremiRNA:2-25

miRNA annonation:miRNA-28

24. Degradome supported cutsite(s) in Genome:29612044
PremiRNA Locus in Genome:>Chr1(29612040-29612098)_plus strand
PremiRNA sequence:CUAAUUUUACGGUUUUGGCGGGAAAACGCAAUUCCAUAAUUUUUCCGCCAAAAUCGUGA
PremiRNA secondary structure:......((((((((((((((((((((............))))))))))))))))))))
Degradome supported cutsite(s) in premiRNA:5
miRNA sequence and Count(AGO1/wt):UUUACGGUUUUGGCGGGAAAA_1.08/0
miRNA Position in PremiRNA:6-26
miRNA* sequence and Count(AGO1/wt):UUUUUCCGCCAAAAUCGUGAUUU_0.36/0
miRNA Position in PremiRNA:42-62
miRNA annonation:miRNA-29

25. Degradome supported cutsite(s) in Genome:19659579
PremiRNA Locus in Genome:>Chr3(19659673-19659574)_minus strand
PremiRNA
sequence:ACUGUCGUCUCGGUUCGCGAUCCACAAGUAAUCUUUUGUGGAGAUUAUGAAAACAAUCAUCACGAGAGGCUACGUGUGGGUGGCAAACAAA
GACGACAAU
PremiRNA secondary structure:(.((((((((..(((.((.(((((((.(((..((((((((..((((.(....).))))..))))))))..))).))))))).)).)))..)))))))).)
Degradome supported cutsite(s) in premiRNA:96
miRNA sequence and Count(AGO1/wt):UGUGGGUGGCAAACAAAGACG_0/0
miRNA Position in PremiRNA:75-95
miRNA* sequence and Count(AGO1/wt):UCUCGGUUCGCGAUCCACAAG_4.66/1
miRNA Position in PremiRNA:8-28
miRNA annonation:miRNA-30

Supplementary table S3c Discovery of novel miRNAs in roots of *Arabidopsis*

1. Degradome supported cutsite(s) in Genome:17825730/17825821
PremiRNA Locus in Genome:>Chr1(17825725-17825824)_plus strand
PremiRNA
sequence:AUGCAUUGAAAGUGACUACAUCGGGGUUCCGAUUUUUUUUGUUCUUCAUAUGAUGAAGCGGAAACAGUAAUCAACCCUGGUUUAGUCACUU
UCACUGCAU
PremiRNA secondary structure:(((((.((((((((((((.(((((((((..((((....(((((((((((...))))))....))))).)))))))))))).))))))))))).)))))
Degradome supported cutsite(s) in premiRNA:6/97
miRNA sequence and Count(AGO1/wt):UUGAAAGUGACUACAUCGGGG_4957.93/843.29
miRNA Position in PremiRNA:6-26
miRNA* sequence and Count(AGO1/wt):UGGUUUAGUCACUUUCACUGC_0.79/1.44
miRNA Position in PremiRNA:77-97
miRNA annonation:miRNA-1

2. Degradome supported cutsite(s) in Genome:19415175
PremiRNA Locus in Genome:>Chr2(19415069-19415180)_plus strand
PremiRNA
sequence:GUAUUACAUGUUUUGUGCUUGAAUCUAAUUCAACAGGCUUUAUGUAAGAGAUUCUUUAACAAUUCCUAUAAUCUUUGUUGUUGGAUUAGA
UUCACGCACAAACUCGUAAUCU
PremiRNA secondary structure:(.(((((..((((.((((.((((((((((((((((.((.....)).(((((((................)))))))..)))))))))))))))).))))))))..))))).)
Degradome supported cutsite(s) in premiRNA:107
miRNA sequence and Count(AGO1/wt):UUAGAUUCACGCACAAACUCGU_675.99/299.33
miRNA Position in PremiRNA:86-107
miRNA* sequence and Count(AGO1/wt):AUGUUUUGUGCUUGAAUCUAAUU_0/0
miRNA Position in PremiRNA:8-30
miRNA annonation:miRNA-2

3. Degradome supported cutsite(s) in Genome:15371933
PremiRNA Locus in Genome:>Chr3(15371931-15372073)_plus strand
PremiRNA
sequence:UCAAAUGAGUUGAUGGGUCAAAUGAGUUAUUGGGUCAAUUGGUUUGAUGAAUAAAAUGAGUUGGGUUGUAAUGAUUAAUGGUUUCAAUGG
UUUACCCAAUUAACCCAUUAACCCAUAACUCAUUUAACCCUAAACUCAUUUGA
PremiRNA secondary structure:((((((((((...((((.(((((((((((.((((.(((.((((.............((((((((...(((.(((.........))).))).)))))))))))).))).))))))))))))))).)))))..))))))))))
Degradome supported cutsite(s) in premiRNA:3

miRNA sequence and Count(AGO1/wt):AAAUGAGUUGAUGGGUCAAAUGAG_1.97/2.88
miRNA Position in PremiRNA:3-26
miRNA* sequence and Count(AGO1/wt):UUUAACCCUAAACUCAUUUGAUU_0.39/1.73
miRNA Position in PremiRNA:121-143
miRNA annonation:miRNA-3

4. Degradome supported cutsite(s) in Genome:29911709
PremiRNA Locus in Genome:>Chr1(29911713-29911101)_minus strand
PremiRNA
sequence:UACUUCGGCGAUGAUGAUGAAACAAGAUUACCAUCAAACUUUGAGAGGUUUUUCGCUCGUUGAUGUAAACUUCUUUUUAUAAUCUGAGCCU
AGUUUGUUUGUUUGAAAUCUUAUGUGUUACAUGUUAUAUGAAAUCGUUUUGGUGAUAGGAAAGGCAACAAAUUAGAGUCUCUGAUUCACUUAUGUC
AAUAGUGGAUAAAGCGCUGUACGUUGCCGAGAUAGAUAUGGCGAUAUUCUGGUACUUGGACUAGUUUUGAUAGUUCCAUGUGCAUUCAAAAGCCCU
UACAUGUUUCUCUCAACUUAUUCCCAUUAGUAUAUGUAUUUUGAUUUGGUAAAGGCAACAAAUUAGAGUCUCUGAUUCACUUAUUCAAUAGUGGAU
AAAGCGCUGUACGUUGCCAAUAAUUACUUGGUCCAAAGAUCUCCCAAUCGGCUUUCAGUCUUAUUUUAGCCGAUAUGAUCGAUGUUCCUAAAUUAUA
GAUUUUGUUACGUGUUUUCAUAUAGCUGAGCUGACCAUAUCAUGCUAUUUACCAAAUCUUUUUGAUCAAUUACCUGACACAUUGACAGACGAUCGAU
UUUUAGACACUGAAAUAUGGGCCGAAAUUUUGAAGGAGUA
PremiRNA secondary
structure:((((((..(((.(((.((((((.(((.((((.(((((....((((.((....)).))))))))))))).))).)))))).))).(.(((((..(((.(((((((...((..((((((((...(((((((((((.((.....(((((((((..((((((.((((((((.((((((((..........((((((.......))))))..((
((((((((.(((((((((.........((((.(((((((.......))).))))).))))........)))).....)))))).))))).........(((..((((....))))......)))....)))))).)))))))).))))))))..........((((((.......))))))..)))))).((((((....((..((..((((.....(((((((............
..)))))))..)))).)).)).)))))...))))))))).)).)))))))))))..((((((.....))).)))......((((....))))..........)))))))).)))))))).))).((((((...))))))..))))))......)))..))))))
Degradome supported cutsite(s) in premiRNA:6
miRNA sequence and Count(AGO1/wt):CGGCGAUGAUGAUGAAACAAGAU_230.7/0
miRNA Position in PremiRNA:6-28
miRNA* sequence and Count(AGO1/wt):CUUCUUUUUAUAAUCUGAGCCUA_0.79/0
miRNA Position in PremiRNA:70-610
miRNA annonation:miRNA-5

5. Degradome supported cutsite(s) in Genome:23988505
PremiRNA Locus in Genome:>Chr5(23988591-23988477)_minus strand
PremiRNA
sequence:UGCUGAUGCAGCAUCAUCAAGAUUCCCACUCGAUAAGGAACCCUUUUCUGCGGAUCGAGAGGCGAAAGCAAAGGGAACCACAUCUCUUGUG
AAUCUUAAUGGUGCUGCAUCUGCG
PremiRNA secondary structure:(((.(((((((((((((.(((((((((.((((((.(((((....)))))....))))))..((....))...)))).((((.....)))).))))).))))))))))))).)))
Degradome supported cutsite(s) in premiRNA:88

miRNA sequence and Count(AGO1/wt):UGAAUCUUAAUGGUGCUGCAU_1.18/1.44
miRNA Position in PremiRNA:90-110
miRNA* sequence and Count(AGO1/wt):UGCAGCAUCAUCAAGAUUCCC_0.39/0
miRNA Position in PremiRNA:8-82
miRNA annonation:miRNA-6

6. Degradome supported cutsite(s) in Genome:8770553
PremiRNA Locus in Genome:>Chr5(8770544-8770622)_plus strand
PremiRNA sequence:UCAAAUUAAAUGAGUUAUGGGUUGACCCAACUCAUUUUGUUAAAUGGGUUGGGUCAACCCAUAACUCAUUUAACCCUAA

PremiRNA secondary structure:.....((((((((((((((((((((((((((((((((....))))))))))))))))))))))))))))))))......
Degradome supported cutsite(s) in premiRNA:10
miRNA sequence and Count(AGO1/wt):UAAAUGAGUUAUGGGUUGACCC_0.39/0
miRNA Position in PremiRNA:2-28
miRNA* sequence and Count(AGO1/wt):UCAACCCAUAACUCAUUUAAC_0.39/0
miRNA Position in PremiRNA:54-74
miRNA annonation:miRNA-8

7. Degradome supported cutsite(s) in Genome:20772272
PremiRNA Locus in Genome:>Chr1(20772225-20772297)_plus strand
PremiRNA sequence:UGCUUCUUUGUCUACACUUUUGGAAAAGGUGAUGAUAUCAUUGCUUUUCCCCAAAUGUAGACAAAGCAAUACC

PremiRNA secondary structure:))...(((((((((((..(((((.(((((..(((....)))..)))))..))))))))))))))))).....))
Degradome supported cutsite(s) in premiRNA:48
miRNA sequence and Count(AGO1/wt):UCCCCAAAUGUAGACAAAGCA_84.62/20.75
miRNA Position in PremiRNA:48-68
miRNA* sequence and Count(AGO1/wt):CUUUGUCUACACUUUUGGAAA_0.25/0.4
miRNA Position in PremiRNA:6-26
miRNA annonation:miRNA-9

8. Degradome supported cutsite(s) in Genome:687897
PremiRNA Locus in Genome:>Chr4(687895-687964)_plus strand
PremiRNA sequence:UGAAUAGCCUCCGAAUAACGGGAAUGUGAAAUAUAGGAACACCUUCUUGUUCAGACAGAGGCUUAUAUCA

PremiRNA secondary structure:(((..((((((.(..(((((((((.(((...........))).)))))))).)..).))))))....)))
Degradome supported cutsite(s) in premiRNA:3
miRNA sequence and Count(AGO1/wt):AUAGCCUCCGAAUAACGGGAA_10.22/0
miRNA Position in PremiRNA:4-24
miRNA* sequence and Count(AGO1/wt):CUUGUUCAGACAGAGGCUUAUA_0.39/0
miRNA Position in PremiRNA:46-67
miRNA annonation:miRNA-11

9. Degradome supported cutsite(s) in Genome:23988566
PremiRNA Locus in Genome:>Chr5(23988478-23988590)_plus strand
PremiRNA
sequence:GCAGAUGCAGCACCAUUAAGAUUCACAAGAGAUGUGGUUCCCUUUGCUUUCGCCUCUCGAUCCGCAGAAAAGGGUUCCUUAUCGAGUGGGA
AUCUUGAUGAUGCUGCAUCAGC
PremiRNA secondary structure:((.(((((((((.(((((((((((.((...((((.((..((((((((..(((.....)))...)))...)))))..)).))))...)).)))))))))))).))))))))).))
Degradome supported cutsite(s) in premiRNA:89
miRNA sequence and Count(AGO1/wt):UGCAGCACCAUUAAGAUUCAC_3.93/1.15
miRNA Position in PremiRNA:6-26
miRNA* sequence and Count(AGO1/wt):GAAUCUUGAUGAUGCUGCAU_6.68/15.83
miRNA Position in PremiRNA:90-110
miRNA annonation:miRNA-12

10. Degradome supported cutsite(s) in Genome:16432049
PremiRNA Locus in Genome:>Chr1(16432023-16432076)_plus strand
PremiRNA sequence:CCCAUCAGAACUCCGCAGUUAAGCGCGCUUGGGCGAGAGUAGUACUAGAAUGGG
PremiRNA secondary structure:((((((((.((((((((...((((....)))).))).))))....)).).)))))
Degradome supported cutsite(s) in premiRNA:27
miRNA sequence and Count(AGO1/wt):CAGAACUCCGCAGUUAAGC_0/0.29
miRNA Position in PremiRNA:6-24
miRNA* sequence and Count(AGO1/wt):CUUGGGCGAGAGUAGUACUAG_1.97/2.88
miRNA Position in PremiRNA:29-50
miRNA annonation:miRNA-13

11. Degradome supported cutsite(s) in Genome:22165414
PremiRNA Locus in Genome:>Chr1(22165407-22165553)_plus strand

PremiRNA
sequence:AGAGUUUUAAAAGAUUUACAAGGGAUUUUAAAAAGUUUUAAAAAGAUUUACAAGAGUUUUUAAAGGGUCUUCUUGUAAAUUCUUUAAGAA
ACUCUUGUACAUCUUUUAAAACCCUUUUAAAAUCUCUUGUAAAUCUUUUAAAACCCU
PremiRNA secondary structure:((.(((((((((((((((((((((((((((((((.(((((((((.(((.(((((((((((((((((((..........))))))).))))))))))))).))))))))))))..))))))))))))))))))))))))))))))).))

Degradome supported cutsite(s) in premiRNA:8
miRNA sequence and Count(AGO1/wt):UUUUAAAAGAUUUACAAGGGAUUU_1.57/2.3
miRNA Position in PremiRNA:5-28
miRNA* sequence and Count(AGO1/wt):AUCUCUUGUAAAUCUUUUAAAAC_0.39/0
miRNA Position in PremiRNA:122-145
miRNA annonation:miRNA-14

12. Degradome supported cutsite(s) in Genome:16159377
PremiRNA Locus in Genome:>Chr3(16159338-16159398)_plus strand
PremiRNA sequence:AGCGGUUGAGUUCCCGAAGUUGUCUCUUGUAGUUGCUGAUGACUUUUAGAACACGAUAGCU
PremiRNA secondary structure:(((.((((.((((..(((((..((....((....)).))..)))))..)))).)))).)))
Degradome supported cutsite(s) in premiRNA:40
miRNA sequence and Count(AGO1/wt):UGAUGACUUUUAGAACACGAU_2.36/0.29
miRNA Position in PremiRNA:37-57
miRNA* sequence and Count(AGO1/wt):UGAGUUCCCGAAGUUGUCUCU_0/0
miRNA Position in PremiRNA:7-27
miRNA annonation:miRNA-16

13. Degradome supported cutsite(s) in Genome:8858956
PremiRNA Locus in Genome:>Chr4(8858895-8858978)_plus strand
PremiRNA sequence:CAACUUUAUUAGUCAAGUUUUAAAUUGUGGCCAUUUUAAACAUCAAGUUCAUAUGUGGCCAUUUAAAACAUGAACUAAACGUUG

PremiRNA secondary structure:((((....(((((((.(((((((...((((((((....(((.....))).....)))))))))))))).)).))))))..))))
Degradome supported cutsite(s) in premiRNA:62
miRNA sequence and Count(AGO1/wt):UUUAAAACAUGAACUAAACGU_2.75/1.73
miRNA Position in PremiRNA:62-82
miRNA* sequence and Count(AGO1/wt):UUUAUUAGUCAAGUUUUAAAUUGU_0/0.58
miRNA Position in PremiRNA:5-29
miRNA annonation:miRNA-17

14. Degradome supported cutsite(s) in Genome:8770553
PremiRNA Locus in Genome:>Chr5(8770549-8770616)_plus strand
PremiRNA sequence:UUAAAUGAGUUAUGGGUUGACCCAACUCAUUUUGUUAAAUGGGUUGGGUCAACCCAUAACUCAUUUAA
PremiRNA secondary structure:((((((((((((((((((((((((((((((((....))))))))))))))))))))))))))))))))
Degradome supported cutsite(s) in premiRNA:5
miRNA sequence and Count(AGO1/wt):UAAAUGAGUUAUGGGUUGACCC_0.39/0
miRNA Position in PremiRNA:2-23
miRNA* sequence and Count(AGO1/wt):UCAACCCAUAACUCAUUUAAC_0.39/0
miRNA Position in PremiRNA:48-69
miRNA annonation:miRNA-19

15. Degradome supported cutsite(s) in Genome:16661145
PremiRNA Locus in Genome:>Chr5(16661213-16661123)_minus strand
PremiRNA
sequence:UAAUUGACUCUCGACUCAUUCAUCCAAAUACCAAAUGAAAGAAUUUGUUCUCAUAUGGUAAAUGAAUGAAUGAUGCGAGAGACAAAUUGA

PremiRNA secondary structure:...(((.((((((..((((((((.((..(((((.((((..(((....))))))).)))))..)).))))))))..)))))).)))....)
Degradome supported cutsite(s) in premiRNA:69
miRNA sequence and Count(AGO1/wt):AAUGAAUGAUGCGAGAGACAA_40.56/20.95
miRNA Position in PremiRNA:69-86
miRNA* sequence and Count(AGO1/wt):GACUCUCGACUCAUUCAUCCA_1.25/7.45
miRNA Position in PremiRNA:6-22
miRNA annonation:miRNA-21

16. Degradome supported cutsite(s) in Genome:22322724
PremiRNA Locus in Genome:>Chr5(22322903-22322720)_minus strand
PremiRNA
sequence:AAGCCUUGAUGAUUCGACAAAGUGAAGGGUUUGGUCUUCAGAAAGUUUUCUUGCAAGGUUCAAGAACGGAUCCUCAAAAUUCAAACUGUUU
CUCACCAAUUUGAAUUGUGAAGAUCCGUUCUUGAACCUUGGAAGAAAACUUUCUGGAGACCAAACCCUUCACUUUGUCGAGUCACCAAGGCUU

PremiRNA secondary
structure:(((((((.(((.(((((((((((((((((.(((.(((((((.((........)).))))))).))).))))))))))))))))).))).)))))))

Degradome supported cutsite(s) in premiRNA:181
miRNA sequence and Count(AGO1/wt):UUGAUGAUUCGACAAAGUGAA_233.45/79.15
miRNA Position in PremiRNA:6-26
miRNA* sequence and Count(AGO1/wt):CACUUUGUCGAGUCACCAAGG_0/0.86
miRNA Position in PremiRNA:161-181
miRNA annonation:miRNA-22

17. Degradome supported cutsite(s) in Genome:771406
PremiRNA Locus in Genome:>Chr2(771517-771382)_minus strand
PremiRNA
sequence:GAAGACACUGAAGGACCUAAACUAACAAAGGUAAACGGCUCAGUGUGCGGGGUAUUACACUCGGUUUAAUGUCUGAAUGCGAUAAUCCGCA
CGAUGAUCUCUUUAUCUUUGUUUGUUUAGGUCCCUUAGUUUCUUC
PremiRNA secondary structure:(((((.(((((.((((((((((.((((((((((((.((.(((.(((((((..(((..((.((((........)))).))..)))..))))))).))).)).)))))))))))).)))))))))).))))).)))))
Degradome supported cutsite(s) in premiRNA:113
miRNA sequence and Count(AGO1/wt):CACUGAAGGACCUAAACUAAC_1.97/2.59
miRNA Position in PremiRNA:6-26
miRNA* sequence and Count(AGO1/wt):UUGUUUAGGUCCCUUAGUUUC_20.44/14.97
miRNA Position in PremiRNA:113-133
miRNA annonation:miRNA-23

18. Degradome supported cutsite(s) in Genome:25784115
PremiRNA Locus in Genome:>Chr5(25784115-25784187)_plus strand
PremiRNA sequence:GGUUUCGUGGUGUAGUUGGUUAUCACGUCAGUCUAACACACUGAAGGUCUCCGGUUCGAACCCGGGCGAAGCC

PremiRNA secondary structure:(((((((((((..((..((..(((...(((((.......))))).)))..))..))...)))...))))))))
Degradome supported cutsite(s) in premiRNA:1
miRNA sequence and Count(AGO1/wt):GGUUUCGUGGUGUAGUUGGU_14.15/2.01
miRNA Position in PremiRNA:1-20
miRNA* sequence and Count(AGO1/wt):UCCGGUUCGAACCCGGGCGAAGCC_0/0
miRNA Position in PremiRNA:52-75
miRNA annonation:miRNA-24

19. Degradome supported cutsite(s) in Genome:8046230
PremiRNA Locus in Genome:>Chr2(8045548-8046257)_plus strand

PremiRNA
sequence:AUGAGGCUUGUUAAUGGAUUUAGAACAUUCCUUAUUUUGCUUCUAUAAAUAAAGAGAAGAAAAUAAUUACUAGCCAAAAUGUUUGCUCAA
UCUCUAACAAUUUUUUUUAUUGUUAAGUUCUUUCAAUGUAACAGAAUAAUAAUAAGUAGAUUAUAACCCGCGGUACACCGCAGAAAUAAUUGUUUU
UUUAAAAUUAGUAUAUAUAAAAGUCUGUAAAUUAUAUUUAUUUAUAAAAUAUUUAUAUUUUAUAGUUUACAAGUAACAUCCUUCCAAACCCGUUCU
ACCAAACCCGUCCCGUUAAAAAAAUCUGCAGUACACCGCUAAUUUAAAAAAAUAUUUUACGGGAUUGCAAUUAUAUUUUAGUUUGUCAAACAAAUU
UUUGUUUUAAAUAGUUGUCGAAAUCUAAUUCAAAUAUUGGAGAAAUAAGAUUUAGUGAACUGAUAAGAAAUGUAUUUAGGUGCGUUUUGCUCAUUU
UAAGGGAUUGCAAUUGUAUUUUGGUUUGUCAAAUAUUUUUUGUUUUAAAUAGUUUCCGAAAUCUAGUUCAAAUUUUGGAGAAAUUUGCGUGAUAUA
UGGGAUUAGAAUAUAUUUGAAAUAUUUUUUGUUUGUUAAUCAAUUUAUUGCCAAAAGAAAAUCAAUAAUUAAAAGCUAGUGGCAGCCAUUGUAAAU
AAGUUACAACUCCAGGAUUUAUUUCACAAAAUGGCUGCAAAAAU
PremiRNA secondary
structure:((..(((((.(((((..((((((((((....(((((((.(((((..........))))))))))))..((.(((((((((.((((....(((((((.........((((((((..((((.......).)))..))))))))....((((((((((..((((....)))).(((((((..((((.(((.(((((((...((((((.....((
(((((((((......(((((.....)))))...)))))))))))...............................(((((((.((((((((..((.........))..)))).........)))))))))))).....)))))).))))))))...))).)))))..))))))).....)))))...))))))).........)))))))).....(((((..((((.(((...(((
((((((....)))))))))..))).)))))..)))))))))...))))))))).))...........))))))))))..((((((((((.........))))))))))..((((.(..(((.((..((((((...(((..............)))...)))))).)).)))..)))))...........))))))))))....(((((((((((((((((((.....
.....))))))))).)))..))))))))....))
Degradome supported cutsite(s) in premiRNA:683
miRNA sequence and Count(AGO1/wt):UUUAUUUCACAAAAUGGCUGCA_0/0.29
miRNA Position in PremiRNA:684-705
miRNA* sequence and Count(AGO1/wt):CAGCCAUUGUAAAUAAGUU_0.39/0
miRNA Position in PremiRNA:653-671
miRNA annonation:miRNA-26

20. Degradome supported cutsite(s) in Genome:9979134
PremiRNA Locus in Genome:>Chr5(9979099-9979161)_plus strand
PremiRNA sequence:AGUGACAGCCAUUGUAAAUAAGUUCCAACUCCAAGAUUUAUUUCACAAAAUGGCUGCAAAAAU
PremiRNA secondary structure:(.((.(((((((((((((((((((..........))))))))).)))..)))))))))....)
Degradome supported cutsite(s) in premiRNA:36
miRNA sequence and Count(AGO1/wt):UUUAUUUCACAAAAUGGCUGCA_0/0.29
miRNA Position in PremiRNA:37-58
miRNA* sequence and Count(AGO1/wt):CAGCCAUUGUAAAUAAGUU_0.39/0
miRNA Position in PremiRNA:6-24
miRNA annonation:miRNA-27

21. Degradome supported cutsite(s) in Genome:19659579
PremiRNA Locus in Genome:>Chr3(19659673-19659574)_minus strand

PremiRNA sequence:ACUGUCGUCUCGGUUCGCGAUCCACAAGUAAUCUUUUGUGGAGAUUAUGAAAACAAUCAUCACGAGAGGCUACGUGUGGGUGGCAAACAAAGACGACAAU

PremiRNA secondary structure:(.((((((((..(((.((.(((((((.(((..((((((((..((((.(....).))))..))))))))..))).))))))).)).)))..)))))))).)

Degradome supported cutsite(s) in premiRNA:96

miRNA sequence and Count(AGO1/wt):UGUGGGUGGCAAACAAAGACG_0/0

miRNA Position in PremiRNA:75-95

miRNA* sequence and Count(AGO1/wt):UCUCGGUUCGCGAUCCACAAG_1.18/0

miRNA Position in PremiRNA:8-28

miRNA annonation:miRNA-30

Supplementary table S3d Discovery of novel miRNAs in roots of *Arabidopsis*

1. Degradome supported cutsite(s) in Genome:17825730/17825821
PremiRNA Locus in Genome:>Chr1(17825725-17825824)_plus strand
PremiRNA sequence:AUGCAUUGAAAGUGACUACAUCGGGGUUCCGAUUUUUUUUGUUCUUCAUAUGAUGAAGCGGAAACAGUAAUCAACCCUGGUUUAGUCACUUUCACUGCAU
PremiRNA secondary structure:(((((.((((((((((((.(((((((((..((((....(((((((((((...))))))....))))).))))))))))))).))))))))))).)))))
Degradome supported cutsite(s) in premiRNA:6/97
miRNA sequence and Count(AGO1/wt):UUGAAAGUGACUACAUCGGGG_9452.95/1002.26
miRNA Position in PremiRNA:6-26
miRNA* sequence and Count(AGO1/wt):UGGUUUAGUCACUUUCACUGC_1.45/0.18
miRNA Position in PremiRNA:77-97
miRNA annonation:miRNA-1

2. Degradome supported cutsite(s) in Genome:19415175
PremiRNA Locus in Genome:>Chr2(19415069-19415180)_plus strand
PremiRNA sequence:GUAUUACAUGUUUUGUGCUUGAAUCUAAUUCAACAGGCUUUAUGUAAGAGAUUCUUUAACAAUUCCUAUAAUCUUUGUUGUUGGAUUAGAUUCACGCACAAACUCGUAAUCU
PremiRNA secondary structure:(.(((((..((((.((((.((((((((((((((((.((.....)).(((((((................)))))))..))))))))))))))))).))))))))..))))).)
Degradome supported cutsite(s) in premiRNA:107
miRNA sequence and Count(AGO1/wt):UUAGAUUCACGCACAAACUCGU_1316.62/260.23
miRNA Position in PremiRNA:86-107
miRNA* sequence and Count(AGO1/wt):AUGUUUUGUGCUUGAAUCUAAUU_0/0
miRNA Position in PremiRNA:8-30
miRNA annonation:miRNA-2

3. Degradome supported cutsite(s) in Genome:15371933
PremiRNA Locus in Genome:>Chr3(15371931-15372073)_plus strand
PremiRNA sequence:UCAAAUGAGUUGAUGGGUCAAAUGAGUUAUUGGGUCAAUUGGUUUGAUGAAUAAAAUGAGUUGGGUUGUAAUGAUUAAUGGUUUCAAUGGUUUACCCAAUUAACCCAUUAACCCAUAACUCAUUUAACCCUAAACUCAUUUGA
PremiRNA secondary structure:((((((((((...((((.(((((((((((.((((.(((.((((..............((((((((...(((.(((.........))).))).))))))))))).))).)))))))))))))).)))))..))))))))))
Degradome supported cutsite(s) in premiRNA:3

miRNA sequence and Count(AGO1/wt):AAAUGAGUUGAUGGGUCAAAUGAG_0.21/1.85
miRNA Position in PremiRNA:3-26
miRNA* sequence and Count(AGO1/wt):UUUAACCCUAAACUCAUUUGAUU_1.03/0.18
miRNA Position in PremiRNA:121-143
miRNA annonation:miRNA-3

4. Degradome supported cutsite(s) in Genome:29911709
PremiRNA Locus in Genome:>Chr1(29911713-29911101)_minus strand
PremiRNA
sequence:UACUUCGGCGAUGAUGAUGAAACAAGAUUACCAUCAAACUUUGAGAGGUUUUUCGCUCGUUGAUGUAAACUUCUUUUUAUAAUCUGAGCCU
AGUUUGUUUGUUUGAAAUCUUAUGUGUUACAUGUUAUAUGAAAUCGUUUUGGUGAUAGGAAAGGCAACAAAUUAGAGUCUCUGAUUCACUUAUGUC
AAUAGUGGAUAAAGCGCUGUACGUUGCCGAGAUAGAUAUGGCGAUAUUCUGGUACUUGGACUAGUUUUGAUAGUUCCAUGUGCAUUCAAAAGCCCU
UACAUGUUUCUCUCAACUUAUUCCCAUUAGUAUAUGUAUUUUGAUUUGGUAAAGGCAACAAAUUAGAGUCUCUGAUUCACUUAUUCAAUAGUGGAU
AAAGCGCUGUACGUUGCCAAUAAUUACUUGGUCCAAAGAUCUCCCAAUCGGCUUUCAGUCUUAUUUUAGCCGAUAUGAUCGAUGUUCCUAAAUUAUA
GAUUUUGUUACGUGUUUUCAUAUAGCUGAGCUGACCAUAUCAUGCUAUUUACCAAAUCUUUUUGAUCAAUUACCUGACACAUUGACAGACGAUCGAU
UUUUAGACACUGAAAUAUGGGCCGAAAUUUUGAAGGAGUA
PremiRNA secondary
structure:((((((..(((.(((.((((((.(((.((((.(((((....((((.((....)).))))))))))))).)))..)))))).))).(.(((((..(((.(((((((...((..((((((((...((((((((((.((.....(((((((((..((((((.((((((((.((((((((...........((((((.......))))))..((
((((((((.(((((((((.........((((.(((((((.......))).)))).))))........)))).....)))))).)))).........(((..((((....))))......)))....)))))).)))))))).))))))))..........((((((.......))))))..)))))).((((((.....((..((..((((.....(((((((............
..))))))..))))..))..))..))))))...)))))))).)).)))))))))..((((((.....))).)))......((((....))))..........))))))).))))))))).))).((((((...))))))..))))))......)))..))))))
Degradome supported cutsite(s) in premiRNA:6
miRNA sequence and Count(AGO1/wt):CGGCGAUGAUGAUGAAACAAGAU_2.89/0.37
miRNA Position in PremiRNA:6-28
miRNA* sequence and Count(AGO1/wt):CUUCUUUUUAUAAUCUGAGCCUA_0/0.18
miRNA Position in PremiRNA:70-610
miRNA annonation:miRNA-5

5. Degradome supported cutsite(s) in Genome:23988505
PremiRNA Locus in Genome:>Chr5(23988591-23988477)_minus strand
PremiRNA
sequence:UGCUGAUGCAGCAUCAUCAAGAUUCCCACUCGAUAAGGAACCCUUUUCUGCGGAUCGAGAGGCGAAAGCAAAGGGAACCACAUCUCUUGUG
AAUCUUAAUGGUGCUGCAUCUGCG
PremiRNA secondary structure:(((.((((((((((((.(((((((((.((((((.(((((....)))))....))))))..((....))...))))..((((.....)))).))))).))))))))))))).)))
Degradome supported cutsite(s) in premiRNA:88

miRNA sequence and Count(AGO1/wt):UGAAUCUUAAUGGUGCUGCAU_1.24/0
miRNA Position in PremiRNA:90-110
miRNA* sequence and Count(AGO1/wt):UGCAGCAUCAUCAAGAUUCCC_0.83/0.18
miRNA Position in PremiRNA:8-82
miRNA annonation:miRNA-6

6. Degradome supported cutsite(s) in Genome:6220763/6220784
PremiRNA Locus in Genome:>Chr1(6220685-6220789)_plus strand
PremiRNA
sequence:UGAAGUAAAGCUGCUAAGCUAUGGAUCCCAUAAGCCUUAUCAAAUUCAAUAUAAUUGAUGAUAAGGUUUUUUUUAUGGAUGCCAUAUCUCA
GGAGCUUUCACUUA
PremiRNA secondary structure:(((.((((((((.((.((.(((((...((((((((((((((...(((((...))))))))))))))).....))))))...))))).)).)).))))))).)))))
Degradome supported cutsite(s) in premiRNA:79/100
miRNA sequence and Count(AGO1/wt):UGCCAUAUCUCAGGAGCUUUC_0.41/0.18
miRNA Position in PremiRNA:80-100
miRNA* sequence and Count(AGO1/wt):AAGCUGCUAAGCUAUGGAUCC_0/0.18
miRNA Position in PremiRNA:8-28
miRNA annonation:miRNA-7

7. Degradome supported cutsite(s) in Genome:8770553
PremiRNA Locus in Genome:>Chr5(8770544-8770622)_plus strand
PremiRNA sequence:UCAAAUUAAAUGAGUUAUGGGUUGACCCAACUCAUUUUGUUAAAUGGGUUGGGUCAACCCAUAACUCAUUUAACCCUAA

PremiRNA secondary structure:.....((((((((((((((((((((((((((((((((....))))))))))))))))))))))))))))))))......
Degradome supported cutsite(s) in premiRNA:10
miRNA sequence and Count(AGO1/wt):UAAAUGAGUUAUGGGUUGACCC_0.41/0.18
miRNA Position in PremiRNA:2-28
miRNA* sequence and Count(AGO1/wt):UCAACCCAUAACUCAUUUAAC_0.62/0
miRNA Position in PremiRNA:54-74
miRNA annonation:miRNA-8

8. Degradome supported cutsite(s) in Genome:20772272
PremiRNA Locus in Genome:>Chr1(20772225-20772297)_plus strand

PremiRNA sequence:UGCUUCUUUGUCUACACUUUUGGAAAAGGUGAUGAUAUCAUUGCUUUUCCCCAAAUGUAGACAAAGCAAUACC

PremiRNA secondary structure:))...(((((((((((..(((((.(((((..(((....)))..)))))..))))))))))))))))).....))
Degradome supported cutsite(s) in premiRNA:48
miRNA sequence and Count(AGO1/wt):UCCCCAAAUGUAGACAAAGCA_84.62/20.75
miRNA Position in PremiRNA:48-68
miRNA* sequence and Count(AGO1/wt):CUUUGUCUACACUUUUGGAAA_0.25/0.4
miRNA Position in PremiRNA:6-26
miRNA annonation:miRNA-9

9. Degradome supported cutsite(s) in Genome:23988566
PremiRNA Locus in Genome:>Chr5(23988478-23988590)_plus strand
PremiRNA
sequence:GCAGAUGCAGCACCAUUAAGAUUCACAAGAGAUGUGGUUCCCUUUGCUUUCGCCUCUCGAUCCGCAGAAAAGGGUUCCUUAUCGAGUGGGA
AUCUUGAUGAUGCUGCAUCAGC
PremiRNA secondary structure:((.(((((((((.(((((((((((.((...((((.((..((((((((..(((.....)))...)))...)))))..)).))))...)).))))))))))).))))))))).))
Degradome supported cutsite(s) in premiRNA:89
miRNA sequence and Count(AGO1/wt):UGCAGCACCAUUAAGAUUCAC_2.27/0
miRNA Position in PremiRNA:6-26
miRNA* sequence and Count(AGO1/wt):GAAUCUUGAUGAUGCUGCAU_14.26/15.35
miRNA Position in PremiRNA:90-110
miRNA annonation:miRNA-12

10. Degradome supported cutsite(s) in Genome:16432049
PremiRNA Locus in Genome:>Chr1(16432023-16432076)_plus strand
PremiRNA sequence:CCCAUCAGAACUCCGCAGUUAAGCGCGCUUGGGCGAGAGUAGUACUAGAAUGGG
PremiRNA secondary structure:((((((((.(((((((...((((....)))).))).))))....)).).)))))
Degradome supported cutsite(s) in premiRNA:27
miRNA sequence and Count(AGO1/wt):CAGAACUCCGCAGUUAAGC_0.21/2.4
miRNA Position in PremiRNA:6-24
miRNA* sequence and Count(AGO1/wt):CUUGGGCGAGAGUAGUACUAG_4.55/9.06
miRNA Position in PremiRNA:29-50
miRNA annonation:miRNA-13

11. Degradome supported cutsite(s) in Genome:22165414

PremiRNA Locus in Genome:>Chr1(22165407-22165553)_plus strand

PremiRNA sequence:AGAGUUUUAAAAGAUUUACAAGGGAUUUUAAAAAGUUUUAAAAAGAUUUACAAGAGUUUUUAAAGGGUCUUCUUGUAAAUUCUUUAAGAAACUCUUGUACAUCUUUUAAAACCCUUUUAAAAUCUCUUGUAAAUCUUUUAAAACCCU

PremiRNA secondary structure:((.((((((((((((((((((((((((((((((.(((((((((.(((.((((((((((((((((((((..........))))))).))))))))))))).))))))))))))..)))))))))))))))))))))))))))))).))

Degradome supported cutsite(s) in premiRNA:8

miRNA sequence and Count(AGO1/wt):UUUUAAAAGAUUUACAAGGGAUUU_1.03/0

miRNA Position in PremiRNA:5-28

miRNA* sequence and Count(AGO1/wt):AUCUCUUGUAAAUCUUUUAAAAC_0/0.74

miRNA Position in PremiRNA:122-145

miRNA annonation:miRNA-14

12. Degradome supported cutsite(s) in Genome:16102366

PremiRNA Locus in Genome:>Chr3(16102362-16102418)_plus strand

PremiRNA sequence:AUGAUUUACAAGGGAUUUUAAAAGUGUCUACAUGGUUUACAAUCGUUUUUAAAGCAU

PremiRNA secondary structure:(((.((((.(((.((((.((((.(((....)))..)))).)))).))).)))).)))

Degradome supported cutsite(s) in premiRNA:5

miRNA sequence and Count(AGO1/wt):UUUACAAUCGUUUUUAAAGCA_1.24/0

miRNA Position in PremiRNA:36-56

miRNA* sequence and Count(AGO1/wt):UUUACAAGGGAUUUUAAAA_0/0

miRNA Position in PremiRNA:4-24

miRNA annonation:miRNA-15

13. Degradome supported cutsite(s) in Genome:16159377

PremiRNA Locus in Genome:>Chr3(16159338-16159398)_plus strand

PremiRNA sequence:AGCGGUUGAGUUCCCGAAGUUGUCUCUUGUAGUUGCUGAUGACUUUUAGAACACGAUAGCU

PremiRNA secondary structure:(((.((((.((((..(((((..((....((....)).))..)))))..)))).)))).)))

Degradome supported cutsite(s) in premiRNA:40

miRNA sequence and Count(AGO1/wt):UGAUGACUUUUAGAACACGAU_5.37/1.11

miRNA Position in PremiRNA:37-57

miRNA* sequence and Count(AGO1/wt):UGAGUUCCCGAAGUUGUCUCU_0.21/0.18

miRNA Position in PremiRNA:7-27

miRNA annonation:miRNA-16

14. Degradome supported cutsite(s) in Genome:8858956
PremiRNA Locus in Genome:>Chr4(8858895-8858978)_plus strand
PremiRNA sequence:CAACUUUAUUAGUCAAGUUUUAAAUUGUGGCCAUUUUAAACAUCAAGUUCAUAUGUGGCCAUUUAAAACAUGAACUAAACGUUG

PremiRNA secondary structure:((((....(((((((.(((((((...((((((((....(((.....))).....))))))))))))))).)).))))).))))
Degradome supported cutsite(s) in premiRNA:62
miRNA sequence and Count(AGO1/wt):UUUAAAACAUGAACUAAACGU_4.13/0
miRNA Position in PremiRNA:62-82
miRNA* sequence and Count(AGO1/wt):UUUAUUAGUCAAGUUUUAAAUUGU_0.21/0.18
miRNA Position in PremiRNA:5-29
miRNA annonation:miRNA-17

15. Degradome supported cutsite(s) in Genome:8770553
PremiRNA Locus in Genome:>Chr5(8770549-8770616)_plus strand
PremiRNA sequence:UUAAAUGAGUUAUGGGUUGACCCAACUCAUUUUGUUAAAUGGGUUGGGUCAACCCAUAACUCAUUUAA
PremiRNA secondary structure:((((((((((((((((((((((((((((((((....))))))))))))))))))))))))))))))))
Degradome supported cutsite(s) in premiRNA:5
miRNA sequence and Count(AGO1/wt):UAAAUGAGUUAUGGGUUGACCC_0.41/0.18
miRNA Position in PremiRNA:2-23
miRNA* sequence and Count(AGO1/wt):UCAACCCAUAACUCAUUUAAC_0.62/0
miRNA Position in PremiRNA:48-69
miRNA annonation:miRNA-19

16. Degradome supported cutsite(s) in Genome:12295343
PremiRNA Locus in Genome:>Chr5(12295232-12295369)_plus strand
PremiRNA
sequence:GAGUGCAAAGGGUAGAGUUAAGUUCUUGUAUUGGGACUGGAGUUGGGAUUACCAUUACAGCUUCAUUGUUAUACUCUGGGUAGAUGGGAU
CUUAUCUCUGUAUGCAUAGCUUGGGACUUACCUUUAGCAUUCUACUAA
PremiRNA secondary structure:((((((.((((((.((((((((((..(((((....((.((((.(((((((.(((((...(((.((..(.....)..))))).)))))))))))).))))))))))).))))).)))))))))))).))))))......
Degradome supported cutsite(s) in premiRNA:112
miRNA sequence and Count(AGO1/wt):UGGGACUUACCUUUAGCAUUCU_0.21/0.55
miRNA Position in PremiRNA:112-133

miRNA* sequence and Count(AGO1/wt):UGCAAAGGGUAGAGUUAAGUUC_0/0
miRNA Position in PremiRNA:2-22
miRNA annonation:miRNA-20

17. Degradome supported cutsite(s) in Genome:16661145
PremiRNA Locus in Genome:>Chr5(16661213-16661123)_minus strand
PremiRNA
sequence:UAAUUGACUCUCGACUCAUUCAUCCAAAUACCAAAUGAAAGAAUUUGUUCUCAUAUGGUAAAUGAAUGAAUGAUGCGAGAGACAAAUUGA

PremiRNA secondary structure:...(((.((((((..((((((((.((..(((((.((((..(((....))))))).)))))..)).))))))))..)))))).)))....)
Degradome supported cutsite(s) in premiRNA:69
miRNA sequence and Count(AGO1/wt):AAUGAAUGAUGCGAGAGACAA_40.56/20.95
miRNA Position in PremiRNA:69-86
miRNA* sequence and Count(AGO1/wt):GACUCUCGACUCAUUCAUCCA_1.25/7.45
miRNA Position in PremiRNA:6-22
miRNA annonation:miRNA-21

18. Degradome supported cutsite(s) in Genome:22322724
PremiRNA Locus in Genome:>Chr5(22322903-22322720)_minus strand
PremiRNA
sequence:AAGCCUUGAUGAUUCGACAAAGUGAAGGGUUUGGUCUUCAGAAAGUUUUCUUGCAAGGUUCAAGAACGGAUCCUCAAAAUUCAAACUGUUU
CUCACCAAUUUGAAUUGUGAAGAUCCGUUCUUGAACCUUGGAAGAAAACUUUCUGGAGACCAAACCCUUCACUUUGUCGAGUCACCAAGGCUU

PremiRNA secondary
structure:((((((((.(((.(((((((((((((((((((.(((.((((((((.((........)).)))))))).))).))))))))))))))))))).))).))))))))
Degradome supported cutsite(s) in premiRNA:181
miRNA sequence and Count(AGO1/wt):UUGAUGAUUCGACAAAGUGAA_487.64/60.11
miRNA Position in PremiRNA:6-26
miRNA* sequence and Count(AGO1/wt):CACUUUGUCGAGUCACCAAGG_0.21/0.92
miRNA Position in PremiRNA:161-181
miRNA annonation:miRNA-22

19. Degradome supported cutsite(s) in Genome:771406
PremiRNA Locus in Genome:>Chr2(771517-771382)_minus strand

PremiRNA
sequence:GAAGACACUGAAGGACCUAAACUAACAAAGGUAAACGGCUCAGUGUGCGGGGUAUUACACUCGGUUUAAUGUCUGAAUGCGAUAAUCCGCA
CGAUGAUCUCUUUAUCUUUGUUUGUUUAGGUCCCUUAGUUUCUUC
PremiRNA secondary structure:(((((.(((((.((((((((((.((((((((((((.((.(((.(((((((..(((..((.((((........)))).))..)))..))))))).))).)).)))))))))))).)))))))))).))))).)))))
Degradome supported cutsite(s) in premiRNA:113
miRNA sequence and Count(AGO1/wt):CACUGAAGGACCUAAACUAAC_11.57/1.66
miRNA Position in PremiRNA:6-26
miRNA* sequence and Count(AGO1/wt):UUGUUUAGGUCCCUUAGUUUC_17.36/5.92
miRNA Position in PremiRNA:113-133
miRNA annonation:miRNA-23

20. Degradome supported cutsite(s) in Genome:25784115
PremiRNA Locus in Genome:>Chr5(25784115-25784187)_plus strand
PremiRNA sequence:GGUUUCGUGGUGUAGUUGGUUAUCACGUCAGUCUAACACACUGAAGGUCUCCGGUUCGAACCCGGGCGAAGCC

PremiRNA secondary structure:((((((((((..((..((..(((...(((((.......))))).)))..))..))...)))...))))))))
Degradome supported cutsite(s) in premiRNA:1
miRNA sequence and Count(AGO1/wt):GGUUUCGUGGUGUAGUUGGU_0.41/7.21
miRNA Position in PremiRNA:1-20
miRNA* sequence and Count(AGO1/wt):UCCGGUUCGAACCCGGGCGAAGCC_0/0
miRNA Position in PremiRNA:52-75
miRNA annonation:miRNA-24

21. Degradome supported cutsite(s) in Genome:8277186
PremiRNA Locus in Genome:>Chr1(8277133-8277214)_plus strand
PremiRNA sequence:GUUUCAUCGCUUGUAUAUUUAAGAAAUUUGGAAAAAUAUUGAUUUUAUUAAAUUGGUUUAGAUGUGUUGGUUAAACUUAAAU

PremiRNA secondary structure:((((....(((..((((((((((.(((((((.(((((....))))).)))))))..))))))))))..))).))))......
Degradome supported cutsite(s) in premiRNA:54
miRNA sequence and Count(AGO1/wt):UUUAGAUGUGUUGGUUAAACU_0.62/0
miRNA Position in PremiRNA:57-77
miRNA* sequence and Count(AGO1/wt):UUUCAUCGCUUGUAUAUUUAAGAA_0/0
miRNA Position in PremiRNA:2-25
miRNA annonation:miRNA-28

22. Degradome supported cutsite(s) in Genome:19659579

PremiRNA Locus in Genome:>Chr3(19659673-19659574)_minus strand

PremiRNA sequence:ACUGUCGUCUCGGUUCGCGAUCCACAAGUAAUCUUUUGUGGAGAUUAUGAAAACAAUCAUCACGAGAGGCUACGUGUGGGUGGCAAACAAAGACGACAAU

PremiRNA secondary structure:(.((((((((..(((.((.(((((((.(((..((((((((..((((.(....).))))..))))))))..))).))))))).)).)))..)))))))).)

Degradome supported cutsite(s) in premiRNA:96

miRNA sequence and Count(AGO1/wt):UGUGGGUGGCAAACAAAGACG_0/0

miRNA Position in PremiRNA:75-95

miRNA* sequence and Count(AGO1/wt):UCUCGGUUCGCGAUCCACAAG_2.27/0.18

miRNA Position in PremiRNA:8-28

miRNA annonation:miRNA-30
